# Versatile synthesis and enlargement of functionalized distorted heptagon-containing nanographenes†

**DOI:** 10.1039/c6sc02895k

**Published:** 2016-08-31

**Authors:** Irene R. Márquez, Noelia Fuentes, Carlos M. Cruz, Virginia Puente-Muñoz, Lia Sotorrios, M. Luisa Marcos, Duane Choquesillo-Lazarte, Blanca Biel, Luis Crovetto, Enrique Gómez-Bengoa, M. Teresa González, Ruben Martin, Juan M. Cuerva, Araceli G. Campaña

**Affiliations:** a Departamento Química Orgánica, Universidad de Granada (UGR) C. U. Fuentenueva 18071 Granada Spain araceligc@ugr.es; b Departamento de Fisicoquímica, Facultad de Farmacia UGR. Cartuja Campus 18071 Granada Spain; c Departamento de Química Orgánica I, Universidad del País Vasco E-20018 San Sebastián Spain; d Departamento de Química, Universidad Autónoma de Madrid c/Francisco Tomás y Valiente no. 7, Cantoblanco 28049 Madrid Spain; e Laboratorio de Estudios Cristalográficos, Instituto Andaluz de Ciencias de la Tierra (CSIC-UGR) 18100 Armilla Granada Spain; f Departamento de Electrónica y Tecnología de Computadores, Facultad de Ciencias, CITIC, UGR E-18071 Granada Spain; g Fundación IMDEA Nanociencia, Ciudad Universitaria de Cantoblanco E-28049 Madrid Spain; h Institute of Chemical Research of Catalonia (ICIQ), Catalan Institution for Research and Advanced Studies (ICREA) Spain

## Abstract

Highly distorted polycyclic aromatic hydrocarbons (PAHs) are predicted to be attractive goals in nanoscience owing to the new properties they can exhibit. We have shown that a variety of functionalized distorted heptagon-containing nanographenes can be easily prepared from simple building blocks by a sequence of Co-catalyzed cyclotrimerization and cyclodehydrogenation reactions. The versatility of this strategy allows easy subsequent enlargement of these nanostructures by Ni-catalyzed cross-coupling and final cyclodehydrogenation reactions. Soluble extended distorted nanographenes 1 and 2 containing heptagon and an edge-shared pentagon–heptagon combination have been synthesized. High distortion of the polycyclic backbone of 2 caused by non-hexagonal rings and a helicene moiety was confirmed by X-ray crystallography. Experimental data reveal promising optical and electronic properties for distorted PAHs with long fluorescence lifetimes (up to 14.5 ns) and low band gaps (down to 2.27 eV). This straightforward and versatile synthetic strategy, the observed long fluorescence lifetimes and the small optical and electrochemical band gaps for the presented compounds may promote the future implementation of distorted graphene molecules in electronic devices.

## Abstract 2

Straightforward path to curved graphene molecules: distorted polycyclic aromatic hydrocarbons including heptagon moieties are obtained from simple precursors.

## Graphical abstract

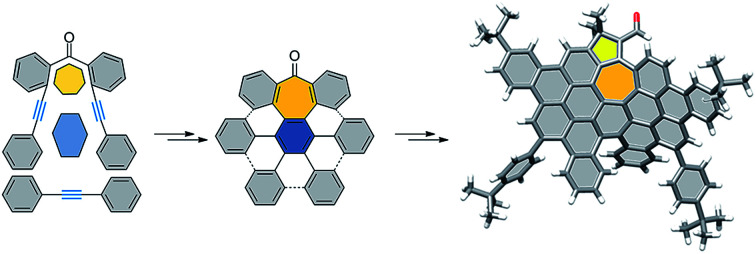

## Introduction

Planar polycyclic aromatic hydrocarbons (PAHs) such as nanographenes and graphene nanoribbons (GNRs) have received significant attention in recent years.^1^ PAHs are attractive materials for nanoscale electronic devices due to their tunable non-zero bandgaps, which are principally controlled by their size and edges. Microscopy studies have shown that graphene samples usually include non-hexagonal rings such as pentagons and heptagons, thus leading to curved carbon based nanostructures^2^ which can significantly modify the original properties.^3^ Particularly, typical topological defects in large area graphene films are disclinations, as individual pentagons or heptagons, and dislocations or grain boundaries, as pairs of pentagons and heptagons, that result in energetically favourable structures.^4^ Unlike commonly studied pentagon-containing PAHs, such as corannulene and its derivatives, which have been extensively synthesized and investigated,^5^ graphene-type molecules containing seven-membered rings are much less common and only very few examples have been reported.

The first synthesis of [7]circulene was reported in 1983 showing a non-planar saddle-shaped geometry.^6^ After these pioneering examples reported by Yamamoto and Nakazaki, graphene molecules incorporating heptagons remained forgotten until 2012, when Miao and co-workers reported the synthesis of curved polycyclic aromatic molecules.^7*a*^ In this case, the introduction of a seven-membered ring in an isoelectronic to planar hexabenzocoronene compound forces the structure out of planarity, increases solubility and enhances fluorescence. Later on, in 2015, they synthesized new saddle-shaped polycyclic arenes containing two heptagons showing a deep distorted structure but with drastically diminished optical properties.^7*b*,*c*^ In 2013, Durola and co-workers unexpectedly obtained an unusual hexa[7]circulene due to rearrangement during the Scholl reaction.^8*a*^ Another structure containing pentagon–heptagon moieties was reported by Kuck in 2012.^8*b*^

Scott, Itami and co-workers made a major breakthrough in 2013, synthesizing a grossly warped nanographene with five embedded heptagons around a central pentagon.^9^ The X-ray crystal structure confirmed the distortion caused by the odd-membered rings, drastically enhancing solubility in organic solvents. Comparison with a purely hexagonal nanographene shows that the distortion of the planar sheet affects its electronic and optical properties. The distorted compound exhibits a wider band gap, higher fluorescence and easier oxidation/reduction than the defect-free nanographene. An in depth study of these unconventional heptagon-containing PAHs is still limited owing to the absence of a general, efficient and versatile method for their synthesis. At present, these curved graphene molecules are prepared either from heptagon-containing precursors or by generating the seven-membered rings through cyclodehydrogenation or Friedel–Craft reactions. Although these approaches have no doubt resulted in interesting and exotic carbon nanostructures, such strategies unfortunately lack flexibility and versatility.

**Scheme 1:**
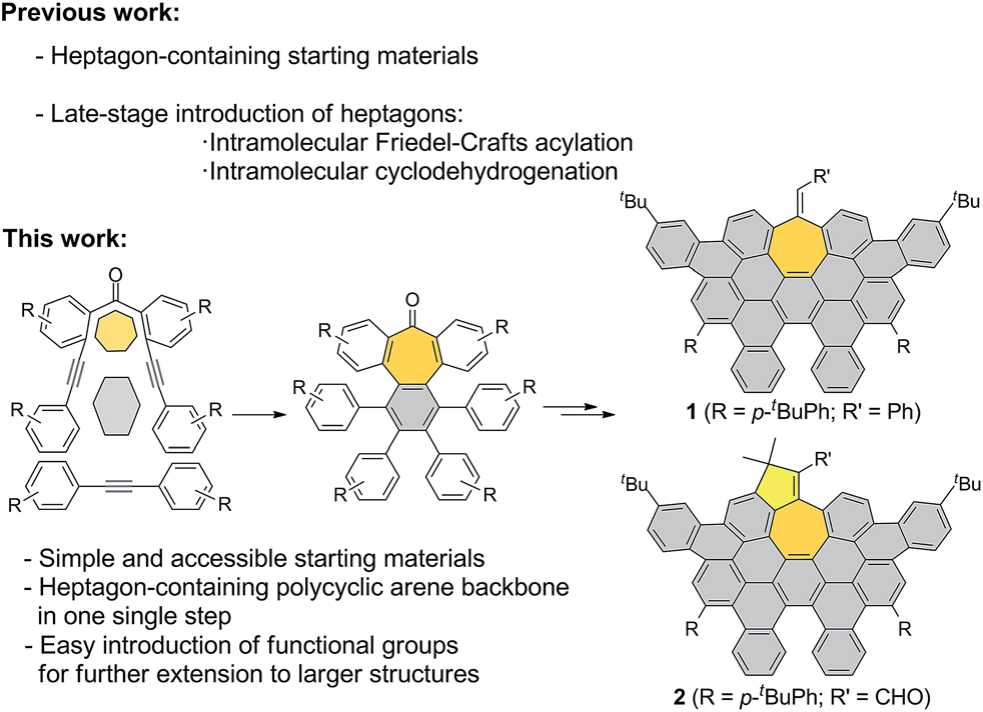
General scheme, important features of synthetic approach, and structures of distorted expanded nanographenes prepared, 1 and 2.

Here, we describe a new and versatile synthetic route to rapidly prepare rather elusive distorted nanographenes containing seven-membered rings *via* intermolecular Co(0)-mediated cyclotrimerization reaction followed by cyclodehydrogenation events from simple acyclic precursors, thus representing a unique tool for rapidly creating molecular diversity (Scheme 1). Interestingly, we have successfully implemented Ni-catalyzed cross-coupling reactions of aryl methyl ethers *en route* to unprecedented all-carbon distorted nanographenes, thus allowing for counterintuitive strategies for preparing graphene-type motifs.

## Results and discussion

### Synthetic methodology and structural characterization

We started our investigations by designing a route to 1 (Scheme 1). Although a variety of structurally well-defined expanded nanographenes and graphene nanoribbons (GNRs), exhibiting different widths, lengths, edge structures and heteroatom doping, have been previously synthesized,^10^ to the best of our knowledge, compounds 1 and 2 constitute new examples of distorted expanded nanographenes containing a heptagon at the edge of the structure. Moreover, compound 2 has a very unusual polycyclic arene backbone containing adjacent five- and seven-membered carbocycles.^8*b*^

**Fig. 1:**
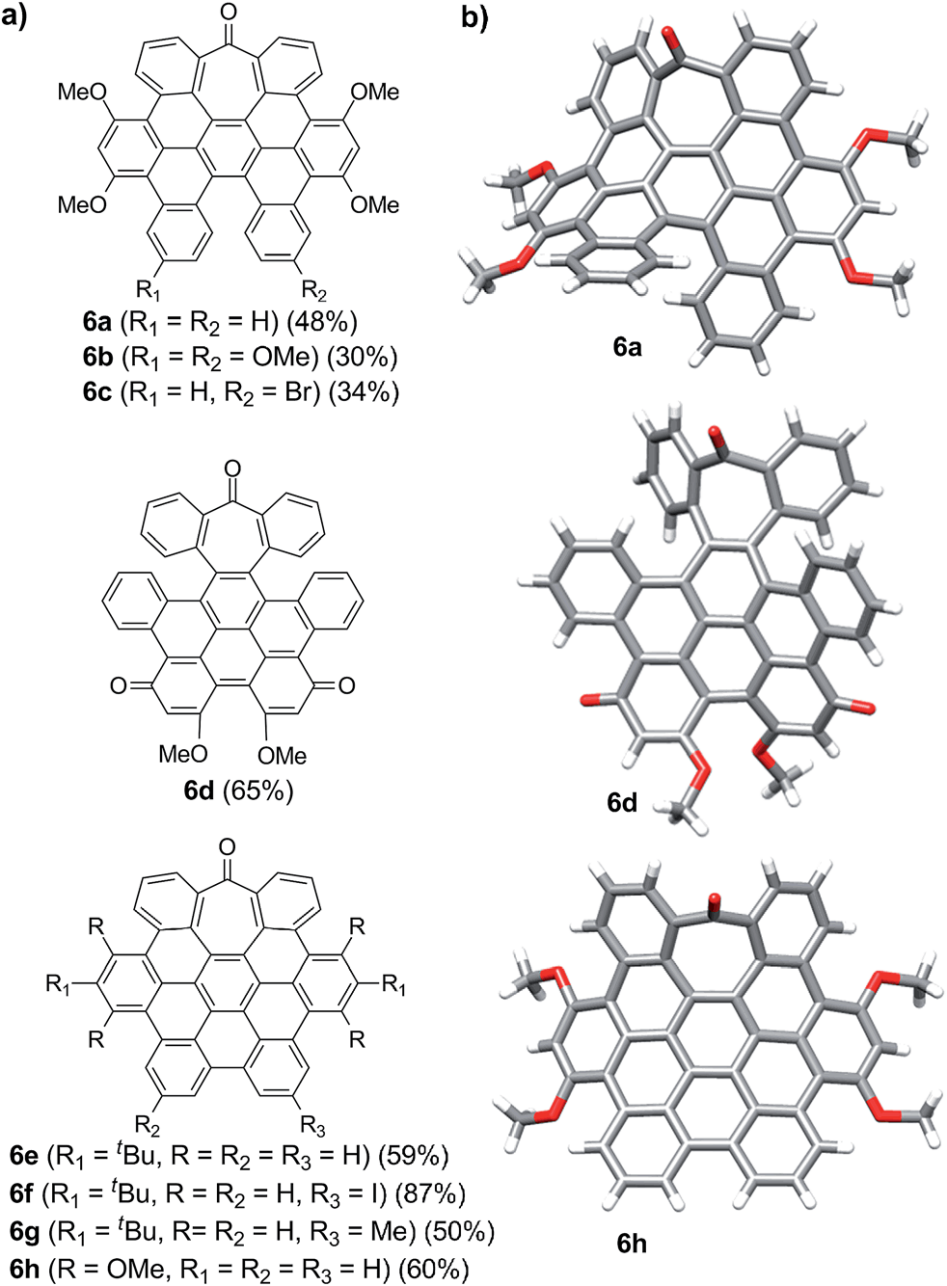
(a) Heptagon-containing nanographenes. (b) Single crystal X-ray diffraction structures of 6a, 6d and 6h. Color code: C: gray, O: red, H: white.

To such an end, we studied the key cyclotrimerization using dialkynes 3 (Table 1) that were easily prepared from simple precursors *via* Sonogashira–Hagihara coupling reactions. The presence of the carbonyl group hindered the formation of undesired 9,10-dihydroanthracenes^11^ while allowing for subsequent functionalization techniques, as recently shown by Miao and co-workers.^7*b*^ We found that a wide variety of functionalized heptagon-containing polyphenylenes 5a–o could easily be obtained by using Co(0)-catalyzed intermolecular cyclotrimerization reactions. Noteworthily, the cyclotrimerization reaction tolerates substitution in both coupling partners. Substitution in the benzophenone moiety in dialkynes 3 affords compounds such as 5b. Dialkynes 3 can also incorporate *meta*- or *para*-substituted ethynylbenzenes affording the corresponding functionalized products 5c–f. *Meta*- and *para*-substituted diphenylacetylenes 4 are also allowed as in compounds 5g–j. Furthermore, compounds 5k–n are relevant examples of cyclotrimerization with substitution in both building blocks simultaneously. The versatility of the strategy is also illustrated in compound 5o incorporating two thiophene moieties as aromatic rings. With a reliable technique *en route* to seven-membered carbocycles in hand, we envisioned that the distorted-PAHs could be prepared by oxidative cyclodehydrogenation, thus accessing heptagon-containing nanographenes (Fig. 1). The presence of methoxy groups allowed controlling of the regioselectivity in the oxidative aromatic coupling when using 2,3-dichloro-5,6-dicyano-*p*-benzoquinone (DDQ) as an oxidant in acidic media (MeSO_3_H).^12^

**Table 1:** General cyclotrimerization reaction and compounds prepared.

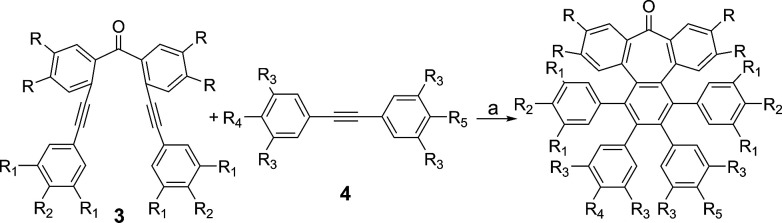
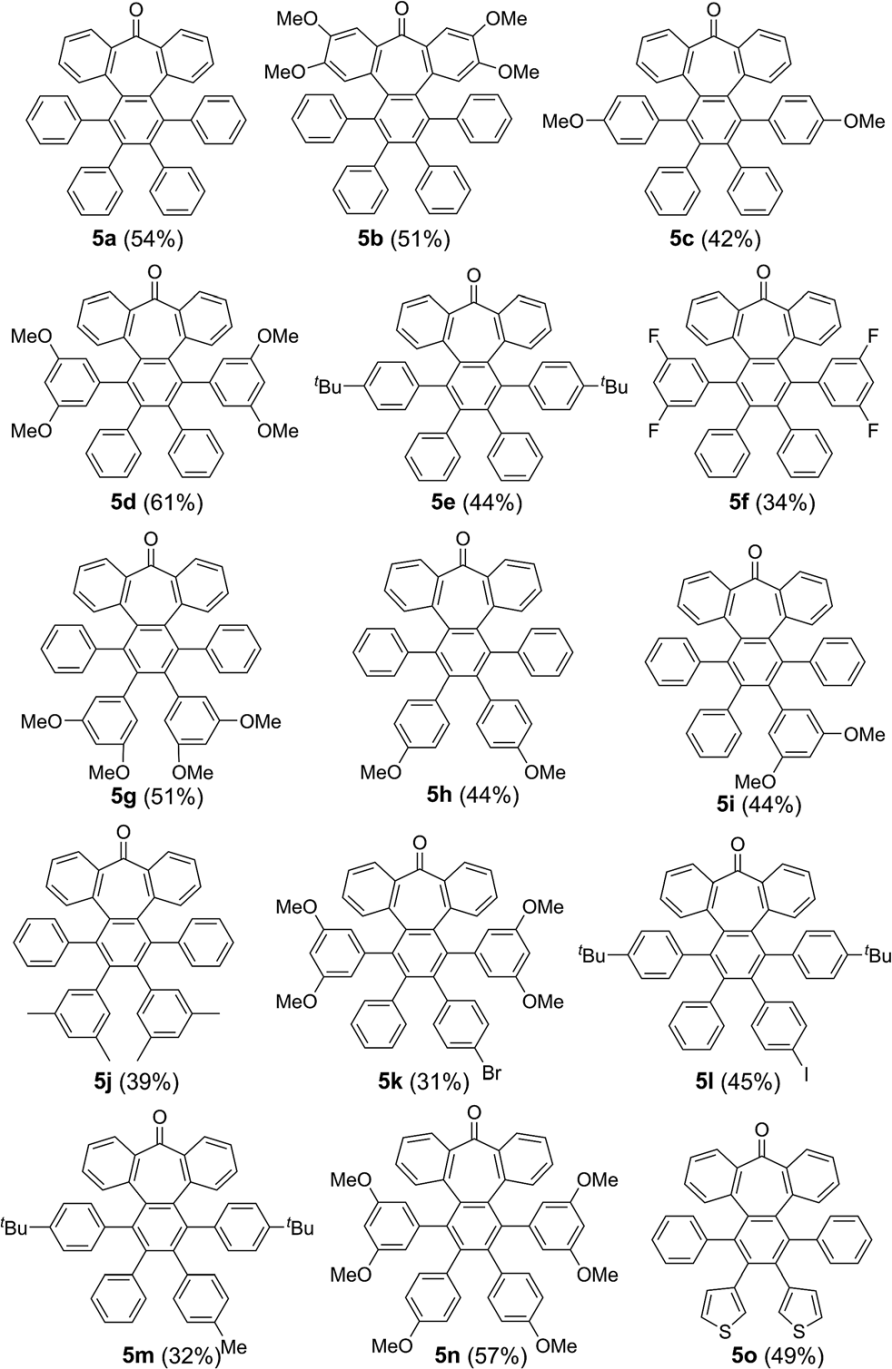

a Dialkyne 3 (0.1 mmol), diphenylacetylene 4 (0.15 mmol), Co_2_(CO)_8_ (0.13 mmol), 1,4-dioxane (6 mL), 100 °C, 16 h

Intriguingly, we observed partial demethylation and oxidation during the cyclodehydrogenation of 5g, yielding exclusively 6d.^13^ Remarkably, incomplete cyclodehydrogenation led to doubly distorted compounds such as 6a and 6d, incorporating both the heptagon and [5]-helicene motifs at different positions of the aromatic backbone. Both compounds, 6a and 6d, were characterized by X-ray crystallography (Fig. 1b), confirming their highly distorted structure. Hence, these compounds constitute a new family of structures within the helical nanographene arena.^7*a*,14^ In contrast to the presence of methoxy groups, less activated *tert*-butylated compounds resulted in being unchanged under similar conditions. In these cases, however, the combination DDQ/CF_3_SO_3_H was required and fully cyclodehydrogenated compounds 6e–g were isolated in good yields while forming five new C–C bonds. These distorted nanographenes can be considered as coronene analogues containing a seven-membered carbocycle in the otherwise hexagonal network. Similar nanographene 6h, incorporating methoxy groups, can be obtained by treatment of 6a with FeCl_3_ in MeNO_2_/DCE (60 °C, 16 h). In this case, the molecular packing of 6h in the X-ray crystal structure exhibits π–π interaction between molecules. The distance between two curved π-faces is 3.8 Å, measured from the distance between two centroids of benzene rings.

The presence of π-interactions between molecules in these distorted nanographenes could support their application as organic semiconductors in the solid state. In this sense, we performed the calculation of the electronic and transport properties of the relaxed stacked 6h structure, following the stacking data obtained by X-ray measurements and relaxing the chain coordinates *via* total energy minimization until changes in the forces were below 0.02 eV Å^−1^. In this manner, the calculated band structure possesses a gap of 1.71 eV (see ESI, Fig. S6† for the calculated conductance, density of states and band structures of the stacked 6h nanographenes). In order to elucidate the influence of the defect on the transport and electronic properties of the 6h nanographenes, a similar analysis was performed for a related planar coronene, resulting in a close gap value of 1.75 eV (see ESI, Fig. S7†). Taking into account its high solubility, crystallinity in the solid state, and the calculated gap value, we anticipate that 6h could be a promising candidate for the future development of printable organic field-effect transistors (OFETs).

**Scheme 2:**
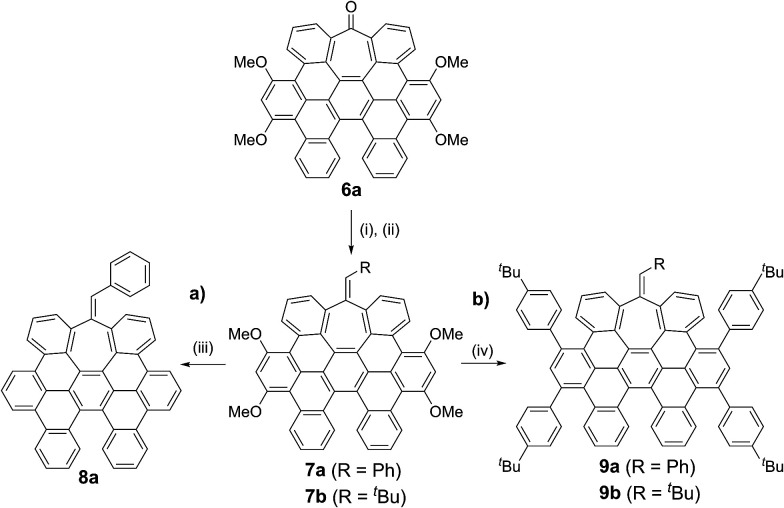
Subsequent transformations of heptagon-containing nanographene 6a: (a) synthesis of all-carbon distorted nanographene 8a containing both heptagon and [5]-helicene motifs. (b) Ni-Catalyzed KTC coupling in distorted nanographenes. Reaction conditions: (i) PhCH_2_MgCl (7a) or *t*BuCH_2_MgCl (7b), THF, RT, 2 h. (ii) SOCl_2_, Py_anh_, RT, 1 h, 54% (7a), 47% (7b) (2 steps). (iii) Ni(cod)_2_, PCy_3_, TMDSO, toluene, 100 °C, 16 h, 80%. (iv) Ni(cod)_2_, PCy_3_, *p*–*t*BuPhMgBr, toluene, 100 °C, 16 h, 79% (9a), 59% (9b).

While the inclusion of methoxy substituents is required for the cyclodehydrogenation event, we wondered whether these motifs could be utilized as coupling partners *via* C–OMe cleavage.^15^ Prompted by our recent work on Ni-catalyzed hydrogenolysis of aryl methyl ethers,^16^ we could easily obtain all-carbon heptagon-containing distorted nanographene 8a in 80% yield upon exposure of 7a under a Ni(cod)_2_/PCy_3_ regime, using tetramethyldisiloxane (TMDSO) as the reducing agent (Scheme 2a).

This result is particularly noteworthy, as producing rather elusive all-carbon doubly distorted nanographenes is now within reach, representing a testament of the prospective impact of the Ni-catalyzed C–OMe bond-cleavage reactions.

**Fig. 2:**
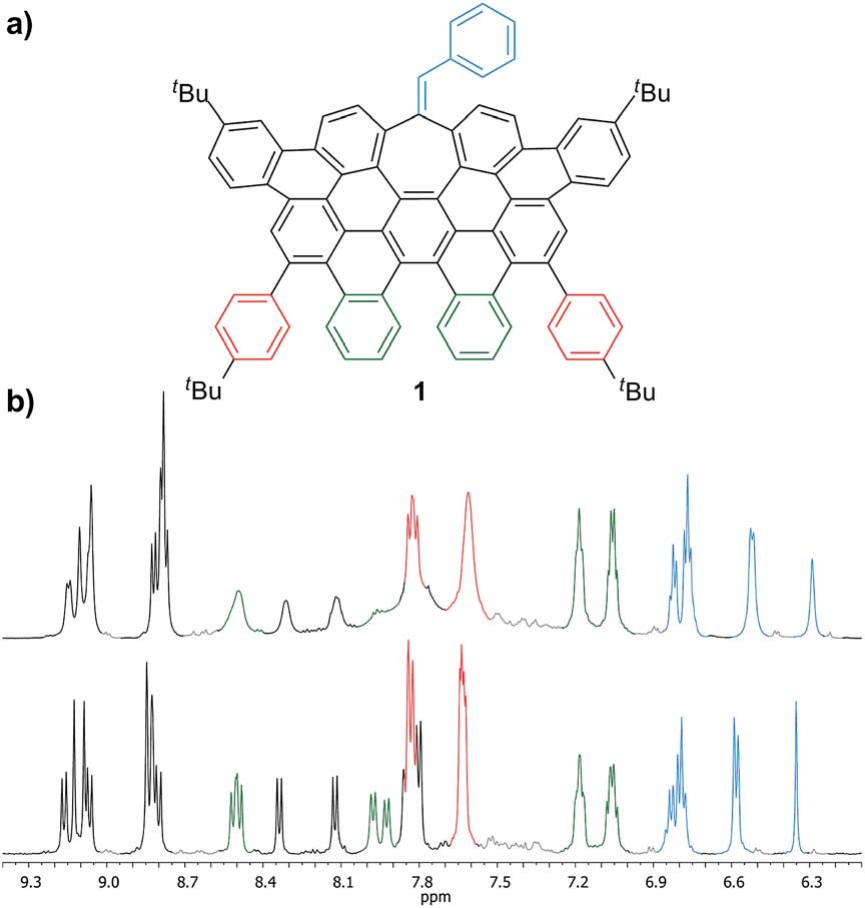
(a) Structure of 1 and (b) partial ^1^H-NMR spectra (500 MHz, C_2_D_2_Cl_4_) of 1 at: 293 K (top) and 369 K (bottom).

More interestingly, 7a and 7b were found to be competent in Kumada–Tamao–Corriu reactions *via* C–OMe cleavage,^17^ rapidly furnishing 9a and 9b in good yields (Scheme 2b). The direction chosen to enlarge the aromatic backbone means that the seven-membered carbocycle would be allocated in the middle and at the edge of the all-carbon structure, suggesting that these compounds could result in strong electron hole conductance asymmetry.^18^ Subsequently, 9a was submitted to oxidative dehydrogenation conditions with DDQ/MeSO_3_H in CH_2_Cl_2_, obtaining as the major product (47%) a highly distorted extended nanographene 1 that was firstly characterized by MALDI-TOF.^19^ As depicted in Fig. S82 (see ESI†) there is only one dominant peak in the mass spectrum of 1 and the isotopic distribution pattern of the high-resolution mass peak is in good agreement with the calculated patterns (Fig. S83, see ESI†). ^1^H-NMR spectroscopy of compound 1 showed broad signals at room temperature. This fact is not related to the solubility of the compound. The rotation of the pendant aromatic rings (Fig. 2, red aromatic rings) is slow at room temperature, yielding poorly resolved signals. VT-NMR spectroscopy in 1,1,2,2-tetrachloroethane-*d*_2_ at 369 K allowed us to unambiguously assign the distorted structure of 1 (Fig. 2b).

**Fig. 3:**
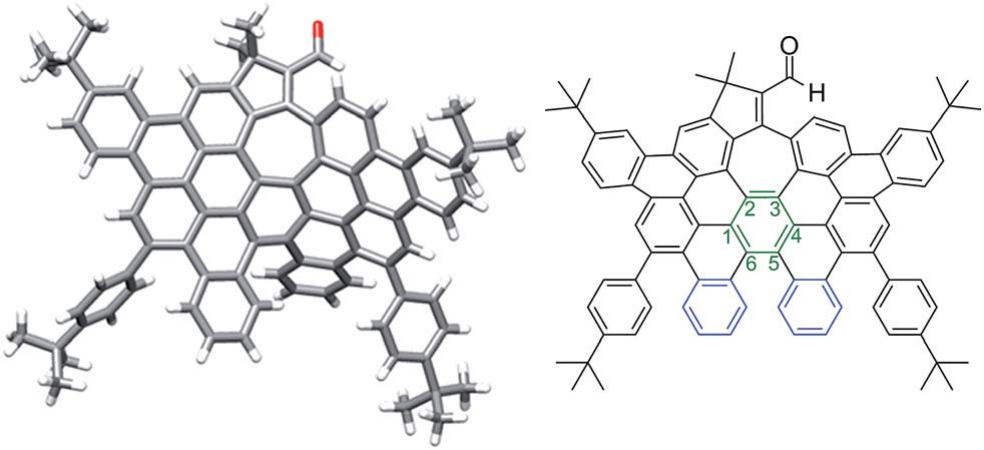
Distorted graphene molecule 2: single crystal structure (left) and corresponding ChemDraw structure (right).

More interestingly, a simple exposure of 9b under classical oxidative dehydrogenation conditions did not only result into two new aromatic rings, but also led to a new edge-sharing heptagon–pentagon combination at the border of the expanded graphene molecule 2. Compound 2, obtained in 44% yield, was characterized by MALDI-TOF and VT-NMR in DMSO-*d*_6_ at 348 K (see ESI, Fig. S84 and S87, respectively†). In this case, the structure of 2 was also confirmed by X-ray crystallography (Fig. 3), revealing that the π-backbone adopts a curved shape caused by the pentagon–heptagon unit and the chiral twisted conformation caused by the [5]-helicene moiety. The torsion angle in the helicene region resulted in 37° being the angle between the terminal helicene rings of 76° (Fig. 3, blue rings). We observed distorted benzene rings, with torsion angles up to 28° (Fig. 3, green ring) with the angle between the planes defined by carbon atoms 1–3 and 4–6 being 38° (see ESI, Fig. S1†). The huge distortion from planarity avoids strong π–π interactions between molecules and therefore enhances solubility in common organic solvents. The side *t*-butyl groups also improve solubility, where this molecule with 88 carbon atoms, a length of *ca.* 15.5 Å and width of *ca.* 9.7 Å, is highly soluble even in hexane.

The observed regioselectivity in the cyclodehydrogenation for 9a and 9b can be rationalized with the aid of theoretical calculations. We performed a detailed theoretical study on the activation barriers for different protonation/cyclization sites.

After initial structure optimization using DFT with B3LYP and the 6-31G(d,p) basis set, further optimization was carried out at the M06-2X/6-311+G(d,p) level of theory in a solvent model (IEFPCM) and the stationary points were characterized by frequency calculations in order to verify that they have the right number of imaginary frequencies. Thus, the energies and coordinates of the lowest energy transition state of each ring closure after each position was protonated were obtained (see ESI, Tables S4 and S5†).

**Scheme 3:**
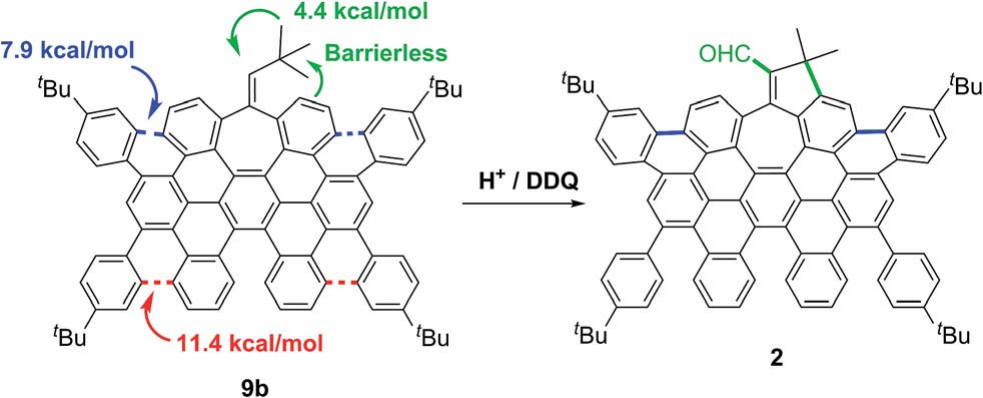
Summary of calculated energies of subsequent transformations of 9b upon protonation.

Hence, in agreement with the experimental results, the theoretical data showed that the carbocationic species, formed upon protonation of the aromatic rings in 9a and 9b, suffer facile cyclization. Particularly cyclizations *en route* to 1 and 2 have lower activation energies (Δ*E*^‡^ = 7.9, Δ*G*^‡^ = 10.1 kcal mol^−1^ for 9a and Δ*E*^‡^ = 7.9, Δ*G*^‡^ = 8.8 kcal mol^−1^ for 9b, Scheme 3, blue). Theoretical calculations also suggest that the presence of the five membered ring in compound 2 derives from the protonation of the exocyclic double bond of 9b, triggering a fast methyl group migration (Δ*G*^‡^ = 4.4 kcal mol^−1^) and a subsequent barrierless electrophilic aromatic substitution (Scheme 3, green. See ESI, Fig. S9†).

Final DDQ-mediated dehydrogenation and oxidation of the allylic moiety give rise to the corresponding conjugated aldehyde.^20^

### Optical and electrochemical properties

**Fig. 4:**
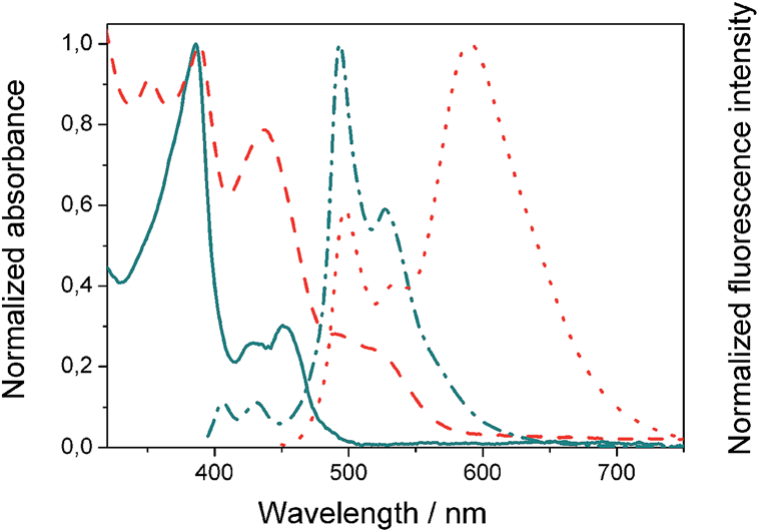
Normalized absorbance of compound 1 (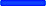) and 2 (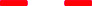) in CH_2_Cl_2_ and corresponding normalized fluorescence emission spectra upon excitation at 385 (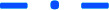) and 440 (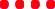) nm, respectively.

**Fig. 5:**
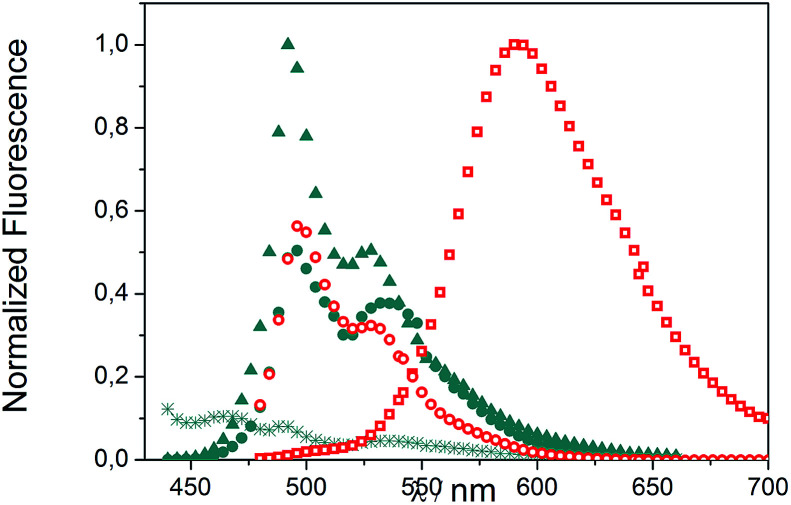
TRES deconvolution of compounds 1: *τ*_1_ = 14.5 ns (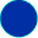), *τ*_2_ = 4.1 ns (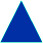) and 2: *τ*_1_ = 3.7 ns (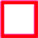), *τ*_2_ = 12.9 ns (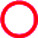) in CH_2_Cl_2_.

We evaluated the optical and electrochemical properties of 1 and 2. Shown in Fig. 4 are the absorption and fluorescence spectra of 1 and 2 in CH_2_Cl_2_. UV-Vis spectra of both compounds showed a very strong absorption band centered at about 385 nm. Optical band gaps of 1 and 2 can be estimated from the longest absorption wavelength as 2.48 and 2.16 eV, respectively, which are in the range of known organic semiconductors.^21^ Notably, compounds 1 and 2 exhibited similar shaped fluorescence patterns when irradiated with UV light, including one intense blue fluorescence in CH_2_Cl_2_ centered at 495 nm followed by a shoulder peak at 530 nm. Their structured emission spectra are characterized by a vibronic progression indicative of a rigid aromatic fluorophore. Compound 2 also showed a broad emission band centered at 590 nm which can be assigned to the conjugated aldehyde chromophore. Both distorted extended nanographenes 1 and 2 showed similar quantum efficiencies (*Φ*) of 7.2 and 7.5%, respectively. Fluorescence lifetimes were also evaluated for both compounds as *τ*_1_ = 14.5 ns and *τ*_2_ = 4.1 ns for 1 and *τ*_1_ = 12.9 ns and *τ*_2_ = 3.7 ns for 2. These exceptionally long fluorescence lifetimes of 14.5 and 12.9 ns are significantly longer than that of related fluorescent dyes, such as perylene bisimides.^22^ Time Resolved Emission Spectroscopy (TRES) allowed the complete deconvolution of the total emission spectrum by recovering the species-associated emission spectra (SAEMS) for each one of the decay times (Fig. 5).

We could confirm in both cases the presence of two main emissive species. In the case of 1, both species were centered at 495 nm and maintained their original rigid aromatic fluorophore, owing to their characteristic vibronic progression. The presence of the two diastereomeric species, owing to the presence of both a helicene-like moiety and a non-planar unit, might be responsible for the observed emission spectra, given that the interconversion between diastereomers is slower than the emission process. The species with the shorter decay time (*τ*_2_ = 4.1 ns) dominated the emission. The longer one (*τ*_1_ = 14.5 ns) could derive from some minor structural changes, possibly due to the presence of the pendant phenyl group in 1 (Fig. 2, blue). For 2, only the component with the longer lifetime (*τ*_2_ = 12.9 ns) kept the rigid fluorophore emitting at 495 nm, while the species with the shorter decay time (*τ*_1_ = 3.7 ns) is responsible for the broad emission centered at 590 nm.

**Fig. 6:**
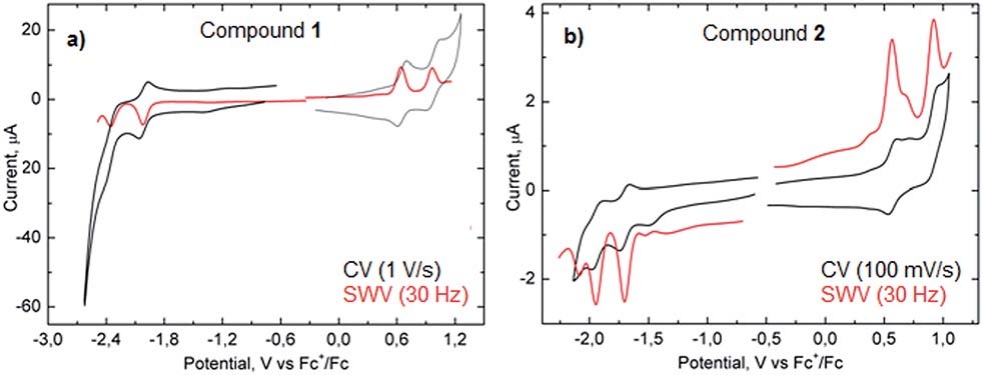
CV and SWV of compound 1 (a) and 2 (b) in CH_2_Cl_2_.

Measurements of the electrochemical behavior of compounds 1 and 2 were possible because they both have sufficient solubility in common organic solvents such as chloroform, dichloromethane and even hexane. In CH_2_Cl_2_, both 1 and 2 display two reversible oxidation waves with first and second oxidation potentials of +0.64 and +0.96 V and +0.58 and +0.91 V (*vs.* Fc^+^/Fc), respectively (Fig. 6). In the negative potential region, two reversible reduction waves were observed with first and second reduction potentials of −2.02 and −2.36 V (*vs.* Fc^+^/Fc) for 1 and −1.69 and −1.95 V (*vs.* Fc^+^/Fc) for 2. Hence, the HOMO–LUMO energy gaps for 1 and 2 based on the first half-wave oxidation and reduction potentials resulted in 2.66 eV and 2.27 eV, respectively, which are in the range of previously described extended aromatic systems.^23^ Therefore, enlarged distorted nanographenes may become attractive candidates for diverse applications such as organic field effect transistors and solar cells.

## Conclusions

We have demonstrated that a wide variety of distorted heptagon-containing nanographene precursors can be easily prepared from simple building blocks by a Co-catalyzed cyclotrimerization reaction. The versatility of this method allows easy introduction of functional groups, affording a selection of distorted nanographenes. Furthermore, these nanostructures can be easily enlarged by the combination of Ni-catalyzed cross-coupling and cyclodehydrogenation reactions. Following this approach we have successfully presented highly distorted derivatives 1 and 2. Notably, distorted PAHs 1 and 2 exhibited interesting optical and electronic properties, with exceptionally long fluorescence lifetimes (14.5 and 12.9 ns, respectively) and promising low band gaps (2.66 and 2.27 eV, respectively). Remarkably, both compounds resemble the elementary structural topological defects in graphene disclinations as heptagons and dislocations as a pair of edge-sharing pentagon–heptagons. The ability to chemically tailor heptagon-containing nanographenes and GNRs in a well-defined manner would help to clarify the properties of defective graphene, and pave the way for the development of distorted graphene molecule-based devices for nanoelectronics and optoelectronics.

## Supplementary Material

SC-008-C6SC02895K-s001

SC-008-C6SC02895K-s002
