# Supplementary material for: Versatile synthesis and enlargement of functionalized distorted heptagon-containing nanographenes
Source: Chem Sci. 2016 Aug 31;8(2):1068–74. doi: 10.1039/c6sc02895k (PMC5357993; doi:10.1039/c6sc02895k)
Supplement: SC-008-C6SC02895K-s001 [file SC-008-C6SC02895K-s001.pdf]

Electronic Supplementary Material (ESI) for Chemical Science.
This journal is © The Royal Society of Chemistry 2016

Electronic Supplementary Information

For:

Versatile Synthesis and Enlargement of Functionalized Distorted Heptagon-Containing Nanographenes

Irene R. Márquez,[a] Noelia Fuentes,[a] Carlos M. Cruz,[a] Virginia Puente-Muñoz,[b] Lia Sotorrios,[c] M. Luisa Marcos,[d] Duane Choquesillo-Lazarte,[e] Blanca Biel,[f] Luis Crovetto,[b] Enrique Gómez-Bengoa,[c] M. Teresa González,[g] Ruben Martin,[h] Juan M. Cuerva,[a] and Araceli G. Campaña*,[a]

[a.] *Departamento Química Orgánica, Universidad de Granada (UGR). C. U. Fuentenueva,18071 Granada, Spain. email: araceligc@ugr.es, jmcuerva@ugr.es*
[b.] *Departamento de Fisicoquímica, Facultad de Farmacia, UGR. Cartuja Campus, 18071 Granada, Spain.*
[c.] *Departamento de Química Orgánica I, Universidad del País Vasco, E-20018, San Sebastián (Spain)*
[d.] *Departamento de Química, Universidad Autónoma de Madrid. c/Francisco Tomás y Valiente nº 7, Cantoblanco, 28049 Madrid, Spain.*
[e.] *Laboratorio de Estudios Cristalográficos, Instituto Andaluz de Ciencias de la Tierra (CSIC-UGR), 18100 Armilla, Granada, Spain.*
[f.] *Departamento de Electrónica y Tecnología de Computadores. Facultad de Ciencias, CITIC, UGR, E-18071 Granada. (Spain)*
[g.] *Fundación IMDEA Nanociencia. Ciudad Universitaria de Cantoblanco, E-28049 Madrid. (Spain)*
[h.] *Institute of Chemical Research of Catalonia (ICIQ). Catalan Institution for Research and Advanced Studies (ICREA)*

Table of Contents

1. General details S2
2. Synthesis and spectroscopy data of new compounds S3
3. Photophysic properties of **1** and **2** S23
4. Electrochemical measurements of **1** and **2** S24
5. Single crystal X-Ray analysis S25
6. Theoretical calculations S29
7. Copies of ^{1}H and ^{13}C-NMR spectra of new compounds S51
8. Copies of VT-NMR, 2D-NMR and HRMS-MALDI spectra of **1** and **2** S87
9. Literature S95

1. General Details

Unless otherwise stated, all reagents and solvents (CH_2Cl_2, EtOAc, hexane, Et_3N, *i*-Pr_2NH, MeOH) were purchased from commercial sources and used without further purification. Dry THF was freshly distilled over Na/benzophenone. Dry CH_2Cl_2, $(CH_2Cl)_2$, toluene and 1,4-dioxane were purchased from Sigma-Aldrich. Flash column chromatography was carried out using Silica gel 60 (230-400 mesh, Scharlab, Spain) as the stationary phase. Analytical TLC was performed on aluminium sheets coated with silica gel with fluorescent indicator UV_{254} (Alugram SIL G/UV_{254}, Mackerey-Nagel, Germany) and observed under UV light (254 nm) and/or staining with Ce/Mo reagent or phosphomolybdic acid solution and subsequent heating. Preparative TLC was performed on Silica gel G preparative layer (20 x 20 cm, 1000 microns). All ^{1}H and ^{13}C NMR spectra were recorded on Varian 300, 400, 500 or 600 MHz spectrometers, at a constant temperature of 298 K. Chemical shifts are reported in ppm and referenced to residual solvent. Coupling constants (*J*) are reported in Hertz (Hz). Standard abbreviations indicating multiplicity were used as follows: m = multiplet, quint. = quintet, q = quartet, t = triplet, d = doublet, s = singlet, b = broad. Assignment of the ^{13}C NMR multiplicities was accomplished by DEPT techniques. MALDI-TOF mass spectra were recorded on a Bruker Ultraflex III mass spectrometer. High resolution ESI-TOF mass spectrometry was carried out on a Waters Synapt G2 mass spectrometer.

2. Synthesis and spectroscopy data of new compounds

- **<u>General procedure I</u>: Sonogashira coupling reaction to obtain compounds type 3**

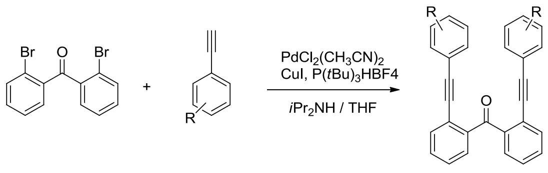

A solution of the corresponding ethynyl benzene (3 equiv.) dissolved in the minimum volume of THF was added to a degassed suspension of the 2,2'-dibromobenzophenone (1.47 mmol, 1 equiv.), $PdCl_2(CH_3CN)_2$ (0.15 equiv.), CuI (0.15 equiv.) and P(*t*Bu)$_3$HBF$_4$ (0.3 equiv.) in *i*-Pr$_2$NH (5 mL). The reaction was stirred under inert atmosphere at room temperature during 2 h, followed by TLC. The mixture was then diluted with EtOAc, washed with aqueous NH_4Cl, dried over anhydrous Na_2SO_4 and the solvent was removed under reduced pressure. The residue was purified by column chromatography (EtOAc/hexane mixtures) to give the corresponding products that were characterized by ^{1}H-RMN, ^{13}C-RMN and HRMS.

Compound 3a

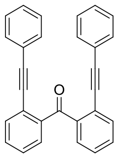

Compound **3a** was prepared from phenylacetylene according to general procedure I. (Eluent mixtures: EtOAc/hexane: 2/8). Yield: 97%, dark solid.

^{1}H-NMR (300 MHz, $CDCl_3$): δ = 7.76 (d, *J* = 7.6, 1.4 Hz, 2H), 7.65 (dd, *J* = 7.4, 1.4 Hz, 2H), 7.54 – 7.41 (m, 4H), 7.28 (bs, 10H). ^{13}C-NMR (75 MHz, $CDCl_3$): δ = 197.0 (C), 141.2 (C), 133.4 (CH), 131.7 (CH), 131.3 (CH), 130.0 (CH), 128.4 (CH), 128.3 (CH), 128.1 (CH), 122.8 (C), 122.7 (C), 95.5 (C), 87.8 (C). HRMS (EI): *m/z* calcd. for $C_{29}H_{18}O$ $[M]^+$: 382.1358; found: 382.1351.

Compound 3b

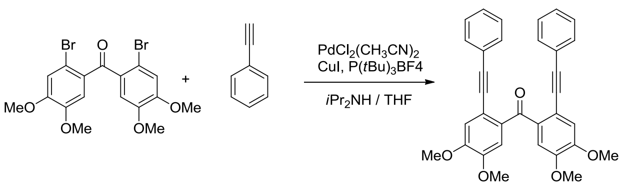

Phenylacetylene (0.22 mL, 2.0 mmol) was added to a degassed suspension of the bis(2-bromo-4,5-dimethoxyphenyl)methanone[S1] (300 mg, 0.667 mmol), $PdCl_2(CH_3CN)_2$ (26 mg, 0.10 mmol), CuI (20 mg, 0.10 mmol) and P(*t*Bu)$_3$BF$_4$ (60 mg, 0.20 mmol) in a mixture of *i*-Pr$_2$NH/THF, 3/1 (4 mL). The reaction was stirred at room temperature during 2 h. The mixture was then diluted with EtOAc, washed with aqueous NH_4Cl, dried over anhydrous Na_2SO_4 and the solvent was removed under reduced pressure. The residue was purified by column chromatography (EtOAc/hexane: 3/7) to give **3b** (238 mg, 71%) as a dark solid.

^{1}H NMR (300 MHz, $CDCl_3$) δ = 7.27 (s, 2H), 7.25 – 7.20 (m, 6H), 7.15 – 7.09 (m, 4H), 6.99 (s, 2H), 3.92 (s, 6H), 3.88 (s, 6H). High quality ^{13}C-NMR was not obtained. HRMS (MALDI, DCTB): *m/z* calcd. for $C_{33}H_{26}NaO_5$ $[M+Na]^+$: 525.1672; found: 525.1666.

Compound 3c

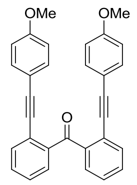

Compound **3c** was prepared from 1-ethynyl-4-methoxybenzene according to general procedure I. (Eluent mixtures: EtOAc/hexane: 3/7). Yield: 83%, dark solid.

^{1}H-NMR (300 MHz, $CDCl_3$): δ = 7.69 (d, *J* = 7.4 Hz, 2H), 7.57 (d, *J* = 7.5 Hz, 2H), 7.45 (t, *J* = 7.2 Hz, 2H), 7.38 (t, *J* = 7.4 Hz, 2H), 7.15 (d, *J* = 8.5 Hz, 4H), 6.76 (d, *J* = 8.6 Hz, 4H), 3.78 (s, 6H). ^{13}C NMR (101 MHz, CD_2Cl_2) δ = 197.4 (C), 160.5 (C), 141.6 (C), 133.7 (CH), 133.6 (CH), 131.7 (CH), 130.4 (CH), 128.5 (CH), 123.5 (C), 115.3 (C), 114.3 (CH), 95.9 (C), 87.1 (C), 55.8 (CH_3). The compound was used to prepare **5c**.

Compound 3d

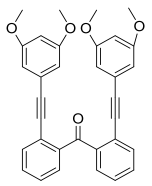

Compound **3d** was prepared from 3,5-dimethoxyphenyl acetylene according to general procedure I. (Eluent mixtures: EtOAc/hexane: 3/7). Yield: 88%, dark solid.

^{1}H-NMR (300 MHz, $CDCl_3$): δ = 7.70 (d, *J* = 7.2 Hz, 2H), 7.60 (d, *J* = 6.8 Hz, 2H), 7.53 – 7.37 (m, 4H), 6.39 (d, *J* = 2.2 Hz, 2H), 6.36 (d, *J* = 2.2 Hz, 4H), 3.75 (s, 12H). ^{13}C-NMR (75 MHz, $CDCl_3$): δ = 196.9 (C), 160.4 (C), 141.2 (C), 133.5 (CH), 131.3 (CH), 130.1 (CH), 128.4 (CH), 124.1 (C), 122.6 (C), 109.4 (CH), 102.2 (CH), 95.5 (C), 87.3 (C), 55.5 (CH_3). HRMS (MALDI, DCTB): *m/z* calcd. for $C_{33}H_{26}NaO_5$ $[M+Na]^+$: 525.1672; found: 525.1673.

Compound 3e

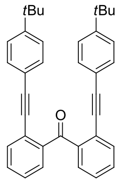

Compound **3e** was prepared from 1-(*tert*-butyl)-4-ethynylbenzene according to general procedure I. (Eluent mixtures: EtOAc/hexane: 5/95). Yield: 93%, dark solid.

^{1}H NMR (300 MHz, CD_2Cl_2) δ = 7.69 (dd, *J* = 7.5, 1.3 Hz, 2H), 7.64 – 7.60 (m, 2H), 7.55 – 7.40 (m, 4H), 7.30 (d, *J* = 8.6 Hz, 4H), 7.14 (d, *J* = 8.6 Hz, 4H), 1.30 (s, 18H). ^{13}C NMR (126 MHz, CD_2Cl_2) δ

= 197.3 (C), 152.5 (C), 141.7 (C), 133.8 (CH), 131.9 (CH), 131.8 (CH), 130.5 (CH), 128.7 (CH), 125.8 (CH), 123.3 (C), 120.2 (C), 96.0 (C), 87.7 (C), 35.3 (C), 31.4 (CH_3). HRMS (EI): *m/z* calcd. for $C_{37}H_{34}O$ $[M]^+$: 494.2610; found: 494.2607.

Compound 3f

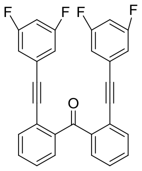

Compound **3f** was prepared from 1-ethynyl-3,5-difluorobenzene according to general procedure I. (Eluent mixtures: EtOAc/hexane: 1/9). Yield: 92%, dark solid.

^{1}H NMR (300 MHz, $CDCl_3$) δ = 7.71 (d, *J* = 6.9 Hz, 2H), 7.61 (d, *J* = 6.9 Hz, 2H), 7.56 – 7.44 (m, 4H), 6.78 – 6.64 (m, 6H). ^{13}C NMR (126 MHz, $CDCl_3$) δ = 196.5 (C), 163.6 (d, *J* = 13.3 Hz, C), 161.6 (d, *J* = 13.3 Hz, C), 141.4 (C), 133.6 (CH), 131.6 (CH), 130.3 (CH), 129.1 (CH), 125.4 (t, *J* = 11.8 Hz, C), 121.9 (C), 114.8 (d, *J* = 6.6 Hz, CH), 114.6 (d, *J* = 6.5 Hz, CH), 104.8 (t, *J* = 25.4 Hz, CH), 93.1 (C), 89.4 (C). HRMS (MALDI, DCTB): *m/z* calcd. for $C_{29}H_{14}F_2NaO$ $[M+Na]^+$: 477.0873; found: 477.0867

- **Synthesis and characterization data of diphenylacetylene compounds type 4**

The following substituted diphenylacetylenes were synthesized according to literature procedure: 1,2-bis(3,5-dimethoxyphenyl)ethyne **4b**,[S2] 1,2-bis(4-methoxyphenyl)ethyne **4c**,[S2] 1,2-bis(3,5-dimethylphenyl)ethyne **4e**,[S2] 1-bromo-4-(phenylethynyl)benzene **4f**,[S2] 1,2-di(thiophen-2-yl)ethyne **4h**.[S2] Their spectroscopic data were identical to the reported compounds: **4b**,[S3] **4c**,[S2] **4e**,[S4] **4f**,[S5] **4h**.[S2]

Compound 4d

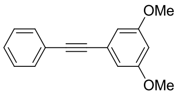

Phenylacetylene (0.62 mL, 5.68 mmol) was added to a degassed suspension of 5-iodo-1,3-dimethoxybenzene (1 g, 3.79 mmol), $PdCl_2(PPh_3)_2$ (27 mg, 0.038 mmol) and CuI (7 mg, 0.038 mmol) in a mixture 3/1 of Et_3N/THF (12 mL). The reaction was stirred for 2 h under argon atmosphere at room temperature. The mixture was then diluted with EtOAc, washed with aqueous NH_4Cl and brine, dried over anhydrous Na_2SO_4 and the solvent was removed under reduced pressure. The residue was purified by column chromatography (EtOAc/hexane: 2/8) to give **4d** (870 mg, 97%) as a vitreous solid that showed NMR spectra identical to reported data.[S6]

Compound 4g

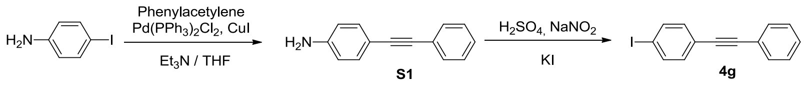

Phenylacetylene (0.75 mL, 6.85 mmol) was added to a degassed suspension of 4-iodoaniline (1000 mg, 4.56 mmol), $PdCl_2(PPh_3)_2$ (32 mg, 0.046 mmol) and CuI (9 mg, 0.046 mmol) in a mixture 3/1 of Et_3N/THF (12 mL). The reaction was stirred for 2 h under argon atmosphere at room temperature. The mixture was then diluted with EtOAc, washed with aqueous NH_4Cl, dried over anhydrous Na_2SO_4 and the solvent was removed under reduced pressure. The residue was purified by column chromatography (EtOAc/hexane: 2/8) to give **S1**.[S7] The amine **S1** (947 mg, 4.901 mmol) was suspended in distilled water (8 mL), a solution of sulfuric acid 98% (0.8 mL) was added and the mixture was cooled to 0 °C. Sodium nitrite (405 mg, 5.88 mmol) in water (1.5 mL) was added dropwise with stirring, keeping the temperature 0–5 °C, during 30 min. THF (4 mL) was added to the reaction mixture and then, a solution of potassium iodide (2.44 g, 14.70 mmol) in water (2 mL) was added slowly during 30 min. After 3 h the reaction mixture was diluted with EtOAc and washed with saturated Na_2SO_3 solution and brine, dried over anhydrous Na_2SO_4 and concentrated under reduced pressure. The residue was purified by column chromatography (EtOAc/hexane: 1/9) to give **4g** (1 g, 67%) as a white solid that showed NMR spectra identical to reported data.[S8]

Compound 4i

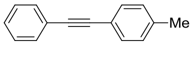

p-Tolylacetylene (0.56 mL, 4.41 mmol) was added to a degassed suspension of iodobenzene (600 mg, 2.941 mmol), $PdCl_2(PPh_3)_2$ (20 mg, 0.029 mmol) and CuI (11 mg, 0.060 mmol) in a mixture 3:1 of Et_3N/THF (4 mL). The reaction was stirred for 2 h under argon atmosphere at room temperature. The mixture was then diluted with EtOAc, washed with aqueous NH_4Cl, dried over anhydrous Na_2SO_4 and the solvent was removed under reduced pressure. The residue was purified by column chromatography (EtOAc/hexane: 2/98) to give **4i** (555 mg, 98%) as a white solid that showed NMR spectra identical to reported data.[S9]

- **<u>General procedure II:</u> cyclotrimerization reaction to obtain compounds type 5**

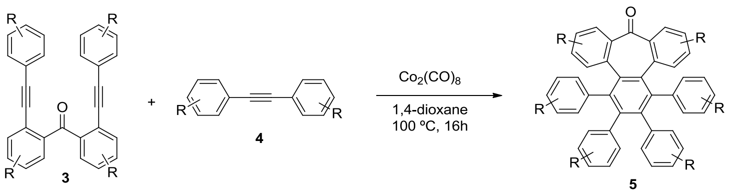

A degassed solution of the corresponding dialkyne **3** (0.19 mmol, 1 equiv.) in 1,4-dioxane (2 mL) was added to a degassed solution of $Co_2(CO)_8$ (1.3 equiv.) in 1,4-dioxane (6 mL) and the mixture was stirred at 100°C during 30 min. Then a degassed solution of the diphenylacetylene **4** (1.5 equiv.) in 1,4-dioxane (2 mL) was added dropwise during 30 min. The reaction was stirred 16 h under argon atmosphere at 100°C. The mixture was then cooled to room temperature, and the solvent was removed under reduced pressure. The residue was

adsorbed on silica gel and purified by column chromatography (CH_2Cl_2/hexane mixtures) to give the corresponding products that were characterized by ^{1}H-RMN, ^{13}C-RMN and HRMS.

Compound 5a

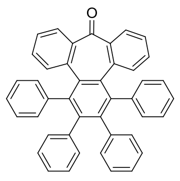

Compound **5a** was prepared from compound **3a** and diphenylacetylene according to general procedure II. (Eluent mixtures: CH_2Cl_2/hexane: 3/7). Yield: 54 %, white solid.

^{1}H-NMR (600 MHz, $CDCl_3$): δ = 7.41 (d, *J* = 7.6 Hz, 2H), 7.36 (d, *J* = 7.8 Hz, 2H), 7.16 (d, *J* = 7.7 Hz, 2H), 7.10 (t, *J* = 7.6 Hz, 2H), 7.03 (t, *J* = 7.8 Hz, 2H), 7.01 (d, *J* = 7.6 Hz, 2H), 6.95 (t, *J* = 7.7 Hz, 2H), 6.86 (t, *J* = 7.7 Hz, 2H), 6.83 (t, *J* = 7.6 Hz, 2H), 6.81 – 6.77 (m, 4H), 6.67 (t, *J* = 7.8 Hz, 2H), 6.50 (d, *J* = 7.7 Hz, 2H), 6.40 (d, *J* = 7.8 Hz, 2H). ^{13}C-NMR (151 MHz, $CDCl_3$): δ = 200.5 (C), 146.2 (C), 142.9 (C), 141.5 (C), 140.8 (C), 140.3 (C), 135.5 (C), 134.9 (C), 133.3 (CH), 132.7 (CH), 131.7 (CH), 130.7 (CH), 130.4 (CH), 128.7 (CH), 127.3 (CH), 127.2 (CH), 127.1 (CH), 126.6 (CH), 126.1 (CH), 125.7 (CH), 125.6 (CH), 124.4 (CH). HRMS (MALDI, DCTB): *m/z* calcd. for $C_{43}H_{28}O$ $[M]^+$: 560.2135; found: 560.2115.

Compound 5b

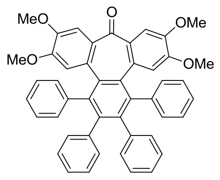

Compound **5b** was prepared from **3b** and diphenylacetylene according to general procedure II. (Eluent mixtures: CH_2Cl_2/hexane: 4/6). Yield: 51%, white solid.

^{1}H NMR (400 MHz, $CDCl_3$) δ = 7.36 (d, *J* = 7.7 Hz, 2H), 7.16 (d, *J* = 7.7 Hz, 2H), 7.06 – 6.98 (m, 4H), 6.98 – 6.92 (m, 2H), 6.91 – 6.82 (m, 4H), 6.79 (t, *J* = 7.5 Hz, 2H), 6.66 (t, *J* = 7.6 Hz, 2H), 6.55 (d, *J* = 6.8 Hz, 2H), 6.51 (s, 2H), 6.38 (d, *J* = 7.6 Hz, 2H), 3.88 (s, 6H), 3.31 (s, 6H). ^{13}C NMR (101 MHz, $CDCl_3$) δ = 198.2 (C), 148.9 (C), 147.9 (C), 142.7 (C), 141.3 (C), 141.1 (C), 140.4 (C), 139.3 (C), 135.2 (C), 132.4 (CH), 131.8 (CH), 130.9 (CH), 130.5 (CH), 128.6 (C), 127.9 (CH), 127.1 (CH), 126.7 (CH), 126.0 (CH), 125.8 (CH), 125.6 (CH), 116.8 (CH), 107.8 (CH), 56.1 (CH_3), 55.6 (CH_3). HRMS (MALDI, DCTB): *m/z* calcd. for $C_{47}H_{36}O_5$ $[M]^+$: 680.2557; found: 680.2531.

Compound 5c

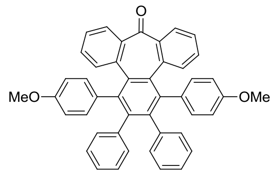

Compound **5c** was prepared from **3c** and diphenylacetylene according to general procedure II. (Eluent mixtures: CH_2Cl_2/hexane: 4/6). Yield: 42%, white solid.

^{1}H NMR (400 MHz, $CDCl_3$) δ = 7.40 (d, *J* = 7.6, 2H), 7.33 (d, *J* = 7.7, 2H), 7.09 (t, *J* = 7.6, 2H), 7.05 – 6.97 (m, 6H), 6.86 (t, *J* = 7.7, 2H), 6.80 (t, *J* = 7.5, 2H), 6.69 (t, *J* = 7.6, 2H), 6.49 (d, *J* = 7.6, 2H), 6.39 (d, *J* = 8.5, 4H), 6.33 (d, *J* = 7.3, 2H), 3.61 (s, 6H). ^{13}C NMR (101 MHz, $CDCl_3$) δ = 200.7 (C), 157.4 (C), 146.2 (C), 143.2 (C), 141.0 (C), 140.5 (C), 135.8 (C), 135.2 (C), 133.6 (CH), 133.3 (CH), 133.2 (C), 131.8 (CH), 131.7 (CH), 130.4 (CH), 128.7 (CH), 127.2 (CH), 127.1 (CH), 126.1 (CH), 125.6 (CH), 124.3 (CH), 112.6 (CH), 112.4 (CH), 55.1 (CH_3). MS (MALDI, DCTB): *m/z* calcd. for $C_{45}H_{32}O_3$ $[M]^+$: 620.2; found: 620.3. (HRMS was not obtained).

Compound 5d

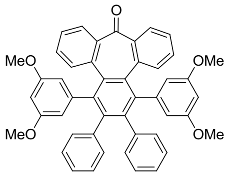

Compound **5d** was prepared from **3d** and diphenylacetylene according to general procedure II. (Eluent mixtures: CH_2Cl_2/hexane: 7/3). Yield: 61%, white solid.

^{1}H-NMR (500 MHz, $CDCl_3$): δ = 7.42 (d, *J* = 7.6, 1.1 Hz, 2H), 7.38 (d, *J* = 7.7 Hz, 2H), 7.15 (dt, *J* = 7.5, 0.9 Hz, 2H), 7.10 (d, *J* = 7.4 Hz, 2H), 7.04 (t, *J* = 7.6 Hz, 2H), 6.93 (dt, *J* = 7.7, 1.3 Hz, 2H), 6.83 (t, *J* = 7.5 Hz, 2H), 6.73 (t, *J* = 7.6 Hz, 2H), 6.45 (d, *J* = 7.7 Hz, 2H), 6.33 (dd, *J* = 2.1, 1.2 Hz, 2H), 6.00 (t, *J* = 2.3 Hz, 2H), 5.63 (dd, *J* = 2.0, 1.2 Hz, 2H), 3.54 (s, 6H), 3.37 (s, 6H). ^{13}C-NMR (126 MHz, $CDCl_3$): δ = 200.3 (C), 159.8 (C), 159.4 (C), 146.1 (C), 142.7 (C), 142.3 (C), 141.3 (C), 140.2 (C), 135.5 (C), 135.1 (C), 132.9 (CH), 131.8 (CH), 130.2 (CH), 129.1 (CH), 127.4 (CH), 127.3 (CH), 126.0 (CH), 125.8 (CH), 124.4 (CH), 111.8 (CH), 108.4 (CH), 99.4 (CH), 55.4 (CH_3), 55.3 (CH_3). HRMS (MALDI, DCTB): *m/z* calcd. for $C_{47}H_{36}O_5$ $[M]^+$: 680.2557; found: 680.2543.

Compound 5e

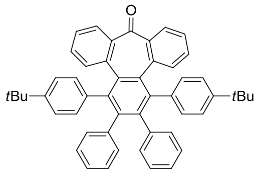

Compound **5e** was prepared from **3e** and diphenylacetylene according to general procedure II. (Eluent mixtures: CH_2Cl_2/hexane: 4/6). Yield: 44%, white solid.

^{1}H NMR (500 MHz, $CDCl_3$) δ = 7.39 (d, *J* = 7.4 Hz, 2H), 7.35 (d, *J* = 7.7 Hz, 2H), 7.06 (t, *J* = 7.5 Hz, 2H), 7.02 (t, *J* = 7.8 Hz, 4H), 6.94 (t, *J* = 8.7 Hz, 4H), 6.80 – 6.74 (m, 6H), 6.66 (t, *J* = 7.6 Hz, 2H), 6.39 (t, *J* = 8.8 Hz, 4H), 1.13 (s, 18H). ^{13}C NMR (126 MHz, $CDCl_3$) δ = 200.6 (C), 148.4 (C), 146.2 (C), 142.8 (C), 141.4 (C), 140.5 (C), 137.7 (C), 135.7 (C), 135.2 (C), 133.2 (CH), 132.3 (CH), 131.8 (CH), 130.6 (CH), 130.3 (CH), 128.4 (CH), 127.0 (CH), 126.9 (CH), 126.0 (CH), 125.5 (CH), 124.3 (CH), 124.1 (CH), 123.4 (CH), 34.3 (C), 31.3 (CH_3). HRMS (MALDI, DCTB): *m/z* calcd. for $C_{51}H_{44}O$ $[M]^+$: 672.3387; found: 672.3366.

Compound 5f

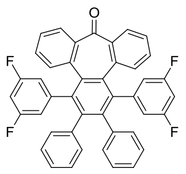

Compound **5f** was prepared from **3f** and diphenylacetylene according to general procedure II. (Eluent mixtures: CH_2Cl_2/hexane: 2/8). Yield: 34%, white solid.

^{1}H-NMR (400 MHz, CD_2Cl_2): δ = 7.44 (d, *J* = 7.6 Hz, 2H), 7.37 (d, *J* = 7.7 Hz, 2H), 7.22 (t, *J* = 7.6 Hz, 2H), 7.11 (t, *J* = 7.7 Hz, 2H), 7.05 (d, *J* = 7.6 Hz, 2H), 6.99 (t, *J* = 7.6 Hz, 2H), 6.90 (t, *J* = 7.7 Hz, 2H), 6.78 (t, *J* = 7.7 Hz, 2H), 6.74 (d, *J* = 9.2 Hz, 2H), 6.47 (d, *J* = 7.7 Hz, 2H), 6.40 (tt, *J* = 9.2, 2.3 Hz, 2H), 6.07 (d, *J* = 9.2 Hz, 2H). ^{13}C-NMR (101 MHz, CD_2Cl_2): δ = 199.4 (C), 163.7 (dd, *J* = 28.3, 12.9 Hz, C), 161.2 (dd, *J* = 27.5, 12.9 Hz, C), 146.7 (C), 144.7 (t, *J* = 10.0 Hz, C), 143.1 (C), 140.4 (t, *J* = 2.1 Hz, C), 139.9 (C), 136.0 (C), 134.4 (C), 133.0 (CH), 132.1 (CH), 130.5 (CH), 129.5 (CH), 128.4 (CH), 127.8 (CH), 127.0 (CH), 126.7 (CH), 125.2 (CH), 116.3 (dd, *J* = 22.0, 3.5 Hz, CH), 113.9 (dd, *J* = 21.9, 3.3 Hz, CH), 101.8 (t, *J* = 25.4 Hz, CH). HRMS (MALDI. DCTB): *m/z* calcd. for $C_{43}H_{24}F_4NaO$ $[M+Na]^+$: 655.1655; found: 655.1643.

Compound 5g

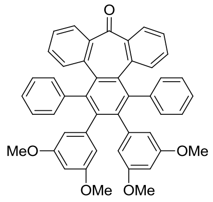

Compound **5g** was prepared from **3a** and **4b** according to general procedure II. (Eluent mixtures: CH_2Cl_2/hexane: 8/2). Yield: 51%, white solid.

^{1}H-NMR (500 MHz, CD_2Cl_2): δ = 7.37 (d, *J* = 7.6 Hz, 2H), 7.29 (d, *J* = 7.7 Hz, 2H), 7.12 (t, *J* = 7.5 Hz, 2H), 7.06 (t, *J* = 7.1 Hz, 2H), 7.01 (d, *J* = 7.7 Hz, 2H), 6.95 (t, *J* = 7.4 Hz, 2H), 6.85 (t, *J* = 7.0 Hz, 4H), 6.64 (s, 2H), 6.53 (d, *J* = 7.5 Hz, 2H), 5.98 (t, *J* = 2.2 Hz, 2H), 5.65 (s, 2H), 3.71 (s, 6H), 3.32 (s, 6H). ^{13}C-NMR (126 MHz, CD_2Cl_2): δ = 200.4 (C), 159.8 (C), 159.4 (C), 146.7 (C), 142.6 (C), 142.5 (C), 141.7 (C), 141.4 (C), 136.1 (C), 135.2 (C), 133.6 (CH), 133.1 (CH), 131.0 (CH), 129.0 (CH), 127.7 (CH), 127.6 (CH), 127.2 (CH), 126.2 (CH), 124.6 (CH), 111.8 (CH), 109.1 (CH), 98.9 (CH), 55.8 (CH_3), 55.6 (CH_3). HRMS (MALDI, DCTB): *m/z* calcd. for $C_{47}H_{36}NaO_5$ $[M+Na]^+$: 703.2455; found: 703.2458.

Compound 5h

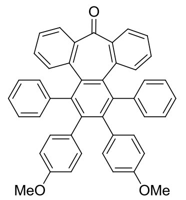

Compound **5h** was prepared from **3a** and **4c** according to general procedure II. (Eluent mixtures: CH_2Cl_2/hexane: 1/1). Yield: 44%, white solid.

^{1}H-NMR (500 MHz, CD_2Cl_2): δ = 7.34 (dd, *J* = 7.7, 1.1 Hz, 2H), 7.25 (dd, *J* = 8.4, 2.1 Hz, 2H), 7.16 (d, *J* = 7.7 Hz, 2H), 7.10 (dt, *J* = 7.7, 1.1 Hz, 2H), 7.00 (t, *J* = 8.4 Hz, 2H), 6.97 (d, *J* = 8.4 Hz, 2H), 6.90 (tt, *J* = 7.7, 1.1 Hz, 2H), 6.83 – 6.77 (m, 4H), 6.60 (dd, *J* = 8.5, 2.7 Hz, 2H), 6.48 (d, *J* = 7.7 Hz, 2H), 6.32 (dd, *J* = 8.5, 2.2 Hz, 2H), 6.23 (dd, *J* = 8.5, 2.2 Hz, 2H), 3.58 (s, 6H). ^{13}C-NMR (126 MHz, CD_2Cl_2): δ = 200.4 (C), 157.7 (C), 146.7 (C), 143.3 (C), 142.3 (C), 141.7 (C), 135.8 (C), 135.5 (C), 133.6 (CH), 133.5 (C), 133.4 (CH), 133.3 (CH), 131.9 (CH), 131.1 (CH), 129.0 (CH), 127.7 (CH), 127.5 (CH), 127.1 (CH), 125.9 (CH), 124.6 (CH), 112.6 (CH), 112.2 (CH), 55.3 (CH_3). HRMS (MALDI, DCTB): *m/z* calcd. for $C_{45}H_{32}O_3$ $[M]^+$: 620.2346; found: 620.2337.

Compound 5i

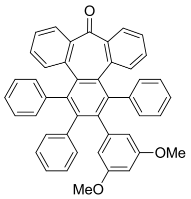

Compound **5i** was prepared from **3a** and **4d** according to general procedure II. (Eluent mixtures: CH_2Cl_2/hexane: 3/7). Yield: 44%, white solid.

^{1}H NMR (500 MHz, $CDCl_3$) δ = 7.45 – 7.38 (m, 3H), 7.22 (d, *J* = 7.7 Hz, 1H), 7.18 (d, *J* = 7.8 Hz, 1H), 7.13 – 7.04 (m, 3H), 7.03 – 6.98 (m, 3H), 6.96 (t, *J* = 7.6 Hz, 1H), 6.90 (t, *J* = 7.5 Hz, 1H), 6.88 – 6.76 (m, 6H), 6.70 (t, *J* = 7.5 Hz, 1H), 6.56 (s, 1H), 6.52 (d, *J* = 7.7 Hz, 1H), 6.48 (d, *J* = 7.7 Hz, 1H), 6.43 (d, *J* = 7.7 Hz, 1H), 5.93 (s, 1H), 5.59 (s, 1H), 3.68 (s, 3H), 3.29 (s, 3H). ^{13}C NMR (126 MHz, $CDCl_3$) δ = 200.5 (C), 159.4 (C), 158.8 (C), 146.2 (C), 146.1 (C), 142.6 (C), 142.5 (C), 141.9 (C), 141.6 (C), 141.3 (C), 140.7 (C), 140.1 (C), 135.6 (C), 135.5 (C), 134.9 (C), 134.8 (C), 133.3 (CH), 133.2 (CH), 132.7 (CH), 132.4 (CH), 131.8 (CH), 130.7 (CH), 130.6 (CH), 130.3 (CH), 128.7 (CH), 127.3 (CH), 127.2 (CH), 127.1 (CH), 126.8 (CH), 126.6 (CH), 126.0 (CH), 125.8 (CH), 125.7 (CH), 125.6 (CH), 124.33 (CH), 124.32 (CH), 111.5 (CH), 108.5 (CH), 98.7 (CH), 55.4 (CH_3), 55.2 (CH_3), (four carbon signals were not observed). HRMS (MALDI, DCTB): *m/z* calcd. for $C_{45}H_{32}O_3$ $[M]^+$: 620.2346; found: 620.2337.

Compound 5j

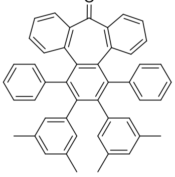

Compound **5j** was prepared from **3a** and **4e** according to general procedure II. (Eluent mixtures: CH_2Cl_2/hexane: 3/7). Yield: 39%, white solid.

^{1}H-NMR (500 MHz, CD_2Cl_2): δ = 7.34 (dd, *J* = 7.6, 1.1 Hz, 2H), 7.17 (d, *J* = 7.7 Hz, 2H), 7.10 (dt, *J* = 7.6, 1.0 Hz, 2H), 7.00 (s, 2H), 6.97 (d, *J* = 7.6 Hz, 4H), 6.89 (t, *J* = 7.4 Hz, 2H), 6.85 – 6.78 (m, 4H), 6.48 (d, *J* = 7.7 Hz, 2H), 6.45 (s, 2H), 6.02 (s, 2H), 2.18 (s, 6H), 1.79 (s, 6H). ^{13}C-NMR (126 MHz,

CD_2Cl_2): δ = 200.3 (C), 146.7 (C), 143.4 (C), 141.8 (C), 141.7 (C), 140.6 (C), 136.5 (C), 135.7 (C), 135.6 (C), 135.5 (C), 133.7 (CH), 133.2 (CH), 131.2 (CH), 130.5 (CH), 129.0 (CH), 127.5 (CH), 127.2 (CH), 126.9 (CH), 125.9 (CH), 124.6 (CH), 21.4 (CH_3), 21.0 (CH_3), (two carbon signals were not observed). HRMS (MALDI, DCTB): *m/z* calcd. for $C_{47}H_{36}O$ $[M]^+$: 616.2761; found: 616.2745.

Compound 5k

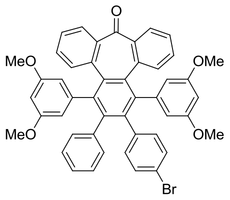

Compound **5k** was prepared from **3d** and **4f** according to general procedure II. (Eluent mixtures: CH_2Cl_2/hexane: 4/6). Yield: 31%, white solid.

^{1}H-NMR (500 MHz, CD_2Cl_2): δ = 7.40 (d, *J* = 7.2 Hz, 3H), 7.29 (d, *J* = 7.8 Hz, 1H), 7.23 – 7.15 (m, 3H), 7.11 (d, *J* = 7.9 Hz, 3H), 6.98 – 6.91 (m, 3H), 6.89 (d, *J* = 8.0 Hz, 1H), 6.79 (t, *J* = 7.4 Hz, 1H), 6.49 (d, *J* = 7.4 Hz, 1H), 6.40 (d, *J* = 7.8 Hz, 1H), 6.33 (s, 1H), 6.32 (s, 1H), 6.04 (s, 1H), 6.00 (s, 1H), 5.63 (s, 2H), 3.59 (s, 3H), 3.56 (s, 3H), 3.40 (s, 3H), 3.38 (s, 3H). ^{13}C-NMR (126 MHz, CD_2Cl_2): δ = 200.0 (C), 160.5 (C), 160.3 (C), 160.1 (C), 160.0 (C), 146.7 (C), 142.8 (C), 142.7 (C), 141.9 (C), 141.6 (C), 141.5 (C), 140.7 (C), 140.1 (C), 136.3 (C), 136.0 (C), 135.34 (C), 135.28 (C), 134.0 (CH), 133.1 (CH), 133.0 (CH), 132.5 (CH), 132.3 (CH), 131.6 (C), 130.7 (CH), 130.6 (CH), 129.7 (CH), 129.4 (CH), 128.6 (C), 127.84 (CH), 127.75 (CH), 126.8 (CH), 126.5 (CH), 124.7 (CH), 120.2 (C), 112.1 (CH), 112.0 (CH), 109.1 (CH), 109.0 (CH), 99.40 (CH), 99.36 (CH), 55.83 (CH_3), 55.78 (CH_3), 55.68 (CH_3), 55.66 (CH_3), (three carbon signals were not observed). HRMS (MALDI, DCTB): *m/z* calcd. for $C_{47}H_{35}BrNaO_5$ $[M+Na]^+$: 781.1560; found: 781.1544.

Compound 5l

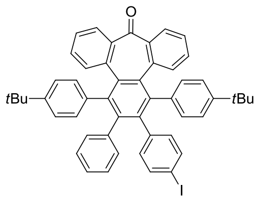

Compound **5l** was prepared from **3e** and **4g** according to general procedure II. (Eluent mixtures: CH_2Cl_2/hexane: 2/8). Yield: 45%, white solid.

^{1}H NMR (500 MHz, $CDCl_3$) δ = 7.39 (d, *J* = 7.7 Hz, 2H), 7.37 – 7.29 (m, 2H), 7.10 – 7.02 (m, 4H), 7.02 – 6.96 (m, 4H), 6.96 – 6.92 (m, 3H), 6.83 (t, *J* = 7.5 Hz, 1H), 6.79 – 6.74 (m, 4H), 6.69 (t, *J* = 7.4 Hz, 1H), 6.39 – 6.33 (m, 3H), 6.14 (dd, *J* = 8.2, 2.2 Hz, 1H), 1.16 (s, 9H), 1.13 (s, 9H). ^{13}C NMR (126 MHz, $CDCl_3$) δ = 200.5 (C), 148.8 (C), 148.5 (C), 146.1 (C), 142.6 (C), 141.6 (C), 141.5 (C), 141.4 (C), 140.2 (C), 140.1 (C), 137.5 (C), 137.4 (C), 136.1 (CH), 136.0 (C), 135.8 (C), 135.2 (CH), 135.1 (C), 135.0 (C), 133.6 (CH), 133.2 (CH), 133.1 (CH), 132.6 (CH), 132.3 (CH), 131.6 (CH), 130.4 (CH), 130.3 (CH), 130.2 (CH), 128.5 (CH), 128.4 (CH), 127.3 (CH), 127.02 (CH), 127.00 (CH), 126.3 (CH), 125.8 (CH), 124.4 (CH), 124.32 (CH), 124.30 (CH), 124.2 (CH), 124.1 (CH), 123.7 (CH), 123.4 (CH), 91.2 (C), 34.4 (C), 34.3 (C), 31.4 (CH_3), 31.3 (CH_3), (one carbon signal

was not observed). HRMS (MALDI, DCTB): *m/z* calcd. for $C_{51}H_{43}INaO$ $[M+Na]^+$: 821.2251; found: 821.2241.

Compound 5m

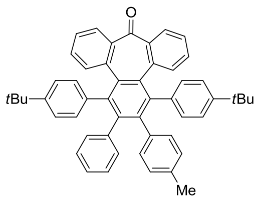

Compound **5m** was prepared from **3e** and **4i** according to general procedure II. (Eluent mixtures: CH_2Cl_2/hexane: 1/1). Yield: 32%, white solid.

^1H NMR (500 MHz, CD_2Cl_2) δ = 7.36 (dd, *J* = 20.9, 7.7 Hz, 2H), 7.34 (d, *J* = 7.6 Hz, 1H), 7.27 (d, *J* = 7.9 Hz, 1H), 7.11 – 6.96 (m, 7H), 6.95 – 6.74 (m, 8H), 6.70 (t, *J* = 7.6 Hz, 1H), 6.49 (dd, *J* = 26.2, 7.8 Hz, 2H), 6.41 – 6.31 (m, 3H), 2.07 (s, 3H), 1.16 (s, 9H), 1.15 (s, 9H). ^{13}C NMR (126 MHz, CD_2Cl_2) δ = 200.4 (C), 149.02 (C), 149.00 (C), 146.7 (C), 146.6 (C), 143.2 (C), 143.0 (C), 142.0 (C), 141.8 (C), 141.3 (C), 138.5 (C), 138.4 (C), 138.1 (C), 136.2 (C), 136.1 (C), 135.7 (C), 135.6 (C), 135.4 (C), 133.6 (CH), 133.5 (CH), 133.0 (CH), 132.9 (CH), 132.4 (CH), 132.2 (CH), 131.0 (CH), 130.8 (CH), 130.7 (CH), 128.8 (CH), 128.0 (CH), 127.32 (CH), 127.30 (CH), 127.27 (CH), 127.25 (CH), 126.6 (CH), 125.8 (CH), 124.52 (CH), 124.50 (CH), 124.47 (CH), 123.9 (CH), 123.8 (CH), 34.64 (C), 34.62 (C), 31.51 (CH_3), 31.50 (CH_3), 21.2 (CH_3), (three carbon signals were not observed). HRMS (MALDI, DCTB): *m/z* calcd. for $C_{52}H_{46}O_1$ $[M]^+$: 686.3543; found: 686.3529.

Compound 5n

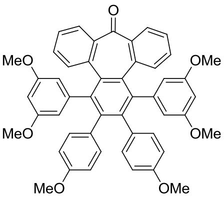

Compound **5n** was prepared from **3d** and **4c** according to general procedure II. (Eluent mixtures: CH_2Cl_2/hexane: 1/1). Yield: 57%, white solid.

^1H-NMR (500 MHz, CD_2Cl_2): δ = 7.39 (d, *J* = 6.8 Hz, 2H), 7.29 (dd, *J* = 8.4, 2.1 Hz, 2H), 7.16 (t, *J* = 7.6 Hz, 2H), 7.09 (d, *J* = 7.6 Hz, 2H), 6.93 (t, *J* = 7.6 Hz, 2H), 6.64 (dd, *J* = 8.4, 2.1 Hz, 2H), 6.40 (dd, *J* = 8.5, 2.1 Hz, 2H), 6.33 (s, 2H), 6.32 (dd, *J* = 8.5, 2.1 Hz, 2H), 6.01 (t, *J* = 2.2 Hz, 2H), 5.63 (s, 2H), 3.61 (s, 6H), 3.59 (s, 6H), 3.39 (s, 6H). ^{13}C-NMR (126 MHz, CD_2Cl_2): δ = 200.2 (C), 160.3 (C), 160.0 (C), 157.9 (C), 146.7 (C), 143.3 (C), 143.0 (C), 142.1 (C), 135.7 (C), 135.6 (C), 133.4 (C), 133.3 (CH), 133.1 (CH), 131.7 (CH), 129.3 (CH), 127.7 (CH), 124.6 (CH), 112.8 (CH), 112.2 (CH), 112.1 (CH), 109.1 (CH), 99.3 (CH), 55.8 (CH_3), 55.7 (CH_3), 55.4 (CH_3). HRMS (MALDI, DCTB): *m/z* calcd. for $C_{49}H_{40}O_7$ $[M]^+$: 740.2769; found: 740.2796.

Compound 5o

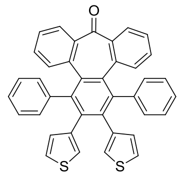

Compound **5o** was prepared from **3a** and **4h** according to general procedure II. (Eluent mixtures: CH_2Cl_2/hexane: 3/7). Yield: 49%, white solid.

^{1}H-NMR (500 MHz, CD_2Cl_2): δ = 7.35 (dd, *J* = 7.6, 1.1 Hz, 2H), 7.23 (d, *J* = 7.6 Hz, 2H), 7.11 (dt, *J* = 7.5, 1.0 Hz, 2H), 7.06 (t, *J* = 8.0 Hz, 2H), 6.99 – 6.93 (m, 4H), 6.87 – 6.80 (m, 6H), 6.68 (bs, 2H), 6.59 (bs, 2H), 6.49 (d, *J* = 7.7 Hz, 2H). ^{13}C-NMR (126 MHz, CD_2Cl_2): δ = 200.2 (C), 146.7 (C), 142.6 (C), 141.4 (C), 140.7 (C), 139.0 (C), 136.1 (C), 135.1 (C), 133.6 (CH), 132.8 (CH), 130.9 (CH), 130.8 (CH), 129.0 (CH), 127.8 (CH), 127.7 (CH), 127.2 (CH), 126.2 (CH), 125.0 (CH), 124.6 (CH), 123.4 (CH). HRMS (MALDI, DCTB): *m/z* calcd. for $C_{39}H_{24}NaOS_2$ $[M+Na]^+$: 595.1161; found: 595.1154.

- **General procedure IIIa: cyclodehydrogenation reaction to obtain compounds type 6**

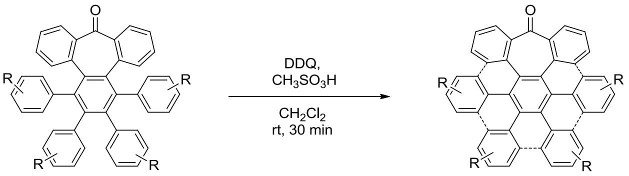

To a solution of the corresponding polyphenylene **5** (0.088 mmol, 1 equiv.) and 2,3-dichloro-5,6-dicyano-1,4-benzoquinone (DDQ) (5 equiv.) in dry CH_2Cl_2 (4 mL), methanesulphonic acid (0.15 mL) was slowly added. The reaction was stirred for 30 min under argon atmosphere at room temperature. The mixture was then diluted with CH_2Cl_2, washed with water, dried over anhydrous Na_2SO_4 and the solvent was removed under reduced pressure. The residue was purified by column chromatography (CH_2Cl_2/hexane mixtures) to give the corresponding products that were characterized by ^{1}H-RMN, ^{13}C-RMN and HRMS.

- **General procedure IIIb: cyclodehydrogenation reaction to obtain compounds type 6**

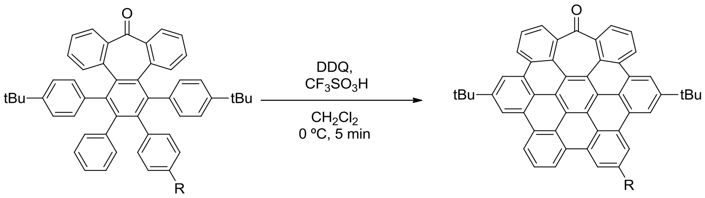

To a solution of the corresponding polyphenylene **5** (0.088 mmol, 1 equiv.) and 2,3-dichloro-5,6-dicyano-1,4-benzoquinone (DDQ) (6 equiv.) in dry CH_2Cl_2 (4 mL), trifluoromethanesulfonic acid (0.1 mL) was slowly added at 0°C. The reaction was stirred for 5 min under argon atmosphere at room temperature. The mixture was then diluted with CH_2Cl_2, washed with

water, dried over anhydrous Na_2SO_4 and the solvent was removed under reduced pressure. The residue was purified by column chromatography (CH_2Cl_2/hexane mixtures) to give the corresponding products that were characterized by ^{1}H-RMN, ^{13}C-RMN and HRMS.

Compound 6a

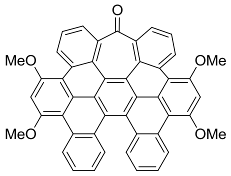

Compound **6a** was prepared from **5d** according to general procedure IIIa. (Eluent mixtures: CH_2Cl_2/hexane: 1/1). Yield: 48%, red solid.

^{1}H-NMR (500 MHz, CD_2Cl_2): δ = 9.61 (dd, *J* = 8.3, 1.5 Hz, 2H), 9.23 (d, *J* = 8.4 Hz, 2H), 8.15 (d, *J* = 78.2 Hz, 2H), 7.90 (dd, *J* = 7.3, 1.5 Hz, 2H), 7.75 (t, *J* = 7.4 Hz, 2H), 7.46 (dt, *J* = 8.3, 1.3 Hz, 2H), 7.30 (s, 2H), 7.14 (dt, *J* = 8.2, 1.1 Hz, 2H), 4.29 (s, 6H), 4.21 (s, 6H). ^{13}C-NMR (126 MHz, CD_2Cl_2): δ = 199.8 (C), 158.0 (C) , 157.5 (C), 141.1 (C), 131.0 (CH), 130.9 (C), 130.4 (C), 129.7 (CH), 129.3 (C), 128.6 (C), 127.8 (CH), 127.33 (CH), 127.27 (CH), 127.1 (C), 126.9 (C), 125.40 (CH), 125.36 (C), 125.0 (C), 124.8 (CH) , 112.4 (C), 111.5 (C), 98.0 (CH), 57.0 (CH_3), 56.8 (CH_3). HRMS (MALDI, DCTB): *m/z* calcd. for $C_{47}H_{28}O_5$ $[M]^+$: 672.1938; found: 672.1931.

Compound 6b

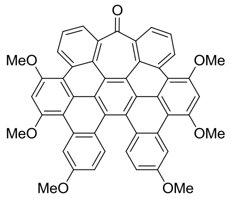

Compound **6b** was prepared from **5n** according to general procedure IIIa. (Eluent mixtures: CH_2Cl_2/hexane: 7/3). Yield: 30%, red solid.

^{1}H-NMR (400 MHz, CD_2Cl_2): δ = 9.57 (d, *J* = 8.3 Hz, 2H), 8.76 (d, *J* = 2.3 Hz, 2H), 8.13 (d, *J* = 9.1 Hz, 2H), 7.90 (d, *J* = 7.3 Hz, 2H), 7.71 (t, *J* = 8.3 Hz, 2H), 7.27 (s, 2H), 6.80 (dd, *J* = 9.1, 2.3 Hz, 2H), 4.28 (s, 6H), 4.19 (s, 6H), 3.95 (s, 6H). ^{13}C-NMR (101 MHz, CD_2Cl_2): δ = 199.6 (C), 158.8 (C), 158.0 (C), 157.5 (C), 140.9 (C), 131.9 (CH), 130.6 (C), 130.3 (C), 129.7 (CH), 129.0 (C), 127.4 (C), 127.1 (CH), 126.3 (C), 125.2 (C), 125.0 (CH), 124.7 (C), 124.5 (C), 114.1 (CH), 112.4 (C), 111.7 (C), 110.4 (CH), 97.9 (CH), 57.0 (CH_3), 56.9 (CH_3), 55.8 (CH_3). HRMS (MALDI, DCTB): *m/z* calcd. for $C_{49}H_{32}O_7$ $[M]^+$: 732.2143; found: 732.2129.

Compound 6c

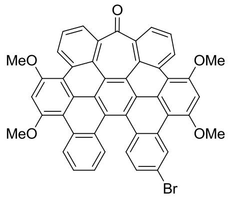

Compound **6c** was prepared from **5k** according to general procedure IIIa. (Eluent mixtures: CH_2Cl_2/hexane: 6/4). Yield: 34%, red solid.

^{1}H-NMR (600 MHz, $C_2D_2Cl_4$): δ = 9.61 (dd, *J* = 8.3, 1.3 Hz, 1H), 9.58 (dd, *J* = 8.3, 1.3 Hz, 1H), 9.44 (d, *J* = 1.9 Hz, 1H), 9.19 (d, *J* = 8.7 Hz, 1H), 8.16 – 8.11 (m, 2H), 7.97 (d, *J* = 7.2 Hz, 1H), 7.93 (d, *J* = 6.7 Hz, 1H), 7.78 (t, *J* = 8.1 Hz, 2H), 7.50 (t, *J* = 7.9 Hz, 1H), 7.28 – 7.24 (m, 3H), 7.21 (t, *J* = 7.1 Hz, 1H), 4.32 (s, 3H), 4.31 (s, 3H), 4.22 (s, 6H). Good quality ^{13}C NMR was not obtained due to low solubility. HRMS (MALDI, DCTB): *m/z* calcd. for $C_{47}H_{27}BrO_5$ $[M]^+$: 750.1036; found: 750.1037.

Compound 6d

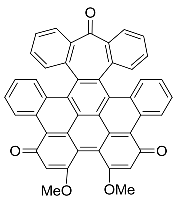

Compound **6d** was prepared from **5g** according to general procedure IIIa. (Eluent mixtures: CH_2Cl_2/hexane: 8/2). Yield: 65%, black solid.

^{1}H-NMR (400 MHz, CD_2Cl_2): δ = 9.72 (bs, 2H), 7.80 (d, *J* = 7.6 Hz, 2H), 7.62 (d, *J* = 7.2 Hz, 2H), 7.52 (t, *J* = 7.6 Hz, 2H), 7.39 (d, *J* = 8.1 Hz, 2H), 7.20 – 7.11 (m, 4H), 6.90 (d, *J* = 7.6 Hz, 2H), 4.06 (s, 6H). ^{13}C-NMR (101 MHz, CD_2Cl_2): δ = 198.8 (C), 188.1 (C), 166.1 (C), 146.0 (C), 136.5 (C), 135.6 (CH), 135.5 (C), 132.1 (C), 131.1 (C), 130.1 (CH), 129.7 (CH), 129.5 (CH), 128.8 (CH), 128.6 (C), 126.0 (CH), 125.9 (CH), 107.2 (CH), 56.9 (CH_3), (five carbon signals were not observed). HRMS (MALDI, DCTB): *m/z* calcd. for $C_{45}H_{24}O_5$ $[M]^+$: 644.1618; found: 644.1610.

Compound 6e

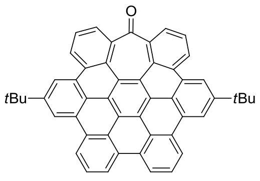

Compound **6e** was prepared from **5e** according to general procedure IIIb. (Eluent mixtures: CH_2Cl_2/hexane: 4/6). Yield: 59%, yellow solid.

^{1}H NMR (500 MHz, $CDCl_3$) δ = 8.91 (dd, *J* = 8.2, 1.6, 2H), 8.75 (d, *J* = 1.2, 2H), 8.67 (d, *J* = 1.8, 2H), 8.59 (d, *J* = 7.8, 2H), 8.57 (d, *J* = 7.9, 2H), 7.88 (dd, *J* = 7.2, 1.4, 2H), 7.83 (t, *J* = 7.6, 2H), 7.75 (t, *J* = 7.8, 2H), 1.60 (s, 18H). ^{13}C NMR (126 MHz, $CDCl_3$) δ = 203.0 (C), 149.9 (C), 142.6 (C), 131.5 (C), 130.2 (C), 129.8 (C), 129.6 (C), 128.3 (C), 127.6 (C), 127.5 (CH), 127.1 (CH), 126.4 (CH), 124.7 (C), 124.3 (C), 123.9 (CH), 123.2 (C), 123.1 (C), 121.8 (CH), 121.4 (CH), 121.0 (C), 120.7 (CH), 118.1 (CH), 35.6 (C), 31.9 (CH_3). HRMS (MALDI, DCTB): *m/z* calcd. for $C_{51}H_{34}O$ $[M]^+$: 662.2604; found: 662.2609.

Compound 6f

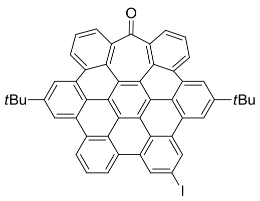

Compound **6f** was prepared from **5l** according to general procedure IIIb. (Eluent mixtures: CH_2Cl_2/hexane: 1/1). Yield: 87%, yellow solid.

^{1}H NMR (400 MHz, $CDCl_3$) δ = 8.76 – 8.67 (m, 4H), 8.58 (s, 2H), 8.45 (d, *J* = 7.7, 2H), 8.26 (d, *J* = 7.8, 1H), 8.05 (d, *J* = 7.9, 1H), 7.86 – 7.80 (m, 2H), 7.75 – 7.61 (m, 2H), 7.38 (t, *J* = 7.8, 1H), 1.65 (s, 9H), 1.53 (s, 9H). ^{13}C NMR (101 MHz, $CDCl_3$) δ = 202.6 (C), 150.2 (C), 150.0 (C), 142.6 (C), 142.5 (C), 132.1 (C), 131.6 (C), 131.4 (C), 131.3 (C), 130.4 (CH), 130.2 (CH), 130.1 (C), 129.4 (C), 128.4 (C), 128.29 (C), 128.26 (C), 128.2 (C), 127.62 (CH), 127.61 (CH), 127.4 (C), 127.35 (C), 127.26 (CH), 126.37 (CH), 126.36 (CH), 125.1 (C), 124.9 (C), 124.3 (C), 124.2 (CH), 124.1 (CH), 123.5 (C), 123.3 (C), 123.1 (C), 123.0 (C), 122.8 (C), 122.4 (CH), 121.4 (CH), 120.93 (CH), 120.87 (C), 120.6 (CH), 120.4 (C), 118.9 (CH), 118.4 (CH), 94.1 (C), 35.8 (C), 35.7 (C), 32.0 (CH_3), 31.9 (CH_3). HRMS (MALDI, DCTB): *m/z* calcd. for $C_{51}H_{33}IO$ $[M]^+$: 788.1571; found: 788.1565.

Compound 6g

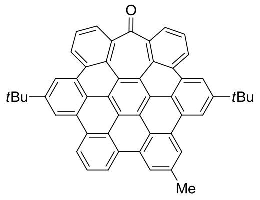

Compound **6g** was prepared from **5m** according to general procedure IIIb. (Eluent mixtures: CH_2Cl_2/hexane: 6/4). Yield: 50%, yellow solid.

^{1}H NMR (600 MHz, $CDCl_3$) δ = 8.80 (t, *J* = 8.9 Hz, 2H), 8.70 (s, 1H), 8.66 (d, *J* = 7.5 Hz, 2H), 8.57 (s, 1H), 8.39 (d, *J* = 7.6 Hz, 1H), 8.32 – 8.28 (m, 2H), 8.12 (s, 1H), 7.86 (dd, *J* = 6.7, 3.0 Hz, 2H), 7.75 – 7.70 (m, 2H), 7.51 (t, *J* = 7.6 Hz, 1H), 2.49 (s, 3H), 1.64 (s, 9H), 1.58 (s, 9H). ^{13}C NMR (151 MHz, $CDCl_3$) δ = 203.1 (C), 149.64 (C), 149.57 (C), 142.5 (C), 142.4 (C), 136.6 (C), 131.4 (C), 131.3 (C), 130.0 (C), 129.9 (C), 129.60 (C), 129.56 (C), 129.5 (C), 129.4 (C), 128.24 (C), 128.18 (C), 127.53 (C), 127.51 (C), 127.3 (CH), 127.2 (CH), 126.8 (CH), 126.2 (CH), 124.6 (C), 124.2 (C), 124.1 (C), 123.9 (CH), 123.8 (CH), 123.2 (C), 123.04 (C), 122.98 (C), 122.7 (C), 122.4 (CH), 122.2 (C), 122.0 (CH), 121.3 (CH), 121.1 (CH), 121.0 (C), 120.6 (C), 120.5 (CH), 120.3 (CH), 118.0 (CH), 117.9 (CH), 35.7 (C), 35.6 (C), 32.0 (CH_3), 31.9 (CH_3), 22.4 (CH_3), (one signal was not observed). HRMS (MALDI, DCTB): *m/z* calcd. for $C_{52}H_{36}O$ $[M]^+$: 676.2761; found: 677.2754.

Compound 6h

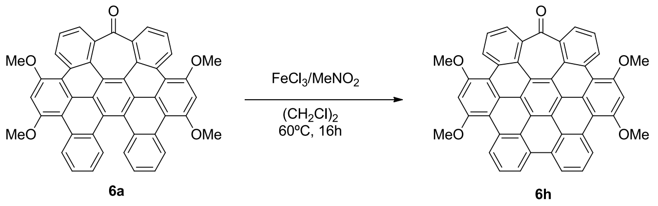

A suspension of compound **6a** (20 mg, 0.030 mmol) in dry $(CH_2Cl)_2$ (20 mL) was degassed by argon bubbling for 15 min. Then, a solution of $FeCl_3$ (15 mg, 0.090 mmol) in nitromethane (0.15 mL) was added dropwise during 30 min. After stirring at 60°C for 16 h under continuous bubbling with argon, the reaction was quenched by the addition of methanol. Then, the mixture was diluted with CH_2Cl_2, washed with water and brine, dried over anhydrous Na_2SO_4 and the solvent was removed under reduced pressure. The residue was purified by column chromatography (CH_2Cl_2/hexane: 7/3) to give the corresponding product **6h** (12 mg, 60%) as a yellow solid.

^1H NMR (400 MHz, CD_2Cl_2) δ = δ 9.69 (dd, *J* = 5.9, 4.0 Hz, 2H), 9.35 (d, *J* = 8.0 Hz, 2H), 9.00 (d, *J* = 8.0 Hz, 2H), 8.06 (t, *J* = 8.0 Hz, 2H), 7.79 (d, *J* = 5.9 Hz, 2H), 7.78 (d, *J* = 4.0 Hz, 2H), 7.25 (s, 2H), 4.22 (s, 6H), 4.21 (s, 6H). ^{13}C NMR (126 MHz, CD_2Cl_2) δ = 202.3 (C), 157.6 (C), 157.0 (C), 143.0 (C), 131.3 (C), 130.3 (C), 129.3 (C), 128.6 (C), 128.5 (CH), 127.7 (CH), 126.9 (CH), 126.6 (CH), 126.4 (C), 124.4 (C), 124.2 (C), 124.1 (C), 123.5 (CH), 120.5 (C), 120.3 (CH), 111.8 (C), 110.4 (C), 97.7 (CH), 56.9 (CH_3), 56.6 (CH_3). HRMS (MALDI, DCTB): *m/z* calcd. for $C_{47}H_{26}O_5$ $[M]^+$: 670.1775; found: 670.1754.

- **General procedure IV: to obtain compounds type 7**

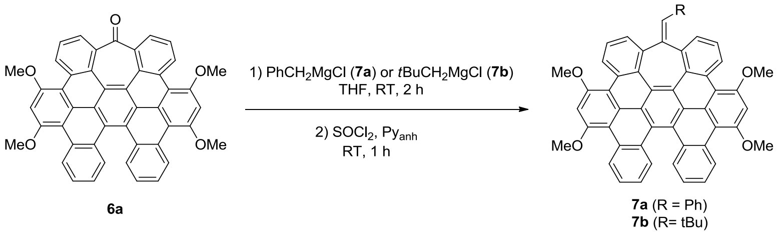

A benzyl or 2,2-dimethylpropylmagnesium chloride solution (1 M in THF, 5 equiv.) was added to a degassed solution of the compound **6a** (0.149 mmol, 1 equiv.) in dry THF (20 mL). The reaction was stirred 2 h under argon atmosphere at room temperature. The mixture was then diluted with CH_2Cl_2, washed with water and HCl (10%), dried over anhydrous Na_2SO_4 and the solvent was removed under reduced pressure. Then, the residue was dissolved in anhydrous pyridine (2 mL) and thionyl chloride (2 equiv.) was slowly added. The reaction mixture was stirred 1 h at room temperature under argon atmosphere. The mixture was then diluted with CH_2Cl_2, washed with water and HCl (10%), dried over anhydrous Na_2SO_4 and the solvent was removed under reduced pressure. The residue was purified by column chromatography (CH_2Cl_2/hexane mixtures) to give the corresponding products that were characterized by ^1H-RMN, ^{13}C-RMN and HRMS.

Compound 7a

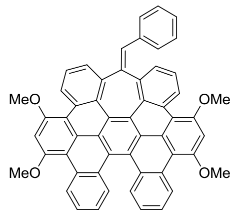

(Eluent mixtures: CH_2Cl_2/hexane: 7/3). Yield: 54% (2 steps), yellow solid.

^{1}H-NMR (600 MHz, CD_2Cl_2): δ = 9.50 (dd, *J* = 8.3, 1.2 Hz, 1H), 9.42 (dd, *J* = 8.3, 1.2 Hz, 1H), 9.21 (d, *J* = 8.3 Hz, 1H), 9.17 (d, *J* = 8.3 Hz, 1H), 8.10 (d, *J* = 8.3 Hz, 2H), 7.76 (t, *J* = 7.2 Hz, 1H), 7.69 (dd, *J* = 7.2, 1.3 Hz, 1H), 7.65 (t, *J* = 7.3 Hz, 1H), 7.43 (dt, *J* = 7.1, 1.1, 2H), 7.39 (dd, *J* = 7.2, 1.2 Hz, 1H), 7.29 (s, 1H), 7.25 (s, 1H), 7.13 – 7.09 (m, 2H), 6.92 – 6.85 (m, 3H), 6.55 (d, *J* = 7.1 Hz, 2H), 6.29 (s, 1H), 4.27 (s, 3H), 4.25 (s, 3H), 4.21 (s, 3H), 4.18 (s, 3H). ^{13}C-NMR (151 MHz, CD_2Cl_2): δ = 157.5 (C), 157.44 (C), 157.43 (C), 145.0 (C), 144.5 (C), 139.9 (C), 137.0 (C), 131.5 (C), 131.4 (CH), 131.3 (CH), 131.1 (C), 131.0 (C), 130.6 (C), 129.5 (C), 129.4 (C), 129.3 (CH), 128.8 (CH), 128.6 (CH), 128.3 (CH), 128.1 (CH), 127.74 (C), 127.70 (C), 127.48 (CH), 127.46 (CH), 127.12 (C), 127.11 (C), 127.01 (CH), 126.96 (CH), 126.93 (CH), 126.4 (C), 126.2 (C), 125.2 (CH), 125.13 (CH), 125.09 (CH), 125.0 (CH), 124.62 (C), 124.58 (C), 124.1 (CH), 123.3 (CH), 112.9 (C), 112.7 (C), 111.9 (C), 111.8 (C), 98.23 (CH), 98.15 (CH), 57.1 (CH_3), 56.79 (CH_3) , 56.75 (CH_3), (four carbon signals were not observed). HRMS (MALDI, DCTB): *m/z* calcd. for $C_{54}H_{34}O_4$ $[M]^+$: 746.2452; found: 746.2445.

Compound 7b

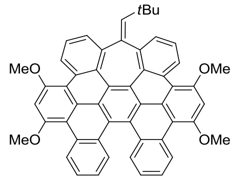

(Eluent mixtures: CH_2Cl_2/hexane: 6/4). Yield: 47% (2 steps), yellow solid.

^{1}H NMR (300 MHz, CD_2Cl_2): δ = 9.46 – 9.32 (m, 2H), 9.19 (d, *J* = 8.2 Hz, 2H), 8.08 (t, *J* = 9 Hz, 2H), 7.69 (d, *J* = 7.3 Hz, 2H), 7.60 - 7.49 (m, 2H), 7.47 - 7.38 (m, 2H), 7.26 (s, 2H), 7.18 - 7.08 (m, 2H), 5.29 (s, 1H), 4.27 (s, 3H), 4.25 (s, 3H), 4.20 (s, 3H), 4.17 (s, 3H), 0.55 (s, 9H). Good quality ^{13}C NMR was not obtained due to low solubility. HRMS (MALDI, DCTB): *m/z* calcd. for $C_{52}H_{38}O_4$ $[M]^+$: 726.2765; found: 726.2771.

Compound 8a

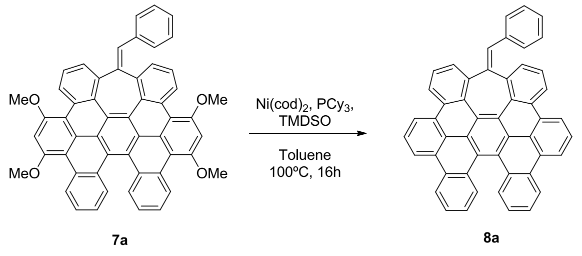

An oven-dried screw-cap test tube containing a stirring bar was charged with the aryl ether **7a** (20 mg, 0.027 mmol), $Ni(cod)_2$ (5 mol%) and PCy_3 (10 mol%) inside the drybox. Then, the flask was removed from the drybox and 1,1,3,3-tetramethyldisiloxane (TMDSO) (20 µL, 0.108 mmol) and toluene (1 mL) were added by syringe under a positive argon atmosphere. The mixture was stirred and refluxed overnight under argon atmosphere. The mixture was then allowed to warm to room temperature and the solvent was removed under reduced pressure. The residue was adsorbed on silica gel and purified firstly by column chromatography (CH_2Cl_2/hexane: 1/9) and then by preparative TLC (CH_2Cl_2/hexane: 3/7) to give the corresponding compound **8a** (13.5 mg, 80%) as a yellow solid.

^{1}H NMR (300 MHz, CD_2Cl_2) δ = 9.02 – 8.75 (m, 6H), 8.65 (t, *J* = 9.8 Hz, 2H), 8.21 – 8.00 (m, 4H), 7.93 (t, *J* = 7.6 Hz, 1H), 7.87 – 7.81 (m, 1H), 7.78 (t, *J* = 7.9 Hz, 1H), 7.66 – 7.50 (m, 3H), 7.22 (t, *J* = 7.4 Hz, 2H), 6.93 – 6.83 (m, 3H), 6.57 – 6.49 (m, 2H), 6.33 (s, 1H). ^{13}C NMR (126 MHz, CD_2Cl_2) δ = 144.4, 136.7, 132.3, 130.8, 129.9, 129.5, 129.0, 128.4, 128.2, 127.7, 127.2, 126.3, 124.1, 122.7, 122.5, 122.4. Some carbon signals could not be listed due to the overlapping observed. HRMS (MALDI, DCTB): *m/z* calcd. for $C_{50}H_{26}$ $[M]^+$: 626.2029; found: 626.2043.

- <u>**General procedure V:**</u> **Ni-catalyzed Kumada-Tamao-Corriu coupling reaction to obtain compounds type 9**

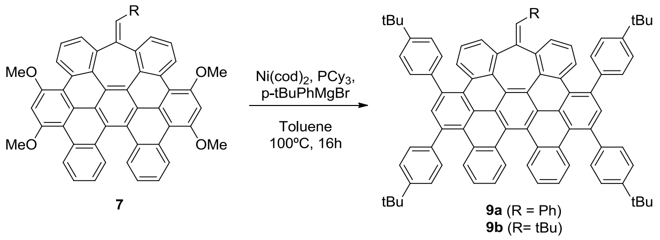

A solution of *p-tert*butylmagnesium bromide (0.5 M in 2-MeTHF) (6 equiv.) was added to a degassed suspension of compound **7** (0.026 mmol, 1 equiv.), $Ni(cod)_2$ (0.05 equiv.) and PCy_3 (0.10 equiv.) in anhydrous toluene (1.5 mL). The reaction mixture was stirred and refluxed overnight under argon atmosphere. The mixture was then cooled to room temperature and the solvent was removed under reduced pressure. The residue was adsorbed on silica gel and purified firstly by column chromatography (CH_2Cl_2/hexane mixtures) and then by preparative TLC (CH_2Cl_2/hexane mixtures) to give the corresponding products that were characterized by ^{1}H-RMN, ^{13}C-RMN and HRMS.

Compound 9a

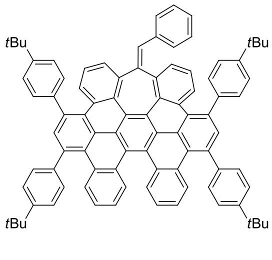

Compound **9a** was prepared from **7a** according to general procedure V. (Eluent mixtures: Column Chromatography: CH_2Cl_2/hexane: 5/95; Preparative TLC: CH_2Cl_2/hexane: 4/6). Yield: 79%, yellow solid.

^{1}H-NMR (500 MHz, CD_2Cl_2): δ = 8.43 (d, J = 8.2, 1H), 8.39 (d, J = 8.2, 1H), 8.09 (d, J = 8.2, 2H), 7.96 (s, 1H), 7.90 (s, 1H), 7.88 (d, J = 8.3, 1H), 7.78 (d, J = 8.2, 1H), 7.75 – 7.69 (m, 3H), 7.69 – 7.64 (m, 2H), 7.64 – 7.55 (m, 6H), 7.55 – 7.45 (m, 4H), 7.42 – 7.33 (m, 2H), 7.30 (d, J = 7.0, 2H), 7.24 – 7.13 (m, 3H), 7.05 (t, J = 7.5, 2H), 6.95 - 6.85 (m, 3H), 6.51 (d, J = 7.2, 2H), 6.31 (s, 1H), 1.45 (s, 18H), 1.39 (s, 9H), 1.37 (s, 9H). ^{13}C-NMR (126 MHz, CD_2Cl_2): δ = 151.2 (C), 151.18 (C), 151.17 (C), 151.13 (C), 145.5 (C), 144.4 (C), 141.8 (C), 141.7 (C), 141.4 (C), 140.1 (C), 138.3 (C), 138.2 (C), 138.1 (C), 137.8 (C), 136.8 (C), 135.0 (CH), 134.9 (CH), 133.5 (C), 133.0 (C), 132.5 (C), 132.2 (C), 131.8 (C), 131.7 (C), 131.4 (CH), 131.2 (CH), 130.6 (C), 130.4 (CH), 130.1 (CH), 129.99 (CH), 129.97 (CH) 129.96 (CH), 129.85 (CH), 129.84 (CH), 129.6 (CH), 129.4 (C), 129.3 (CH), 129.2 (C), 128.9 (CH), 128.83 (CH), 128.81 (CH), 128.5 (C), 128.2 (CH), 127.7 (C), 127.5 (CH), 127.4 (CH), 127.2 (CH), 127.1 (C), 127.0 (C), 126.9 (C), 126.8 (C), 126.7 (CH), 126.68 (CH), 126.63 (CH), 126.54 (CH), 126.52 (CH), 126.4 (C), 126.1 (CH), 126.0 (CH), 125.98 (CH), 125.95 (CH), 125.5 (CH), 124.9 (C), 124.7 (C), 124.2 (CH), 35.2 (C), 35.18 (C), 35.11 (C), 35.10 (C), 31.8 (CH_3), 31.73 (CH_3), 31.71 (CH_3). Some carbon signals could not be listed due to the overlapping observed. HRMS (MALDI, DCTB): m/z calcd. for $C_{90}H_{74}$ $[M]^{+}$: 1154.5785; found: 1154.5756.

Compound 9b

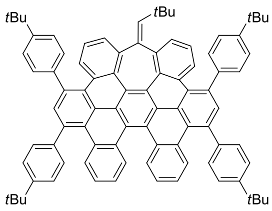

Compound **9b** was prepared from **7b** according to general procedure V. (Eluent mixtures: CH_2Cl_2/hexane: 2/98). Yield: 59%, yellow solid.

^{1}H NMR (400 MHz, CD_2Cl_2): δ = 8.39 (t, J = 8.5 Hz, 2H), 8.01 (d, J = 8.4 Hz, 1H), 7.94 (d, J = 8.2 Hz, 1H), 7.90 (s, 1H), 7.84 (s, 1H), 7.83 (d, J = 6.7 Hz, 1H), 7.80 (d, J = 8.2 Hz, 1H), 7.70 (d, J = 8.2 Hz, 2H), 7.66 (d, J = 7.8 Hz, 2H), 7.59 (d, J = 8.2 Hz, 4H), 7.53 - 7.40 (m, 8H), 7.29 (t, J = 7.7 Hz, 2H), 7.24 (t, J = 8.0 Hz, 2H), 7.18 (dt, J = 7.6, 2.7 Hz, 2H), 7.05 (t, J = 7.7 Hz, 2H), 5.32 (s, 1H), 1.45 (s, 18H), 1.40 (s, 9H), 1.36 (s, 9H), 0.56 (s, 9H). ^{13}C NMR (101 MHz, CD_2Cl_2): δ = 151.2 (C), 151.1 (C), 151.0 (C), 146.9 (C), 141.99 (C), 141.96 (CH), 141.6 (C), 141.5 (C), 140.5 (C), 139.8 (C), 138.4 (C), 138.1 (C), 138.0 (C), 137.7 (C), 135.3 (CH), 134.7 (CH), 133.0 (C), 132.8 (C), 131.7 (C), 131.5 (CH), 131.3 (CH), 130.9 (C), 130.4 (C), 130.2 (C), 130.1 (C), 129.9 (CH), 129.9 (CH), 129.7 (CH) ,129.1 (C), 128.7 (C), 128.5 (C), 128.3 (CH), 127.8 (CH), 127.6 (C), 127.1 (CH), 126.9 (C), 126.81 (C), 126.76 (C), 126.6 (CH), 126.5 (CH), 126.0 (CH), 125.96 (CH), 125.89 (CH), 125.86 (CH), 125.83 (CH), 124.6 (C), 124.5 (C), 124.1 (CH), 35.18 (C), 35.11 (C), 35.08 (C), 33.9 (C), 31.8 (CH_3), 31.75 (CH_3), 31.71 (CH_3), 31.1 (CH_3). Some carbon signals could not be listed due to the overlapping observed. HRMS (MALDI, DCTB): m/z calcd. for $C_{88}H_{78}$ $[M]^{+}$: 1134.6098; found: 1134.6086.

- <u>General procedure VI</u>: **cyclodehydrogenation reaction to obtain compounds 1 and 2**

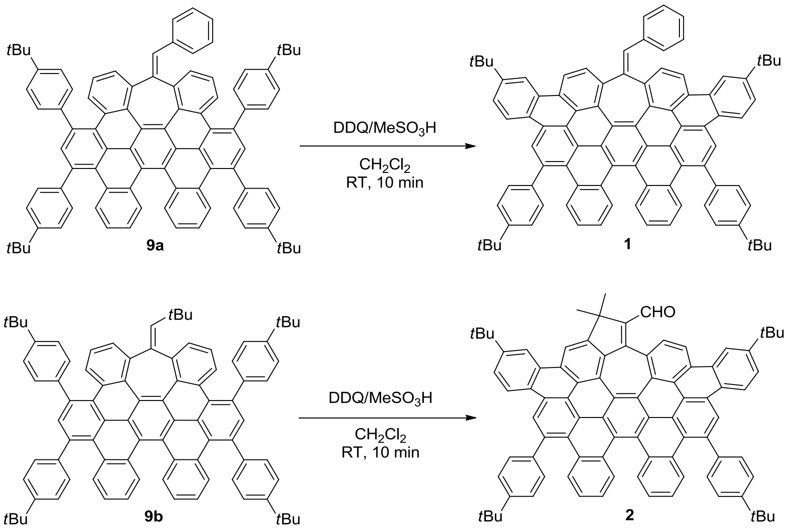

To a solution of the compound **9** (0.043 mmol, 1equiv.) and 2,3-dichloro-5,6-dicyano-1,4-benzoquinone (DDQ) (0.130 mmol, 3 equiv.) in dry CH_2Cl_2 (2 mL), methanesulphonic acid (0.05 mL) was slowly added. The reaction was stirred for 10 min under argon atmosphere at room temperature. The mixture was then diluted with CH_2Cl_2, washed with water, dried over anhydrous Na_2SO_4 and the solvent was removed under reduced pressure. The residue was purified firstly by column chromatography (CH_2Cl_2/hexane mixtures) and then by preparative TLC (CH_2Cl_2/hexane mixtures) to give the corresponding products that were characterized by ^{1}H-RMN, ^{13}C-RMN and HRMS.

Compound 1

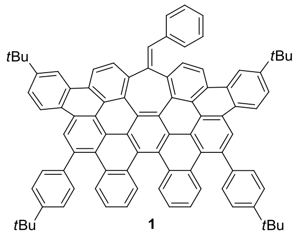

(Eluent mixtures: Column chromatography: CH_2Cl_2/hexane: 5/95; Preparative TLC: CH_2Cl_2/hexane: 4/6). Yield: 47%, orange solid.

^{1}H-NMR (500 MHz, $C_2D_2Cl_4$, 369 K): δ = 9.26 (d, *J* = 8.1 Hz, 1H), 9.23 (s, 1H), 9.19 (s, 1H), 9.17 (d, *J* = 8.3 Hz, 1H), 8.95 – 8.93 (m, 3H), 8.90 (d, *J* = 8.6 Hz, 1H), 8.60 (dd, *J* = 11.6, 8.5 Hz, 2H), 8.44 (d, *J* = 8.1 Hz, 1H), 8.22 (d, *J* = 8.3 Hz, 1H), 8.08 (d, *J* = 8.3 Hz, 1H), 8.02 (d, *J* = 8.3 Hz, 1H), 7.97 – 7.88 (m, 6H), 7.75 – 7.71 (m, 4H), 7.31 – 7.26 (m, 2H), 7.19 – 7.13 (m, 2H), 6.93 (d, *J* = 7.0 Hz, 1H), 6.89 (t, *J* = 7.2 Hz, 2H), 6.68 (d, *J* = 7.2 Hz, 2H), 6.45 (s, 1H), 1.67 (s, 9H), 1.65 (s, 9H), 1.59 (s, 9H), 1.58 (s, 9H). Good quality ^{13}C NMR was not obtained. HRMS (MALDI, DCTB): *m/z* calcd. for $C_{90}H_{70}$ $[M]^+$: 1150.5472; found: 1150.5452.

Compound 2

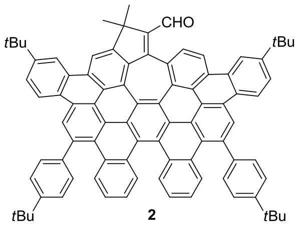

2

(Eluent mixtures: CH_2Cl_2/hexane: 1/9). Yield: 44%, red solid.

^{1}H NMR (500 MHz, DMSO-d$_6$, 348 K): δ = 9.56 (s, 1H), 9.43 (d, *J* = 8.5 Hz, 1H), 9.39 (s, 1H), 9.23 (d, *J* = 6.2 Hz, 2H), 9.22 (s, 1H), 9.11 – 8.99 (m, 4H), 8.48 (d, *J* = 8.4 Hz, 1H), 8.25 (d, *J* = 8.1 Hz, 1H), 8.20 (d, *J* = 8.1 Hz, 1H), 7.96 (d, *J* = 8.6 Hz, 2H), 7.81 – 7.69 (m, 6H), 7.68 - 7.62 (m, 2H), 7.56 (d, *J* = 8.6 Hz, 1H), 7.30 (t, *J* = 7.5 Hz, 1H), 7.21 (t, *J* = 7.6 Hz, 1H), 7.06 (t, *J* = 8 Hz, 1H), 6.95 (t, *J* = 7.6 Hz, 1H), 1.63 (s, 9H), 1.57 (s, 9H), 1.55 (s, 3H), 1.51 (s, 3H), 1.50 (s, 9H), 1.46 (s, 9H). ^{13}C NMR (101 MHz, CD_2Cl_2): δ = 188.9 (CH), 159.4 (C), 154.4 (C), 151.7 (C), 151.4 (C), 151.3 (C), 150.9 (C), 146.7 (C), 144.9 (C), 143.0 (C), 142.0 (C), 139.6 (C), 138.9 (C), 134.6 (C), 132.7 (C), 132.3 (C), 132.2 (C), 132.0 (C), 131.5 (C), 131.3 (CH), 131.1 (CH), 130.9 (CH), 130.8 (CH), 130.6 (C), 130.2 (C), 129.9 (C), 129.7 (CH), 129.6 (C), 129.5 (C), 129.4 (C), 129.3 (C), 128.53 (C), 128.52 (C), 128.0 (C), 127.9 (C), 127.2 (CH), 127.1 (C), 127.0 (CH), 126.8 (CH), 126.7 (CH), 126.6 (C), 126.5 (C), 126.3 (CH), 126.0 (CH), 125.9 (C), 125.7 (C), 125.4 (CH), 124.5 (C), 124.4 (C), 124.3 (CH), 124.1 (C), 124.01 (CH), 123.96 (C), 123.8 (CH), 122.4 (C), 121.1 (C), 120.9 (CH), 120.8 (CH), 114.8 (CH), 49.3 (C), 35.8 (C), 35.7 (C), 35.3 (C), 35.1 (C), 31.9 (CH_3), 31.8 (CH_3), 25.6 (CH_3), 25.5 (CH_3). Some carbon signals could not be listed due to the overlapping observed. HRMS (MALDI, DCTB): *m/z* calcd. for $C_{88}H_{70}O$ $[M]^+$: 1142.5421; found: 1142.5394.

3. Photophysical properties of 1 and 2

Absorption spectra were recorded on a Perkin-Elmer Lambda 650 UV/vis spectrophotometer with a temperature-controlled cell.

Steady-state fluorescence emission spectra were performed on a JASCO FP-6500 spectrofluorometer equipped with a 450 W Xenon lamp for excitation with ETC-273T temperature controller.

Quantum yield values from steady-state fluorescence measurements were calculated for **2** using fluorescein in 0.1M NaOH as a reference (Φ=0.79),[S10] and quinine sulfate in 0.1M H_2SO_4 for **1** as a reference (Φ=0.54).[S11]

Fluorescence decay traces were recorded by via the time-correlated single photon counting (TCSPC)[S10],[S11] method using a FluoTime 200 fluorometer (PicoQuant GmbH, Germany). The excitation source consisted of LDH-405 for **1** and **2** pulsed laser and the observation was performed through a monochromator from 440 to 600nm and 480 to 700 nm every 4nm for **1** and **2**, respectively. The time increment per channel was 36 ps. The pulse repetition rate was 10 MHz. Fluorescence decay histograms were collected in 1320 channels using 10 × 10 mm cuvettes. Histograms of the instrument response functions (using a LUDOX scatterer) and sample decays were recorded until they typically reached to 2×10^4 counts in the peak channel. Two fluorescence decays were recorded for all of the samples. The fluorescence decay traces were individually analyzed using an iteractive deconvolution method with exponential models using FluoFit software (PicoQuant).

Time-resolved emission spectroscopy (TRES) of compounds **1** and **2** dissolved in CH_2Cl_2 was performed by collecting 42 and 57 fluorescence decay traces between 440-600 nm and 480-700 nm respectively to emission range ($\Delta\lambda_{em}$ = 4 nm) during a fixed amount of time, to maintain the overall intensity information.

For the TRES (Time Resolved Emission Spectroscopy) analysis and the estimation of the species-associated emission spectra (SAEMS), the fitting procedure described above was performed, by fitting globally all decay traces. The SAEMS of each species *i* at any given emission wavelength ($SAEMS_i(\lambda_{em})$) is given by the fluorescence intensity emitted by the species *i* ($A_{i,\lambda em} \times \tau_i$), normalized by the total intensity and corrected for the different detection sensitivity using the total intensity of the steady-state spectrum ($I_{ss,\lambda em}$):

$$SAEMS_i(\lambda_{em}) = \frac{A_{i,\lambda em} \times \tau_i}{\sum_i A_{i,\lambda em} \times \tau_i} \cdot I_{ss,\lambda em}$$

The approximate contribution of each species can be assessed as the area under the SAEMS. This estimation assumes equal excitation rate for all the species, as the initial amount of each form in the excited state (after the pulse excitation) is unknown.

Table S1. Optical data for compounds **1** and **2**.

Compound	Absorbance λ_{max} /nm	Fluorescence λ_{max} /nm	Fluorescence Quantum Yields (Φ)	Fluorescence Lifetimes (τ) / ns	
				τ_1	τ_2
**1**	386	493	0.072	14.51	4.14
**2**	389	590	0.075	12.92	3.7

4. Electrochemical measurements of 1 and 2

Cyclic and Square Wave Voltammetry (CV and SWV, respectively) experiments were performed with a three electrode cell under N_2 atmosphere at 25°C. A Pt-mesh counterelectrode and an Ag-wire quasireference electrode were used. The working electrode was a glassy carbon disk. The solvent was CH_2Cl_2 containing 0.15 M tetrabutylammonium hexafluorophostate ($TBAPF_6$) as supporting electrolyte. Potential values are referred to the ferrocenium/ferrocene ($FeCp_2^+/FeCp_2^0$ (Fc = ferrocene) system, as Fc was added as an internal reference after each short series of measurements.

Table S2. Electrochemical data for compounds **1** and **2**.

Compound	E_{ox} V vs Fc	ΔE_{ox}, V	E_{red} V vs Fc	ΔE_{red}, V	GAP homo-lumo, eV
**1**	+0.64, +0.96	0.32	-2.01, -2.36	0.36	2.66
**2**	+0.58; +0.91	0.33	-1.69; -1.95	0.26	2.27

5. Single Crystal X-Ray Analysis

X-Ray Structure Determinations. Crystals of **2**, **6a**, **6d** and **6h** were grown from butanone (**2**, **6a**), dichloromethane (**6d**) and hexane (**6h**) saturated solutions under slow evaporation at room temperature. Measured crystals were prepared under inert conditions immersed in perfluoropolyether as protecting oil for manipulation. Suitable crystals were mounted on MiTeGen Micromounts™ and these samples were used for data collection. Data were collected with a Bruker D8 Venture diffractometer. The data were processed with APEX3 suite (Bruker, APEX3 Software, V2016.1, Bruker AXS Inc., Madison, Wisconsin, USA, 2016). The structures were solved by direct methods,[S12] which revealed the position of all non-hydrogen atoms. These atoms were refined on F^2 by a full-matrix least-squares procedure using anisotropic displacement parameters.[S12] All hydrogen atoms were located in difference Fourier maps and included as fixed contributions riding on attached atoms with isotropic thermal displacement parameters 1.2 times those of the respective atom. For **6b** and **6h**, the solvent masking procedure as implemented in Olex2[S13] was used to remove the electronic contribution of a solvent molecule from the refinement. Crystallographic data for the structures of compounds **2**, **6a**, **6b** and **6h** reported in this paper have been deposited with the Cambridge Crystallographic Data Center as supplementary publication no. CCDC-1485682 (**2**), 1485683 (**6a**), 1485684 (**6b**) and 1485685 (**6h**). Copies of the data can be obtained free of charge at http://www.ccdc.cam.ac.uk/products/csd/request/.

Table S3. Crystal data and structure refinement for compounds **2**, **6a**, **6d** and **6h**.

Identification code	**2**	**6a**	**6d**	**6h**
Empirical formula	$C_{88}H_{70}O$	$C_{47}H_{28}O_5$	$C_{46}H_{22}Cl_2O_5$	$C_{50}H_{33}O_5$
Formula weight	1143.44	672.69	725.53	713.76
Temperature (K)	100.0	100.0	100.0	100.0
Wavelength (Å)	1.54178	1.54178	1.54178	1.54178
Crystal system	Monoclinic	Monoclinic	Triclinic	Triclinic
Space group	$P2_1/c$	$P2_1/c$	P-1	P-1
a (Å)	15.5050(5)	7.5395(5)	11.664(2)	13.1702(8)
b(Å)	34.4906(10)	34.5192(18)	12.471(2)	17.3964(12)
c(Å)	17.2521(5)	11.7823(6)	13.809(3)	17.5084(11)
α(°)	90	90	116.004(15)	63.486(5)
β(°)	112.9897(17)	100.084(3)	114.439(13)	70.365(4)
γ(°)	90	90	91.219(14)	81.999(4)
Volume (Å^3)	8493.2(5)	3019.1(3)	1592.3(6)	3380.5(4)
Z	4	4	2	4
Density (calc.) (Mg/m^3)	0.894	1.480	1.513	1.402
Absorption coefficient (mm^{-1})	0.389	0.764	2.280	0.714
F(000)	2424	1400	744	1492

Crystal size (mm^3)	0.1 x 0.08 x 0.08	0.12 x 0.1 x 0.1	0.1 x 0.08 x 0.06	0.1 x 0.08 x 0.08
2⍰ range for data collection (º)	2.562 to 39.966	2.560 to 66.584	4.037 to 66.593	2.839 to 66.592
Reflections collected	26449	21377	31508	38935
Independent reflections [R(int)]	4676 [0.0707]	5048 [0.0823]	5577 [0.1897]	11864 [0.2037]
Data / restraints / parameters	4676 / 864 / 816	5048 / 0 / 473	5577 / 0 / 454	11864 / 0 / 945
Goodness-of-fit on F^2	1.090	1.017	0.925	0.849
Final R indices [I>2σ(I)]	R_1 = 0.0843, wR_2 = 0.2088	R_1 = 0.0613, wR_2 = 0.1171	R_1 = 0.0718, wR_2 = 0.1540	R_1 = 0.0779, wR_2 = 0.1537
R indices (all data)	R_1 = 0.1182, wR_2 = 0.2232	R_1 = 0.1218, wR_2 = 0.1404	R_1 = 0.1575, wR_2 = 0.1814	R_1 = 0.1842, wR_2 = 0.1873
Extinction coefficient	--	--	0.0013(3)	--
Largest diff. peak and hole(e· $Å^{-3}$)	0.348 and -0.244	0.256 and -0.259	0.234 and -0.232	0.255 and -0.245

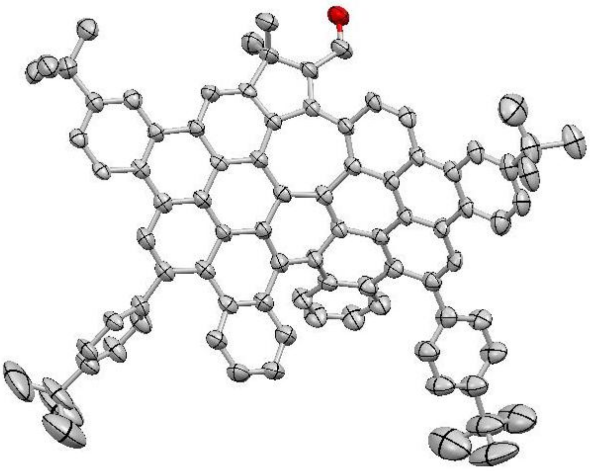

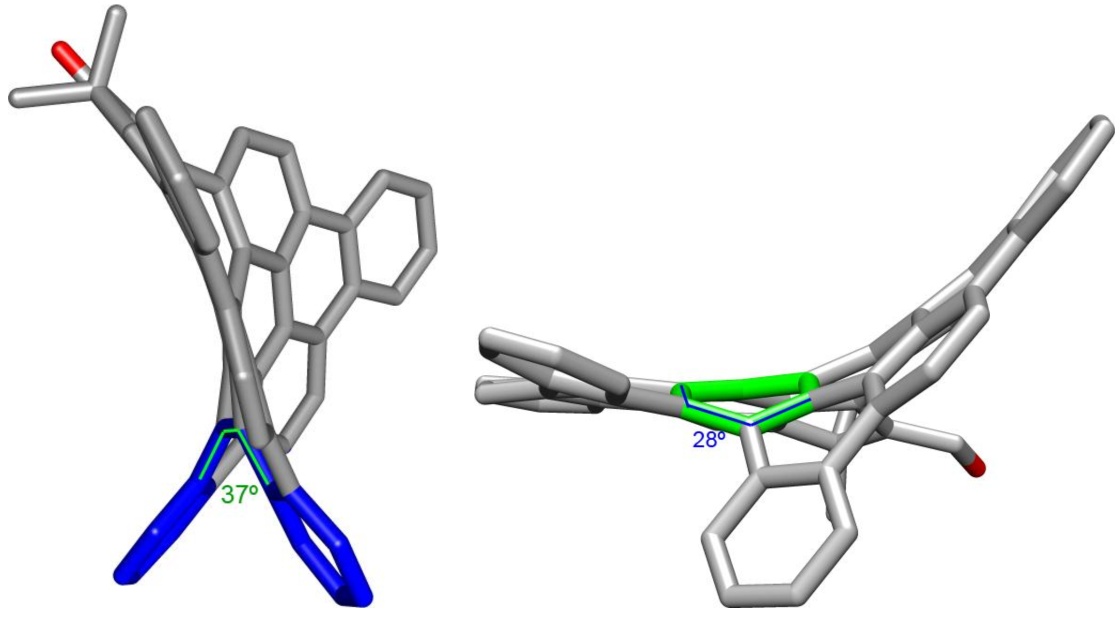

Figure S1. Top: Single crystal X-Ray diffraction structure of compound **2** with ellipsoid probability level shown at 50%. **Bottom:** Detailed on the bending angles of the helicene moiety (left) and a distorted benzene ring (right).

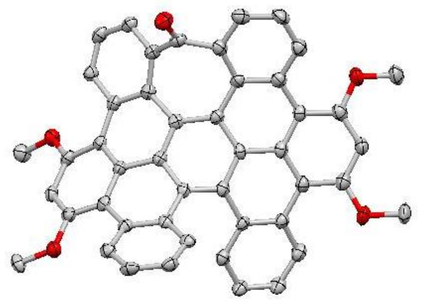

Figure S2. Single crystal X-Ray diffraction structure of compound **6a** with ellipsoid probability level shown at 50%.

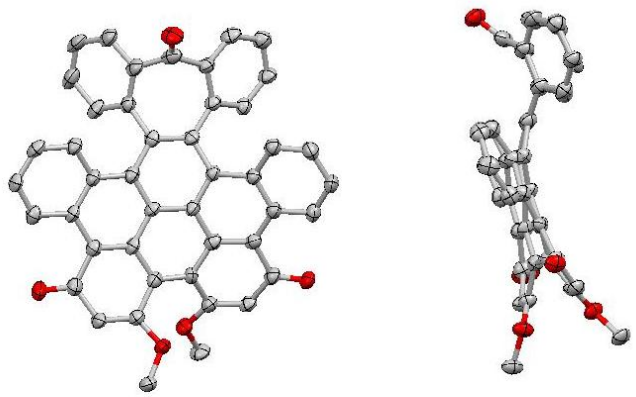

Figure S3. Single crystal X-Ray diffraction structure of compound **6d** with ellipsoid probability level shown at 50%. Left) Front view. Right) Lateral view.

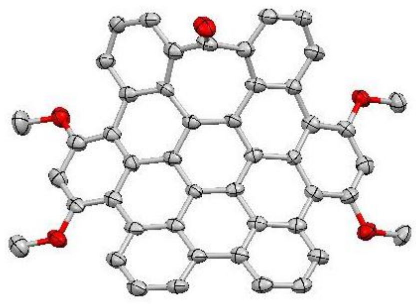

Figure S4. Single crystal X-Ray diffraction structure of compound **6h** with ellipsoid probability level shown at 50%.

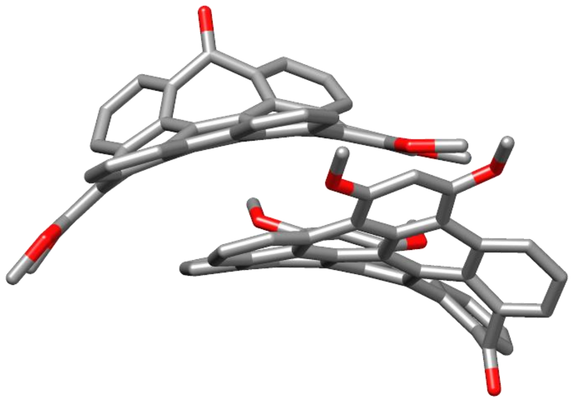

Figure S5. Molecular packing of compound **6h**.

6. Theoretical calculations

6.a. Calculated electronic and transport properties of 6h and its defect-free counterpart coronene derivative S2

Computational Methods: In order to evaluate the theoretical electronic and transport properties of the **6h** nanographene, *ab initio* i.e. Density Functional Theory (DFT)-based calculations were performed by means of the SIESTA[S14] method, using the pseudopotential approximation and the vdW-DF implementation of the functional of Dion *et al*.[S15], [S16] in the optB88-vdW version for the exchange-correlation potential.[S17] The basis set employed was a double-zeta polarized (DZP) that yields an optimised lattice constant of 1.437 Å for two-dimensional graphene.

We first started by characterizing the isolated **6h** nanographene (Fig. S6, top left panel) within the supercell approach, by repeating the unit cell periodically in the three spatial directions to avoid interaction with neighboring supercells. The atomic coordinates were relaxed via total energy minimization until changes in forces were below 0.02 eV/Å. The calculated bandgap for the isolated **6h** structure was of 2.11 eV.

Once the isolated **6h** geometry was optimized we performed the calculation of the electronic and transport properties of the relaxed stacked **6h** structure, following the stacking data obtained by X-ray measurements and relaxing the chain coordinates using the same conditions aforementioned. The final relaxed structure is displayed in the top right panel of Fig. S6. Transport simulations were carried out by means of the TRANSIESTA method.[S18] TheBrillouin zone (BZ) was sampled with a Monkhorst-Pack scheme of 1×1×9 special points for the structure optimization and of 1×1×15 for the calculation of the transport and electronic properties. The calculated gap of the **6h** chain was diminished to 1.71 eV, suggesting the potential of the stacked **6h** nanographenes as active components in nanoelectronic devices. The calculated conductance, density of states and bandstructured of the stacked **6h** nanographenes are shown in the bottom panel of Fig. S6.

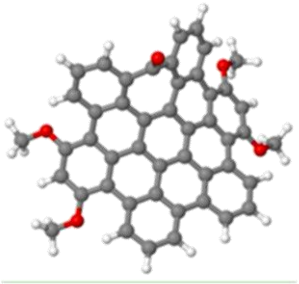

6h

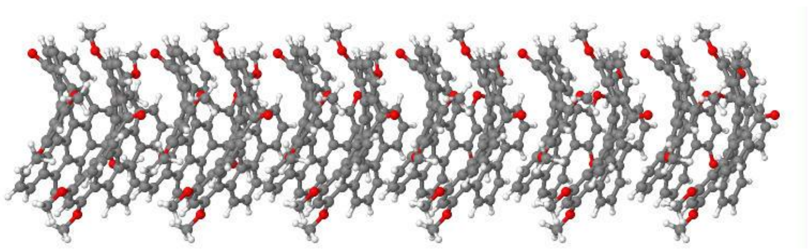

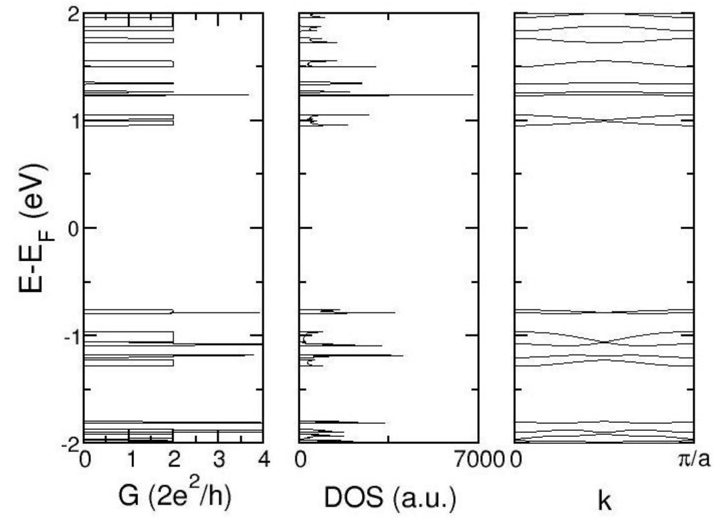

Figure S6. Ball-and-stick model of the **6h** nanographene, both as an isolated molecule (top left) and in the stacked geometry (top right). C, O and H atoms are shown in grey, red and white, respectively. Calculated conductance (bottom left), density of states (bottom center) and bandstructure (bottom right) for the **6h** chain showed in the top right panel.

In order to elucidate on the influence of the defect on the transport and electronic properties of the **6h** nanographene a similar analysis was performed for the coronene derivative **S2**, shown in Fig. S7. It is immediately noticeable that this molecule shows a more planar geometry. The calculated gaps for this compound both as an isolated molecule and in its stacked formed are 2.10 eV and 1.75 eV, showing that the defect has not induced a significant reduction of the bandgap. The calculated conductance (bottom left), density of states (bottom center) and bandstructure (bottom right) of the coronene chain are shown in the bottom panel of Fig. S7. A comparison of these quantities for both chains is displayed in Fig. S8.

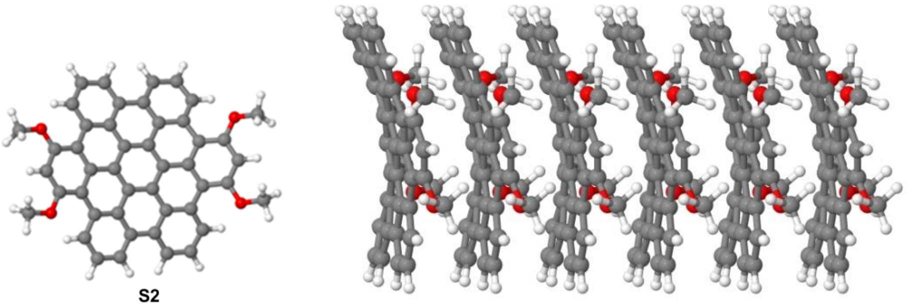

S2

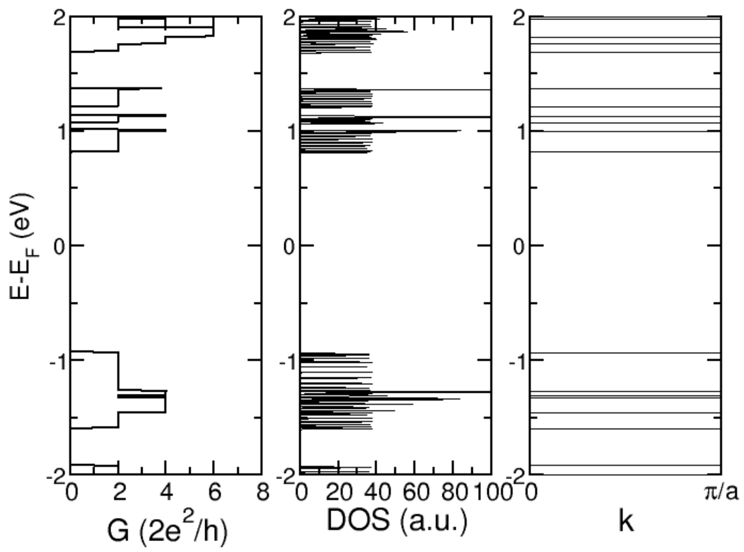

Figure S7. Ball-and-stick model of coronene derivative S2 (non-defect counterpart of the 6h nanographene) both as an isolated molecule (top left) and in the stacked geometry (top right). C, O and H atoms are shown in grey, red and white, respectively. Calculated conductance (bottom left), density of states (bottom center) and bandstructure (bottom right) for the chain showed in the top right panel.

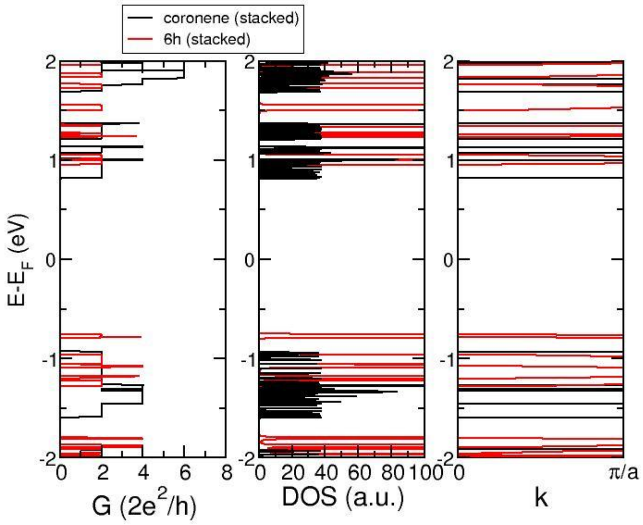

Figure S8. Comparison of the calculated conductance (bottom left), density of states (bottom center) and bandstructure (bottom right) for the stacked structures of the **6h** nanographene (red) and its non-defect counterpart **S2** (black).

6.b. Activation barriers for different protonation/cyclizations sites in simplified models of compounds 9a and 9b.

Computational Methods:

Model compounds of compounds **9a** and **9b** were use to make the calculations much less time consuming. All structures were initially optimized using density functional theory (DFT) with B3LYP[S19] and the 6-31G(d,p) basis set as implemented in Gaussian 09.[S20] Further optimizations were carried out at M06-2X/6-311+G(d,p) level of theory,[S21] in a solvent model (IEFPCM, solvent=acetonitrile).[S22] The stationary points were characterized by frequency calculations in order to verify that they have the right number of imaginary frequencies.

The rearrangement of the methyl group from **I** to **III** is shown in Figure S9 along with a 3D representation of the transition state (**II**). Barrierless cyclization from the tertiary carbocation renders **IV**, model of the final product.

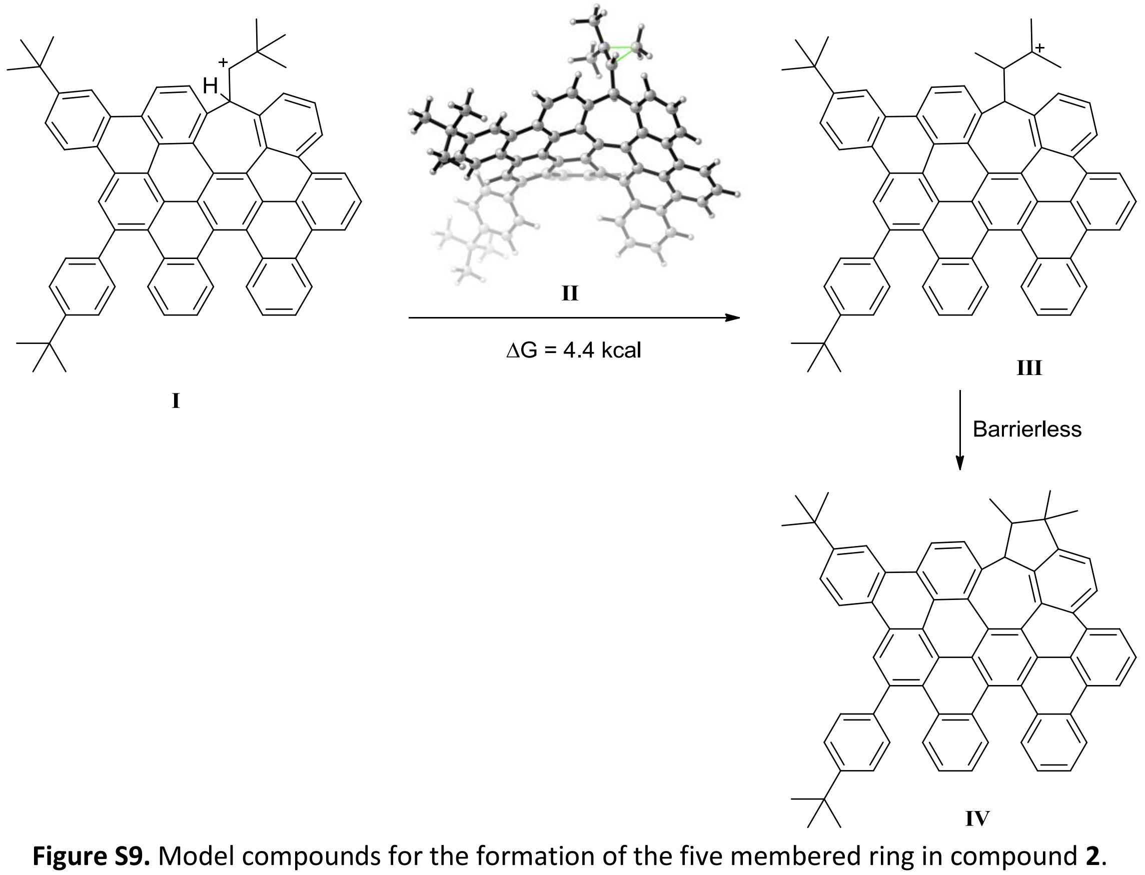

Figure S9. Model compounds for the formation of the five membered ring in compound **2**.

The energies and coordinates of the lowest energy transition state of each ring closure (**V** and **VI**) are also shown below (Tables S4 and S5).

Table S4. Computed absolute electronic energies (hartrees) and relative Free Gibbs energies (kcal/mol) for structure **V**, and imaginary frequencies for the transition states for the blue and red ring closing.

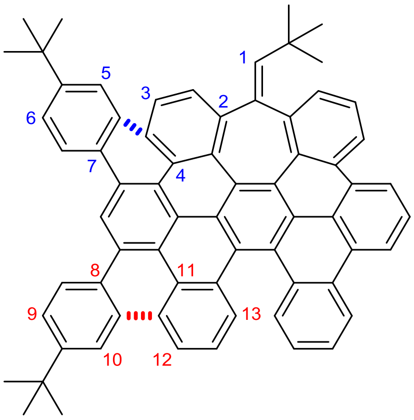

V

Atom nº	E $\mathbf{I}^{H+}$ (M06-2X)	E **TS** (M06-2X)	E (relative)	Freq
1	-2623.848482	-2623.791855	35.5	-520.1
2	-2623.820474	-2623.799736	13.0	-539.6
3	-2623.817500	-2623.780046	23.5	-382.7
4	-2623.794610	-2623.770244	15.3	-382.0
5	-2623.815353	-2623.788571	16.8	-342.8
6	-2623.816589	-2623.792322	15.2	-317.7
7	-2623.807899	-2623.795372	**7.9**	-217.9
8	-2623.808223	-2623.790128	11.4	-292.0
9	n.d.	-2623.791211	n.d.	-352.2
10	-2623.814905	-2623.787788	17.0	-359.0
11	-2623.801469	-2623.778813	14.2	-380.1
12	-2623.822756	-2623.787955	21.8	-619.0
13	-2623.818434	-2623.792483	16.3	-528.4

Table S5. Computed absolute electronic energies (hartrees) and relative Free Gibbs energies (kcal/mol) for structure **VI**, and imaginary frequencies for the transition states for the blue, red and pink ring closing.

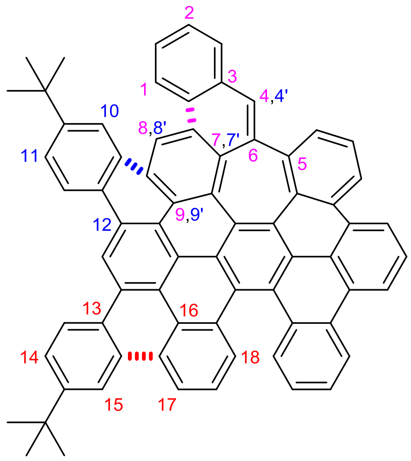

VI

Atom nº	E **II**$^{H+}$ (M06-2X)	E **TS** (M06-2X)	E (releative)	Freq
1	-2697.611101	-2697.585338	16.2	-325.4
2	-2697.611003	-2697.590741	12.7	-292.1
3	-2697.602115	-2697.589576	7.9	-227.9
4	-2697.637016	n.d.	n.d.	n.d.
5	-2697.608912	n.d.	n.d.	n.d.
6	-2697.622632	-2697.588635	21.3	-393.2
7	-2697.621128	-2697.601763	12.2	-402.6
8	-2697.617746	-2697.58174	22.6	-526.3
9	-2697.595011	-2697.555662	24.7	-600.1
4’	-2697.637016	-2697.595972	25.8	-547.1
7’	-2697.621128	-2697.60009	13.2	-498.2
8’	-2697.617746	-2697.589266	17.9	-443.7
9’	-2697.595011	n.d.	n.d.	n.d.
10	-2697.615491	-2697.588429	17.0	-350.6
11	-2697.616914	-2697.592511	15.3	-320.5
**12**	**-2697.608288**	**-2697.595709**	**7.9**	**-216.0**
13	-2697.609386	-2697.591753	11.1	-290.4
14	-2697.61752	-2697.592048	16.0	-352.0
15	-2697.616125	-2697.589287	16.8	-360.8
16	-2697.623605	-2697.59025	20.9	-613.2

17	-2697.620354	-2697.594737	16.1	-521.7
18	-2697.602931	n.d.	n.d.	n.d.

Cartesian Coordinates of the computed structures

Structure **I**

Standard orientation:

Center Number	Atomic Number	Atomic Type	Coordinates (Angstroms) X	Y	Z
1	1	0	-2.111154	5.090965	-0.549048
2	6	0	-2.006124	4.050968	-0.268048
3	6	0	-1.865044	1.289972	0.275952
4	6	0	-3.104105	3.406936	0.268952
5	6	0	-0.756105	3.403004	-0.372048
6	6	0	-0.690065	2.006006	-0.076048
7	6	0	-3.004066	2.044939	0.637952
8	1	0	-4.028121	3.958909	0.397952
9	6	0	0.569955	1.328043	-0.040048
10	6	0	3.042993	0.018114	0.324952
11	6	0	0.599995	-0.036957	0.353952
12	6	0	1.767935	2.031077	-0.294048
13	6	0	2.974955	1.337112	-0.121048
14	6	0	1.839013	-0.679921	0.623952
15	1	0	3.914941	1.825139	-0.349048
16	6	0	1.703894	3.419075	-0.740048
17	6	0	1.552816	6.140071	-1.619048
18	6	0	2.853874	4.132109	-1.138048
19	6	0	0.455875	4.098039	-0.785048
20	6	0	0.419836	5.444038	-1.226048
21	6	0	2.782836	5.444107	-1.561048
22	1	0	3.822888	3.647137	-1.130048
23	1	0	-0.535179	5.948011	-1.274048
24	1	0	3.697821	5.942133	-1.863048
25	6	0	-0.635984	-0.793992	0.321952
26	6	0	-2.812939	-2.327055	-0.572048
27	6	0	-1.911002	-0.166029	0.192952
28	6	0	-0.527943	-2.200989	0.306952
29	6	0	-1.518922	-2.917018	-0.407048
30	6	0	-3.080978	-0.984063	-0.115048
31	6	0	-4.014043	1.244910	1.463952
32	6	0	-4.236074	2.341903	2.388952
33	1	0	-4.948097	3.115883	2.105952
34	6	0	-4.998024	0.598881	0.562952
35	6	0	-6.759985	-0.735170	-1.066048
36	6	0	-6.291038	1.071844	0.423952
37	6	0	-4.492991	-0.533104	-0.149048
38	6	0	-5.469970	-1.278132	-0.876048
39	6	0	-7.171019	0.427818	-0.448048
40	1	0	-6.624062	1.931834	0.996952
41	1	0	-8.176030	0.806789	-0.602048
42	1	0	-7.464970	-1.248190	-1.705048
43	6	0	-3.635079	2.514921	3.731952
44	6	0	-2.447053	1.591955	4.041952

45	1	0	-1.622058	1.762979	3.344952
46	1	0	-2.719022	0.532947	4.004952
47	1	0	-2.084059	1.798966	5.051952
48	6	0	-4.851069	2.146886	4.666952
49	1	0	-5.711088	2.801861	4.505952
50	1	0	-4.511073	2.280895	5.698952
51	1	0	-5.164039	1.107877	4.539952
52	6	0	-3.258122	3.994932	3.966952
53	1	0	-4.100142	4.663907	3.764952
54	1	0	-2.423131	4.287956	3.324952
55	1	0	-2.958126	4.137940	5.008952
56	6	0	-3.855916	-3.143086	-1.173048
57	6	0	-5.925869	-4.759146	-2.217048
58	6	0	-5.165931	-2.621124	-1.349048
59	6	0	-3.592877	-4.485078	-1.590048
60	6	0	-4.644854	-5.272108	-2.075048
61	6	0	-6.177907	-3.441153	-1.875048
62	1	0	-4.467824	-6.303103	-2.353048
63	1	0	-7.188918	-3.071182	-1.979048
64	1	0	-6.726851	-5.388169	-2.592048
65	6	0	1.520774	7.596070	-2.111048
66	1	0	-3.431020	0.473927	1.967952
67	6	0	2.418748	8.463096	-1.196048
68	1	0	3.458758	8.124126	-1.200048
69	1	0	2.409718	9.504096	-1.537048
70	1	0	2.062749	8.443086	-0.161048
71	6	0	0.099756	8.189029	-2.095048
72	1	0	-0.581228	7.640009	-2.754048
73	1	0	-0.328244	8.201017	-1.086048
74	1	0	0.129726	9.224030	-2.448048
75	6	0	2.054772	7.655086	-3.562048
76	1	0	2.045742	8.688085	-3.925048
77	1	0	3.081782	7.288115	-3.637048
78	1	0	1.433789	7.055068	-4.235048
79	6	0	0.595074	-2.778957	1.041952
80	6	0	-1.190885	-4.183008	-1.071048
81	6	0	1.768052	-2.004923	1.245952
82	6	0	0.464110	-4.024961	1.693952
83	1	0	-0.445873	-4.596987	1.559952
84	6	0	2.765066	-2.507894	2.108952
85	1	0	3.647049	-1.913868	2.307952
86	6	0	1.462124	-4.500932	2.525952
87	1	0	1.337151	-5.451935	3.031952
88	6	0	2.618102	-3.733898	2.735952
89	1	0	3.393112	-4.089876	3.407952
90	6	0	-2.223862	-4.992038	-1.599048
91	6	0	4.400011	-0.600847	0.368952
92	6	0	4.694045	-1.761838	-0.364048
93	6	0	5.444993	0.007184	1.073952
94	6	0	5.980060	-2.288801	-0.377048
95	1	0	3.910059	-2.246861	-0.937048
96	6	0	6.733009	-0.533779	1.063952
97	1	0	5.248967	0.905178	1.654952
98	6	0	7.035043	-1.692770	0.338952
99	1	0	6.165086	-3.181795	-0.965048
100	1	0	7.503994	-0.030757	1.634952
101	6	0	8.442061	-2.314730	0.296952

102	6	0	8.947061	-2.341715	-1.166048
103	1	0	9.948074	-2.782686	-1.212048
104	1	0	8.293078	-2.933734	-1.813048
105	1	0	9.003032	-1.330713	-1.581048
106	6	0	9.456038	-1.521700	1.142952
107	1	0	10.438051	-1.999672	1.082952
108	1	0	9.570008	-0.492697	0.784952
109	1	0	9.171037	-1.488708	2.198952
110	6	0	8.380102	-3.760731	0.844952
111	1	0	8.034103	-3.772741	1.882952
112	1	0	7.704121	-4.390751	0.258952
113	1	0	9.373116	-4.220703	0.812952
114	6	0	0.151126	-4.571970	-1.310048
115	1	0	0.958107	-3.916946	-1.018048
116	6	0	0.460160	-5.764961	-1.934048
117	1	0	1.498168	-6.031931	-2.106048
118	6	0	-0.569815	-6.613990	-2.362048
119	1	0	-0.339788	-7.557984	-2.845048
120	6	0	-1.885826	-6.218029	-2.214048
121	1	0	-2.660808	-6.856051	-2.619048

Structure **II**

Standard orientation:

Center Number	Atomic Number	Atomic Type	Coordinates (Angstroms) X	Y	Z
1	1	0	-1.987628	5.202090	0.009983
2	6	0	-1.919698	4.124085	0.095983
3	6	0	-1.845881	1.303081	0.258983
4	6	0	-3.004743	3.427156	0.603983
5	6	0	-0.719741	3.461008	-0.207017
6	6	0	-0.665833	2.046004	-0.044017
7	6	0	-2.953833	2.033152	0.712983
8	1	0	-3.890707	3.980213	0.903983
9	6	0	0.606124	1.379922	-0.037017
10	6	0	3.101042	0.106760	0.334983
11	6	0	0.660036	0.013918	0.343983
12	6	0	1.798171	2.104844	-0.269017
13	6	0	3.015127	1.427766	-0.102017
14	6	0	1.903995	-0.607162	0.625983
15	1	0	3.946160	1.933705	-0.322017
16	6	0	1.721262	3.502849	-0.683017
17	6	0	1.529439	6.237862	-1.490017
18	6	0	2.853309	4.225776	-1.114017
19	6	0	0.476306	4.177930	-0.653017
20	6	0	0.414393	5.527934	-1.064017
21	6	0	2.762394	5.547782	-1.500017
22	1	0	3.820277	3.738713	-1.166017
23	1	0	-0.548575	6.019997	-1.078017
24	1	0	3.662427	6.054724	-1.832017
25	6	0	-0.564015	-0.767002	0.297983
26	6	0	-2.674118	-2.349866	-0.651017
27	6	0	-1.842976	-0.162920	0.131983
28	6	0	-0.430106	-2.173011	0.297983
29	6	0	-1.390155	-2.926949	-0.419017
30	6	0	-2.967030	-0.999847	-0.229017

31	6	0	-4.042887	1.201223	1.372983
32	6	0	-4.692832	2.055265	2.395983
33	1	0	-5.454786	2.760315	2.065983
34	6	0	-4.899926	0.602279	0.290983
35	6	0	-6.509009	-0.676617	-1.549017
36	6	0	-6.142892	1.132359	-0.033017
37	6	0	-4.362000	-0.536756	-0.375017
38	6	0	-5.277047	-1.262697	-1.192017
39	6	0	-6.930931	0.522410	-1.010017
40	1	0	-6.510834	2.031383	0.451983
41	1	0	-7.878903	0.958472	-1.307017
42	1	0	-7.149041	-1.174576	-2.264017
43	6	0	-4.390829	2.100246	3.784983
44	6	0	-3.120870	1.463163	4.333983
45	1	0	-2.290825	2.163109	4.199983
46	1	0	-2.854931	0.527146	3.841983
47	1	0	-3.232883	1.273171	5.403983
48	6	0	-5.667902	0.982328	3.746983
49	1	0	-6.252912	0.827366	2.827983
50	1	0	-6.359877	1.369373	4.493983
51	1	0	-5.215964	0.027299	4.001983
52	6	0	-4.810748	3.359273	4.540983
53	1	0	-5.738720	3.787333	4.151983
54	1	0	-4.021699	4.112222	4.447983
55	1	0	-4.942762	3.143281	5.603983
56	6	0	-3.689171	-3.170800	-1.290017
57	6	0	-5.718276	-4.793668	-2.399017
58	6	0	-4.971136	-2.632717	-1.581017
59	6	0	-3.422259	-4.531817	-1.637017
60	6	0	-4.458310	-5.321750	-2.156017
61	6	0	-5.965189	-3.455652	-2.134017
62	1	0	-4.284378	-6.364761	-2.383017
63	1	0	-6.960164	-3.070588	-2.314017
64	1	0	-6.505317	-5.424617	-2.797017
65	6	0	1.469534	7.702866	-1.954017
66	1	0	-3.528940	0.385190	1.890983
67	6	0	2.397590	8.558806	-1.059017
68	1	0	3.437568	8.222738	-1.107017
69	1	0	2.373657	9.604807	-1.383017
70	1	0	2.080587	8.520826	-0.012017
71	6	0	0.047572	8.287958	-1.872017
72	1	0	-0.655463	7.747003	-2.515017
73	1	0	-0.341429	8.276983	-0.847017
74	1	0	0.059640	9.329957	-2.205017
75	6	0	1.945540	7.793835	-3.424017
76	1	0	1.917607	8.832837	-3.767017
77	1	0	2.971517	7.433768	-3.545017
78	1	0	1.302502	7.200877	-4.082017
79	6	0	0.688859	-2.716084	1.069983
80	6	0	-1.042239	-4.220971	-1.023017
81	6	0	1.847910	-1.924159	1.267983
82	6	0	0.560779	-3.943075	1.756983
83	1	0	-0.342259	-4.529017	1.627983
84	6	0	2.842879	-2.395223	2.151983
85	1	0	3.716919	-1.786280	2.343983
86	6	0	1.554750	-4.387140	2.611983
87	1	0	1.432689	-5.326132	3.142983

88	6	0	2.702801	-3.606214	2.809983
89	1	0	3.475779	-3.937264	3.495983
90	6	0	-2.060293	-5.049905	-1.550017
91	6	0	4.464003	-0.495328	0.381983
92	6	0	4.769927	-1.663348	-0.337017
93	6	0	5.505043	0.131604	1.076983
94	6	0	6.060894	-2.175432	-0.348017
95	1	0	3.988894	-2.163298	-0.902017
96	6	0	6.799009	-0.394480	1.067983
97	1	0	5.301102	1.033617	1.646983
98	6	0	7.111934	-1.559500	0.355983
99	1	0	6.254835	-3.074445	-0.925017
100	1	0	7.567043	0.123470	1.629983
101	6	0	8.525894	-2.164592	0.315983
102	6	0	9.025892	-2.202624	-1.149017
103	1	0	10.031864	-2.633689	-1.195017
104	1	0	8.375853	-2.809582	-1.787017
105	1	0	9.067957	-1.195627	-1.576017
106	6	0	9.533947	-1.348657	1.146983
107	1	0	10.521917	-1.815721	1.087983
108	1	0	9.634014	-0.323664	0.776983
109	1	0	9.253950	-1.307639	2.203983
110	6	0	8.484801	-3.604589	0.879983
111	1	0	8.143801	-3.608567	1.920983
112	1	0	7.814759	-4.249546	0.304983
113	1	0	9.483772	-4.052654	0.848983
114	6	0	0.302735	-4.624059	-1.202017
115	1	0	1.102778	-3.958111	-0.916017
116	6	0	0.627656	-5.847080	-1.757017
117	1	0	1.670638	-6.123147	-1.882017
118	6	0	-0.390401	-6.716014	-2.173017
119	1	0	-0.148463	-7.685029	-2.598017
120	6	0	-1.708374	-6.307928	-2.089017
121	1	0	-2.472417	-6.962879	-2.486017

Transition structure of **V**, for the blue ring closure (C_7 H^+)

Standard orientation:

Center Number	Atomic Number	Atomic Type	Coordinates (Angstroms) X	Y	Z
1	6	0	-1.888186	4.186904	0.657019
2	6	0	-1.796066	1.331908	0.321019
3	6	0	-3.014154	3.420857	0.844019
4	6	0	-0.656159	3.538955	0.415019
5	6	0	-0.613099	2.122957	0.238019
6	6	0	-2.961095	2.009859	0.753019
7	6	0	0.678926	1.510011	0.080019
8	6	0	3.219977	0.290117	0.261019
9	6	0	0.787983	0.143016	0.435019
10	6	0	1.823897	2.215059	-0.341981
11	6	0	3.056923	1.583110	-0.274981
12	6	0	2.073007	-0.430931	0.665019
13	1	0	3.936902	2.078147	-0.675981
14	6	0	1.748839	3.607056	-0.974981
15	6	0	0.945722	6.399022	-1.112981
16	6	0	2.413794	4.681083	-0.162981

17	6	0	0.394820	4.041999	-1.458981
18	6	0	0.149763	5.419989	-1.676981
19	6	0	2.039740	5.978068	-0.258981
20	1	0	3.256806	4.392119	0.457019
21	1	0	2.586708	6.730091	0.300019
22	1	0	-0.150150	3.327976	-2.065981
23	1	0	0.225821	4.030992	0.820019
24	6	0	-0.408983	-0.676034	0.397019
25	6	0	-2.365912	-2.369116	-0.662981
26	6	0	-1.702005	-0.131088	0.140019
27	6	0	-0.197924	-2.076026	0.417019
28	6	0	-1.080890	-2.893062	-0.324981
29	6	0	-2.741968	-1.024132	-0.297981
30	6	0	-4.140062	1.229810	1.163019
31	6	0	-4.578065	1.293792	2.434019
32	1	0	-4.009093	1.965815	3.079019
33	6	0	-4.744033	0.528785	0.010019
34	6	0	-6.121977	-0.815273	-1.980981
35	6	0	-5.945054	1.036735	-0.483981
36	6	0	-4.122986	-0.605189	-0.579981
37	6	0	-4.928954	-1.368223	-1.471981
38	6	0	-6.610027	0.396707	-1.527981
39	1	0	-6.360091	1.931717	-0.031981
40	1	0	-7.523045	0.811669	-1.940981
41	1	0	-6.672955	-1.347296	-2.745981
42	6	0	-5.691035	0.579745	3.186019
43	6	0	-6.838077	1.592697	3.422019
44	1	0	-7.304090	1.890678	2.478019
45	1	0	-6.478115	2.497712	3.924019
46	1	0	-7.612059	1.146665	4.056019
47	6	0	-5.107018	0.164770	4.558019
48	1	0	-4.723054	1.029786	5.110019
49	1	0	-4.286988	-0.551196	4.439019
50	1	0	-5.880998	-0.307263	5.172019
51	6	0	-6.243982	-0.679278	2.494019
52	1	0	-5.443953	-1.389245	2.260019
53	1	0	-6.773992	-0.449300	1.568019
54	1	0	-6.948962	-1.180307	3.165019
55	6	0	-3.304875	-3.244155	-1.343981
56	6	0	-5.203804	-4.964235	-2.526981
57	6	0	-4.567896	-2.748208	-1.765981
58	6	0	-2.983818	-4.613142	-1.597981
59	6	0	-3.958783	-5.452183	-2.157981
60	6	0	-5.496860	-3.620247	-2.351981
61	1	0	-3.746739	-6.500174	-2.318981
62	1	0	-6.481874	-3.266288	-2.625981
63	1	0	-5.945776	-5.633265	-2.950981
64	6	0	0.712660	7.893012	-1.329981
65	6	0	0.404632	8.558000	0.037019
66	1	0	1.223637	8.445034	0.753019
67	1	0	0.242587	9.629993	-0.108981
68	1	0	-0.501351	8.139962	0.487019
69	6	0	2.000634	8.517066	-1.927981
70	1	0	1.850589	9.591060	-2.073981
71	1	0	2.867639	8.390102	-1.273981
72	1	0	2.239652	8.074076	-2.899981
73	6	0	-0.459352	8.174964	-2.287981

74	1	0	-0.295334	7.733970	-3.275981
75	1	0	-1.411337	7.804924	-1.894981
76	1	0	-0.563397	9.254959	-2.425981
77	6	0	2.078061	-1.723931	1.360019
78	6	0	0.931095	-2.543978	1.228019
79	6	0	3.104078	-2.121888	2.241019
80	6	0	0.838145	-3.727982	1.988019
81	6	0	2.998127	-3.294892	2.972019
82	1	0	3.971051	-1.487852	2.374019
83	6	0	1.857160	-4.100940	2.847019
84	1	0	-0.055830	-4.336020	1.908019
85	1	0	3.791138	-3.573859	3.659019
86	1	0	1.762198	-5.006944	3.438019
87	6	0	-1.620798	-5.090085	-1.382981
88	6	0	-1.204745	-6.369068	-1.817981
89	6	0	-0.657835	-4.203045	-0.844981
90	6	0	0.125271	-6.743012	-1.799981
91	1	0	-1.928716	-7.070098	-2.211981
92	6	0	0.705180	-4.574988	-0.927981
93	6	0	1.095232	-5.818972	-1.387981
94	1	0	0.416312	-7.730000	-2.145981
95	1	0	1.471151	-3.870956	-0.639981
96	1	0	2.149243	-6.069928	-1.438981
97	6	0	4.598001	-0.270825	0.247019
98	6	0	4.891051	-1.479813	-0.397981
99	6	0	5.666971	0.441219	0.815019
100	6	0	6.965991	-0.051726	0.758019
101	6	0	7.269042	-1.265714	0.117019
102	6	0	6.198071	-1.960759	-0.462981
103	6	0	8.720063	-1.774653	0.076019
104	6	0	9.612020	-0.728616	-0.633981
105	1	0	9.277012	-0.557630	-1.661981
106	1	0	10.650034	-1.077573	-0.668981
107	1	0	9.605979	0.235384	-0.114981
108	6	0	8.849119	-3.109648	-0.680981
109	1	0	8.531115	-3.020661	-1.724981
110	1	0	8.264152	-3.905672	-0.209981
111	1	0	9.895132	-3.430604	-0.683981
112	6	0	9.228072	-1.985632	1.522019
113	1	0	9.205033	-1.058633	2.103019
114	1	0	10.262087	-2.345589	1.511019
115	1	0	8.617103	-2.726658	2.049019
116	1	0	4.091075	-2.045846	-0.865981
117	1	0	6.374110	-2.895751	-0.981981
118	1	0	5.472932	1.380211	1.328019
119	1	0	7.758967	0.524307	1.226019
120	1	0	-3.957173	3.878818	1.122019
121	1	0	-1.920231	5.263903	0.788019
122	1	0	-0.721248	5.690953	-2.260981
123	1	0	2.346841	3.544081	-1.910981

Transition structure of **V**, for the red ring closure ($C_8 H^+$)

Standard orientation:

Center Number	Atomic Number	Atomic Type	Coordinates (Angstroms) X	Y	Z

1	6	0	-1.696381	4.125817	1.758959
2	6	0	-1.837150	1.442805	0.865959
3	6	0	-2.780312	3.324724	2.109959
4	6	0	-0.637333	3.566908	1.061959
5	6	0	-0.668218	2.229906	0.621959
6	6	0	-2.827197	1.987720	1.712959
7	6	0	0.560839	1.573012	0.158959
8	6	0	3.023961	0.150224	0.028959
9	6	0	0.585960	0.159014	0.272959
10	6	0	1.748782	2.226114	-0.271041
11	6	0	2.950845	1.494217	-0.309041
12	6	0	1.826020	-0.537880	0.298959
13	6	0	1.797663	3.614118	-0.800041
14	6	0	1.980438	6.225134	-1.935041
15	6	0	2.806586	4.508205	-0.406041
16	6	0	0.889626	4.046040	-1.778041
17	6	0	0.983516	5.321048	-2.331041
18	6	0	2.887476	5.783212	-0.956041
19	6	0	-0.660976	-0.579094	0.202959
20	6	0	-2.908868	-1.831287	-0.887041
21	6	0	-1.918032	0.067798	0.332959
22	6	0	-0.570859	-1.934086	-0.202041
23	6	0	-1.631814	-2.462177	-0.973041
24	6	0	-3.097971	-0.635303	-0.099041
25	6	0	-3.935123	1.129625	2.177959
26	6	0	-4.200087	0.713602	3.428959
27	6	0	-4.860094	0.789545	1.071959
28	6	0	-6.762028	0.026381	-0.799041
29	6	0	-6.129143	1.361436	1.079959
30	6	0	-4.473014	-0.134422	0.059959
31	6	0	-5.508973	-0.619511	-0.788041
32	6	0	-7.066114	1.027355	0.102959
33	6	0	-3.462099	0.848665	4.754959
34	6	0	-1.959126	1.159795	4.630959
35	6	0	-3.613982	-0.506348	5.487959
36	6	0	-4.156193	1.945606	5.597959
37	6	0	-4.023815	-2.453383	-1.579041
38	6	0	-6.235705	-3.727573	-2.781041
39	6	0	-5.307867	-1.846494	-1.552041
40	6	0	-3.859709	-3.687369	-2.283041
41	6	0	-4.980655	-4.313465	-2.845041
42	6	0	-6.393811	-2.499587	-2.158041
43	6	0	2.110317	7.640145	-2.525041
44	6	0	2.002227	8.683136	-1.387041
45	6	0	3.486305	7.778263	-3.219041
46	6	0	1.012290	7.944050	-3.562041
47	6	0	1.793142	-1.947882	0.558959
48	6	0	0.616204	-2.669984	0.237959
49	6	0	2.945200	-2.626783	1.076959
50	6	0	0.607323	-4.052984	0.488959
51	6	0	2.895322	-4.037788	1.258959
52	6	0	1.738381	-4.732887	0.965959
53	6	0	-2.517663	-4.217253	-2.509041
54	6	0	-2.278570	-5.300233	-3.386041
55	6	0	-1.411721	-3.547158	-1.937041
56	6	0	-0.998538	-5.667123	-3.755041
57	6	0	-0.117692	-3.875047	-2.413041

58	6	0	0.093397	-4.917029	-3.296041
59	1	0	-1.648470	5.160821	2.082959
60	1	0	-3.582346	3.717655	2.724959
61	1	0	3.842802	2.004294	-0.662041
62	1	0	3.518612	4.212266	0.359959
63	1	0	3.674419	6.446280	-0.611041
64	1	0	0.105684	3.373972	-2.115041
65	1	0	0.248616	4.162985	0.896959
66	1	0	-6.374204	2.074415	1.860959
67	1	0	-8.038156	1.511271	0.079959
68	1	0	-7.513003	-0.268683	-1.520041
69	1	0	-1.769212	2.157811	4.232959
70	1	0	-1.458063	0.434838	3.981959
71	1	0	-1.493121	1.103835	5.620959
72	1	0	-3.140913	-1.316307	4.922959
73	1	0	-4.667960	-0.767438	5.629959
74	1	0	-3.143986	-0.462307	6.475959
75	1	0	-5.227176	1.745513	5.708959
76	1	0	-4.043278	2.931615	5.136959
77	1	0	-3.718197	1.989643	6.601959
78	1	0	-4.878573	-5.266457	-3.348041
79	1	0	-7.386847	-2.074672	-2.103041
80	1	0	-7.093662	-4.229647	-3.218041
81	1	0	2.787238	8.557203	-0.637041
82	1	0	2.094140	9.696144	-1.795041
83	1	0	1.036233	8.611052	-0.878041
84	1	0	3.599218	8.785273	-3.636041
85	1	0	4.314319	7.613335	-2.523041
86	1	0	3.587367	7.059272	-4.039041
87	1	0	1.055349	7.260054	-4.416041
88	1	0	0.010295	7.886964	-3.125041
89	1	0	1.144203	8.958062	-3.949041
90	1	0	3.574153	-2.074729	1.777959
91	1	0	-3.111523	-5.840305	-3.819041
92	1	0	-0.848467	-6.498110	-4.438041
93	1	0	0.728256	-3.275974	-2.107041
94	1	0	1.096416	-5.136942	-3.647041
95	1	0	0.262492	5.603986	-3.089041
96	6	0	4.373022	-0.558660	-0.018041
97	6	0	5.232999	-0.285586	1.182959
98	6	0	6.224072	-1.132501	1.552959
99	6	0	6.477178	-2.366479	0.834959
100	6	0	5.531214	-2.784561	-0.079041
101	6	0	4.344149	-2.034663	-0.338041
102	1	0	3.770173	-2.311712	-1.217041
103	6	0	7.734250	-3.208371	1.096959
104	6	0	8.714191	-2.519287	2.067959
105	1	0	9.035107	-1.540259	1.699959
106	1	0	9.609244	-3.138210	2.175959
107	1	0	8.288180	-2.393323	3.067959
108	6	0	8.470271	-3.453308	-0.243041
109	1	0	8.784190	-2.510281	-0.701041
110	1	0	7.850318	-3.991361	-0.965041
111	1	0	9.366323	-4.056231	-0.064041
112	6	0	7.308367	-4.567408	1.707959
113	1	0	6.659416	-5.133464	1.032959
114	1	0	6.781355	-4.429453	2.657959

115	1	0	8.195420	-5.177331	1.903959
116	1	0	-5.123038	0.139522	3.524959
117	1	0	3.757366	-4.550713	1.673959
118	1	0	1.682474	-5.802892	1.135959
119	1	0	-0.302629	-4.613063	0.303959
120	1	0	6.862049	-0.865446	2.385959
121	1	0	5.085918	0.656401	1.703959
122	1	0	5.641296	-3.741551	-0.577041
123	1	0	4.925985	-0.123613	-0.877041

Transition structure of **VI**, for the pink ring closure (C_3 H^+)

Standard orientation:

Center Number	Atomic Number	Atomic Type	Coordinates (Angstroms) X	Y	Z
1	6	0	-2.180148	3.824999	1.094015
2	6	0	-2.056054	1.041003	0.407015
3	6	0	-3.284119	2.972961	1.356015
4	6	0	-1.027130	3.290038	0.594015
5	6	0	-0.906083	1.898042	0.290015
6	6	0	-3.218073	1.608964	0.953015
7	6	0	0.411936	1.331086	0.098015
8	6	0	2.977974	0.194173	0.529015
9	6	0	0.548982	-0.032909	0.488015
10	6	0	1.580911	2.066126	-0.262985
11	6	0	2.818931	1.472167	-0.035985
12	6	0	1.824000	-0.568866	0.822015
13	1	0	3.707914	1.991197	-0.373985
14	6	0	1.555868	3.356125	-0.998985
15	6	0	1.604786	5.787126	-2.494985
16	6	0	2.406832	4.415153	-0.637985
17	6	0	0.738862	3.533097	-2.125985
18	6	0	0.766822	4.721098	-2.854985
19	6	0	2.421792	5.599154	-1.365985
20	1	0	3.048835	4.313175	0.233015
21	1	0	0.126819	4.802076	-3.725985
22	1	0	3.087765	6.394176	-1.045985
23	1	0	0.090889	2.724075	-2.449985
24	6	0	-0.608989	-0.899948	0.381015
25	6	0	-2.474931	-2.611011	-0.794985
26	6	0	-1.915006	-0.400992	0.127015
27	6	0	-0.352942	-2.290940	0.364015
28	6	0	-1.177914	-3.099968	-0.452985
29	6	0	-2.914975	-1.310026	-0.349985
30	6	0	-4.378046	0.786924	1.299015
31	6	0	-4.939047	0.827905	2.519015
32	6	0	-5.002021	0.062903	0.171015
33	6	0	-6.419976	-1.274144	-1.792985
34	6	0	-6.308034	0.440859	-0.139985
35	6	0	-4.329987	-0.962074	-0.550985
36	6	0	-5.123961	-1.719101	-1.461985
37	6	0	-7.006013	-0.194164	-1.163985
38	1	0	-6.768061	1.247844	0.421015
39	1	0	-8.006023	0.127802	-1.434985
40	1	0	-6.990959	-1.795164	-2.548985
41	6	0	-3.350902	-3.463041	-1.578985

42	6	0	-5.065847	-5.107099	-3.099985
43	6	0	-4.637918	-3.000084	-1.967985
44	6	0	-2.933858	-4.769027	-1.982985
45	6	0	-3.816831	-5.571057	-2.719985
46	6	0	-5.466890	-3.831112	-2.736985
47	1	0	-3.524797	-6.568047	-3.020985
48	1	0	-6.454901	-3.505146	-3.032985
49	1	0	-5.728825	-5.741121	-3.678985
50	6	0	1.662741	7.110128	-3.276985
51	6	0	1.290701	8.279116	-2.333985
52	1	0	1.974699	8.353139	-1.482985
53	1	0	1.333669	9.230117	-2.874985
54	1	0	0.275705	8.160081	-1.939985
55	6	0	3.096734	7.323177	-3.816985
56	1	0	3.156702	8.267179	-4.367985
57	1	0	3.836732	7.361202	-3.011985
58	1	0	3.383761	6.515186	-4.497985
59	6	0	0.689740	7.127095	-4.470985
60	1	0	0.921767	6.342103	-5.197985
61	1	0	-0.351256	7.006060	-4.152985
62	1	0	0.764708	8.087098	-4.990985
63	6	0	1.821044	-1.875866	1.491015
64	6	0	0.738074	-2.748903	1.227015
65	6	0	2.782057	-2.253834	2.451015
66	6	0	0.648115	-3.971906	1.925015
67	6	0	2.680098	-3.462837	3.122015
68	1	0	3.595034	-1.579806	2.688015
69	6	0	1.608127	-4.327874	2.857015
70	1	0	-0.197863	-4.623934	1.740015
71	1	0	3.422107	-3.727812	3.869015
72	1	0	1.515159	-5.264877	3.397015
73	6	0	-1.568843	-5.215981	-1.719985
74	6	0	-1.078802	-6.442964	-2.222985
75	6	0	-0.677872	-4.351951	-1.037985
76	6	0	0.258210	-6.780919	-2.136985
77	1	0	-1.748778	-7.134987	-2.715985
78	6	0	0.700139	-4.679904	-1.047985
79	6	0	1.163179	-5.868889	-1.577985
80	1	0	0.605242	-7.727907	-2.536985
81	1	0	1.419115	-3.982880	-0.643985
82	1	0	2.227186	-6.085853	-1.566985
83	6	0	4.371992	-0.314780	0.640015
84	6	0	5.361966	0.447253	1.273015
85	6	0	6.685981	0.008298	1.327015
86	6	0	7.082021	-1.196689	0.733015
87	6	0	6.083047	-1.946723	0.085015
88	6	0	4.761032	-1.524767	0.040015
89	1	0	5.092934	1.388244	1.747015
90	1	0	7.411960	0.627322	1.841015
91	1	0	6.343078	-2.881714	-0.401985
92	1	0	4.023053	-2.127792	-0.477985
93	6	0	8.533039	-1.709640	0.762015
94	6	0	9.477006	-0.750608	1.512015
95	1	0	9.516973	0.236393	1.040015
96	1	0	10.492020	-1.157574	1.507015
97	1	0	9.181002	-0.620618	2.558015
98	6	0	9.051044	-1.863622	-0.687985

99	1	0	9.036011	-0.903623	-1.214985
100	1	0	8.449068	-2.571643	-1.265985
101	1	0	10.082056	-2.232588	-0.682985
102	6	0	8.575085	-3.084638	1.471015
103	1	0	7.955110	-3.827659	0.961015
104	1	0	8.222082	-3.004650	2.504015
105	1	0	9.601098	-3.466604	1.493015
106	6	0	-4.488077	1.706921	3.671015
107	6	0	-5.607108	2.619883	4.101015
108	6	0	-3.173101	2.421965	3.480015
109	6	0	-2.965143	3.659972	4.154015
110	6	0	-4.022166	4.354936	4.681015
111	6	0	-5.372149	3.860891	4.584015
112	1	0	-6.616094	2.221849	4.058015
113	1	0	-5.818026	0.218876	2.715015
114	1	0	-2.239184	4.881997	1.334015
115	1	0	-0.161152	3.923067	0.472015
116	1	0	-4.267134	3.404928	1.523015
117	1	0	-2.308079	1.787994	3.321015
118	1	0	-1.960156	4.066006	4.206015
119	1	0	-3.847198	5.302942	5.181015
120	1	0	-6.189170	4.484863	4.930015
121	1	0	-4.302054	1.040927	4.543015

Transition structure of **VI**, for the blue ring closure (C_{12} H^+)

Standard orientation:

Center Number	Atomic Number	Atomic Type	Coordinates (Angstroms) X	Y	Z
1	6	0	-3.021143	3.299856	0.094002
2	6	0	-2.102032	0.576893	0.018002
3	6	0	-3.884101	2.261821	0.339002
4	6	0	-1.644131	3.018912	-0.062998
5	6	0	-1.191076	1.666930	-0.110998
6	6	0	-3.426046	0.920839	0.376002
7	6	0	0.226933	1.439988	-0.190998
8	6	0	2.998950	1.019101	0.150002
9	6	0	0.702984	0.196007	0.287002
10	6	0	1.142893	2.408025	-0.652998
11	6	0	2.499903	2.163081	-0.505998
12	6	0	2.086990	0.035064	0.595002
13	1	0	3.219875	2.854110	-0.933998
14	6	0	0.701842	3.663007	-1.412998
15	6	0	-0.862256	6.081944	-1.816998
16	6	0	0.994790	4.946019	-0.688998
17	6	0	-0.697157	3.645950	-1.957998
18	6	0	-1.318207	4.870925	-2.303998
19	6	0	0.268744	6.066990	-0.908998
20	1	0	1.857789	4.965054	-0.030998
21	1	0	-2.205205	4.830889	-2.924998
22	1	0	0.552707	6.987001	-0.409998
23	6	0	-0.206970	-0.934030	0.293002
24	6	0	-1.545878	-3.193084	-0.667998
25	6	0	-1.586976	-0.806086	-0.043998
26	6	0	0.393081	-2.207005	0.438002
27	6	0	-0.181874	-3.296029	-0.252998

28	6	0	-2.307928	-1.987115	-0.437998
29	6	0	-4.385004	-0.117200	0.763002
30	6	0	-5.074001	-0.175228	1.924002
31	6	0	-4.686966	-1.049212	-0.346998
32	6	0	-5.527889	-2.928246	-2.196998
33	6	0	-5.971968	-0.985264	-0.884998
34	6	0	-3.735927	-2.005173	-0.799998
35	6	0	-4.247884	-3.051194	-1.619998
36	6	0	-6.376932	-1.890281	-1.864998
37	1	0	-6.651000	-0.219292	-0.525998
38	1	0	-7.357935	-1.811321	-2.320998
39	1	0	-5.861859	-3.666260	-2.915998
40	6	0	-2.159831	-4.356109	-1.284998
41	6	0	-3.432738	-6.652161	-2.319998
42	6	0	-3.491834	-4.288163	-1.769998
43	6	0	-1.447781	-5.590080	-1.404998
44	6	0	-2.114735	-6.721107	-1.892998
45	6	0	-4.107787	-5.442189	-2.279998
46	1	0	-1.602696	-7.673087	-1.948998
47	1	0	-5.141788	-5.416231	-2.595998
48	1	0	-3.933701	-7.543181	-2.685998
49	6	0	-1.502311	7.421918	-2.174998
50	6	0	-2.044338	8.083896	-0.879998
51	1	0	-1.258345	8.268928	-0.142998
52	1	0	-2.495377	9.048877	-1.128998
53	1	0	-2.814312	7.463864	-0.409998
54	6	0	-0.421348	8.336962	-2.806998
55	1	0	-0.863387	9.305944	-3.055998
56	1	0	0.416645	8.519996	-2.128998
57	1	0	-0.023330	7.899978	-3.727998
58	6	0	-2.664305	7.273870	-3.173998
59	1	0	-2.339286	6.818884	-4.114998
60	1	0	-3.486280	6.676837	-2.764998
61	1	0	-3.067345	8.261854	-3.410998
62	6	0	2.426038	-1.142922	1.402002
63	6	0	1.570084	-2.266957	1.313002
64	6	0	3.479039	-1.157880	2.339002
65	6	0	1.783129	-3.365949	2.170002
66	6	0	3.678083	-2.252871	3.166002
67	1	0	4.120003	-0.291853	2.438002
68	6	0	2.824128	-3.361906	3.083002
69	1	0	1.106163	-4.210976	2.122002
70	1	0	4.483083	-2.237839	3.894002
71	1	0	2.963163	-4.208901	3.747002
72	6	0	-0.014779	-5.628022	-1.127998
73	6	0	0.773267	-6.756990	-1.448998
74	6	0	0.624173	-4.460996	-0.648998
75	6	0	2.152265	-6.722934	-1.373998
76	1	0	0.302304	-7.666009	-1.799998
77	6	0	2.039172	-4.422938	-0.671998
78	6	0	2.793217	-5.527907	-1.018998
79	1	0	2.732301	-7.602910	-1.631998
80	1	0	2.554134	-3.504917	-0.427998
81	1	0	3.876214	-5.461863	-1.025998
82	6	0	4.478956	0.877161	0.216002
83	6	0	5.274914	1.912194	0.723002
84	6	0	6.666918	1.810250	0.740002

85	6	0	7.324964	0.681277	0.235002
86	6	0	6.517006	-0.344756	-0.287998
87	6	0	5.131002	-0.256812	-0.296998
88	1	0	4.800877	2.800174	1.134002
89	1	0	7.236884	2.632273	1.158002
90	1	0	6.979042	-1.235737	-0.702998
91	1	0	4.545035	-1.067836	-0.717998
92	6	0	8.855970	0.533339	0.236002
93	6	0	9.561920	1.758368	0.846002
94	1	0	9.348883	2.674359	0.286002
95	1	0	10.644926	1.605412	0.824002
96	1	0	9.274913	1.917356	1.891002
97	6	0	9.354977	0.360360	-1.218998
98	1	0	9.096941	1.232349	-1.828998
99	1	0	8.925013	-0.523658	-1.697998
100	1	0	10.443981	0.248404	-1.233998
101	6	0	9.247021	-0.714645	1.063002
102	1	0	8.810058	-1.630662	0.655002
103	1	0	8.914017	-0.617658	2.102002
104	1	0	10.335026	-0.839600	1.067002
105	6	0	-4.954035	0.645777	3.137002
106	6	0	-6.082040	0.763731	3.971002
107	6	0	-3.763061	1.281826	3.536002
108	6	0	-3.717091	2.032827	4.709002
109	6	0	-4.854097	2.163781	5.509002
110	6	0	-6.038071	1.522733	5.137002
111	1	0	-7.003019	0.257694	3.692002
112	1	0	-4.928108	2.449778	0.565002
113	1	0	-2.863056	1.158862	2.945002
114	1	0	-2.786110	2.504865	5.008002
115	1	0	-4.814120	2.746783	6.424002
116	1	0	-6.923074	1.606697	5.761002
117	1	0	-5.825969	-0.960258	1.983002
118	1	0	-3.366185	4.328842	0.119002
119	1	0	-0.958162	3.775940	0.309002
120	1	0	-0.989121	2.757938	-2.507998
121	1	0	1.333841	3.697033	-2.326998

Transition structure of **VI**, for the red ring closure (C_{13} H^+)

Standard orientation:

Center Number	Atomic Number	Atomic Type	Coordinates (Angstroms) X	Y	Z
1	6	0	-1.843814	4.090098	1.329994
2	6	0	-1.889959	1.365101	0.552994
3	6	0	-2.916857	3.279155	1.681994
4	6	0	-0.752844	3.531040	0.684994
5	6	0	-0.734916	2.174039	0.307994
6	6	0	-2.923929	1.928156	1.331994
7	6	0	0.529050	1.535972	-0.081006
8	6	0	3.039979	0.195838	-0.054006
9	6	0	0.595975	0.132968	0.116994
10	6	0	1.707086	2.204909	-0.510006
11	6	0	2.934049	1.514844	-0.471006
12	6	0	1.857941	-0.516099	0.222994
13	1	0	3.819077	2.035797	-0.826006

14	6	0	1.728158	3.564908	-1.110006
15	6	0	1.861294	6.122901	-2.366006
16	6	0	2.686209	4.516857	-0.720006
17	6	0	0.847177	3.911955	-2.144006
18	6	0	0.918243	5.159951	-2.758006
19	6	0	2.740275	5.765854	-1.329006
20	6	0	-0.623067	-0.650967	0.054994
21	6	0	-2.787141	-2.054852	-1.021006
22	6	0	-1.906034	-0.042898	0.100994
23	6	0	-0.473140	-2.024975	-0.258006
24	6	0	-1.486172	-2.639921	-1.027006
25	6	0	-3.045075	-0.817838	-0.319006
26	6	0	-4.032974	1.079215	1.803994
27	6	0	-4.361988	0.825232	3.087994
28	6	0	-4.901000	0.607261	0.703994
29	6	0	-6.713051	-0.360642	-1.160006
30	6	0	-6.194972	1.118330	0.641994
31	6	0	-4.443051	-0.363763	-0.232006
32	6	0	-5.434082	-0.948711	-1.073006
33	6	0	-7.087996	0.680378	-0.335006
34	1	0	-6.490932	1.872346	1.362994
35	1	0	-8.076972	1.118430	-0.417006
36	1	0	-7.427071	-0.734604	-1.881006
37	6	0	-3.855179	-2.765795	-1.701006
38	6	0	-5.980256	-4.209681	-2.870006
39	6	0	-5.163150	-2.213725	-1.747006
40	6	0	-3.621247	-4.035807	-2.316006
41	6	0	-4.700285	-4.744750	-2.862006
42	6	0	-6.203189	-2.949670	-2.336006
43	1	0	-4.545337	-5.724758	-3.294006
44	1	0	-7.214168	-2.564616	-2.334006
45	1	0	-6.804286	-4.774638	-3.292006
46	6	0	1.958368	7.513896	-3.015006
47	6	0	1.714426	8.596909	-1.937006
48	1	0	2.448423	8.539870	-1.128006
49	1	0	1.786479	9.595905	-2.381006
50	1	0	0.717421	8.494962	-1.495006
51	6	0	3.370378	7.701821	-3.620006
52	1	0	3.453431	8.689816	-4.084006
53	1	0	4.154374	7.625779	-2.861006
54	1	0	3.572338	6.948810	-4.389006
55	6	0	0.922379	7.708951	-4.139006
56	1	0	1.060340	6.987944	-4.951006
57	1	0	-0.104626	7.619006	-3.769006
58	1	0	1.030432	8.709945	-4.567006
59	6	0	1.864867	-1.908099	0.569994
60	6	0	0.723825	-2.689039	0.262994
61	6	0	3.023834	-2.512161	1.160994
62	6	0	0.752752	-4.054040	0.599994
63	6	0	3.015760	-3.909161	1.429994
64	1	0	3.613867	-1.897192	1.842994
65	6	0	1.891720	-4.662101	1.149994
66	1	0	-0.132280	-4.654993	0.426994
67	1	0	3.881736	-4.365207	1.899994
68	1	0	1.866664	-5.720099	1.386994
69	6	0	-2.254273	-4.520880	-2.474006
70	6	0	-1.946332	-5.642896	-3.278006

71	6	0	-1.193233	-3.771936	-1.914006
72	6	0	-0.641350	-5.977966	-3.588006
73	1	0	-2.743364	-6.239854	-3.703006
74	6	0	0.126751	-4.074007	-2.332006
75	6	0	0.405693	-5.158022	-3.143006
76	1	0	-0.437396	-6.838977	-4.216006
77	1	0	0.938786	-3.424050	-2.038006
78	1	0	1.426683	-5.357076	-3.452006
79	6	0	4.412943	-0.467235	-0.022006
80	6	0	5.231963	-0.093279	1.179994
81	6	0	6.239921	-0.881332	1.623994
82	6	0	6.552854	-2.146349	0.987994
83	6	0	5.643827	-2.652301	0.080994
84	6	0	4.440864	-1.960237	-0.252006
85	1	0	5.040015	0.871732	1.638994
86	1	0	6.847939	-0.544365	2.454994
87	1	0	5.798775	-3.633309	-0.355006
88	1	0	3.900845	-2.309208	-1.126006
89	6	0	7.833812	-2.923417	1.326994
90	6	0	8.756854	-2.151466	2.289994
91	1	0	9.050905	-1.177482	1.884994
92	1	0	9.670823	-2.727515	2.451994
93	1	0	8.296862	-1.994442	3.270994
94	6	0	8.616798	-3.200459	0.018994
95	1	0	8.906847	-2.267474	-0.474006
96	1	0	8.036766	-3.793428	-0.693006
97	1	0	9.528768	-3.759508	0.248994
98	6	0	7.444741	-4.269397	1.990994
99	1	0	6.843707	-4.896365	1.324994
100	1	0	6.880749	-4.109366	2.915994
101	1	0	8.350711	-4.829445	2.244994
102	6	0	-3.683969	1.183196	4.344994
103	6	0	-4.452968	1.191237	5.523994
104	6	0	-2.309953	1.471123	4.452994
105	6	0	-1.741937	1.779093	5.685994
106	6	0	-2.525936	1.807135	6.841994
107	6	0	-3.885952	1.508207	6.755994
108	1	0	-5.511981	0.951294	5.465994
109	1	0	-3.741836	3.672199	2.264994
110	1	0	-1.681955	1.439090	3.569994
111	1	0	-0.678926	1.995036	5.746994
112	1	0	-2.077923	2.049111	7.800994
113	1	0	-4.504951	1.516240	7.648994
114	1	0	-5.279018	0.254281	3.230994
115	1	0	-1.829758	5.138097	1.612994
116	1	0	0.122189	4.141993	0.516994
117	1	0	0.104138	3.193994	-2.478006
118	1	0	0.222255	5.376988	-3.560006
119	1	0	3.375196	4.285820	0.087994
120	1	0	3.484313	6.476814	-0.984006
121	1	0	4.973965	-0.067265	-0.893006

7. Copies of ^{1}H and ^{13}C-NMR of new compounds

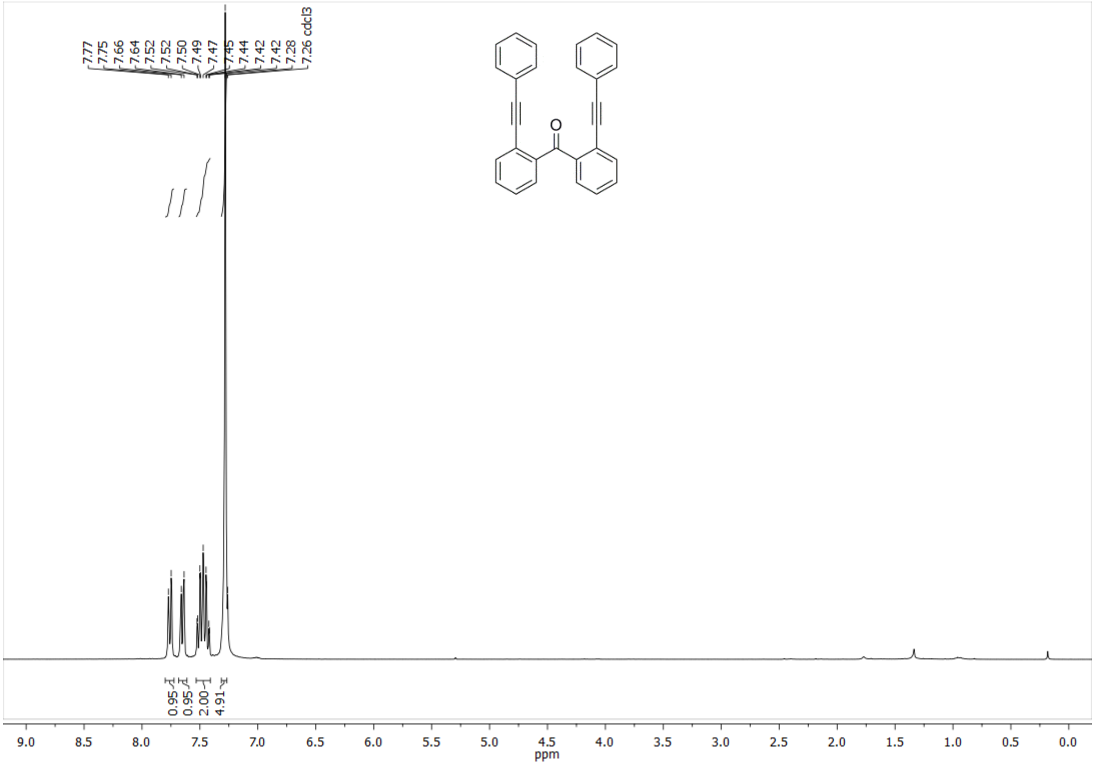

Figure S10: ^{1}H NMR spectrum of **3a** in $CDCl_3$ (300 MHz).

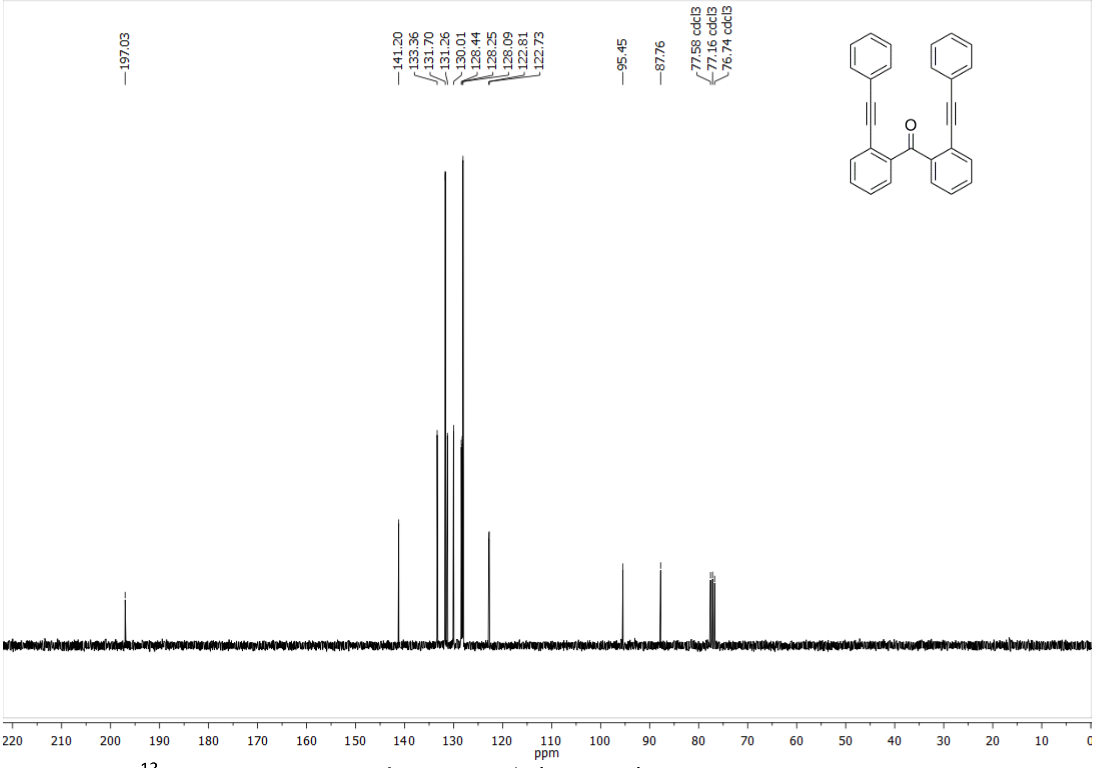

Figure S11: ^{13}C NMR spectrum of **3a** in $CDCl_3$ (75 MHz).

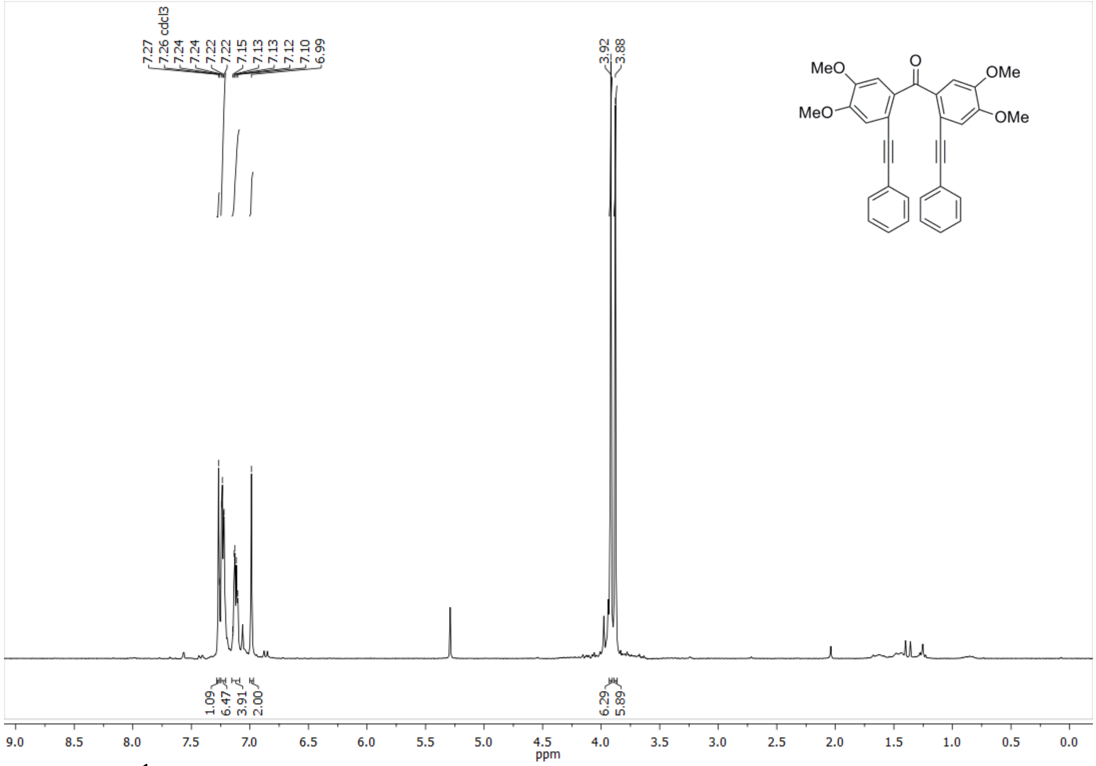

Figure S12: ^{1}H NMR spectrum of **3b** in $CDCl_3$ (300 MHz).

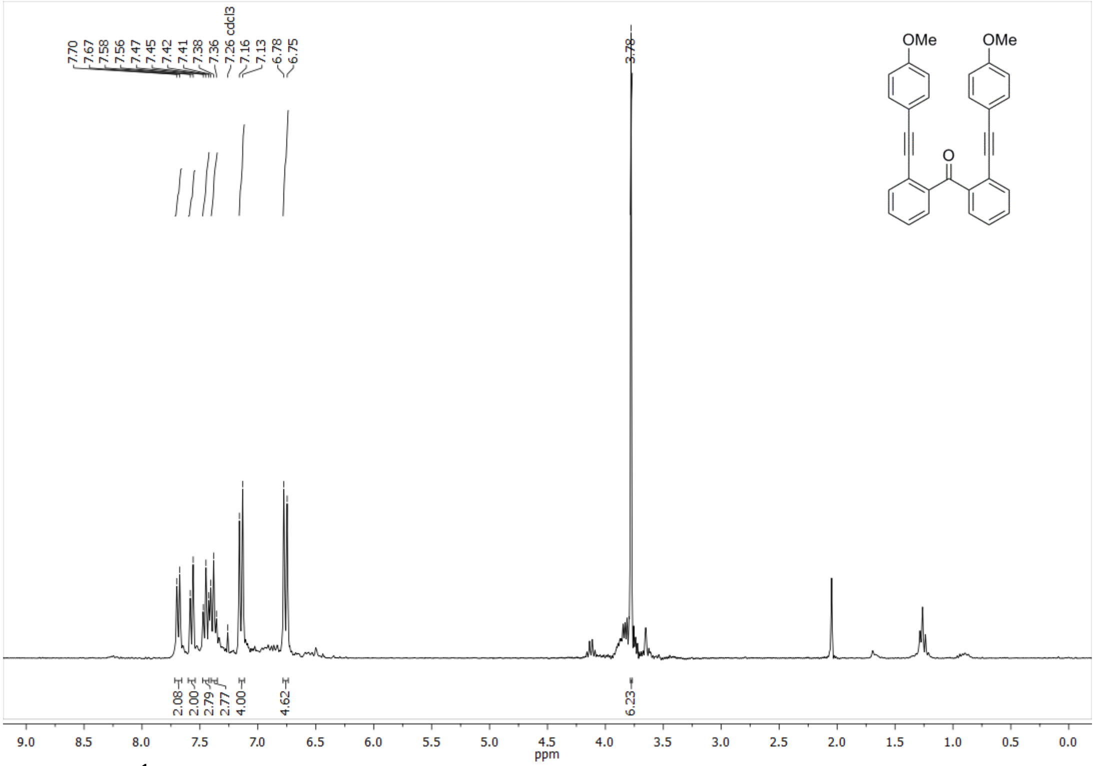

Figure S13: ^{1}H NMR spectrum of **3c** in $CDCl_3$ (300 MHz) with residual ethyl acetate peak.

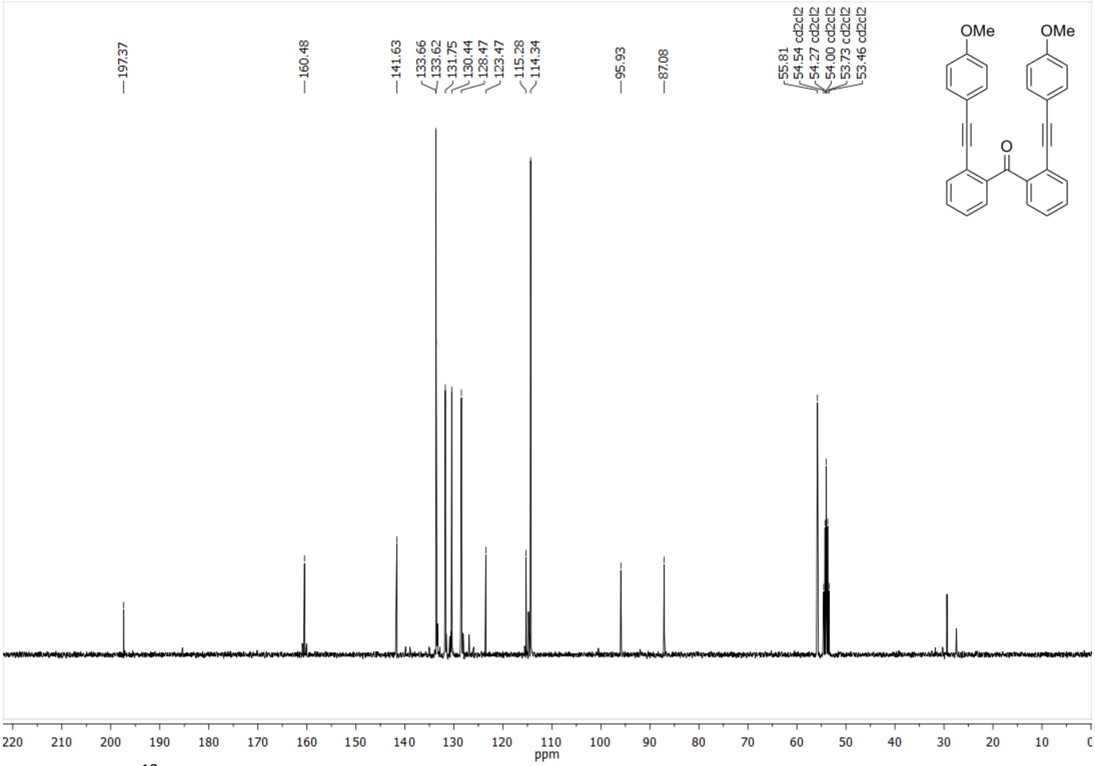

Figure S14: ^{13}C NMR spectrum of **3c** in CD_2Cl_2 (101 MHz).

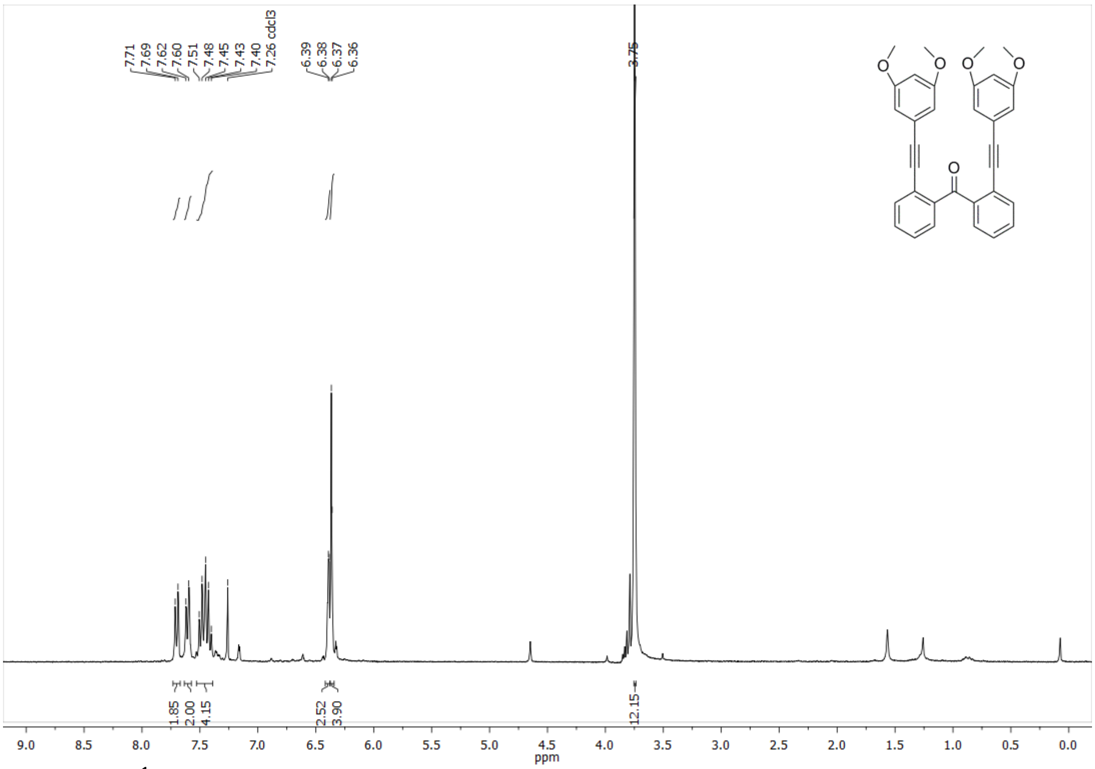

Figure S15: ^{1}H NMR spectrum of **3d** in $CDCl_3$ (300 MHz).

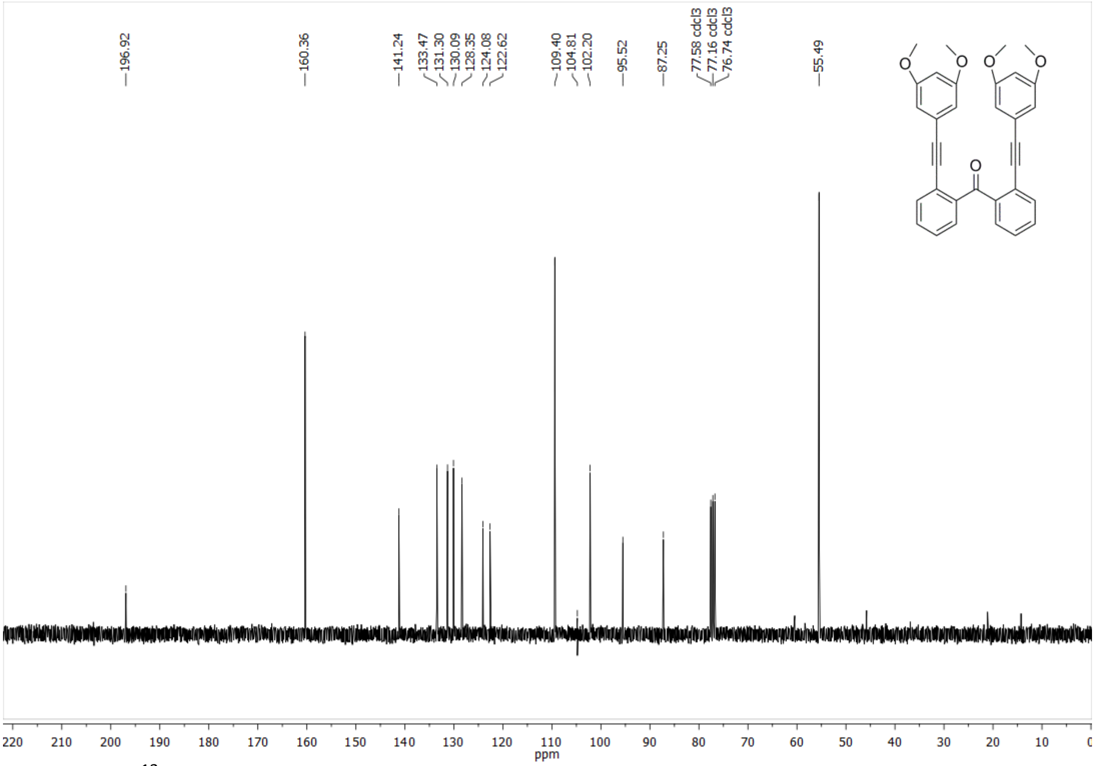

Figure S16: ^{13}C NMR spectrum of **3d** in $CDCl_3$ (75 MHz).

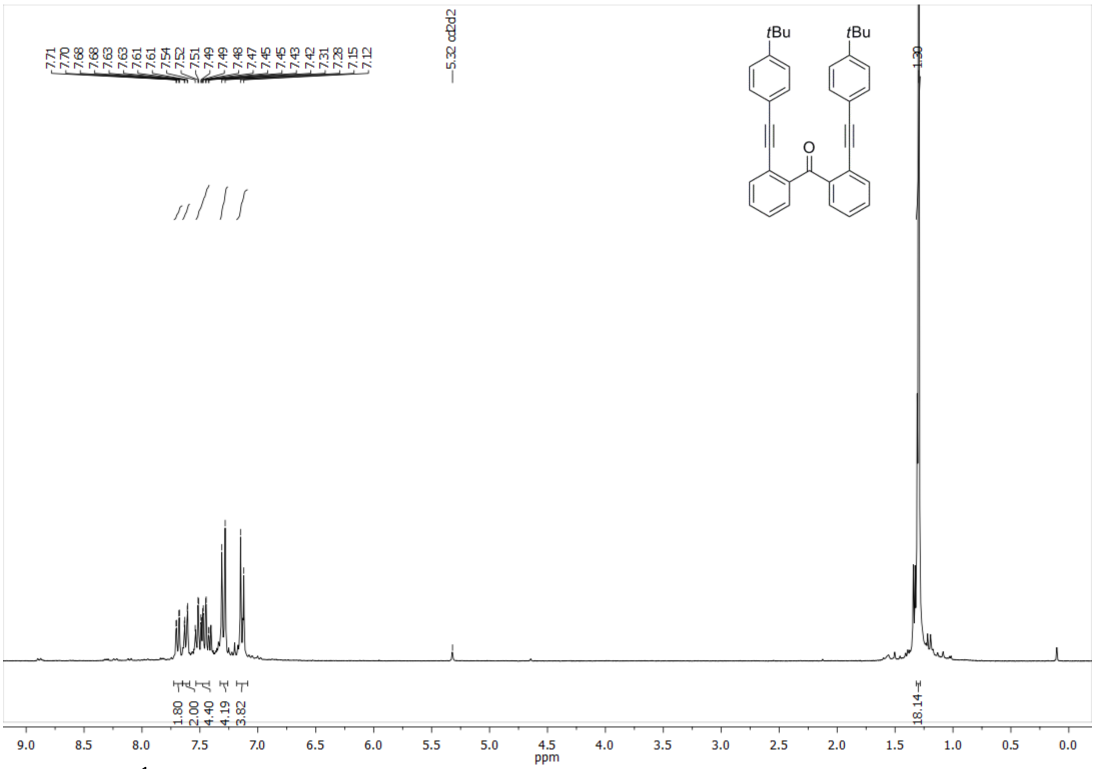

Figure S17: ^{1}H NMR spectrum of **3e** in CD_2Cl_2 (300 MHz).

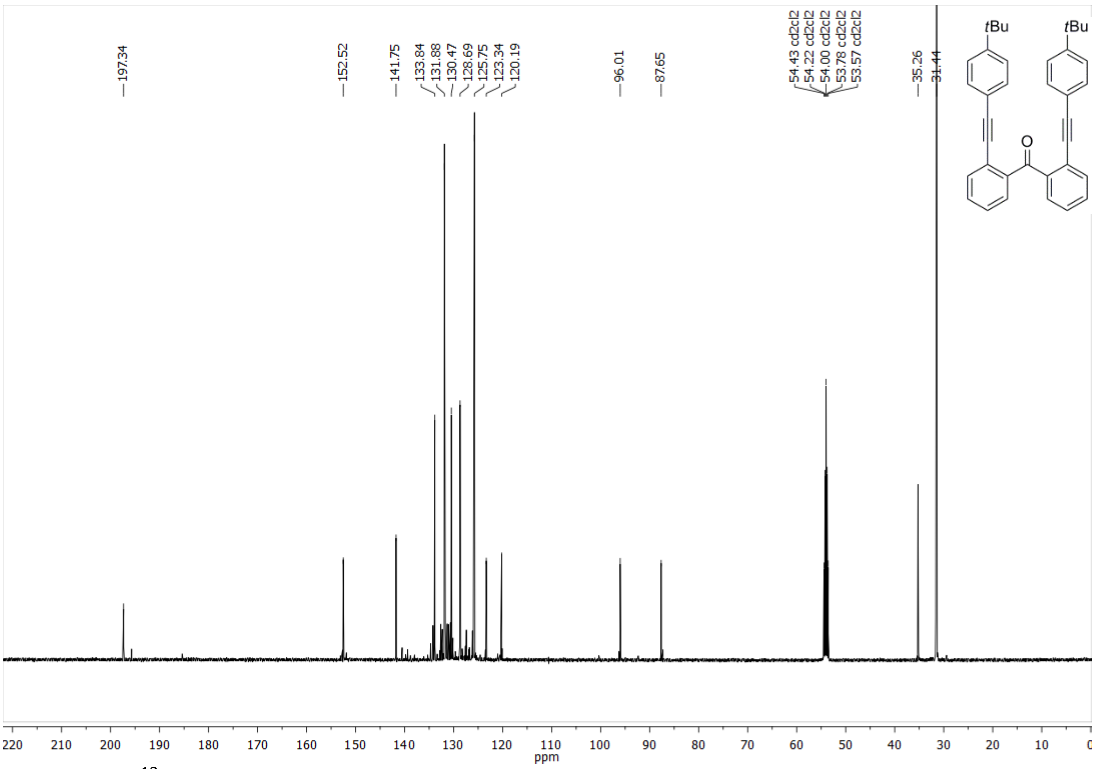

Figure S18: ^{13}C NMR spectrum of **3e** in CD_2Cl_2 (126 MHz).

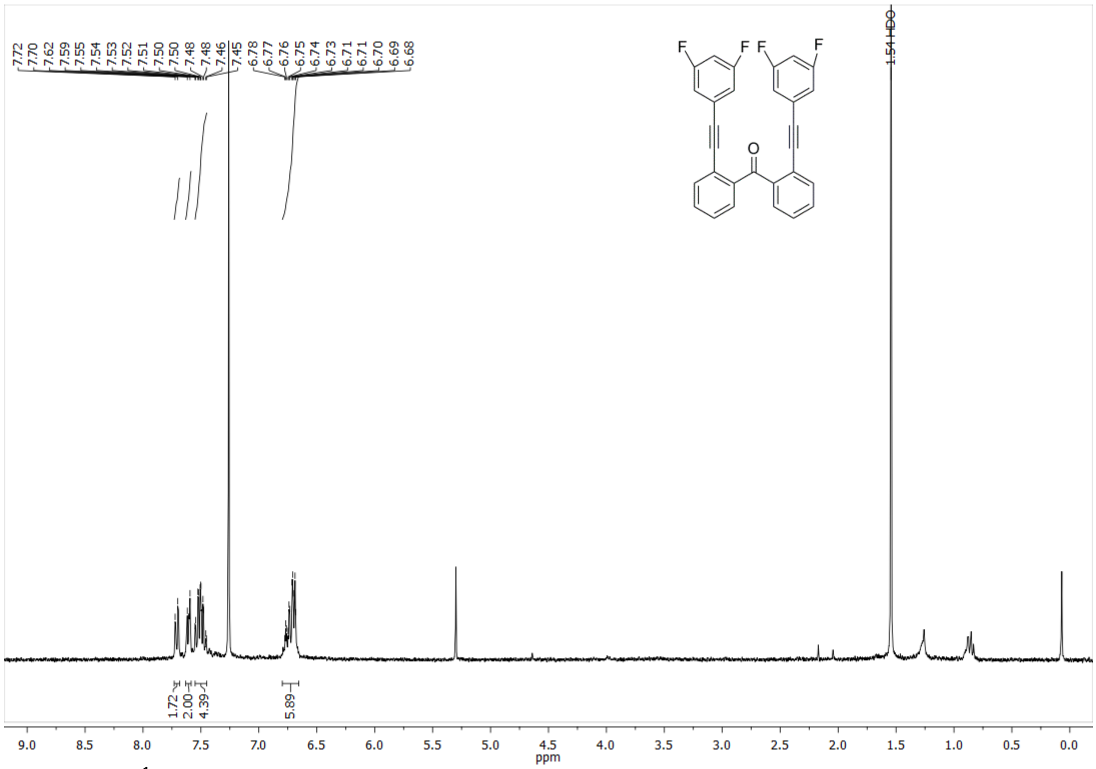

Figure S19: ^{1}H NMR spectrum of **3f** in $CDCl_3$ (300 MHz) with residual water peak at 1.54 ppm.

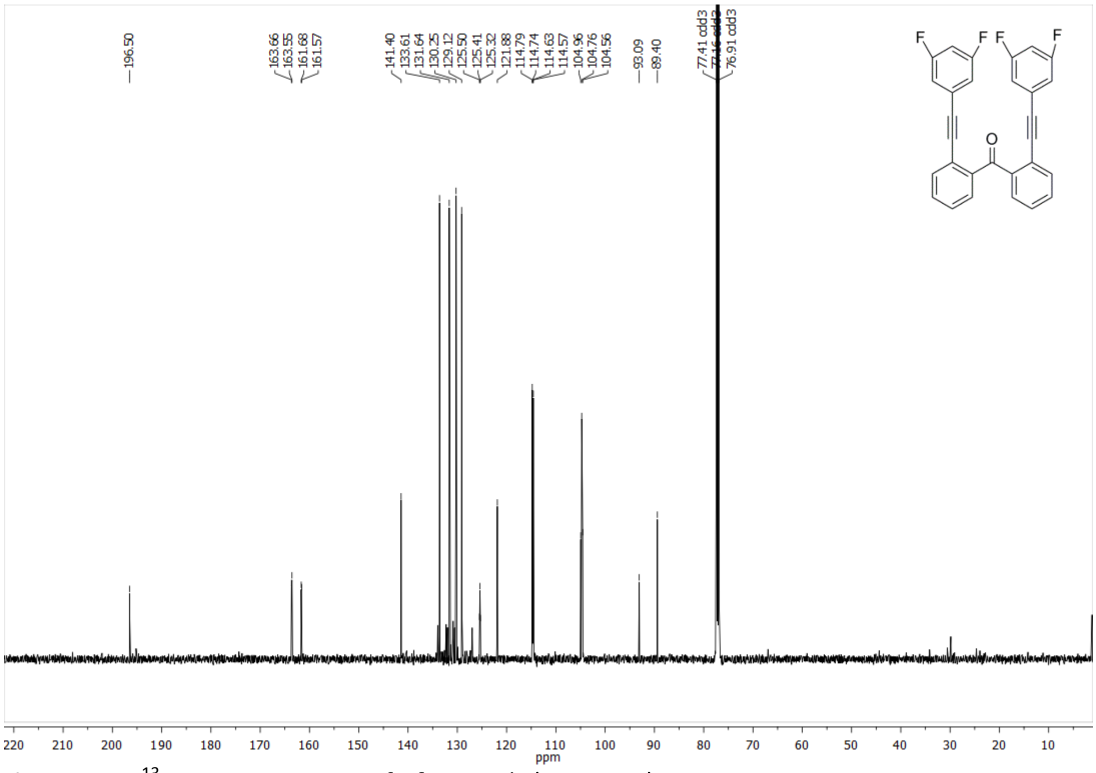

Figure S20: ^{13}C NMR spectrum of **3f** in $CDCl_3$ (126 MHz).

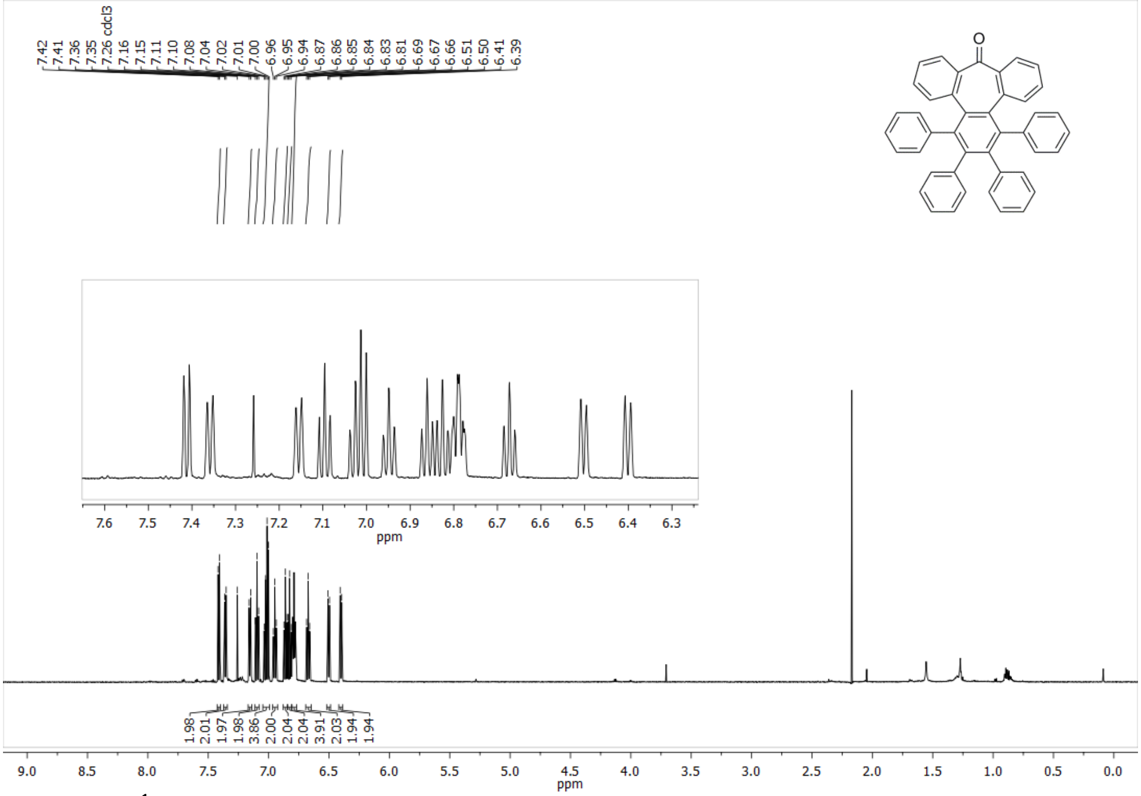

Figure S21: ^{1}H NMR spectrum of **5a** in $CDCl_3$ (600 MHz).

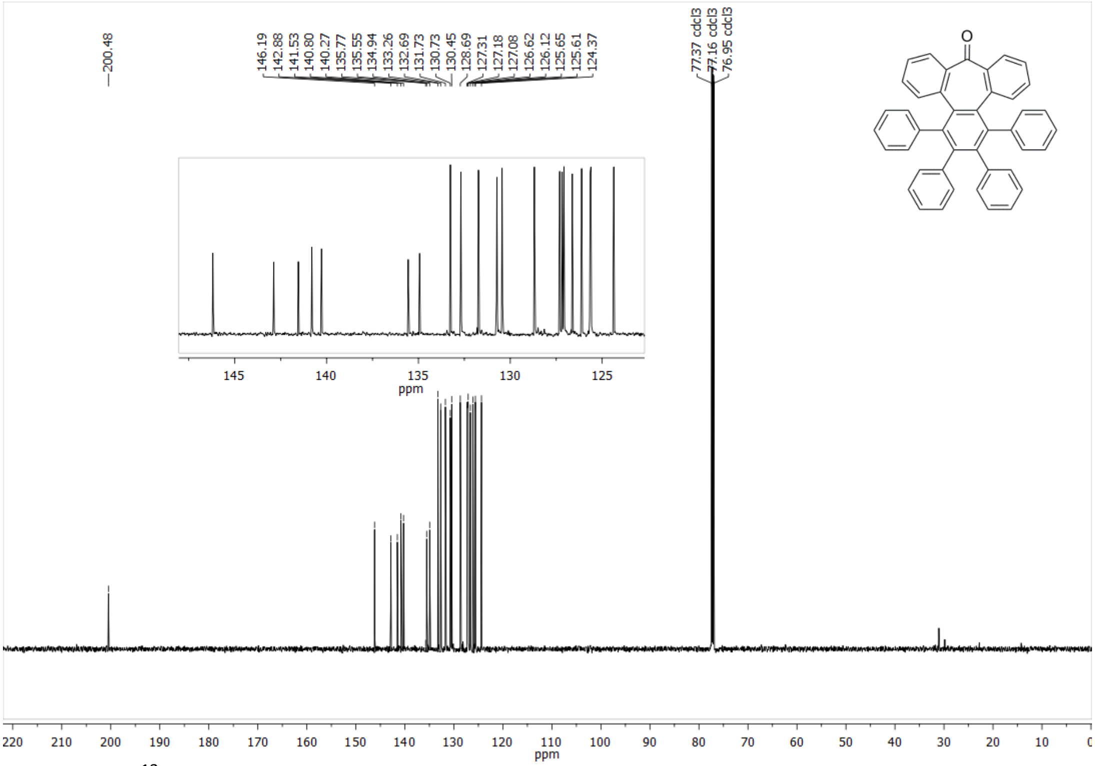

Figure S22: ^{13}C NMR spectrum of **5a** in $CDCl_3$ (151 MHz).

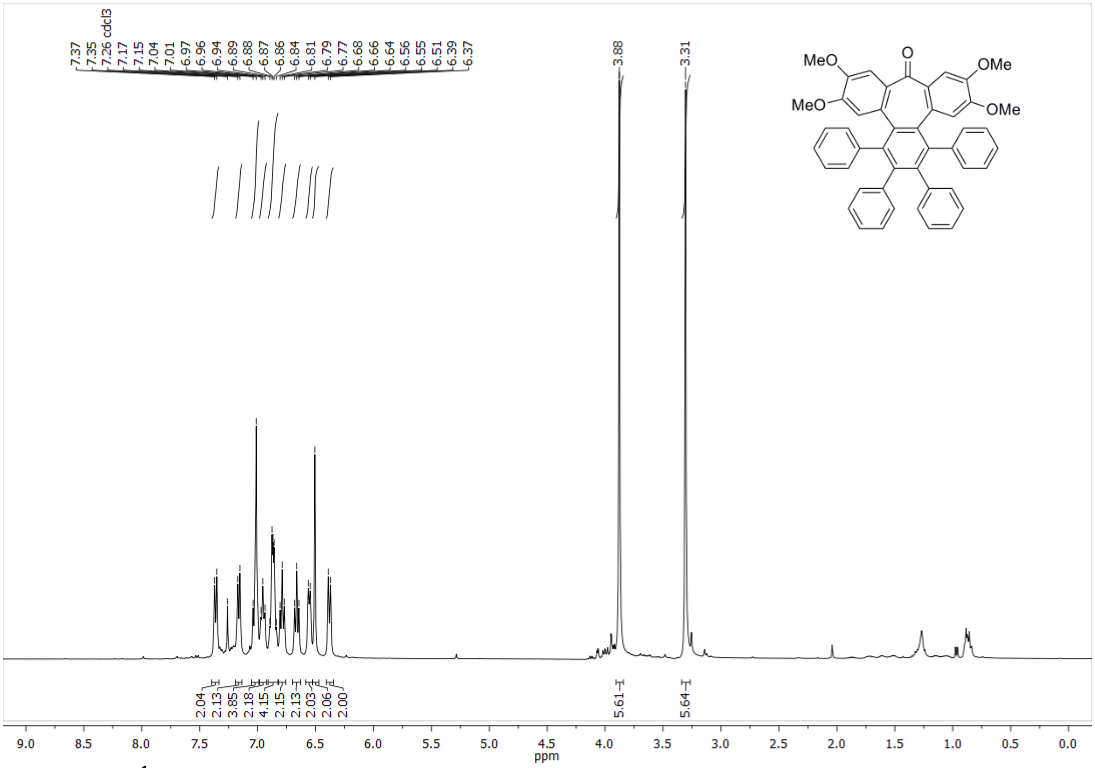

Figure S23: ^{1}H NMR spectrum of **5b** in $CDCl_3$ (400 MHz).

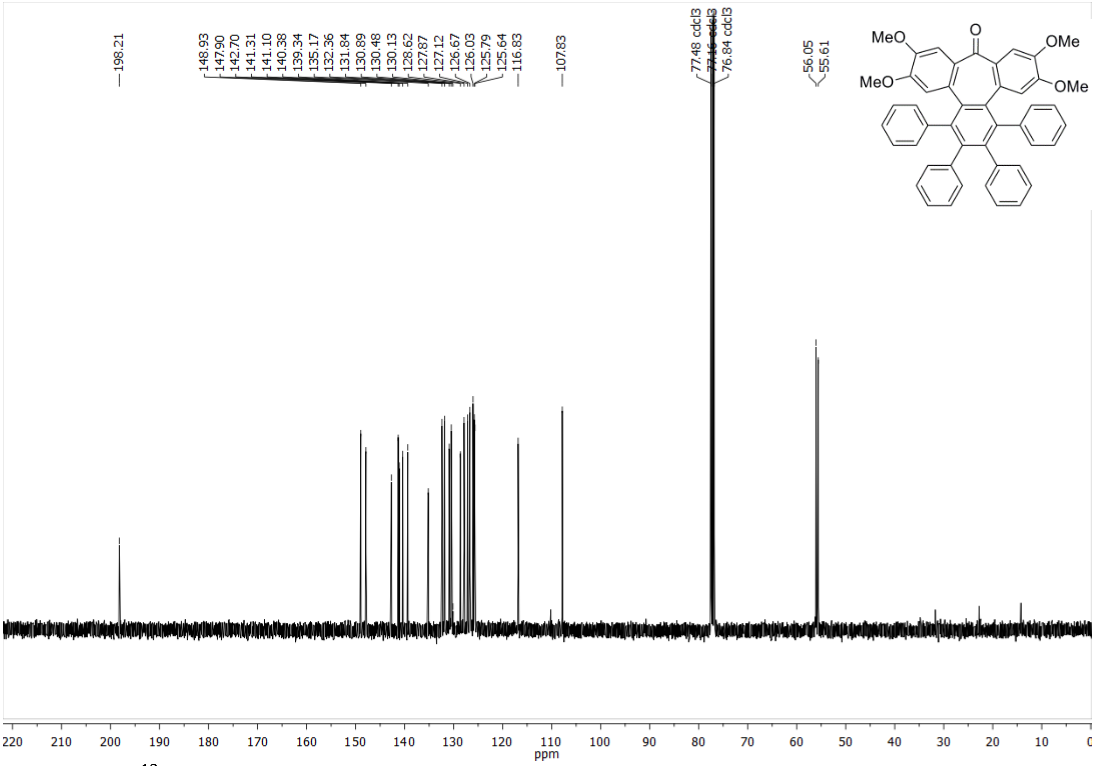

Figure S24: ^{13}C NMR spectrum of **5b** in $CDCl_3$ (101 MHz).

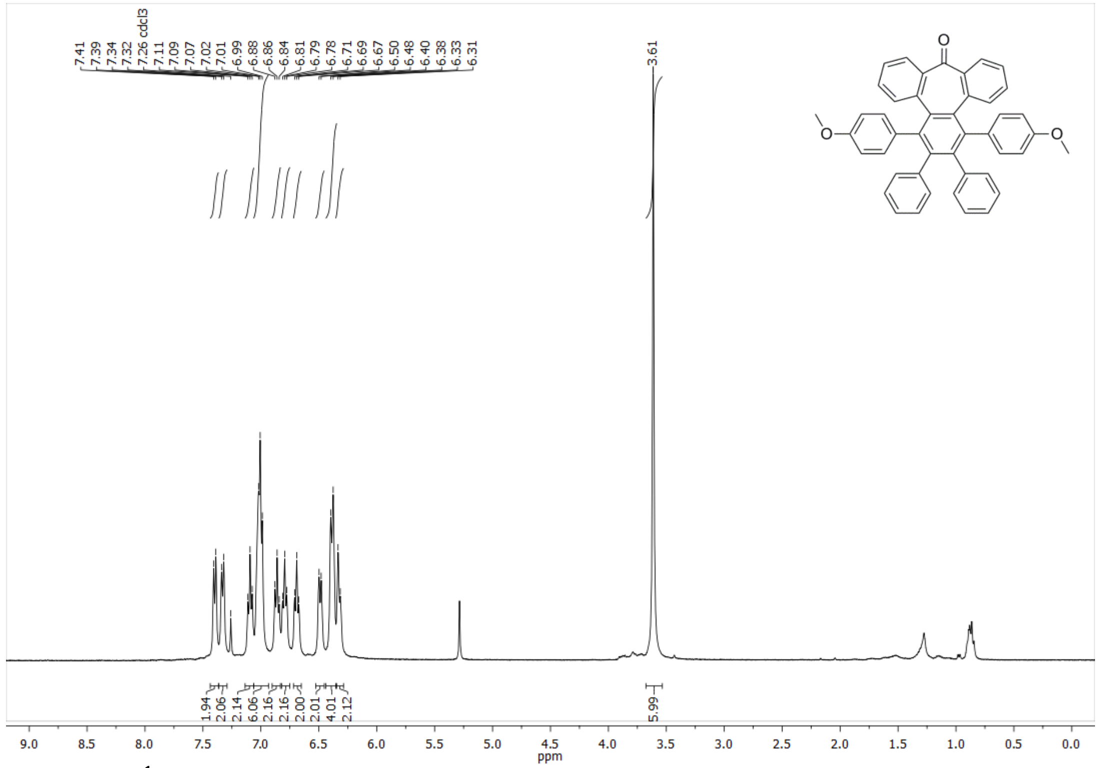

Figure S25: ^{1}H NMR spectrum of **5c** in $CDCl_3$ (400 MHz).

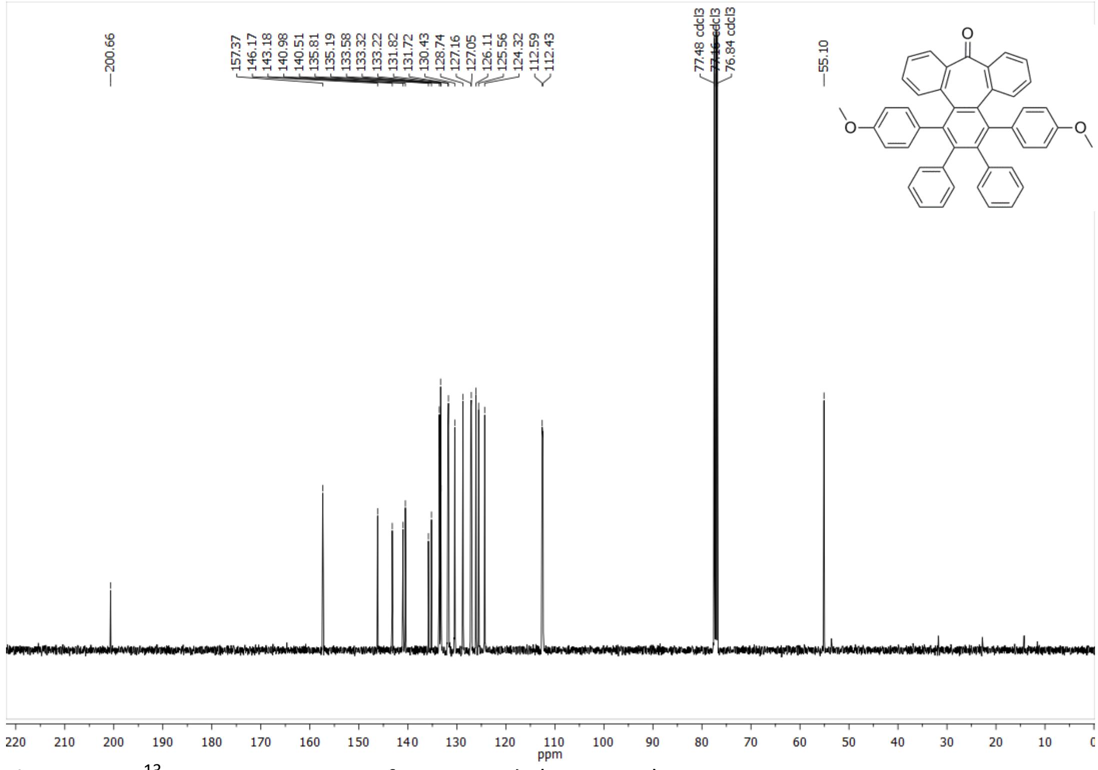

Figure S26: ^{13}C NMR spectrum of **5c** in $CDCl_3$ (101 MHz).

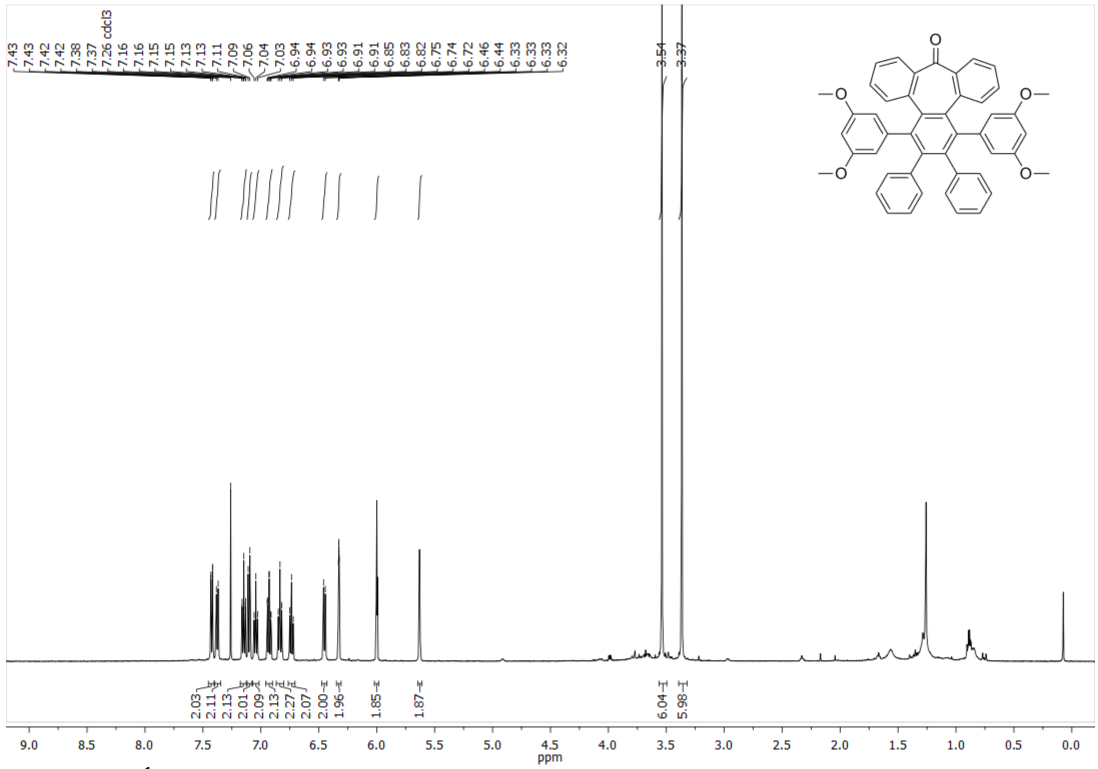

Figure S27: ^{1}H NMR spectrum of **5d** in $CDCl_3$ (500 MHz).

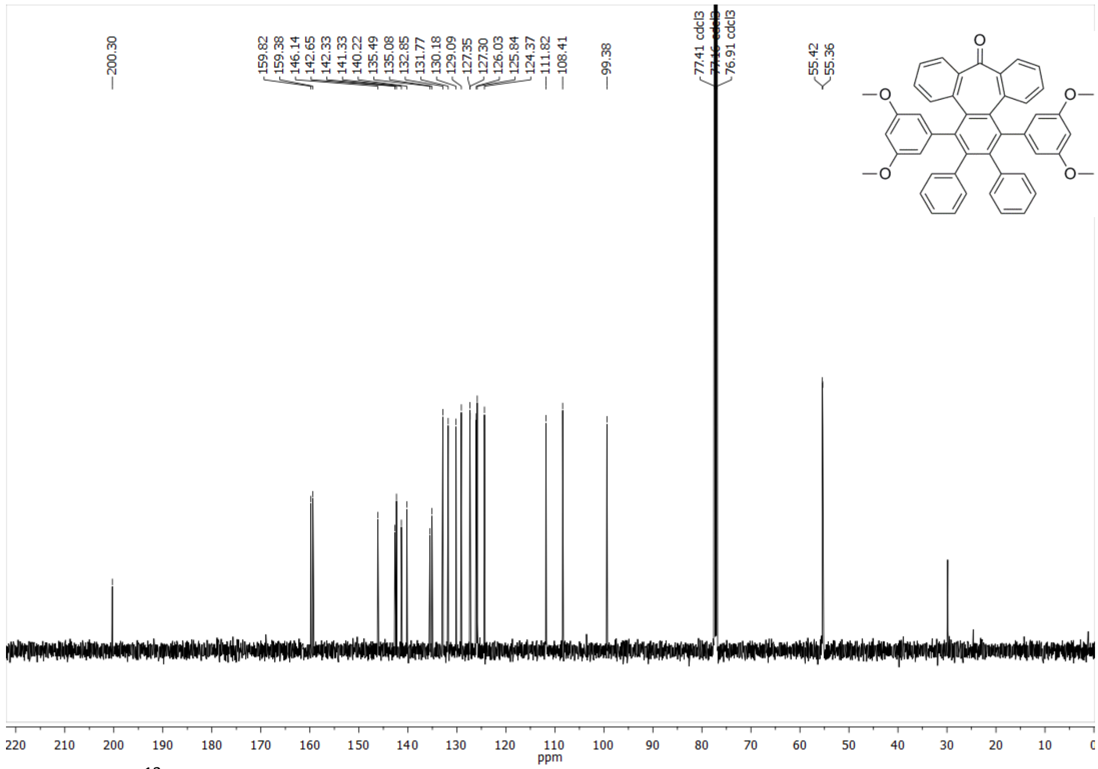

Figure S28: ^{13}C NMR spectrum of **5d** in $CDCl_3$ (126 MHz).

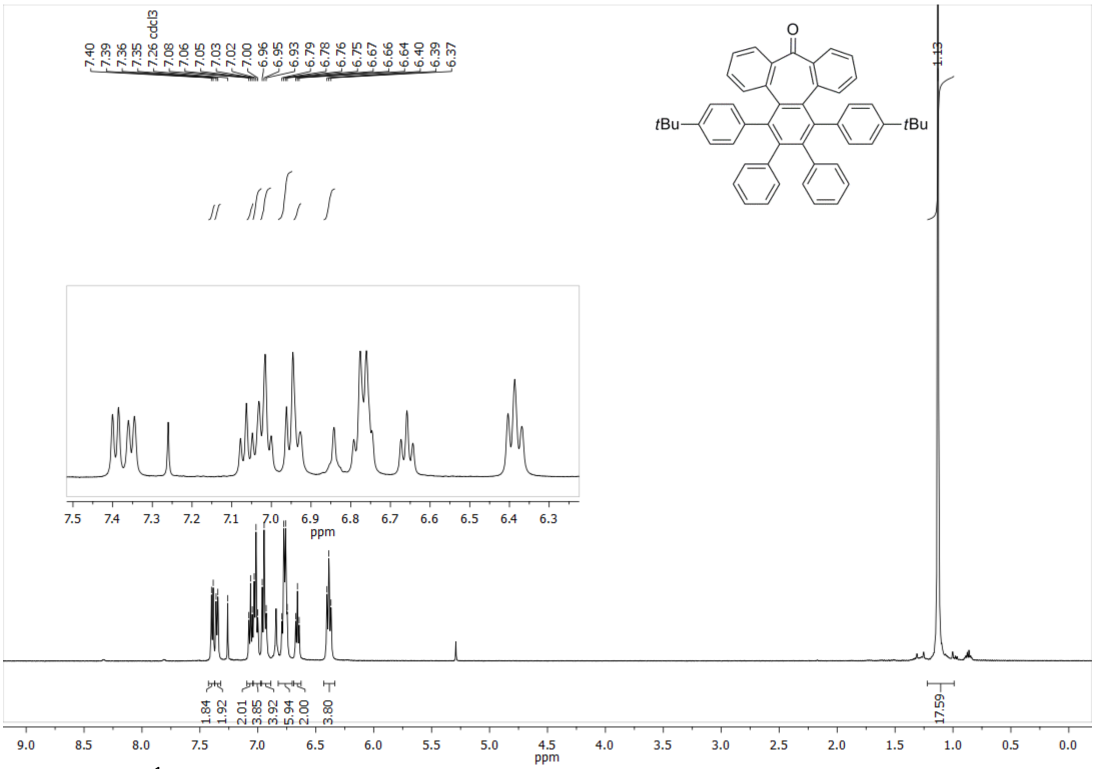

Figure S29: ^{1}H NMR spectrum of **5e** in $CDCl_3$ (500 MHz).

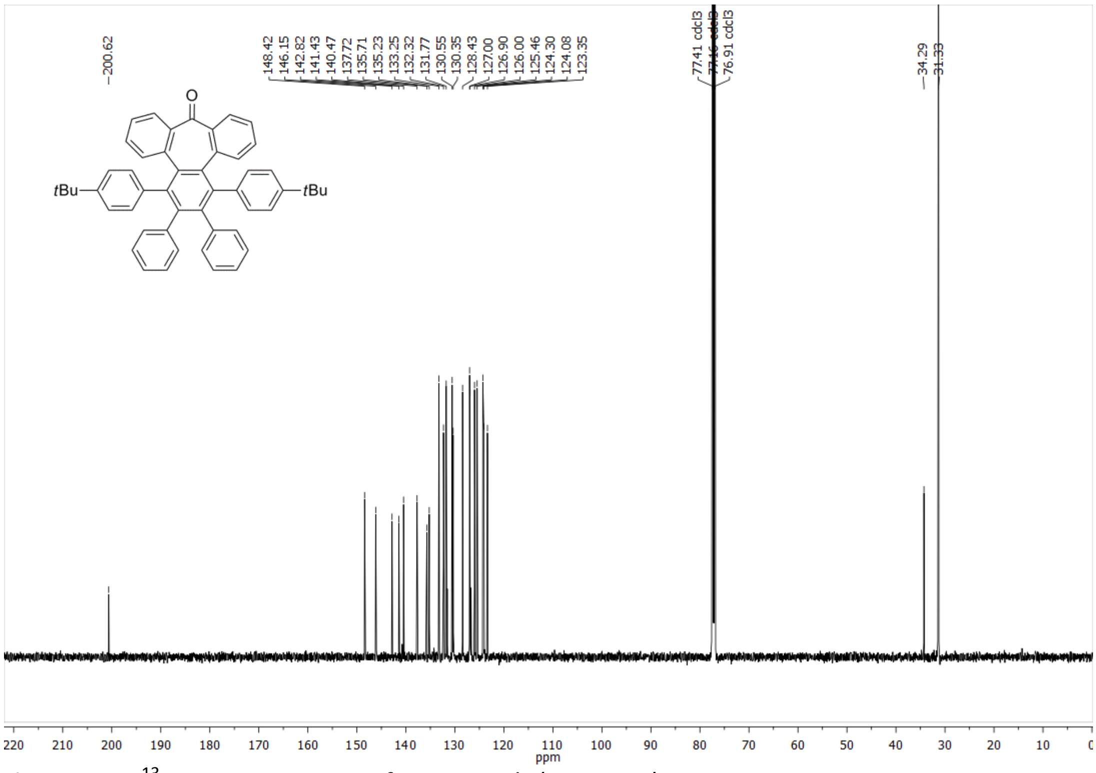

Figure S30: ^{13}C NMR spectrum of **5e** in $CDCl_3$ (126 MHz).

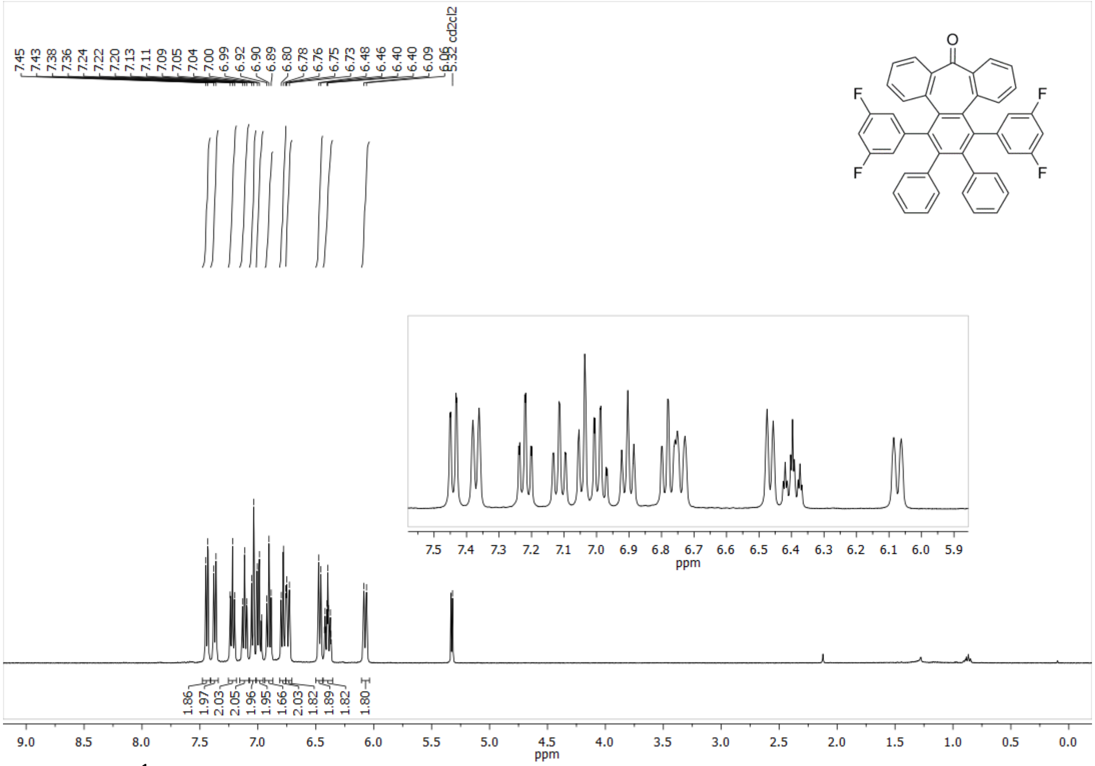

Figure S31: ^{1}H NMR spectrum of **5f** in CD_2Cl_2 (400 MHz).

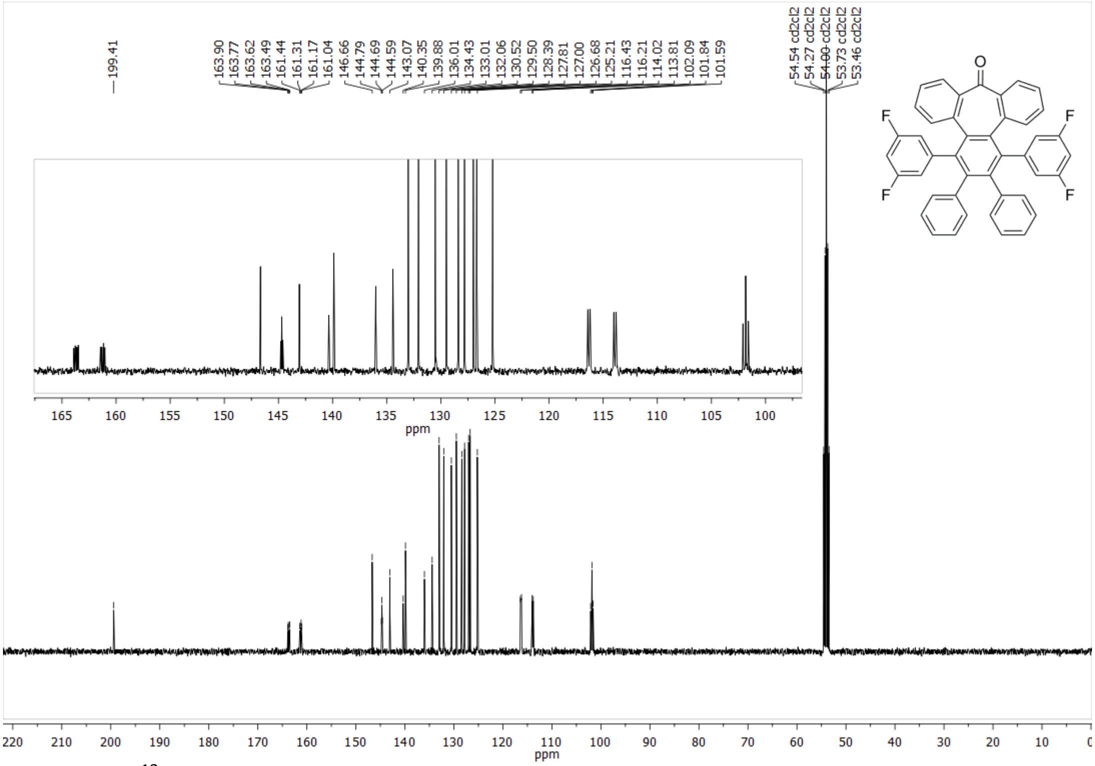

Figure S32: ^{13}C NMR spectrum of **5f** in CD_2Cl_2 (101 MHz).

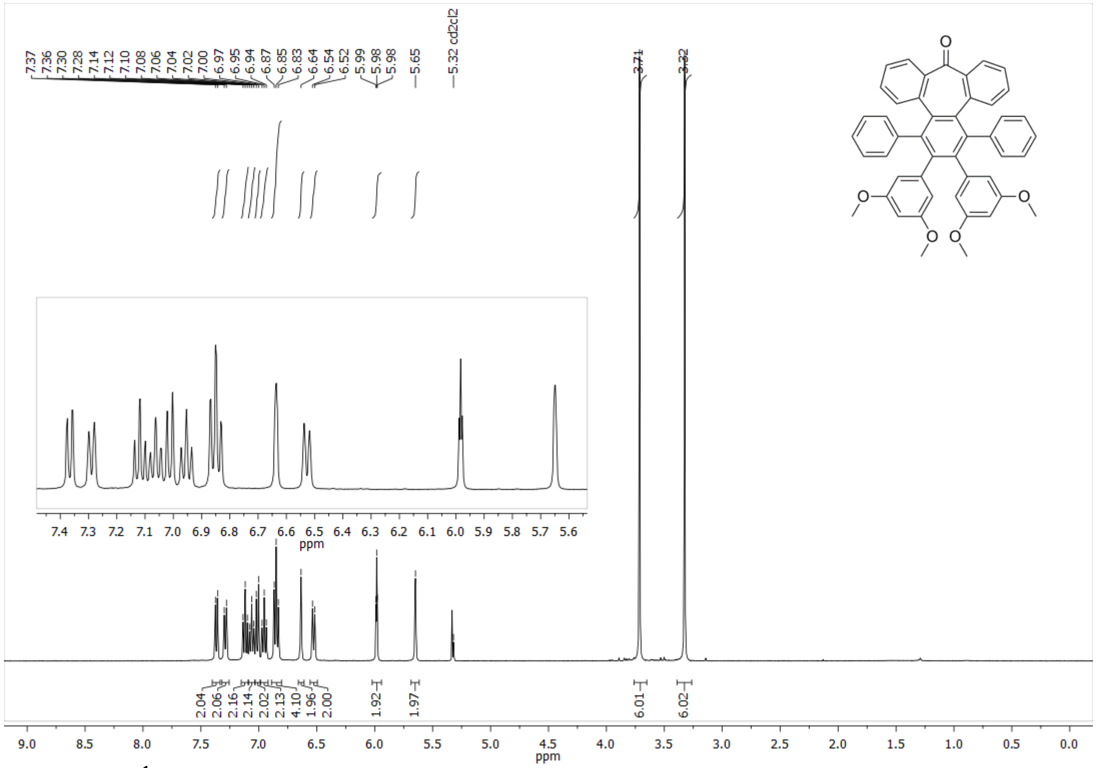

Figure S33: ^{1}H NMR spectrum of **5g** in CD_2Cl_2 (500 MHz).

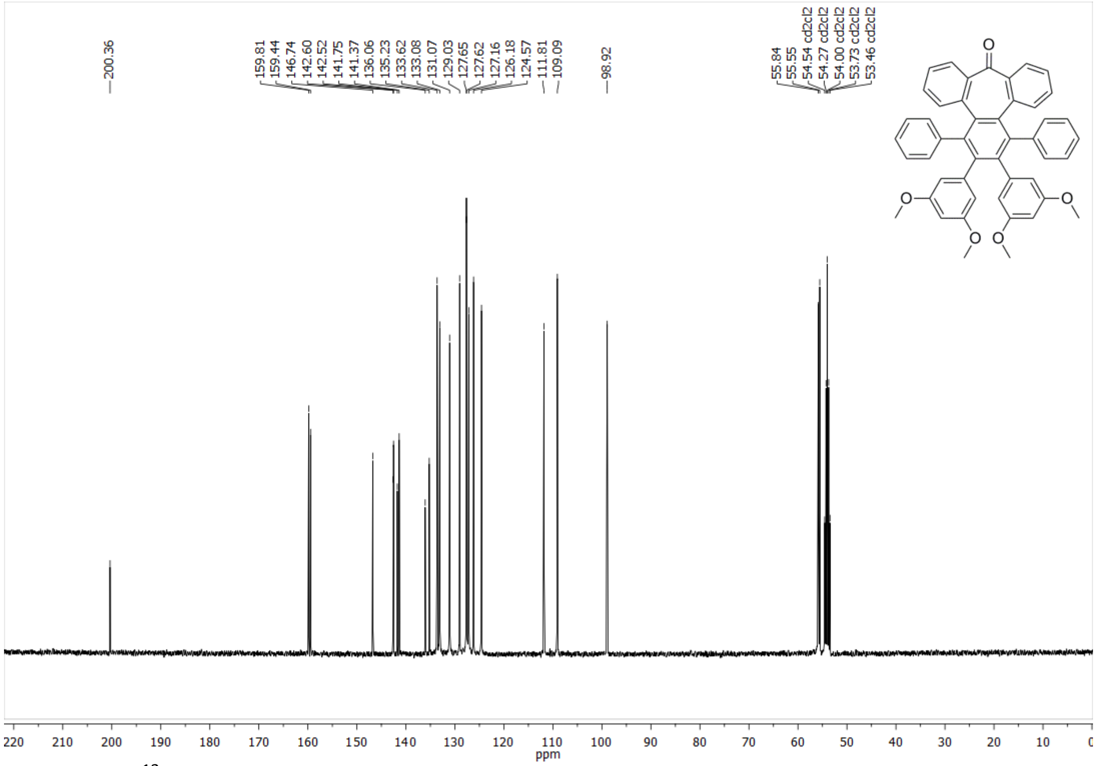

Figure S34: ^{13}C NMR spectrum of **5g** in CD_2Cl_2 (126 MHz).

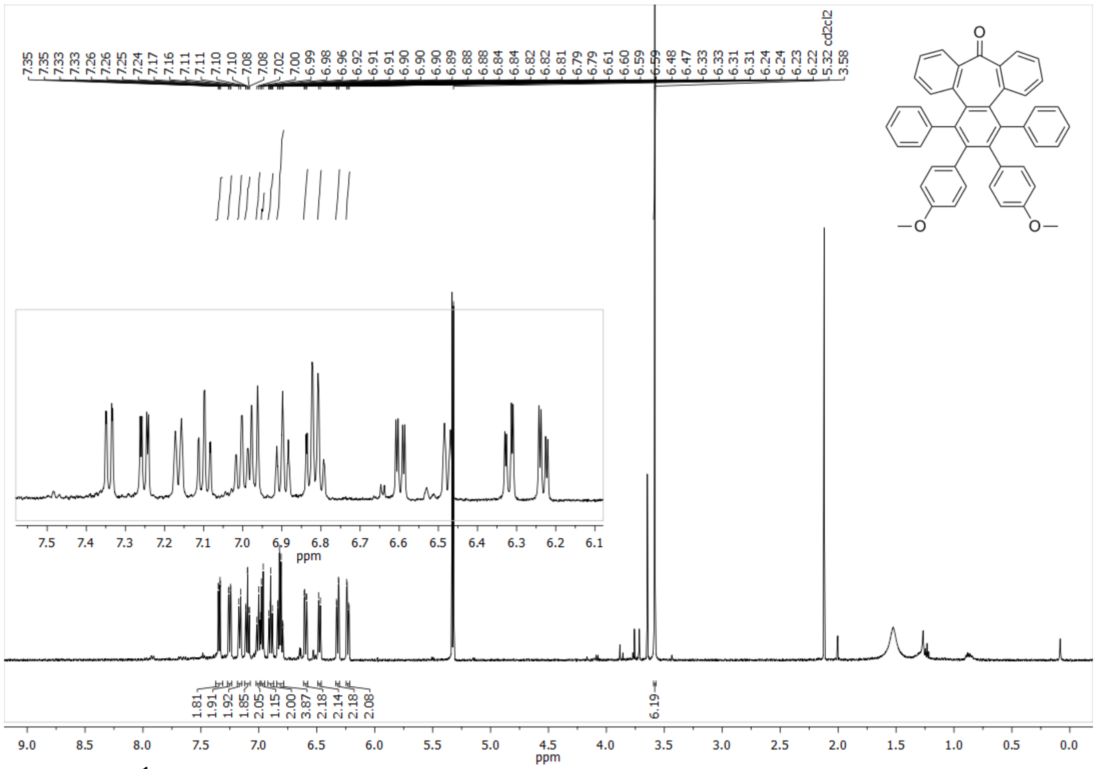

Figure S35: ^{1}H NMR spectrum of **5h** in CD_2Cl_2 (500 MHz).

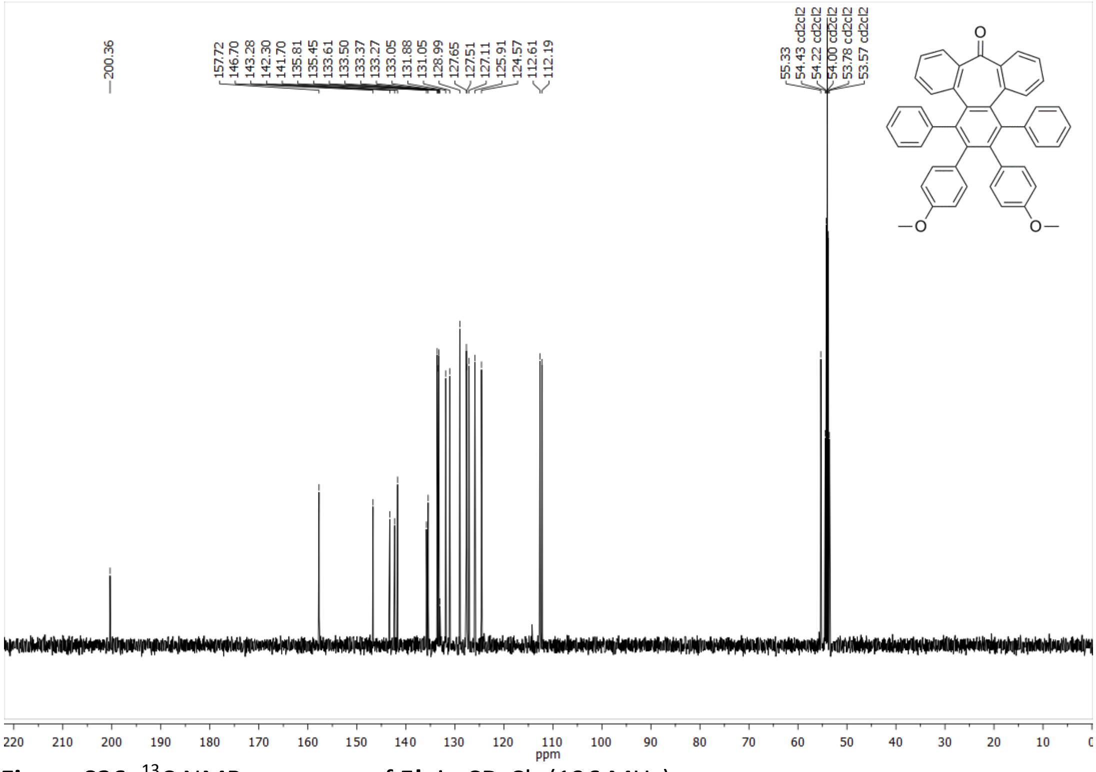

Figure S36: ^{13}C NMR spectrum of **5h** in CD_2Cl_2 (126 MHz).

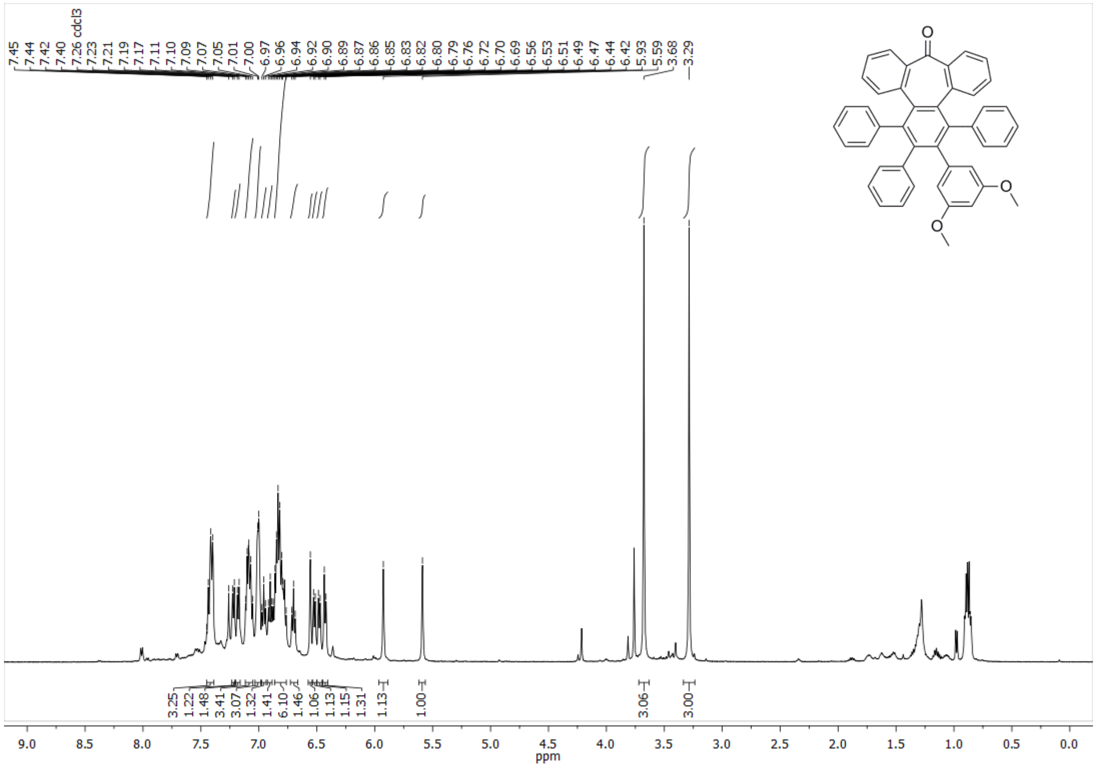

Figure S37: ^{1}H NMR spectrum of **5i** in $CDCl_3$ (500 MHz).

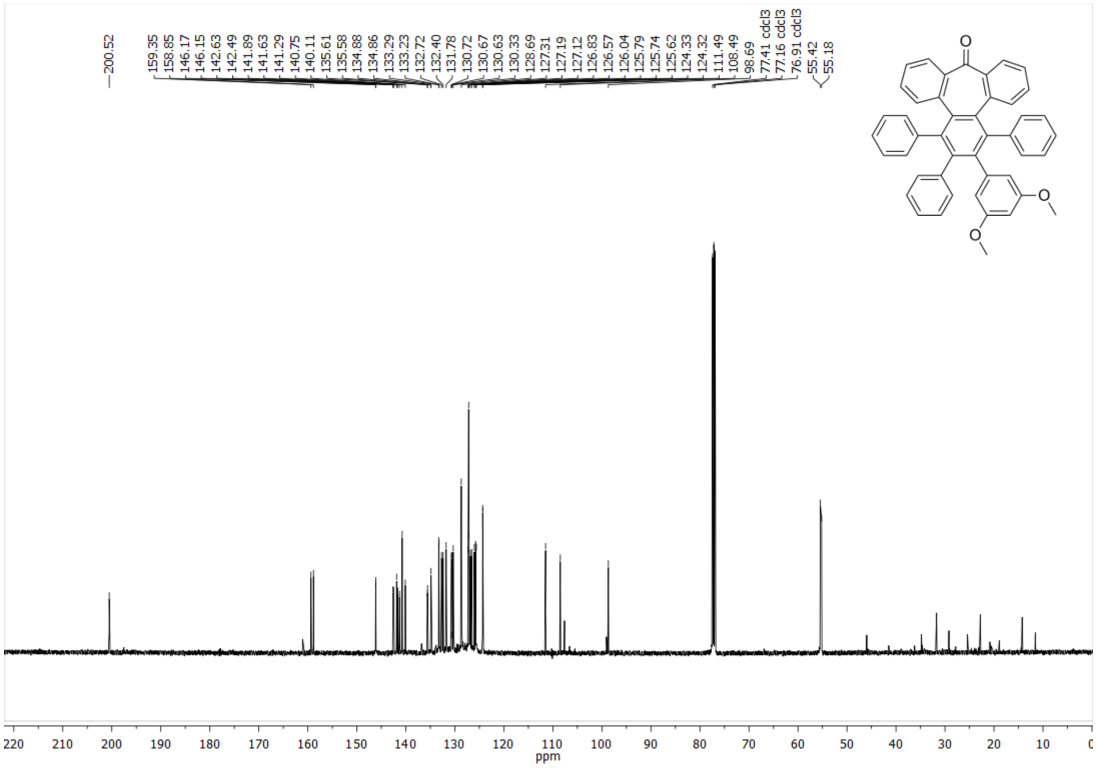

Figure S38: ^{13}C NMR spectrum of **5i** in $CDCl_3$ (126 MHz).

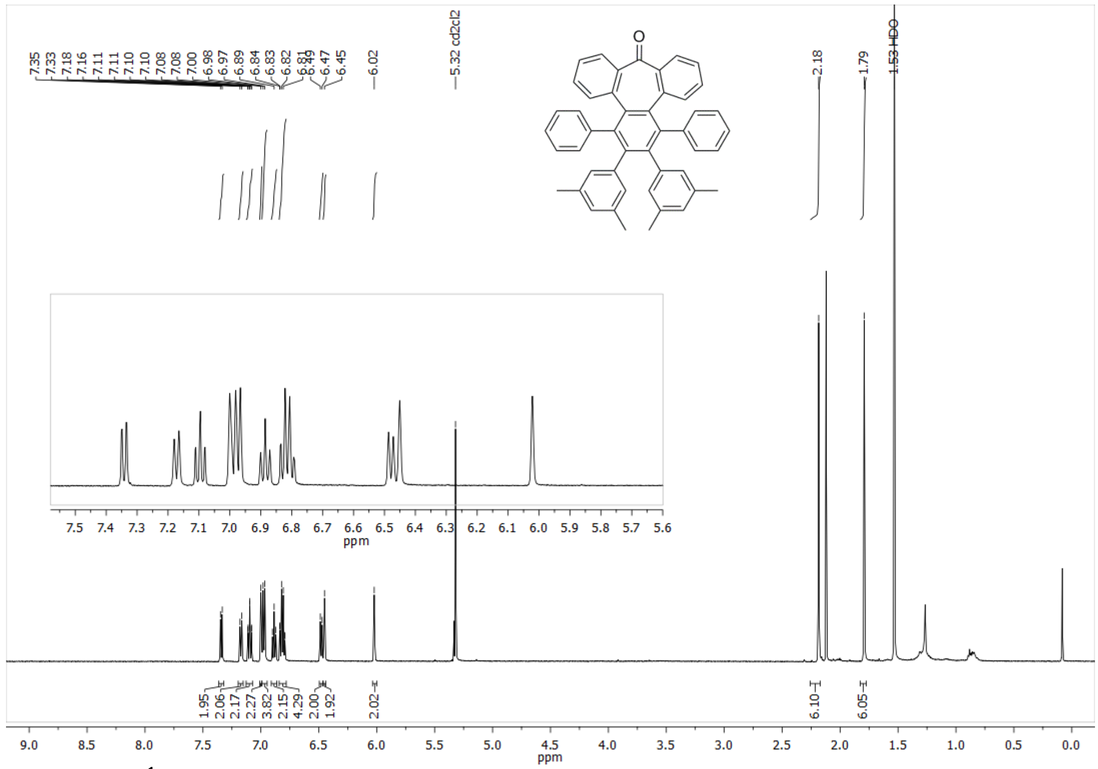

Figure S39: ^{1}H NMR spectrum of **5j** in CD_2Cl_2 (500 MHz) with residual water peak at 1.53 ppm and traces of acetone at 2.12 ppm.

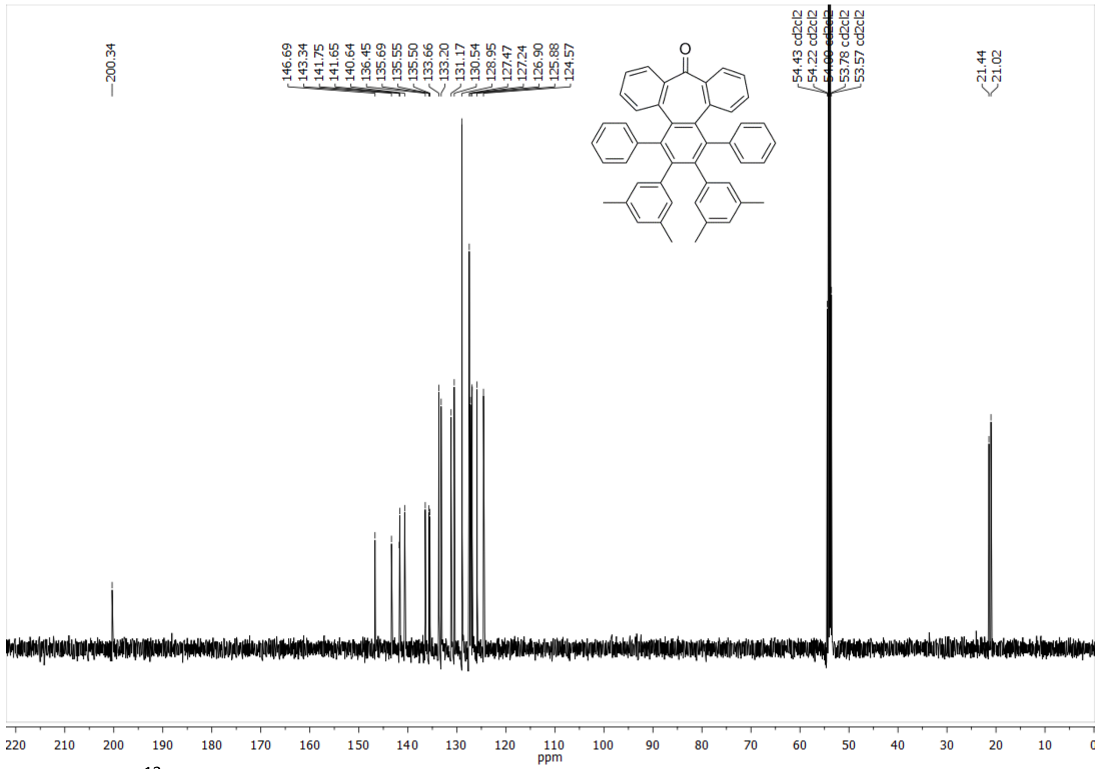

Figure S40: ^{13}C NMR spectrum of **5j** in CD_2Cl_2 (126 MHz).

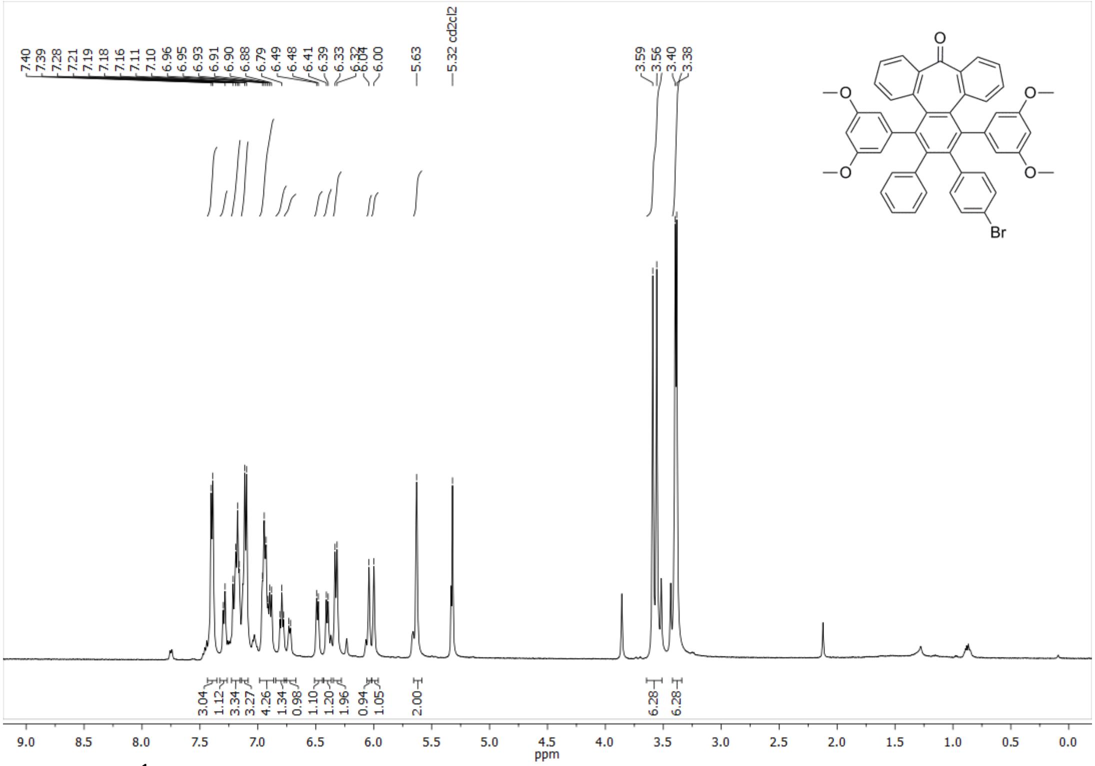

Figure S41: ^{1}H NMR spectrum of **5k** in CD_2Cl_2 (500 MHz).

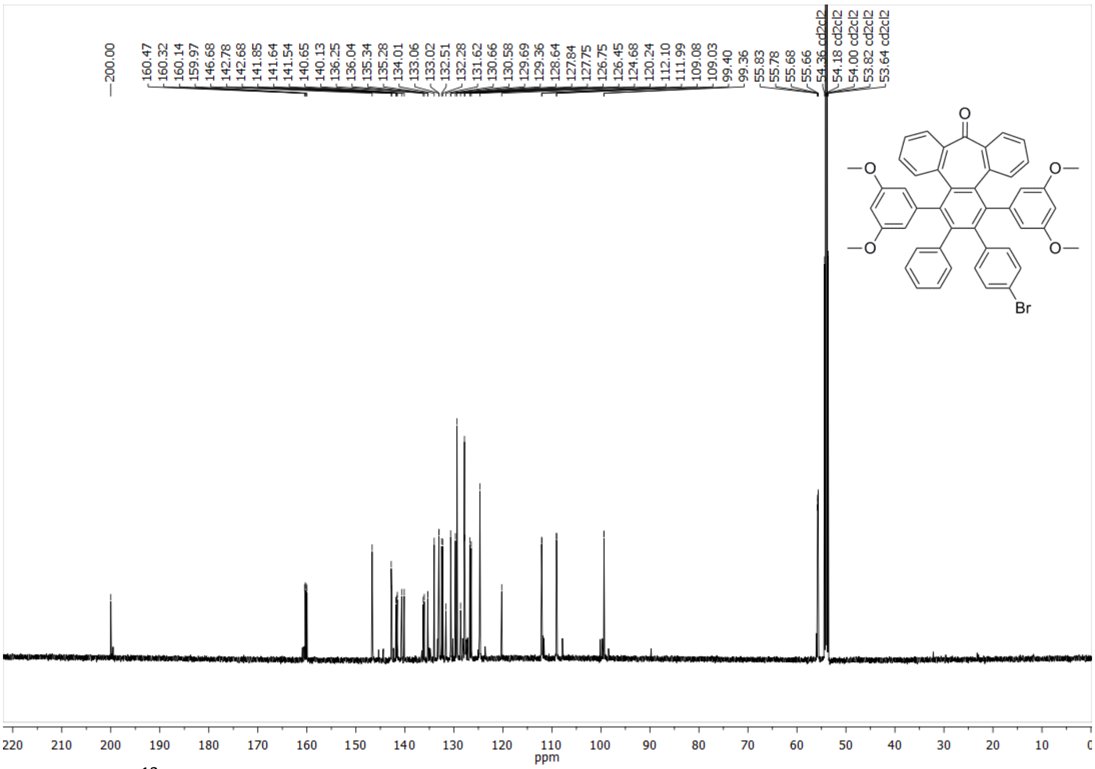

Figure S42: ^{13}C NMR spectrum of **5k** in CD_2Cl_2 (126 MHz).

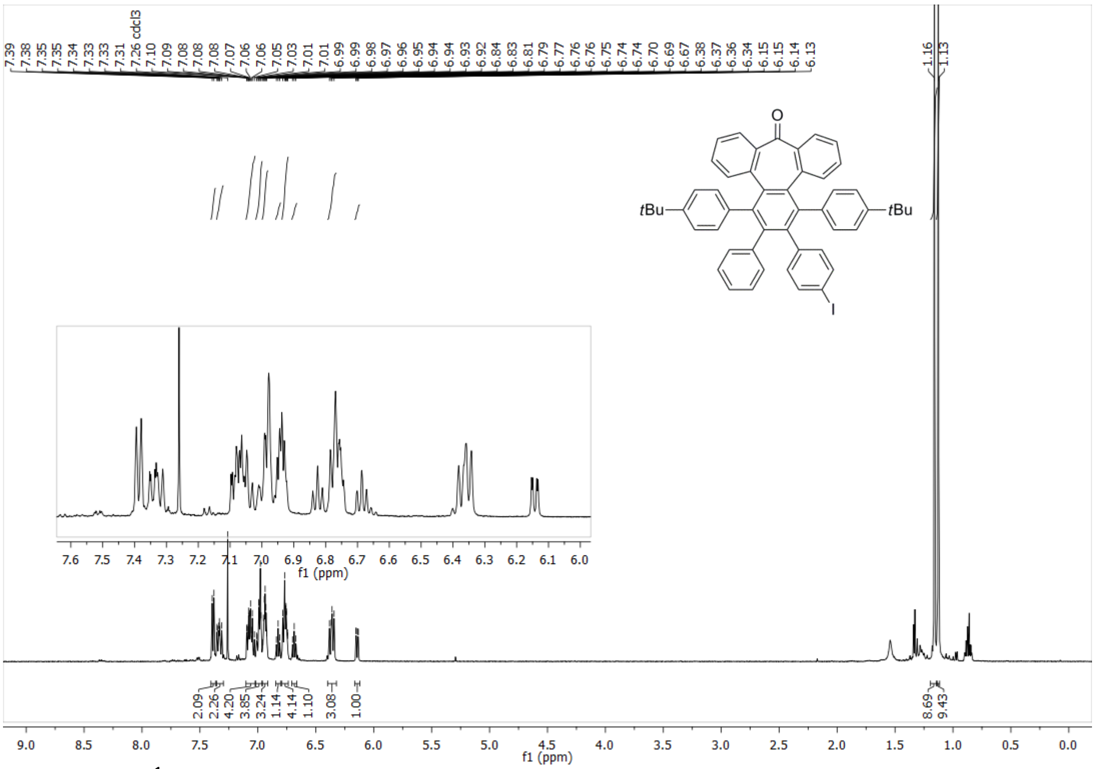

Figure S43: ^{1}H NMR spectrum of **5l** in $CDCl_3$ (500 MHz).

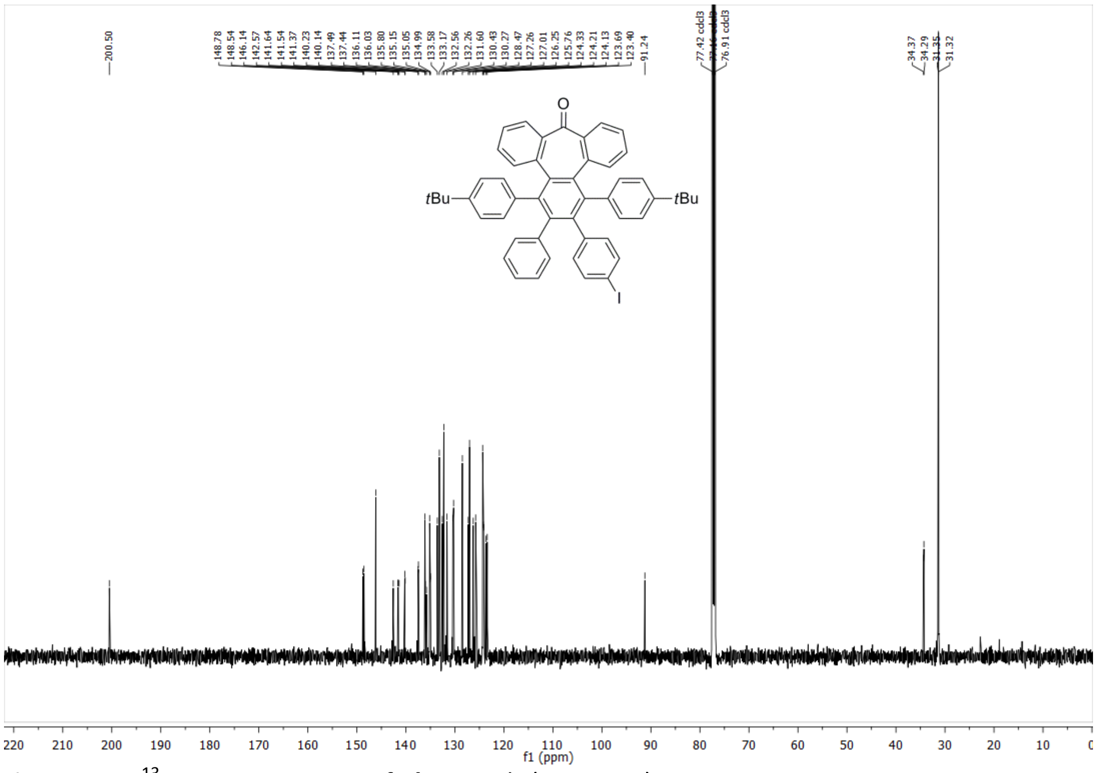

Figure S44: ^{13}C NMR spectrum of **5l** in $CDCl_3$ (126 MHz).

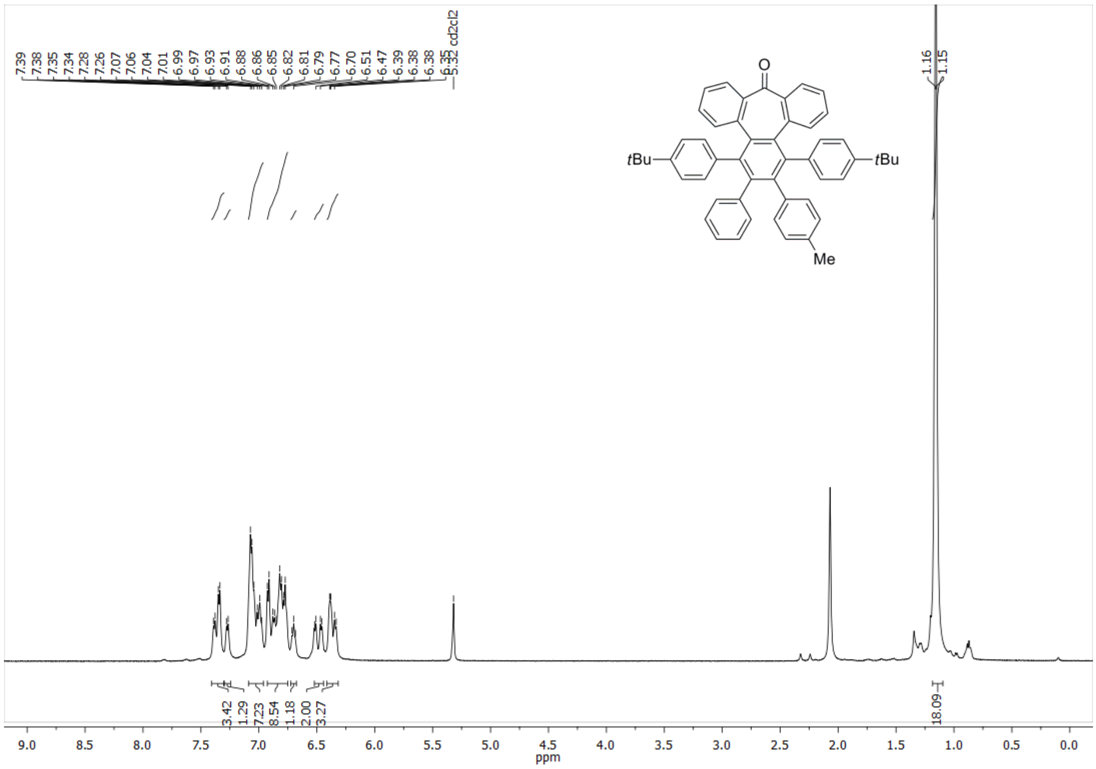

Figure S45: ^{1}H NMR spectrum of **5m** in CD_2Cl_2 (500 MHz) with traces of acetone at 2.12 ppm.

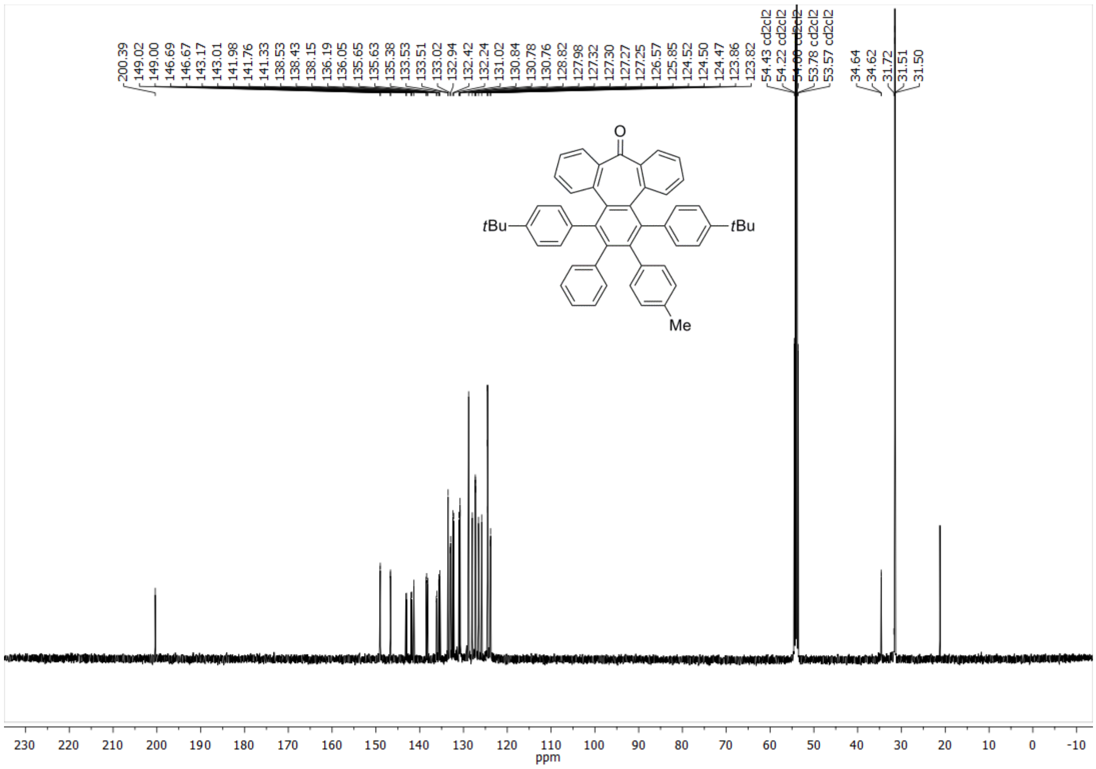

Figure S46: ^{13}C NMR spectrum of **5m** in CD_2Cl_2 (126 MHz).

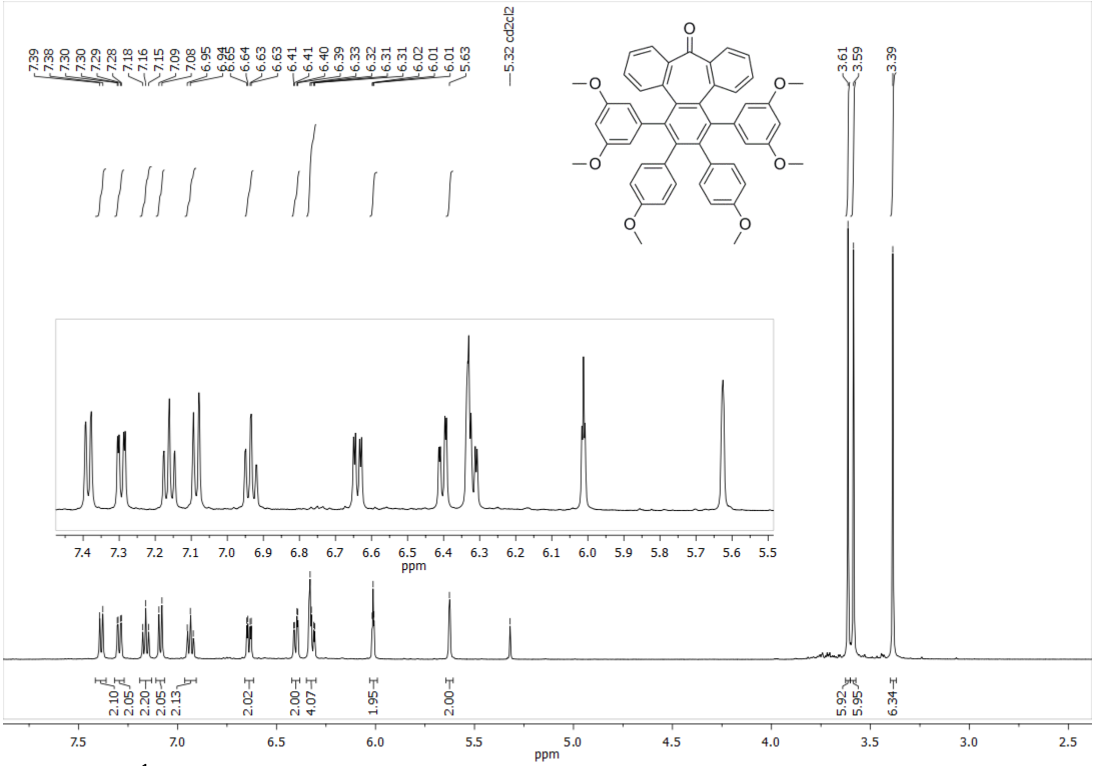

Figure S47: ^{1}H NMR spectrum of **5n** in CD_2Cl_2 (500 MHz).

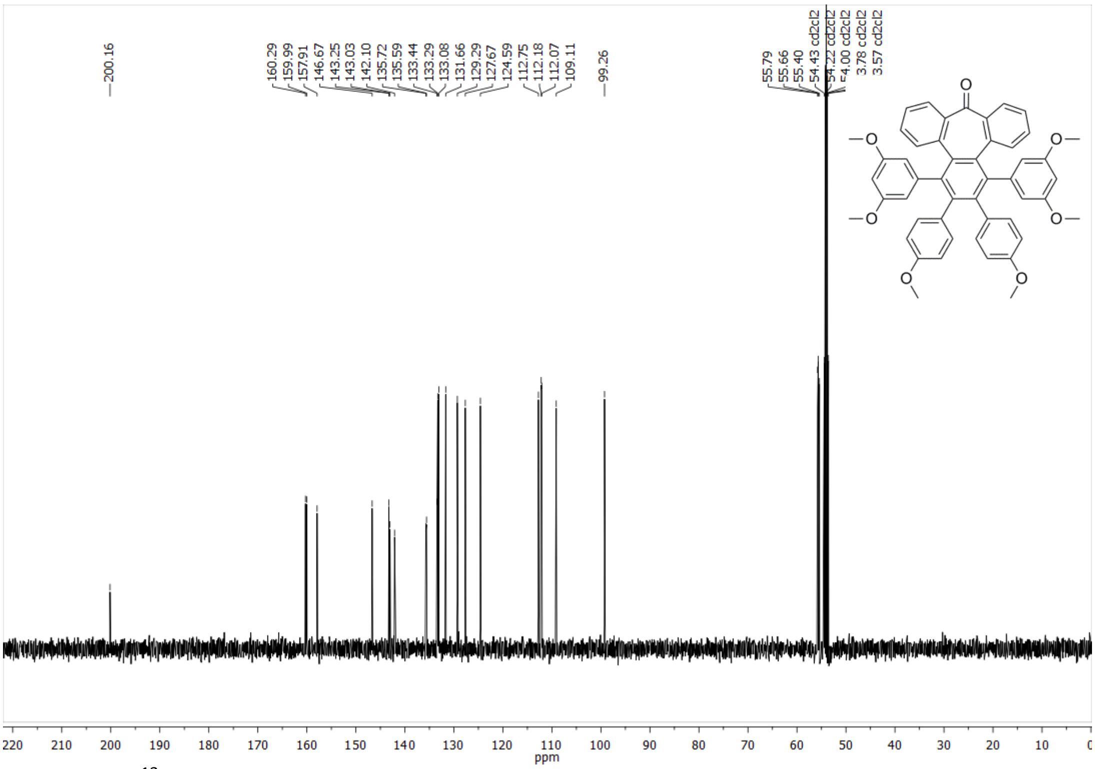

Figure S48: ^{13}C NMR spectrum of **5n** in CD_2Cl_2 (126 MHz).

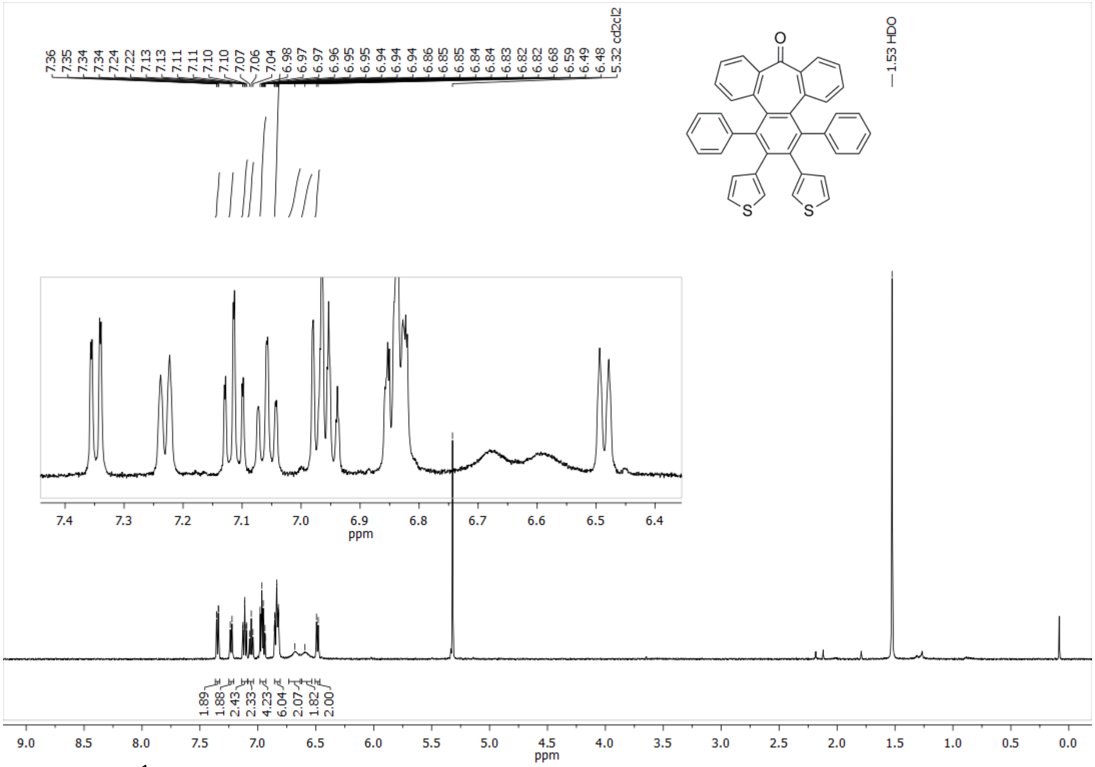

Figure S49: ^{1}H NMR spectrum of **5o** in CD_2Cl_2 (500 MHz).

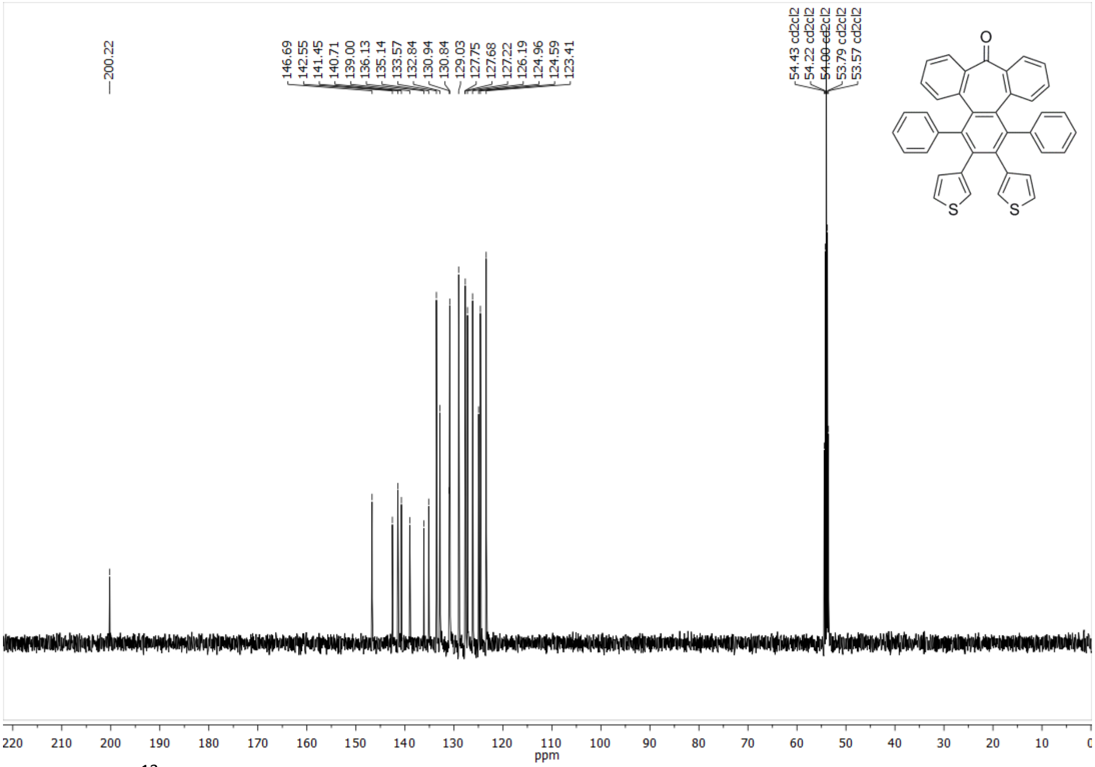

Figure S50: ^{13}C NMR spectrum of **5o** in CD_2Cl_2 (126 MHz).

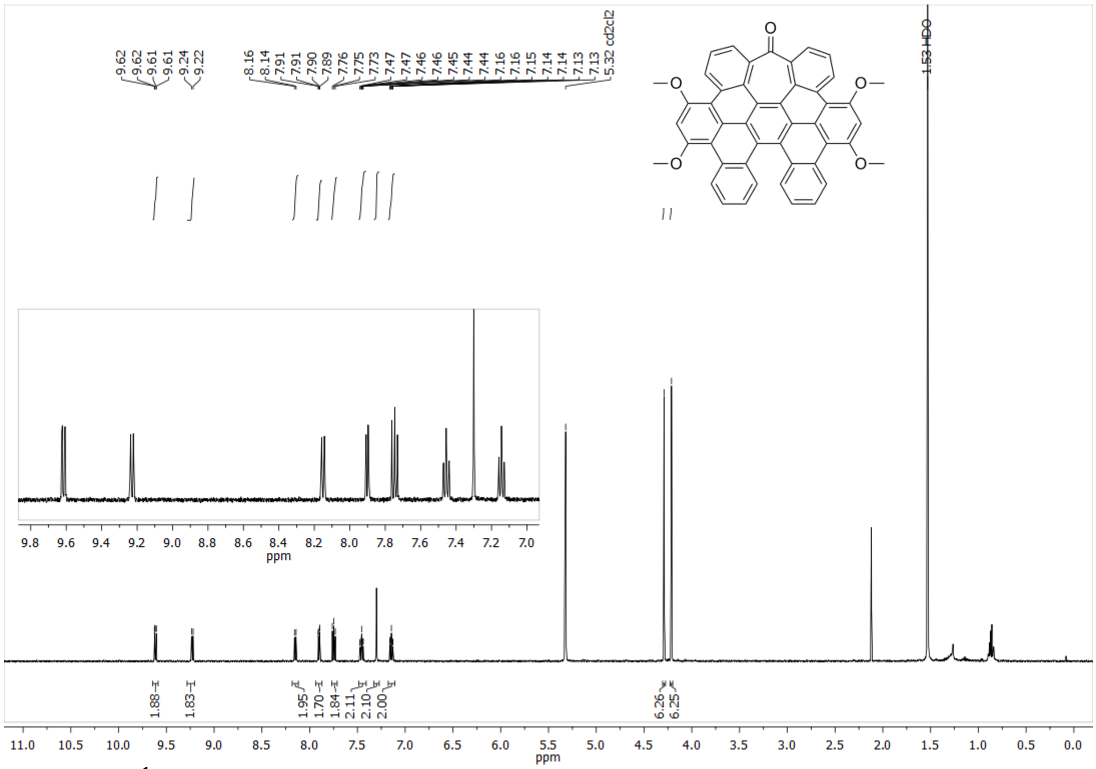

Figure S51: ^{1}H NMR spectrum of **6a** in CD_2Cl_2 (500 MHz) with traces of acetone at 2.12 ppm.

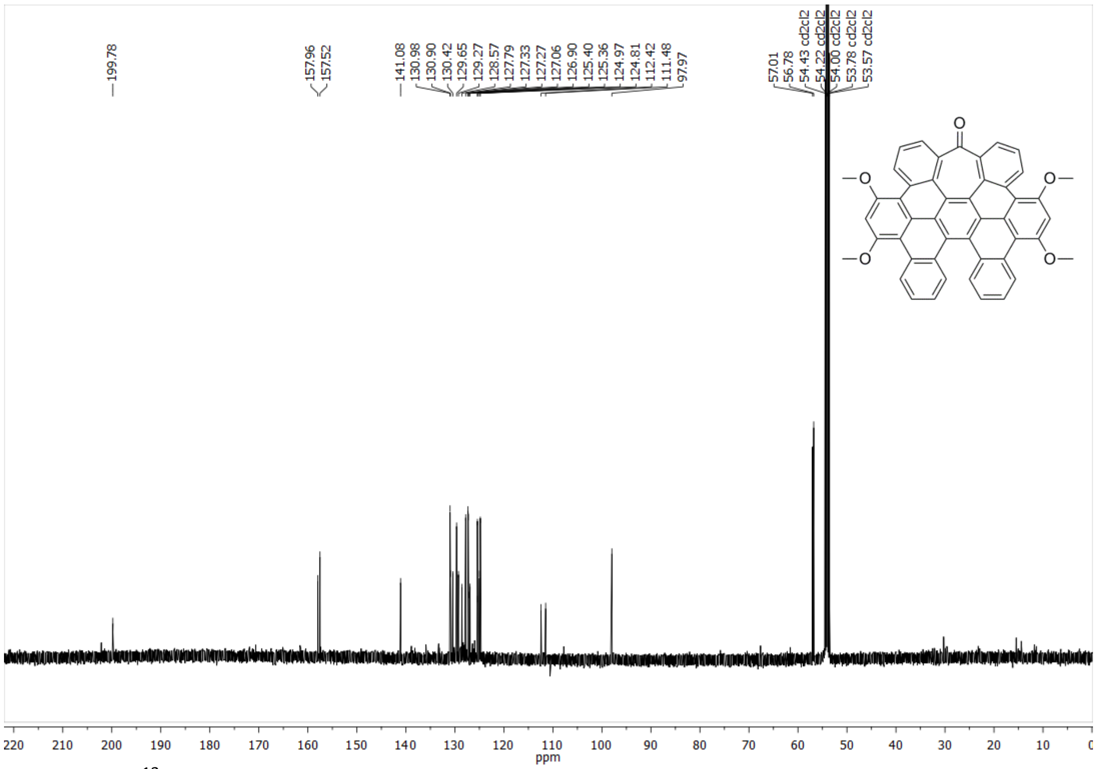

Figure S52: ^{13}C NMR spectrum of **6a** in CD_2Cl_2 (126 MHz).

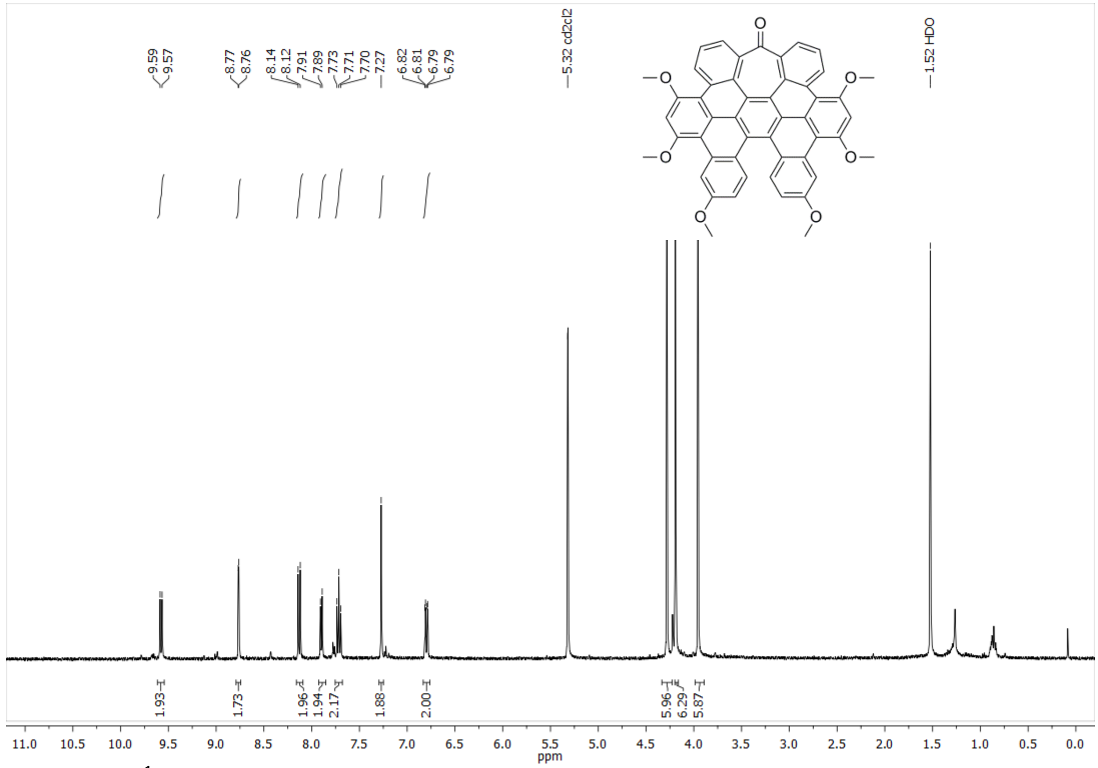

Figure S53: ^{1}H NMR spectrum of **6b** in CD_2Cl_2 (400 MHz) with residual water peak at 1.53 ppm.

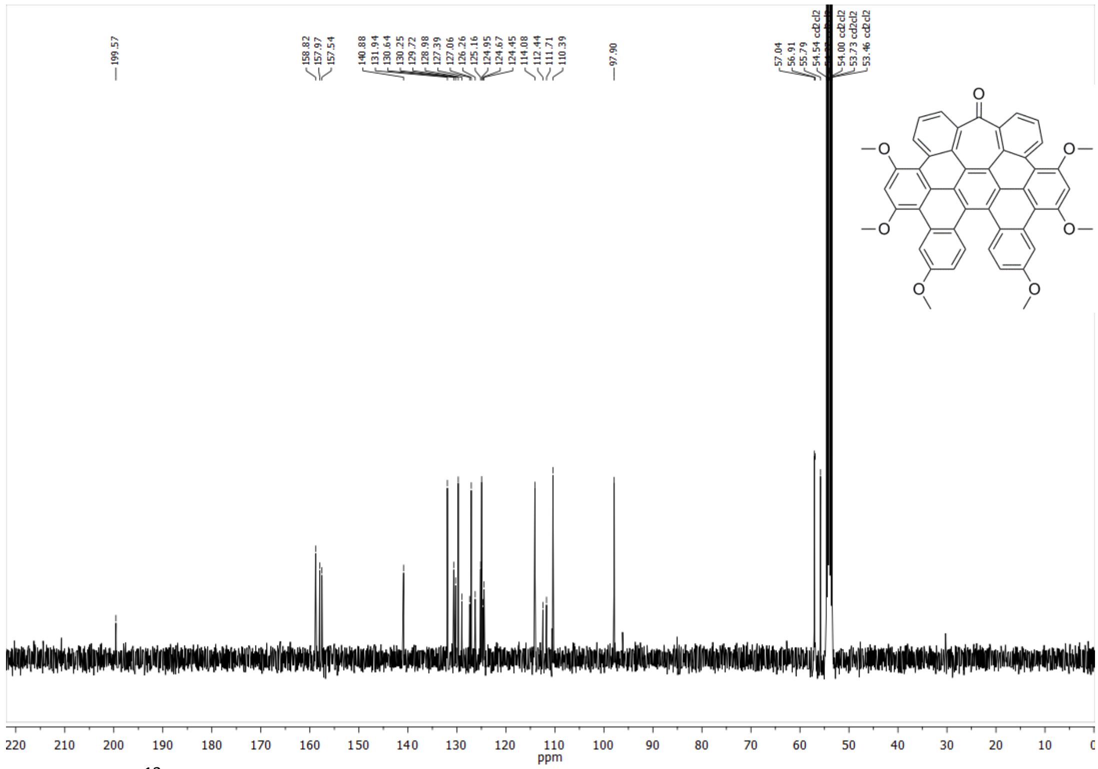

Figure S54: ^{13}C NMR spectrum of **6b** in CD_2Cl_2 (101 MHz).

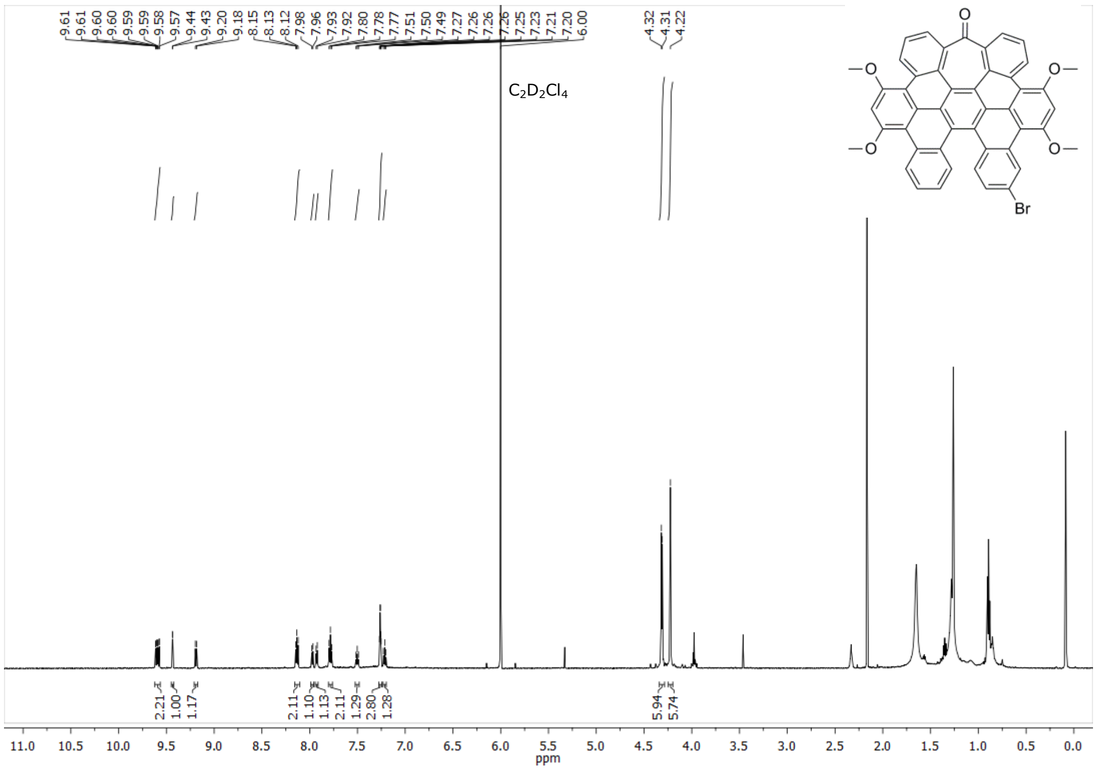

Figure S55: ^{1}H NMR spectrum of **6c** in $C_2D_2Cl_4$ (600 MHz), (Acetone at 2.06 ppm).

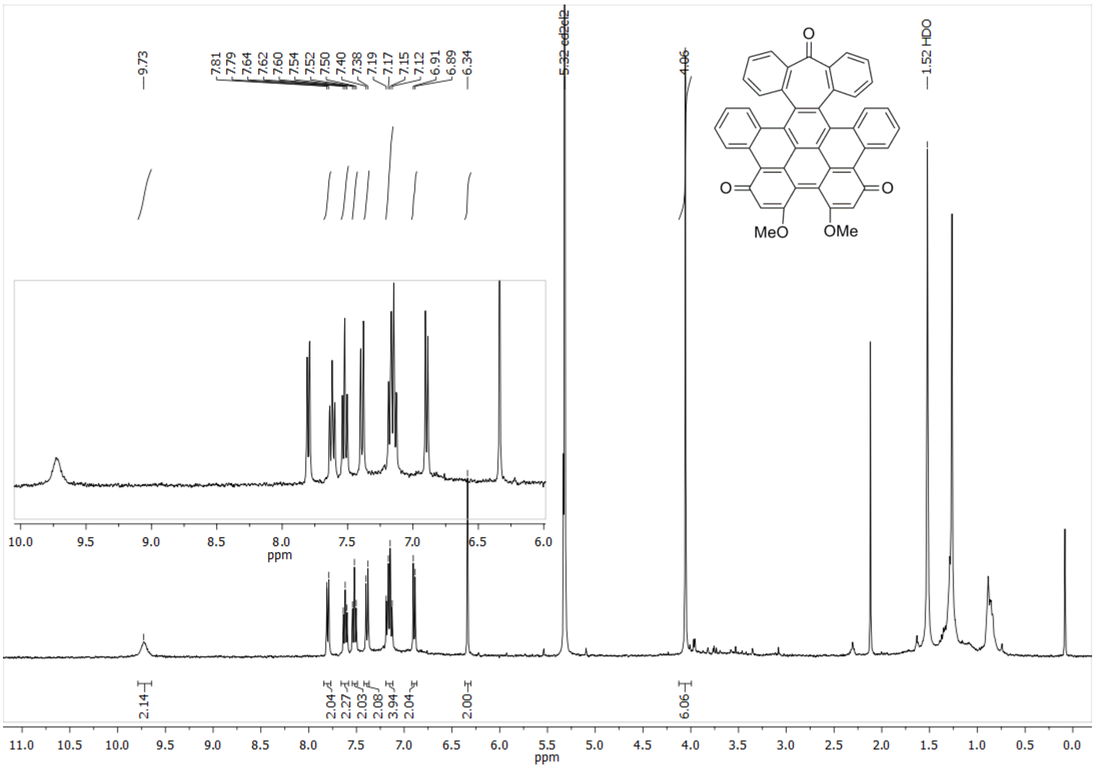

Figure S56: ^{1}H NMR spectrum of **6d** in CD_2Cl_2 (400 MHz) (Acetone at 2.12 ppm).

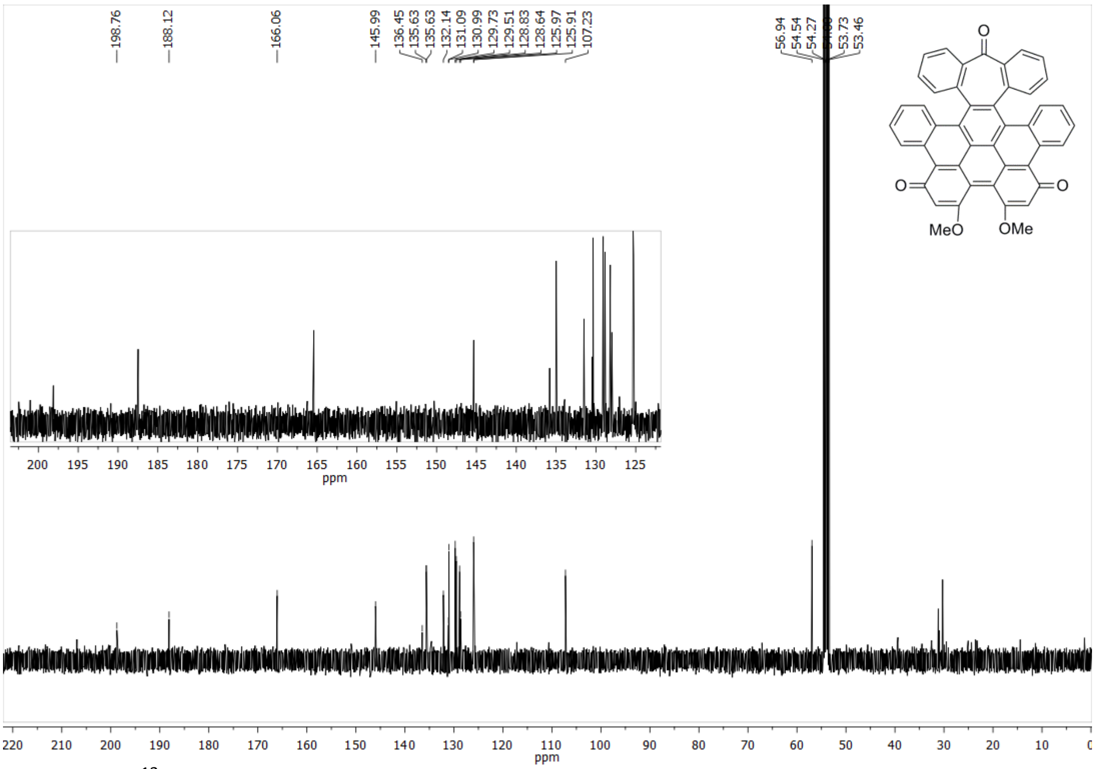

Figure S57: ^{13}C NMR spectrum of **6d** in CD_2Cl_2 (101 MHz).

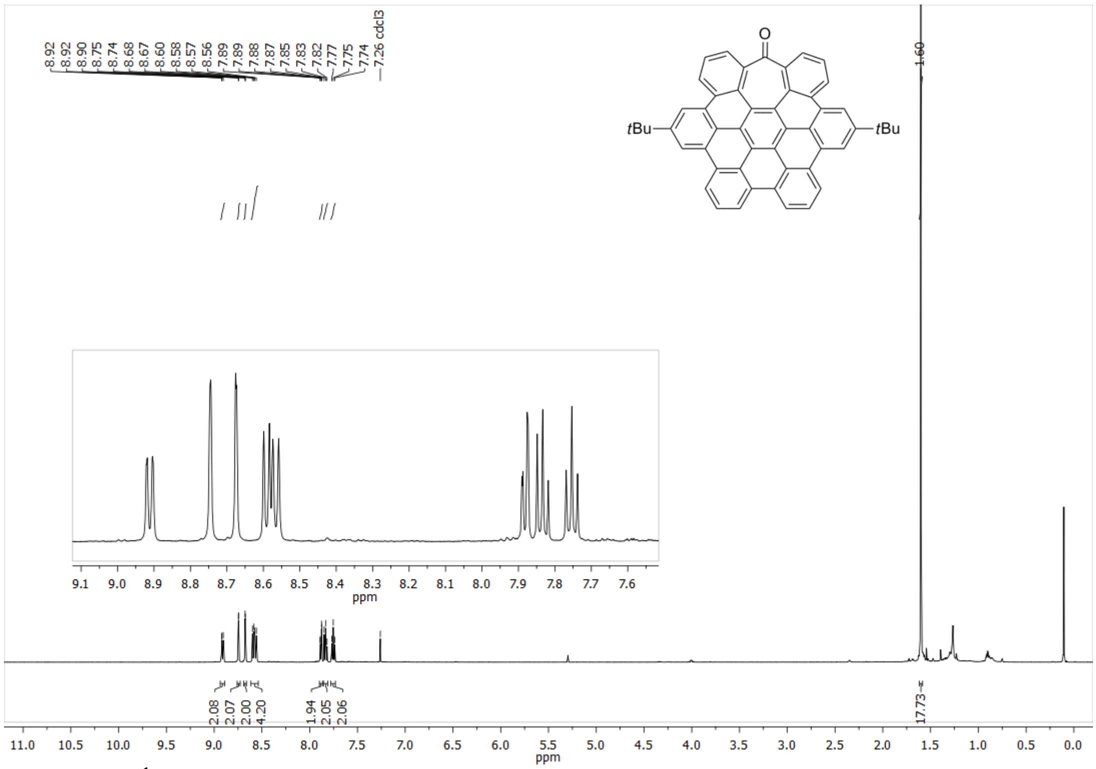

Figure S58: ^{1}H NMR spectrum of **6e** in $CDCl_3$ (500 MHz).

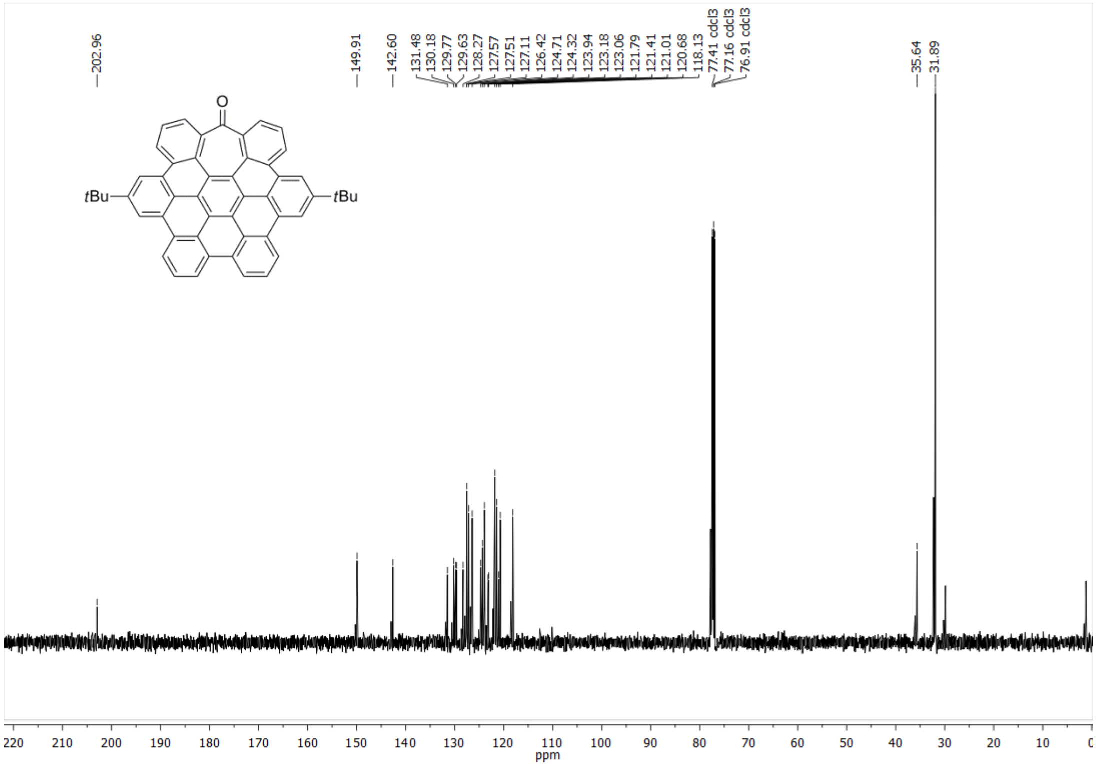

Figure S59: ^{13}C NMR spectrum of **6e** in $CDCl_3$ (126 MHz).

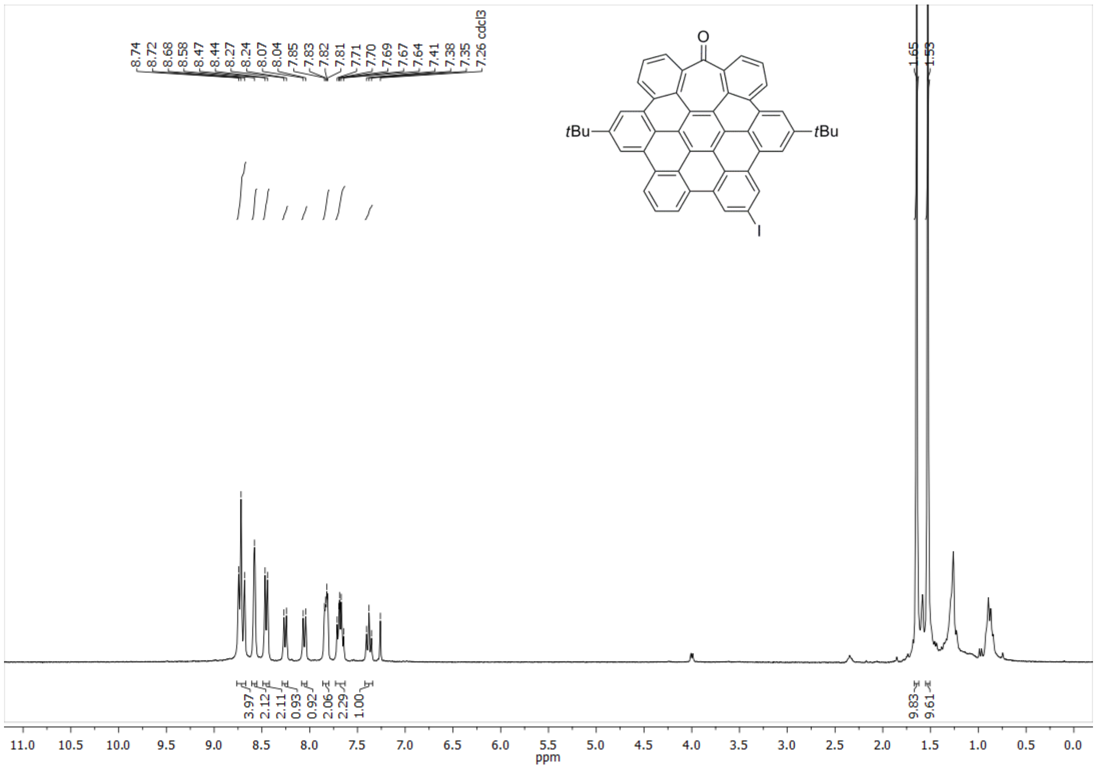

Figure S60: ^{1}H NMR spectrum of **6f** in $CDCl_3$ (400 MHz).

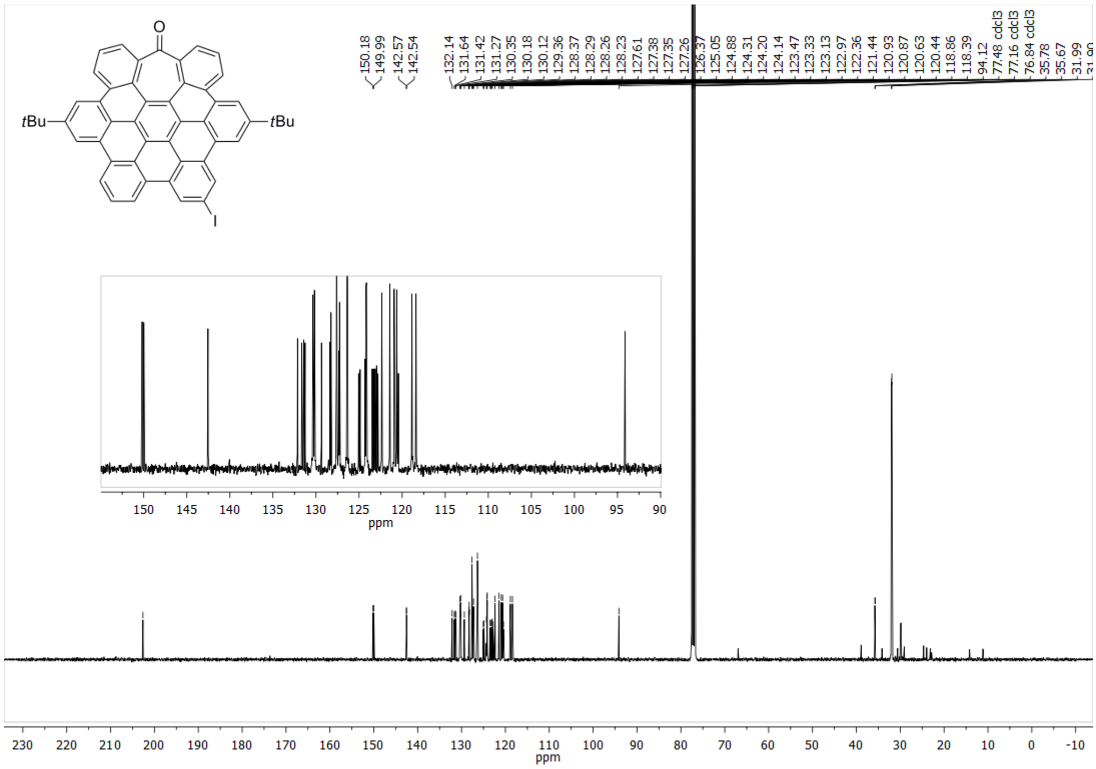

Figure S61: ^{13}C NMR spectrum of **6f** in $CDCl_3$ (101 MHz).

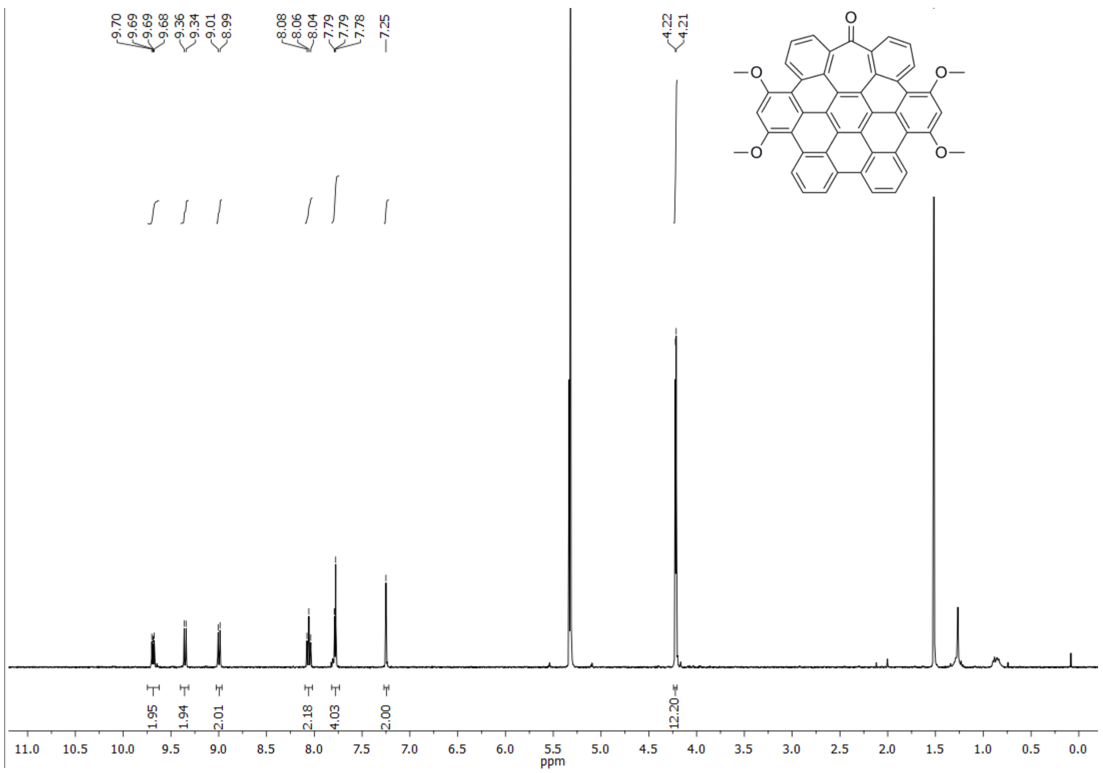

Figure S62: ^{1}H NMR spectrum of **6h** in CD_2Cl_2 (400 MHz) with residual water peak at 1.52 ppm.

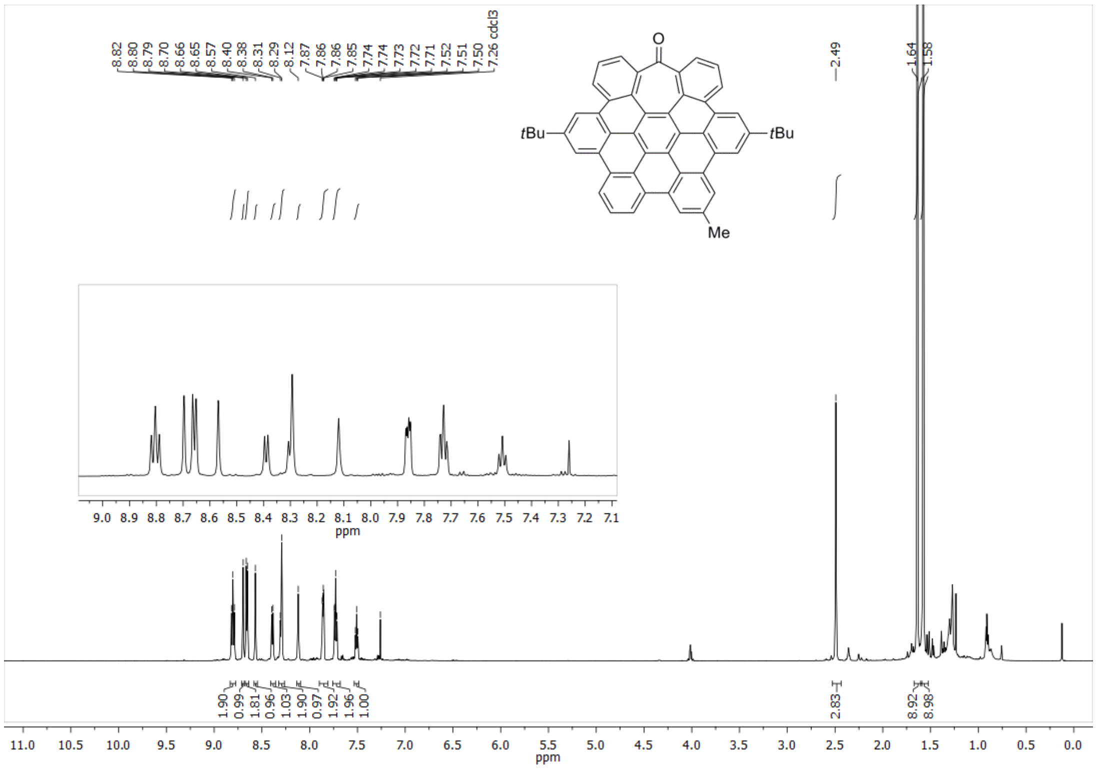

Figure S63: ^{1}H NMR spectrum of **6g** in $CDCl_3$ (600 MHz).

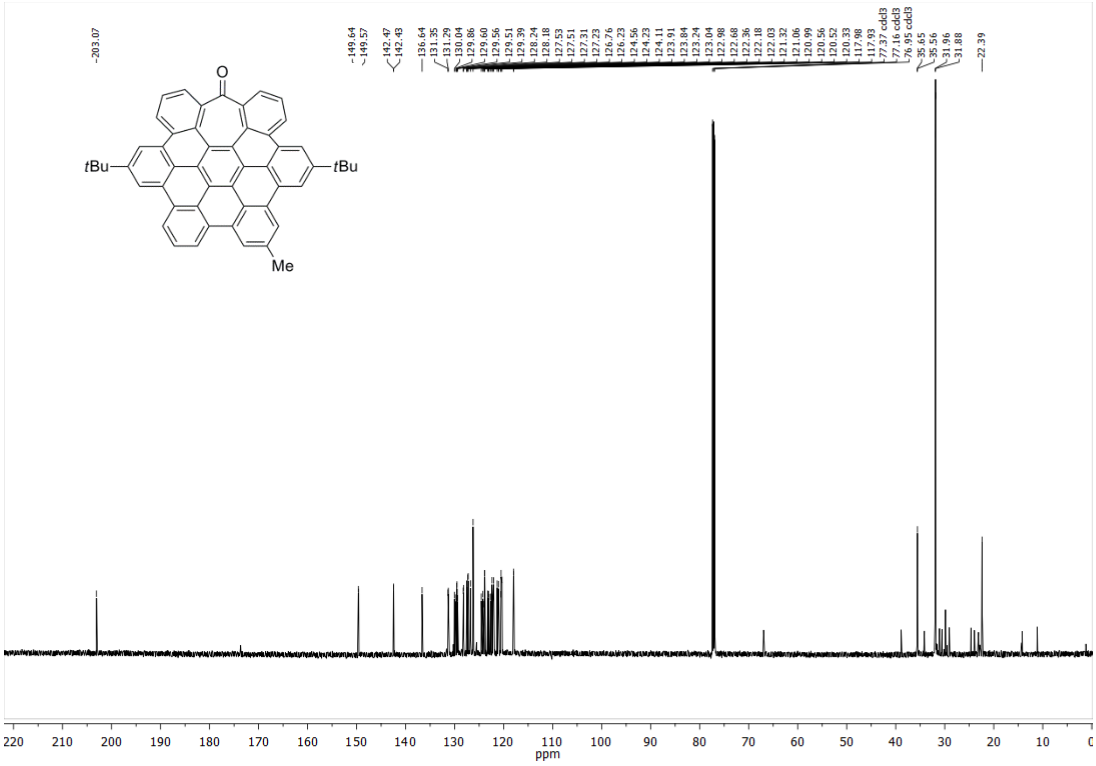

Figure S64: ^{13}C NMR spectrum of **6g** in $CDCl_3$ (151 MHz).

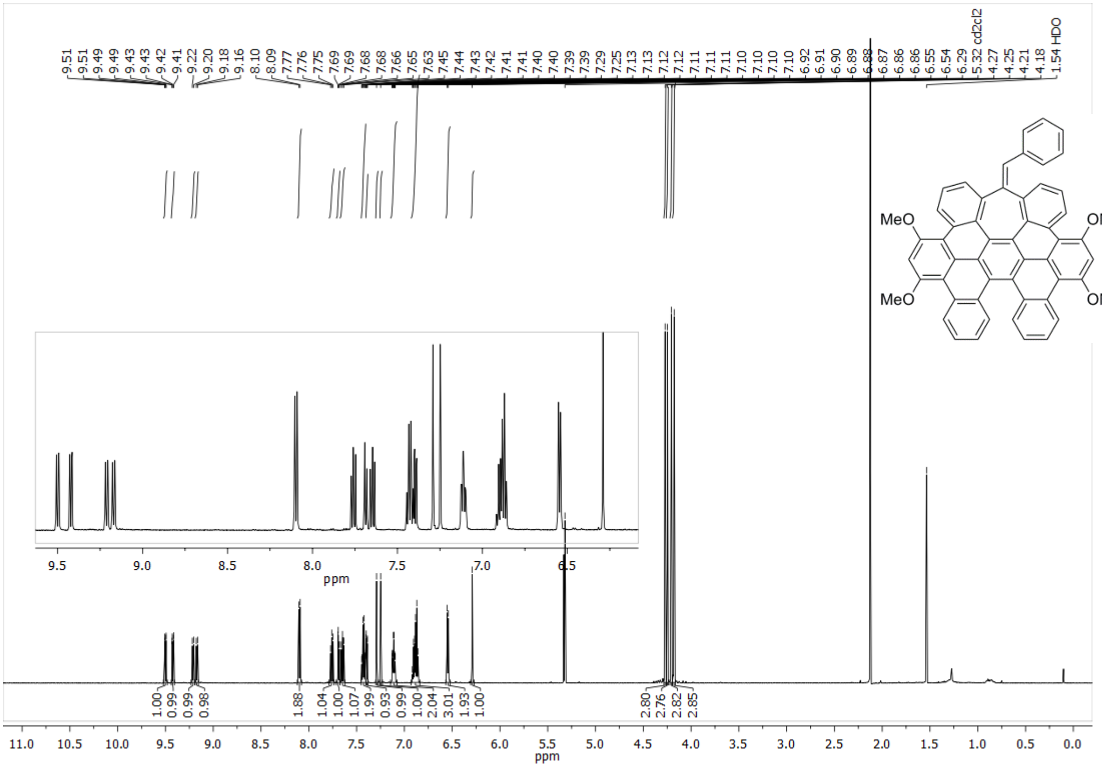

Figure S65: ^{1}H NMR spectrum of **7a** in CD_2Cl_2 (600 MHz) with traces of acetone at 2.12 ppm.

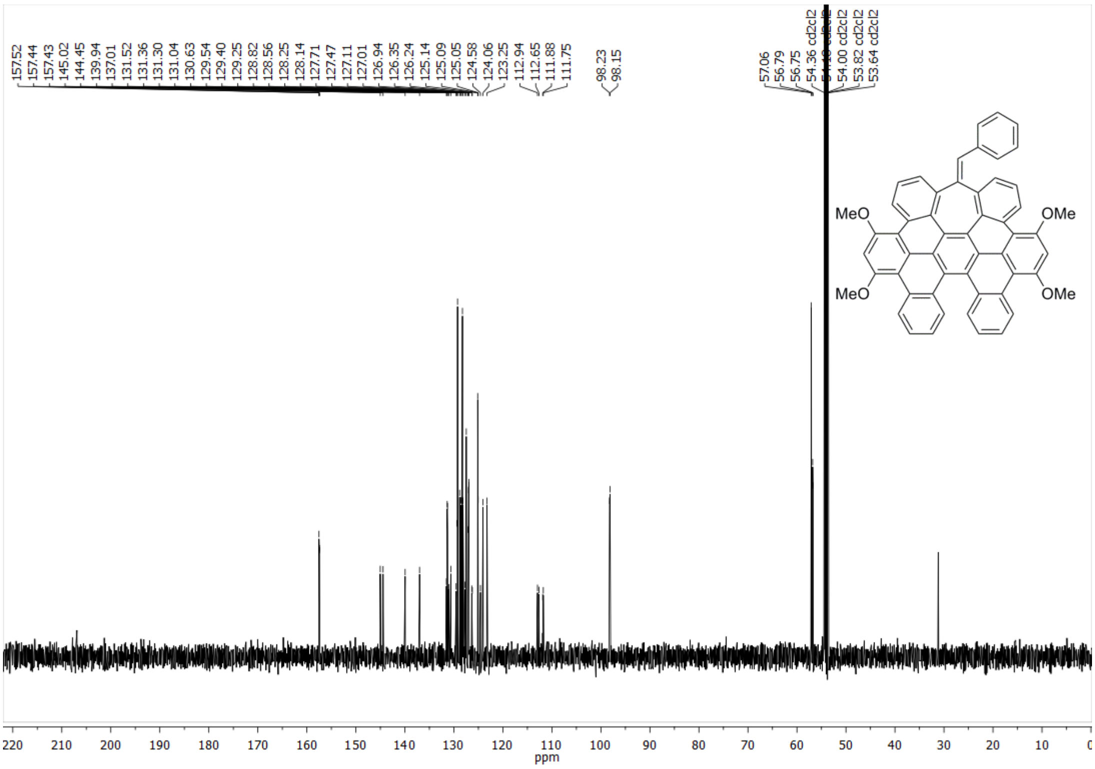

Figure S66: ^{13}C NMR spectrum of **7a** in CD_2Cl_2 (151 MHz).

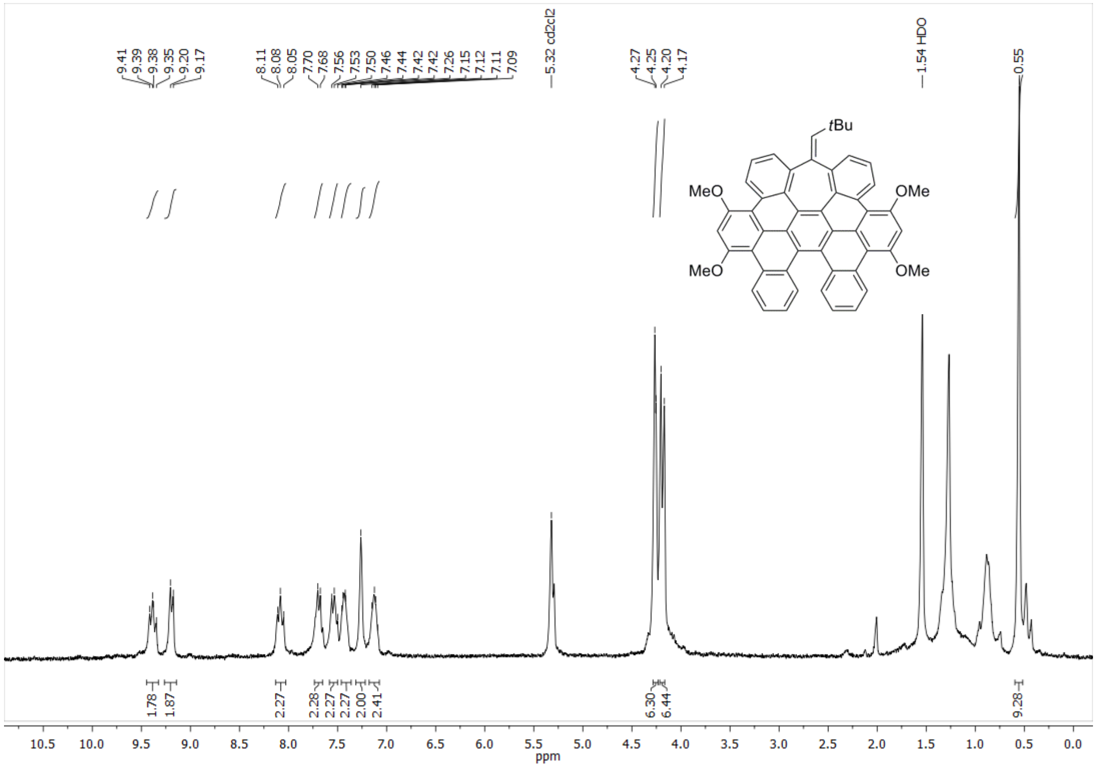

Figure S67: ^{1}H NMR spectrum of **7b** in CD_2Cl_2 (300 MHz).

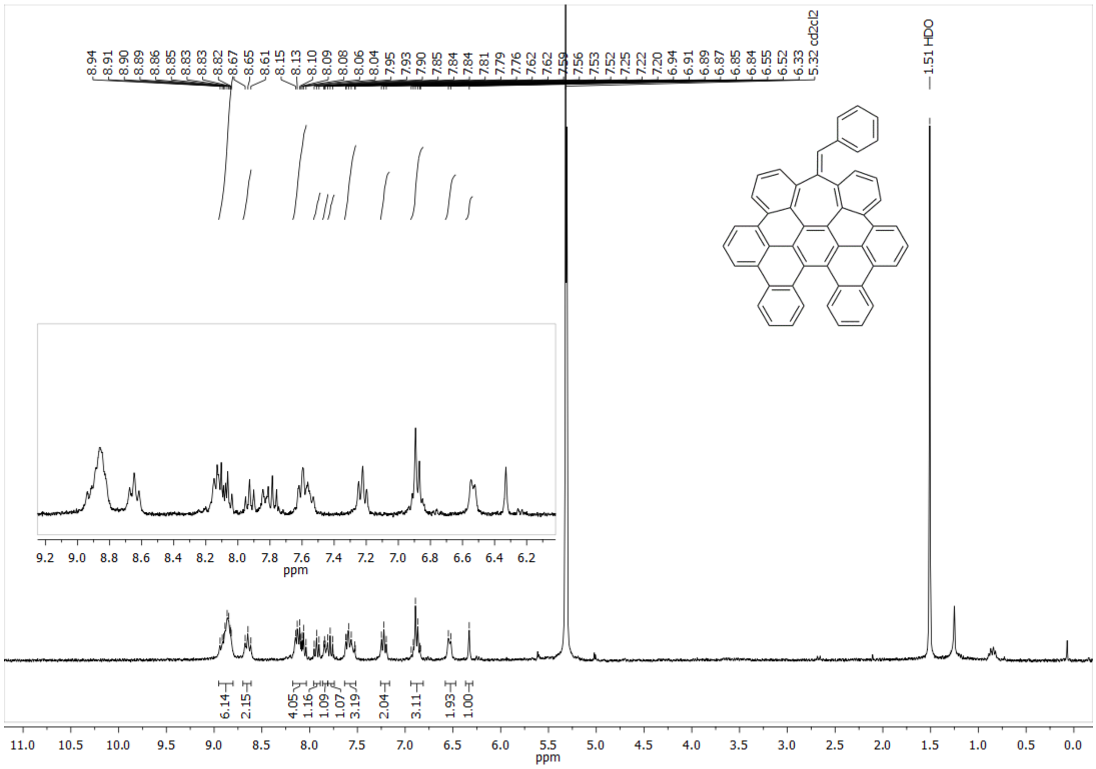

Figure S68: ^{1}H NMR spectrum of **8a** in CD_2Cl_2 (300 MHz).

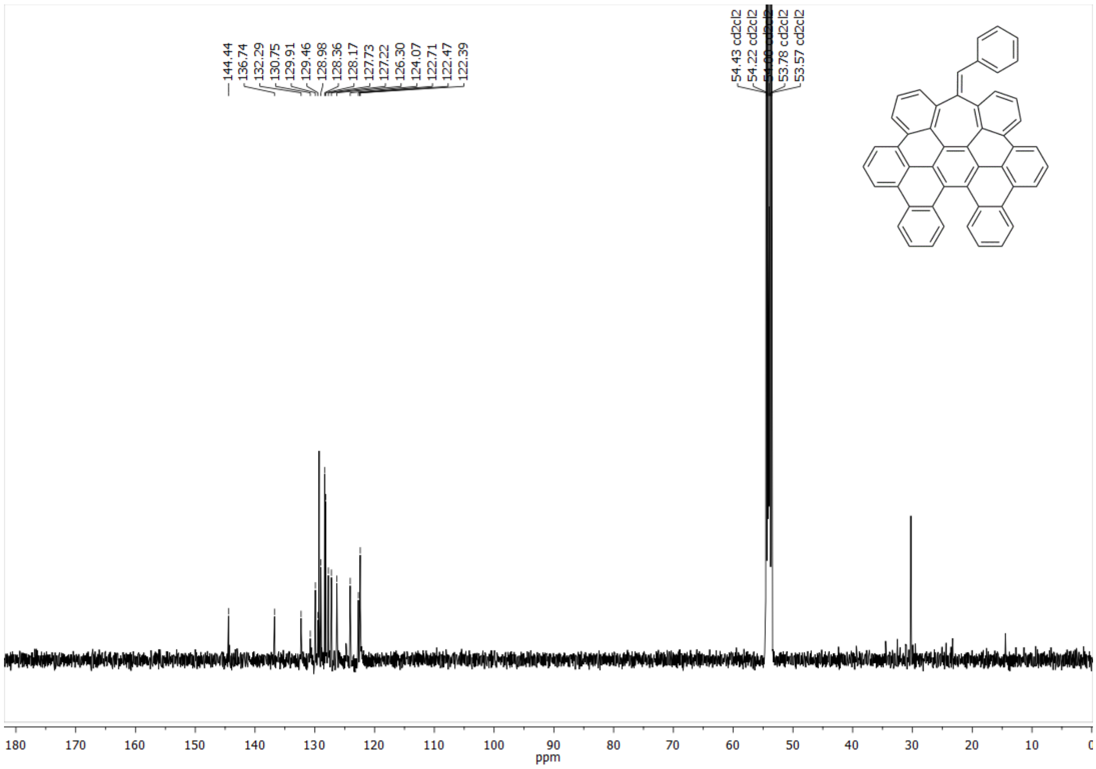

Figure S69: ^{13}C NMR spectrum of **8a** in CD_2Cl_2 (126 MHz).

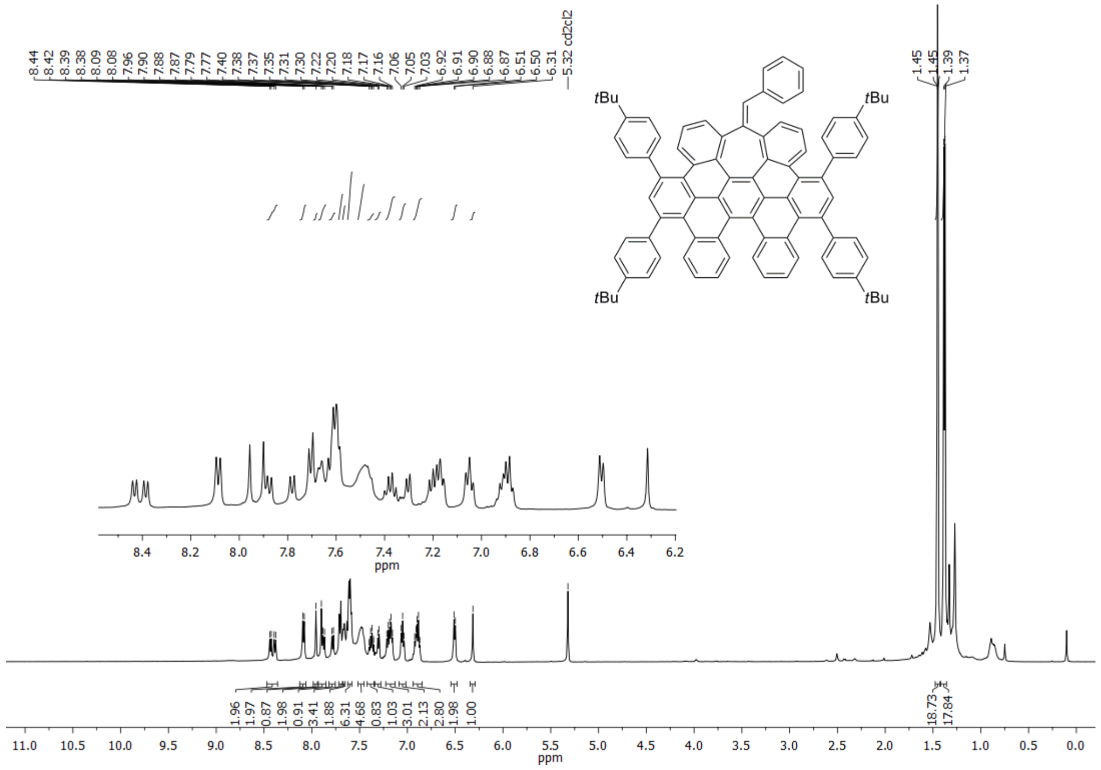

Figure S70: ^{1}H NMR spectrum of **9a** in CD_2Cl_2 (500 MHz).

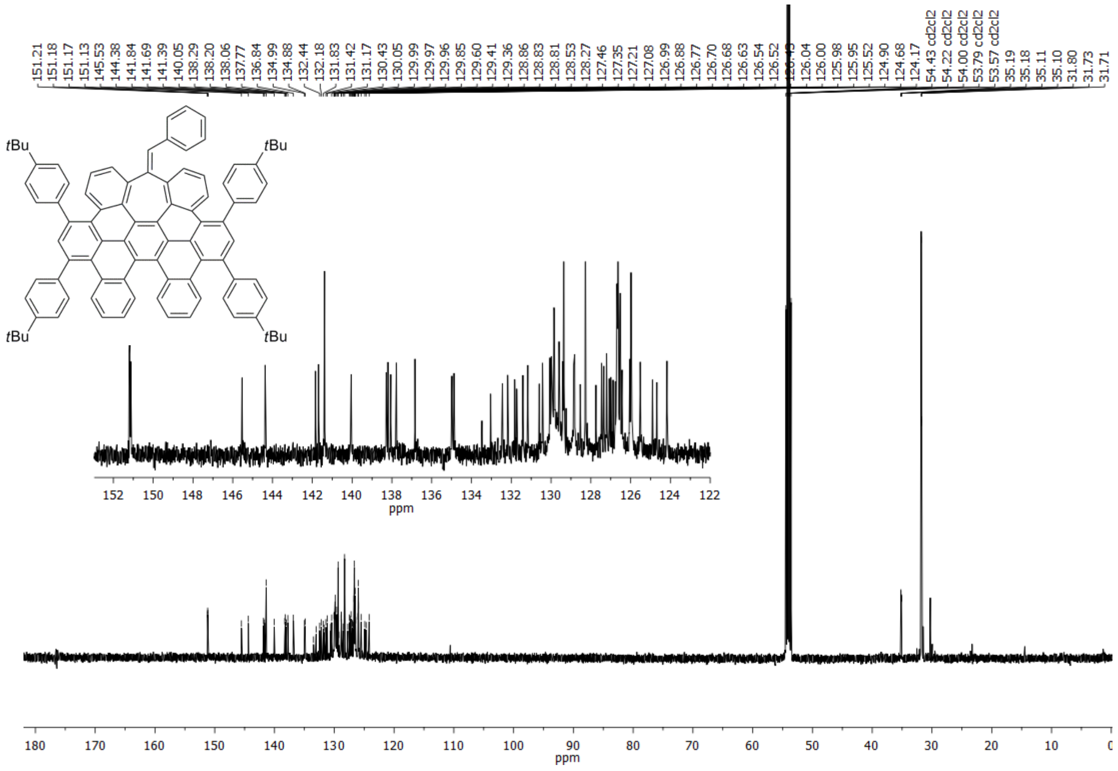

Figure S71: ^{13}C NMR spectrum of **9a** in CD_2Cl_2 (126 MHz).

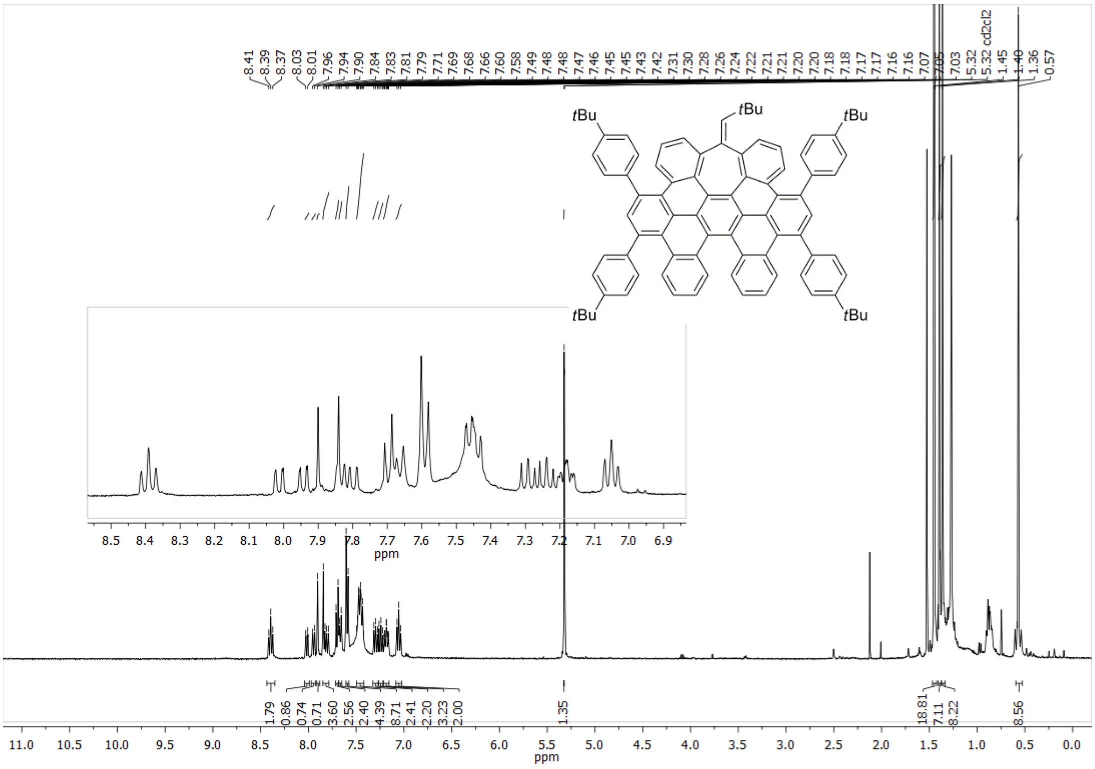

Figure S72: ^{1}H NMR spectrum of **9b** in CD_2Cl_2 (400 MHz).

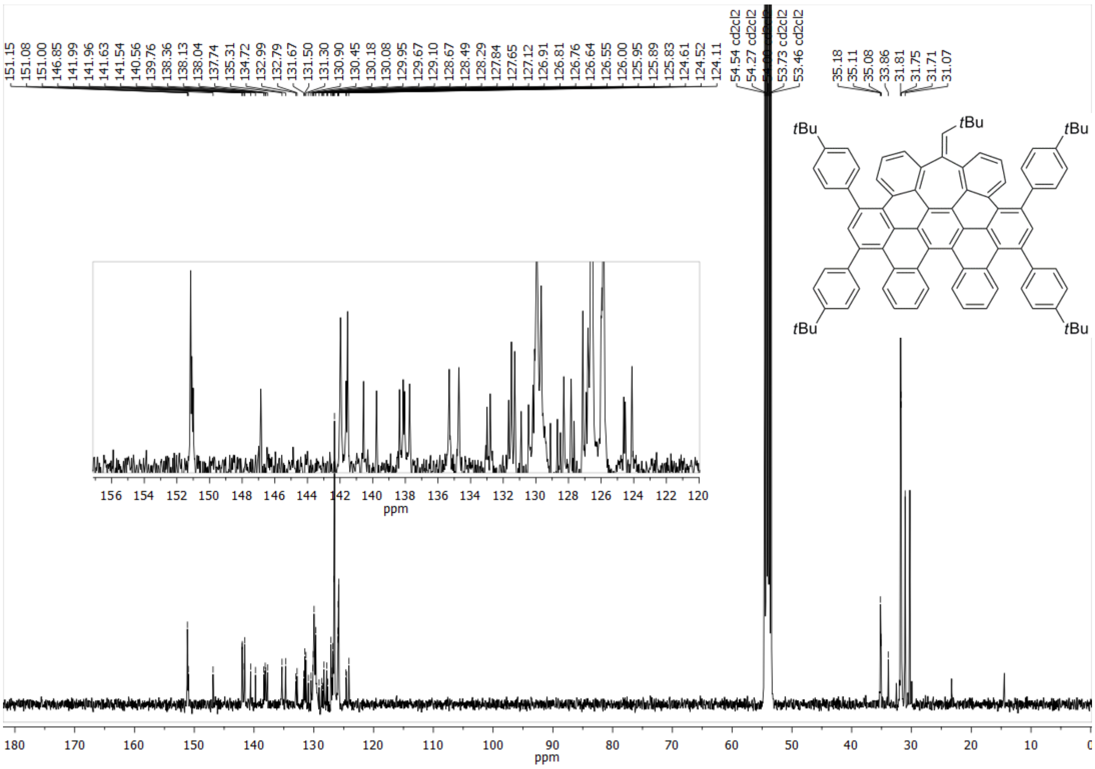

Figure S73: ^{13}C NMR spectrum of **9b** in CD_2Cl_2 (101 MHz).

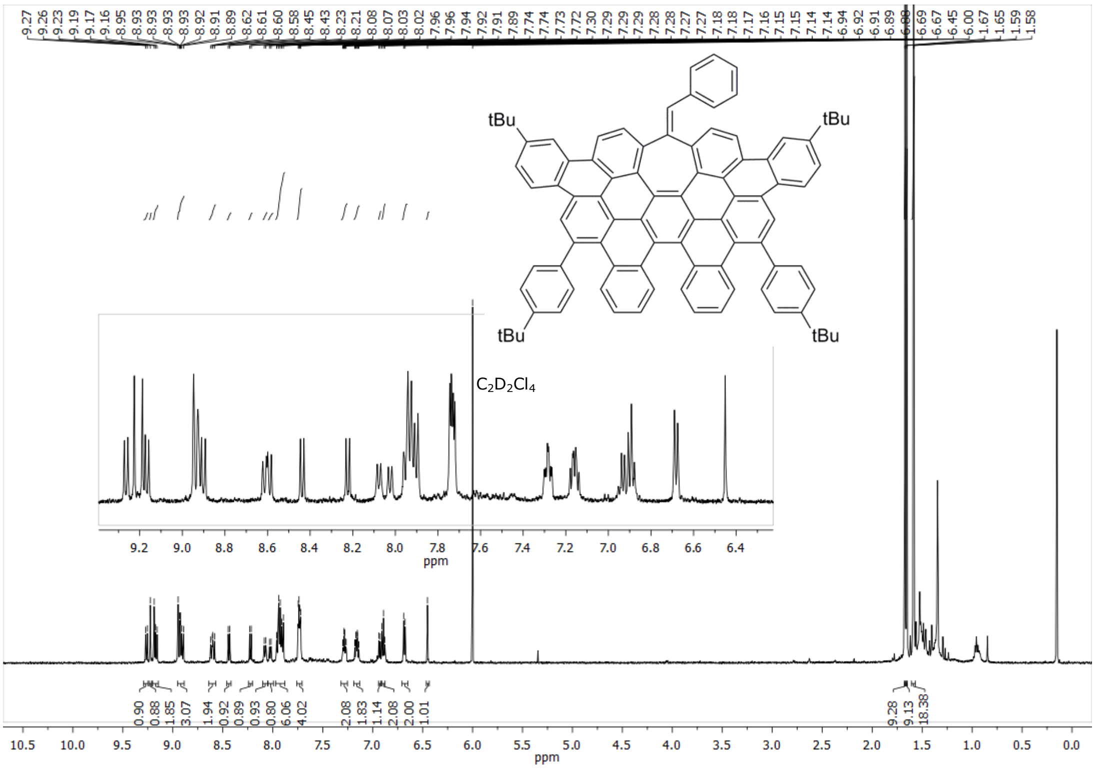

Figure S74: ^{1}H NMR spectrum of **1** in $C_2D_2Cl_4$ (500 MHz, 369K).

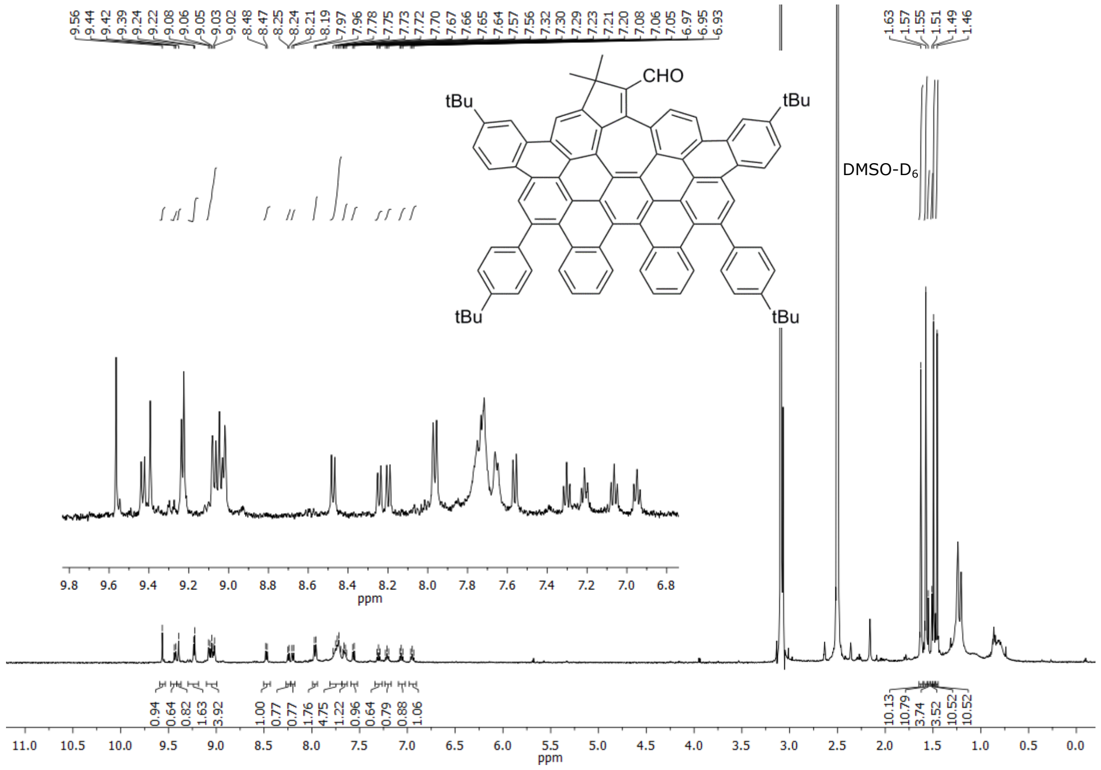

Figure S75: ^{1}H NMR spectrum of **2** in DMSO-d_6 (500 MHz, 348 K).

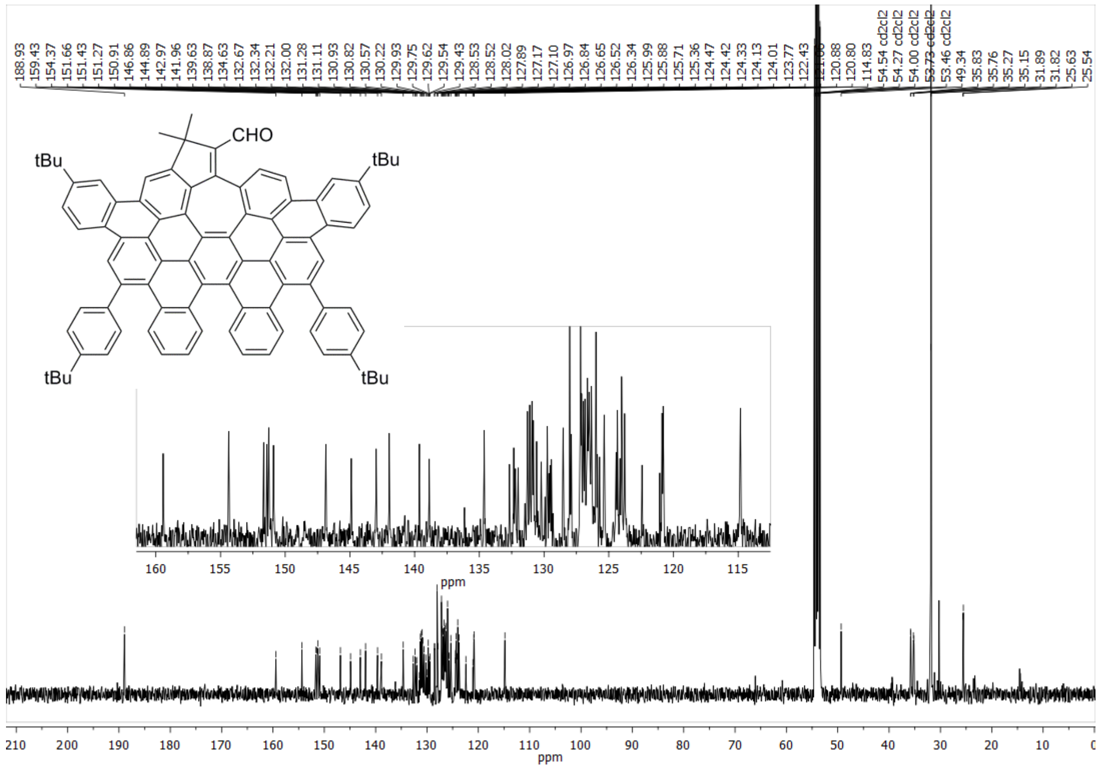

Figure S76: ^{13}C NMR spectrum of **2** in CD_2Cl_2 (101 MHz).

8. Copies of VT-NMR, 2D-NMR and HRMS-MALDI spectra of 1 and 2

Compound 1

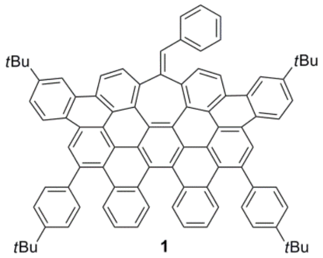

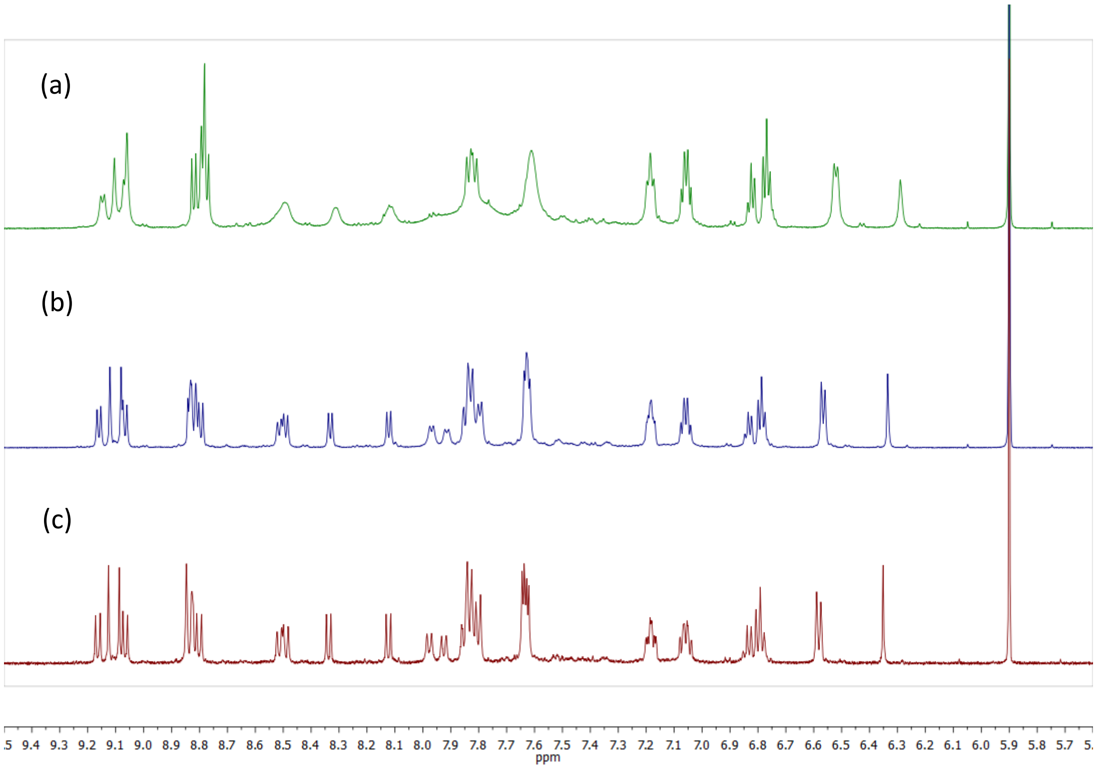

Figure S77: Partial ^{1}H NMR spectra (500 MHz, $C_2D_2Cl_4$) of compound **1** at: (a) 293K (green); (b) 348K (blue); (c) 369K (red).

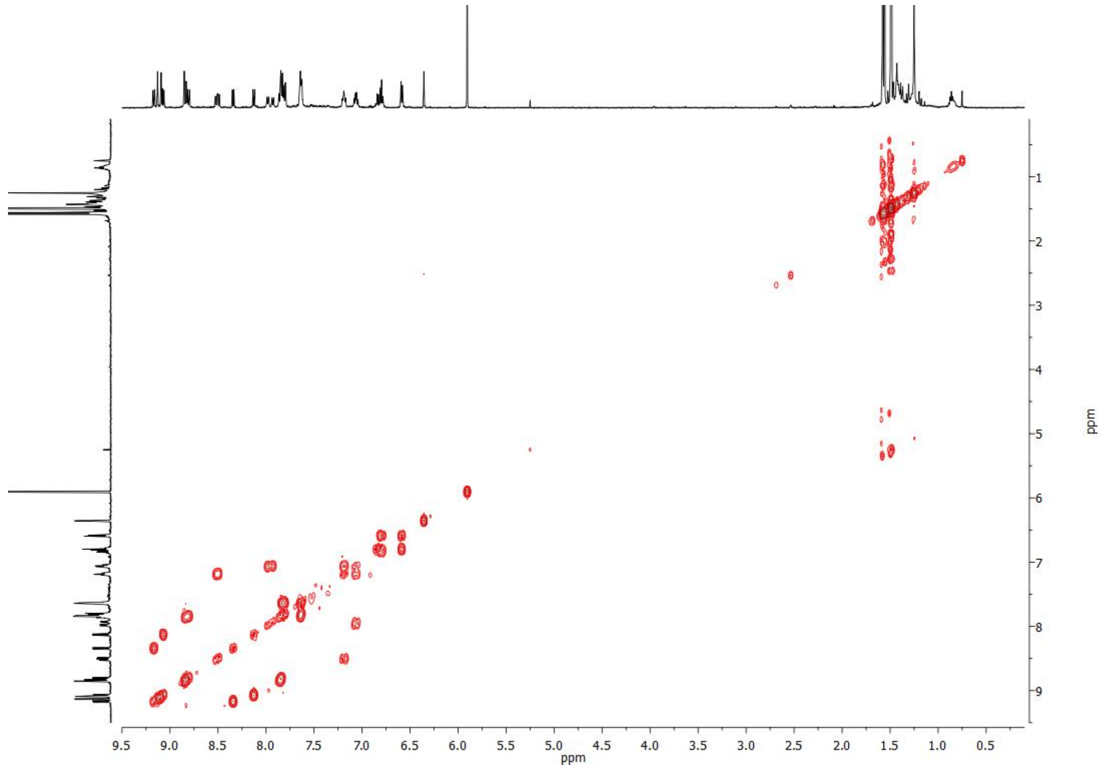

Figure S78: ^{1}H COSY NMR spectra in $C_2D_2Cl_4$ (500 MHz, 369K) of compound **1**.

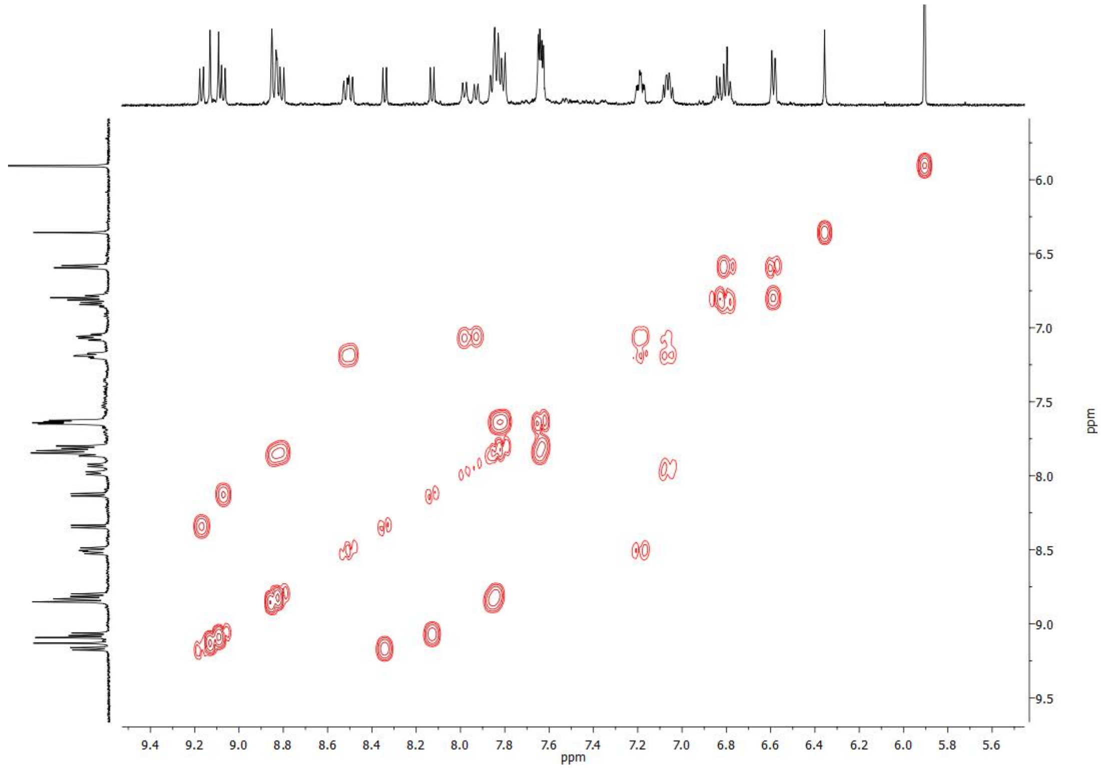

Figure S79: Partial ^{1}H COSY NMR spectra in $C_2D_2Cl_4$ (500 MHz, 369K) of compound **1**.

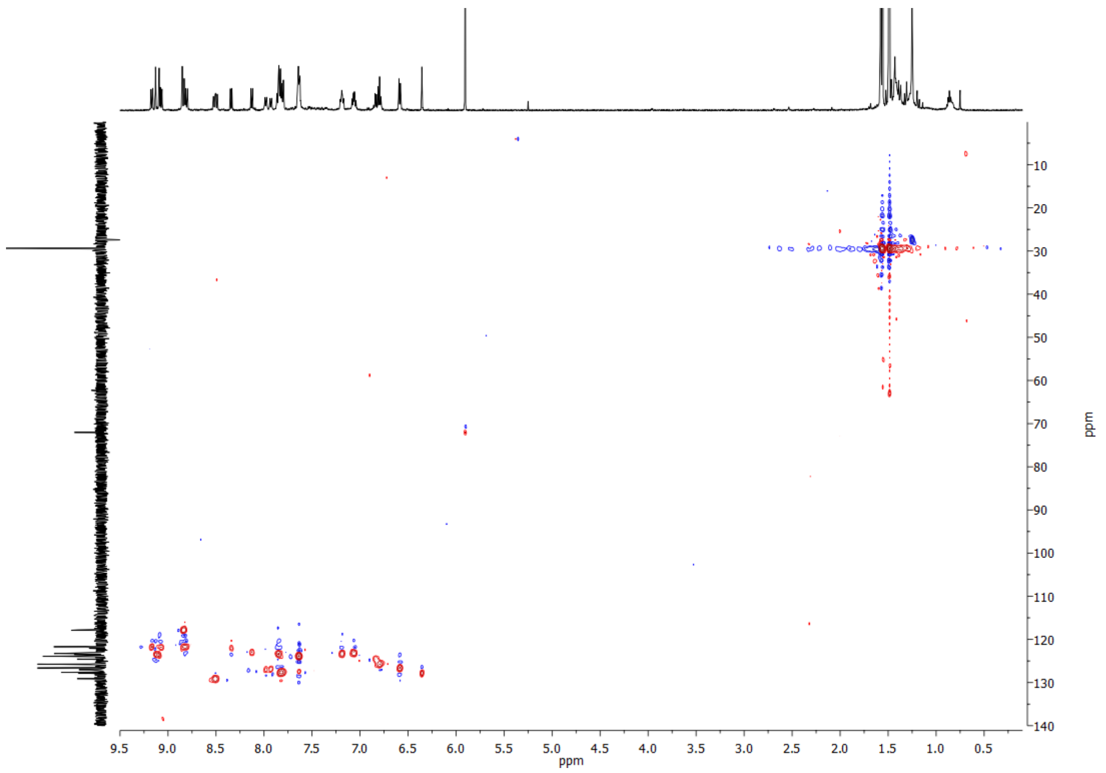

Figure S80: [13]HSQC NMR spectra in $C_2D_2Cl_4$ (500 MHz, 369K) of compound **1**.

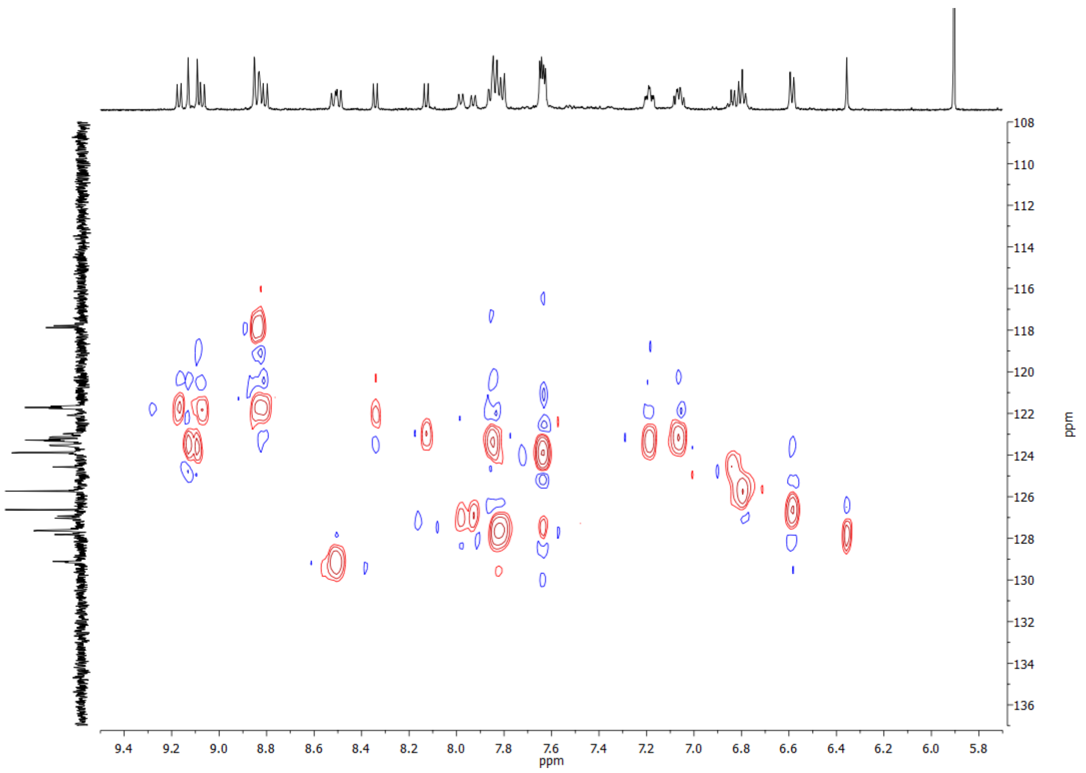

Figure S81: Partial HSQC NMR spectra in $C_2D_2Cl_4$ (500 MHz, 369K) of compound **1**.

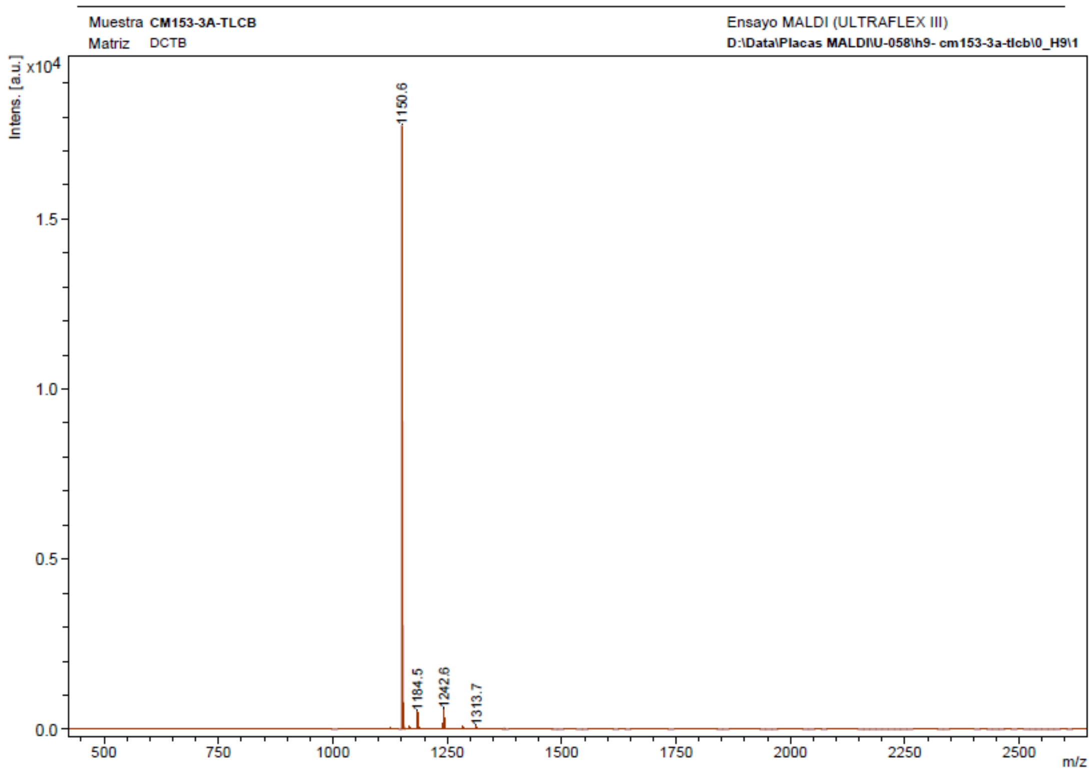

Figure S82: MS (MALDI, DCTB) spectrum of **1** $[M]^{+}$.

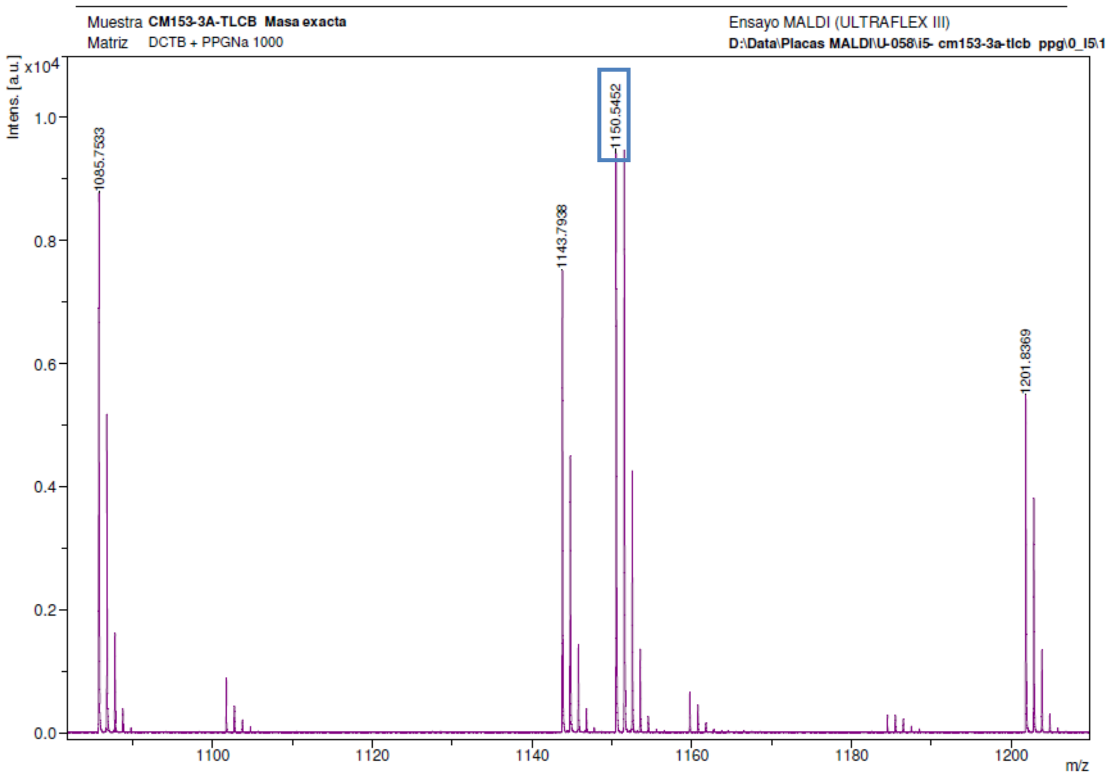

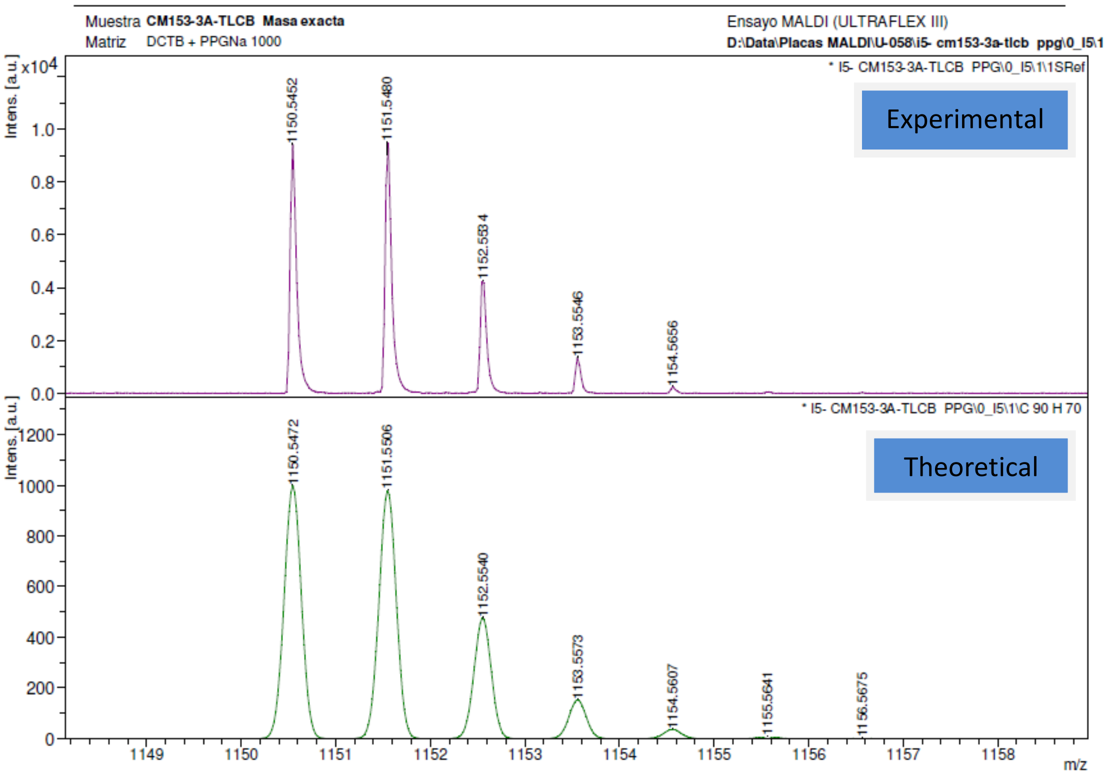

Figure S83: HRMS (MALDI, DCTB) spectrum of **1** $[M]^+$ and experimental *vs* theoretical isotopic distribution.

Compound 2

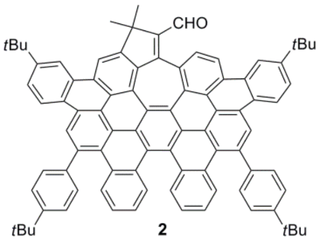

2

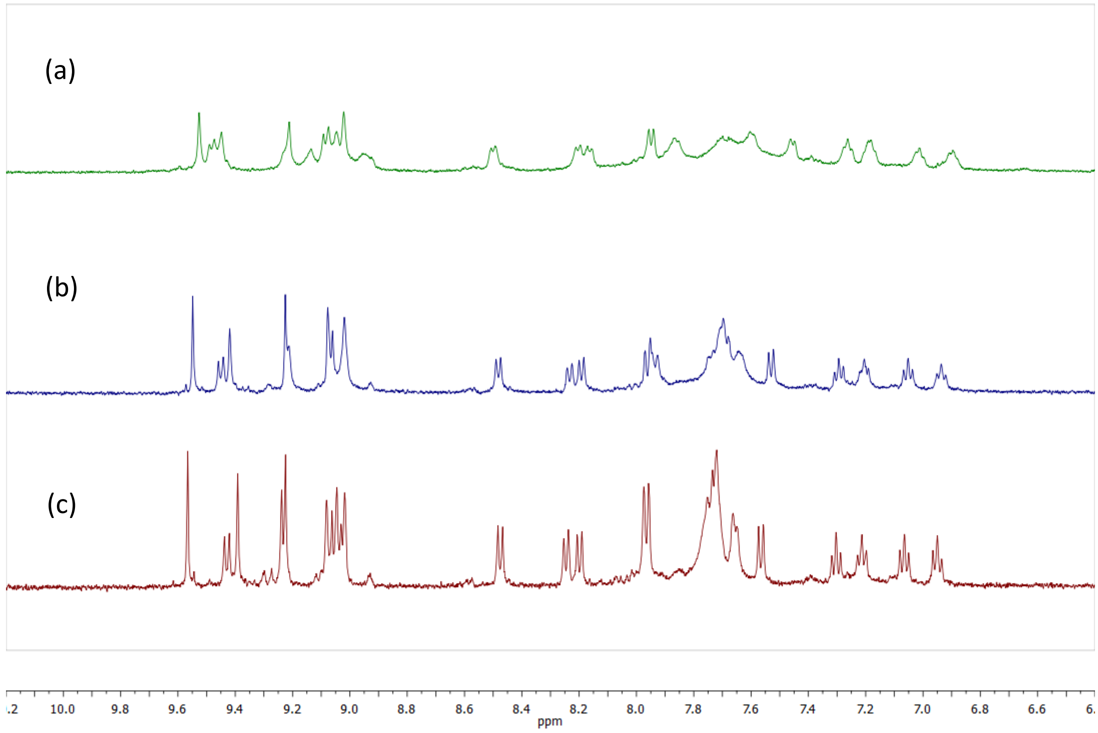

Figure S84: Partial ^{1}H NMR spectra (500 MHz, DMSO-d$_6$) of compound **2** at**:** (a) 293K (green); (b) 328K (blue); (c) 348K (red).

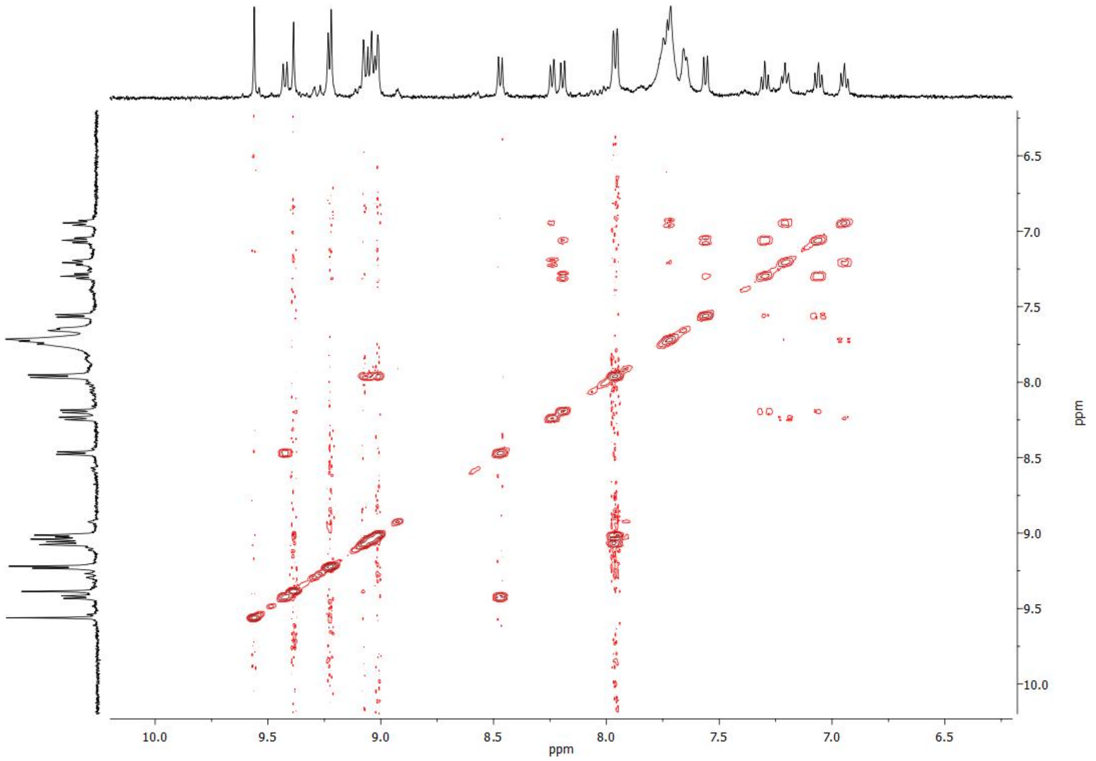

Figure S85: Partial ^{1}H COSY NMR spectra in DMSO-d$_6$ (500 MHz, 348K) of compound **2**.

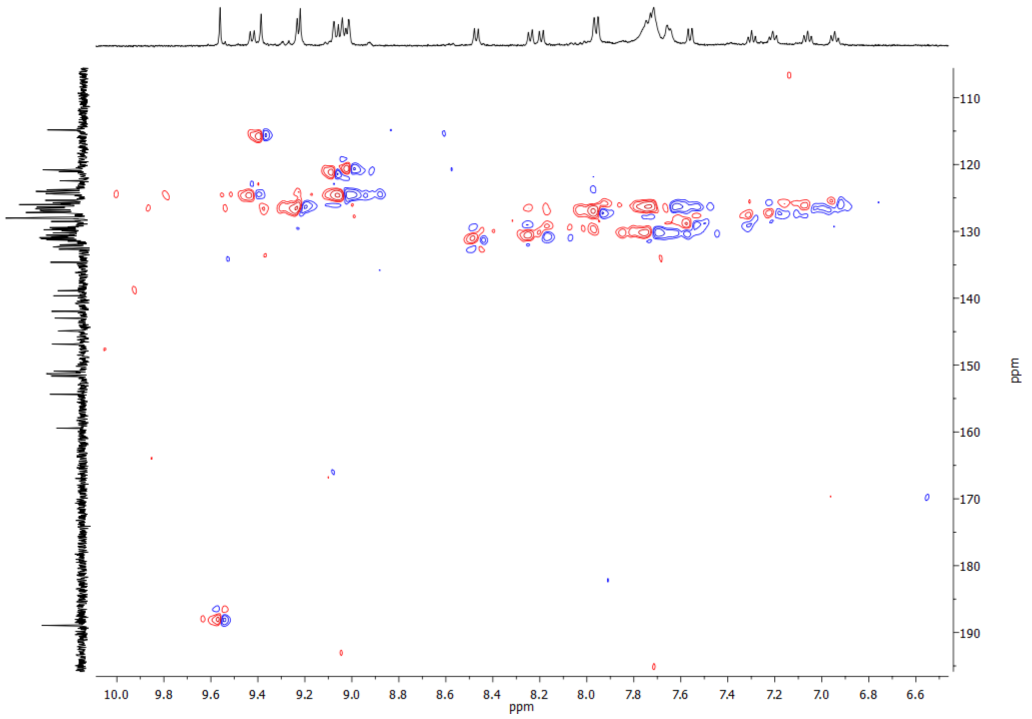

Figure S86: Partial HSQC NMR spectra in DMSO-d$_6$ (500 MHz, 348K) of compound **2**.

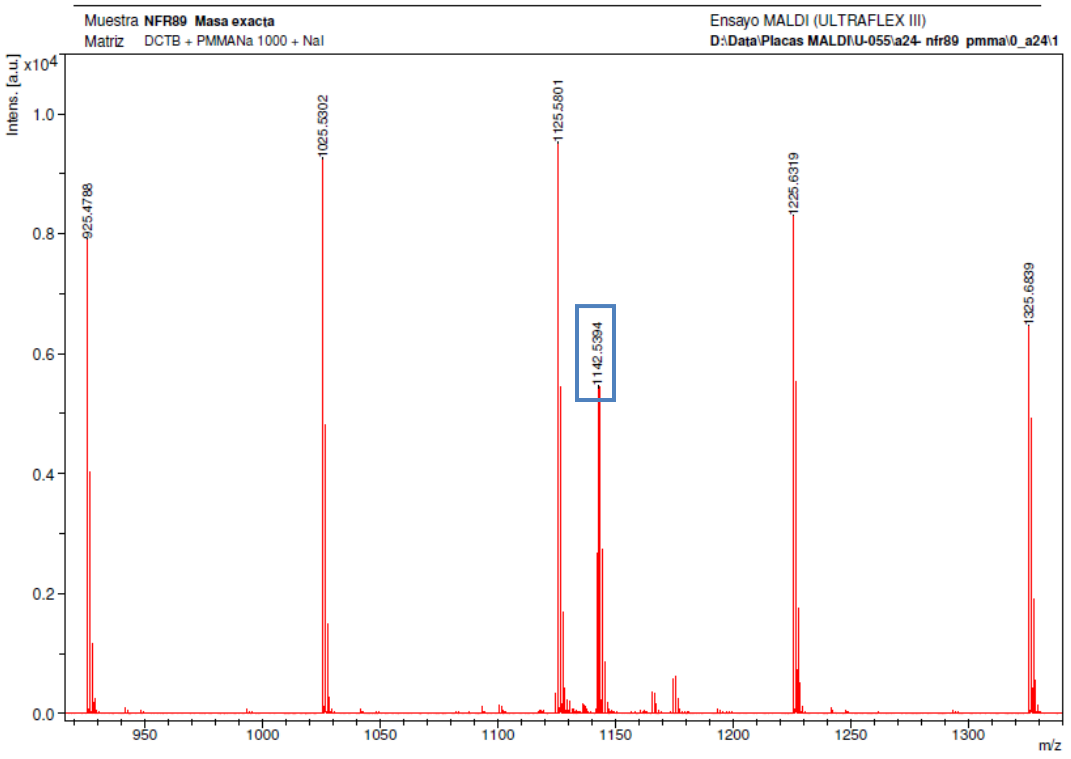

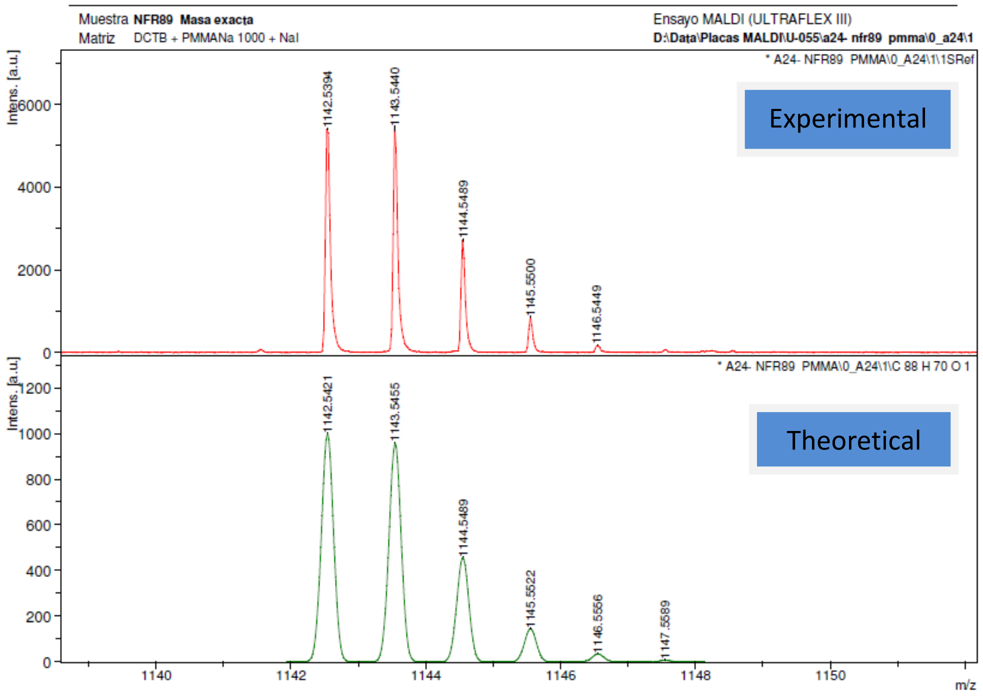

Figure S87: HRMS (MALDI, DCTB) spectrum of **2** $[M]^+$ and experimental *vs* theoretical isotopic distribution.

9. Literature

[S1] D. Shi, J. Li, B. Jiang, S. Guo, H. Su and T. Wang, *Bioorg. Med. Chem. Lett.*, 2012, **22**, 2827-2832.

[S2] M. J. Mio, L. C. Kopel, J. B. Braun, T. L. Gadzikwa, K. L. Hull, R. G. Brisbois, C. J. Markworth and P. A. Grieco, *Org. Lett.*, 2002, **4**, 3199-3202.

[S3] S. Kim, J. Rojas-Martin and F. D. Toste, *Chem. Sci.*, 2016, **7**, 85-88.

[S4] Y. Ji, H. Khaizourane, A. N. Wein, X. Verdaguer and A. Riera, *Eur. J. Org. Chem.*, 2012, 6058-6063.

[S5] C. F. Xu, M. Xu, Y. X. Jia and C. Y. Li, *Org. Lett.*, 2011, **13**, 1556-1559.

[S6] S. Tartaggia, O. De Lucchi and L. J. Gooßen, *Eur. J. Org. Chem.*, 2012, 1431-1438.

[S7] A. Sagadevan and K. C. Hwang, *Adv. Synth. Catal.*, 2012, **354**, 3421-3427.

[S8] J. M. Tour, a. M. Rawlett, M. Kozaki, Y. Yao, R. C. Jagessar, S. M. Dirk, D. W. Price, M. a. Reed, C. W. Zhou, J. Chen, W. Wang and I. Campbell, *Chem. Eur. J.*, 2001, **7**, 5118-5134.

[S9] M. Planellas, Y. Moglie, F. Alonso, M. Yus, R. Pleixats and A. Shafir, *Eur. J. Org. Chem.*, 2014,3001-3008.

[S10] J. Q. Umberger and V. K. LaMer, *J. Am. Chem. Soc.*, 1945, **67**, 1099-1109.

[S11] W. H. Melhuish, *J. Phys. Chem.*, 1961, **65**, 229-235.

[S12] G. M. Sheldrick, *ActaCrystallogr. Sect. A: Found. Crystallogr.*, 2007, **64**, 112-122.

[S13] O. V. Dolomanov, L. J. Bourhis, R. J. Gildea, J. A. K. Howard and H. Puschmann, *J. Appl. Crystallogr.*, 2009, **42**, 339-341.

[S14] J. M. Soler, E. Artacho, J. D. Gale, A. Garcìa, J. Junquera, P. Ordejón and D. Sánchez-Portal, *J. Phys.: Condens. Matter*, 2002, **14**, 2745-2779.

[S15] M. Dion, H. Rydberg, E. Schröder, D. C. Langreth and B. I. Lundqvist, *Phys. Rev. Lett.*, 2004,**92**, 246401-246401.

[S16] G. Román-Pérez and J. M. Soler, *Phys. Rev. Lett.*, 2009, **103**, 1-4.

[S17] J. Klimeš, D. R. Bowler and A. Michaelides, *J. Phys.: Condens. Matter*, 2010, **22**, 022201.

[S18] M. Brandbyge, J.-L. Mozos, P. Ordejón, J. Taylor and K. Stokbro, *Phys. Rev. B*, 2002, **65**, 165401.

[S19] (a) C. Lee, W. Yang and R. G. Parr, *Phys. Rev. B*, 1988, **37**, 785-789; (b) A. D. Becke, *J. Chem. Phys.*, 1993, **98**, 5648-5652; (c) W. Kohn, a. D. Becke and R. G. Parr, *J. Phys. Chem.*, 1996, **0**, 12974-12980.

[S20] M. J. Frisch, G. W. Trucks, H. B. Schlegel, G. E. Scuseria, M. A. Robb, J. R. Cheeseman, G. Scalmani, V. Barone, B. Menuci, G. A. Petersson, H. Nakatsuji, M. Caricato, X. Li, H. P. Hratchian, A. F. Izmaylov, J. Bloino, G. Zheng, J. L. Sonnenberg, M. Hada, M. Ehara, K. Toyota, R. Fukuda, J. Hasegawa, M. Ishida, T. Nakajima, Y. Honda, O. Kitao, H. Nakai, T. Vreven, J. A. Jr. Montgomery, J. E. Peralta, F. Ogliaro, M. Bearpark, J. J. Heyd, E. Brothers, K. N. Kudin, V. N. Staroverov, R. Kobayashi, J. Normand, K. Raghavachari, A. Rendell, J. C. Burant, S. S. Iyengar, J. Tomasi, M. Cossi, N. Rega, J. M. Millam, M. Klene, J. E. Knox, J. B. Cross, V. Bakken, C. Adamo, J. Jaramillo, R. Gomperts, R. E. Stratmann, O. Yazyev, A. J. Austin, R. Cammi, C. Pomelli, J. W. Ochterski, R. L. Martin, K. Morokuma, V. G. Zakrewski, A. G. Voth, P. Salvador, J. J. Dannenberg, S. Dapprich, A. D. Daniels, O. Farkas, J. B. Foresman, J. V. Ortiz, J. Cioslowski and D. J. Fox, Gaussian 09, Revision D.01, Gaussian, Inc., Wallingford CT, 2013.

[S21] Y. Zhao and D. G. Truhlar, *Theor. Chem. Acc.*, 2008, **120**, 215-241.

[S22] (a) E. Cancès, B. Mennucci and J. Tomasi, *J. Chem. Phys.*, 1997, **107**, 3032-3041; (b) M. Cossi, V. Barone, B. Mennucci and J. Tomasi, *Chem. Phys. Lett.*, 1998, **286**, 253-260; (c) J. Tomasi, B. Mennucci and E. Cancès, *J. Mol.Struc.: THEOCHEM*, 1999,**464**, 211-226.
